# Highly Selective Synthesis of 6-Glyoxylamidoquinoline Derivatives via Palladium-Catalyzed Aminocarbonylation

**DOI:** 10.3390/molecules27010004

**Published:** 2021-12-21

**Authors:** Sami Chniti, László Kollár, Attila Bényei, Attila Takács

**Affiliations:** 1 Department of Inorganic Chemistry, University of Pécs, Ifjúság útja 6, H-7624 Pecs, Hungary; samichniti@yahoo.fr (S.C.); kollar@gamma.ttk.pte.hu (L.K.); 2 János Szentágothai Research Centre, University of Pécs, Ifjúság útja 20, H-7624 Pecs, Hungary; 3 MTA-PTE Research Group for Selective Chemical Syntheses, Ifjúság útja 6, H-7624 Pecs, Hungary; 4 Department of Pharmaceutical Chemistry, University of Debrecen, Egyetem tér 1, H-4032 Debrecen, Hungary; benyei.attila@science.unideb.hu

**Keywords:** double carbonylation, palladium, aminocarbonylation, 6-iodoquinoline, carbon monoxide

## Abstract

The aminocarbonylation of 6-iodoquinoline has been investigated in the presence of large series of amine nucleophiles, providing an efficient synthetic route for producing various quinoline-6-carboxamide and quinoline-6-glyoxylamide derivatives. It was shown, after detailed optimization study, that the formation of amides and ketoamides is strongly influenced by the reaction conditions. Performing the reactions at 40 bar of carbon monoxide pressure in the presence of Pd(OAc)_2_/2 PPh_3_, the corresponding 2-ketocarboxamides were formed as major products (up to 63%). When the monodentate triphenylphosphine was replaced by the bidentate XantPhos, the quinoline-6-carboxamide derivatives were synthesized almost exclusively under atmospheric conditions (up to 98%). The isolation and characterization of the new carbonylated products of various structures were also accomplished. Furthermore, the structure of three new mono- and double-carbonylated compounds were unambiguously established by using a single-crystal XRD study.

## 1. Introduction

The activation of carbon monoxide and its use as a C1 building block in the presence of transition metal complexes is one of the most important stories of homogeneous catalysis. Thus, carbonylation reactions have become an indispensable tool for the synthesis of carboxylic acids and their derivatives (esters, amides, aldehydes and ketones) not only in academic but also in industrial fields [1,2,3,4]. Since the seminal research of Heck et al. [5,6,7], the palladium-catalyzed aminocarbonylation of aryl- and alkenyl halides (and their synthetic surrogates) has been demonstrated to be an effective synthetic approach for the synthesis of carboxamides with various structures [8,9,10,11,12,13,14,15,16,17]. Amides are important class of compounds found in a wide range of chemical products (pesticides, pharmaceuticals, polymers and peptides) [18,19,20], and can be frequently found in pharmaceutically relevant heteroaryl skeletons [21,22,23]. Due to the good functional group tolerance and the high selectivity of the palladium-catalyzed aminocarbonylation, this method could be considered as the most effective one-pot method for introducing the amide motif into the heteroaryl core [24,25,26,27,28,29].

As reported by Ozawa and Yamamoto in 1982 [30], the palladium-catalyzed double-carbonylative amination reaction has emerged as an efficient amidation approach to install the ketoamide moiety, as a captivating *privileged unit* that has a crucial role in modern medicinal chemistry [31,32]. Despite the elevated carbon monoxide pressure that is usually recommended for the production of 2-ketocarboxamides, Han and coworkers described a palladium-catalyzed ligand-free selective double carbonylation method under atmospheric conditions [33]. This special structural motif, also known as *glyoxamide* or *glyoxylamide*, could be involved in several drug candidates conferring to their skeletons important structural features [34] and, consequently, critical pharmacokinetic properties, including metabolic stability in biological medium [35]. Its ability to establish hydrogen bonds and to increase conformational constraints of lead compounds makes it a key component of various new ligand candidates with excellent bioreactivity towards amino acid residues of various receptors and enzymes [31,36,37,38].

Quinoline nucleus, as an alkaloid parental compound, is widely introduced in many potential therapeutic agents. Particularly, many quinoline-based approved drugs exhibiting a broad spectrum of pharmacological effects have been marketed as antimicrobial, antitumor, antimalarial, anti-inflammatory, hypotensive, analgesic or anti-HIV agents [39].

The incorporation of two or more pharmacophores into one simple framework remains one of the efficient strategies in modern organic chemistry and drug design, yielding to original future medicinally relevant lead compounds. In this context, many efforts have focused on the synthesis of *carboxamide- and ketocarboxamide-based quinoline hybrids* using conventional pathways such as classic amidification involving corresponding carboxylic acid forms and amines as coupling partners [40], the aminolysis of quinoline-carboxylic esters [41], the transition-metal-catalyzed direct amidation of methylquinolines and quinolone-carboxaldehyde derivatives, using amines or in situ prepared *N*-chloroamines, respectively [42,43], the selective homolytic carbamoylation of unsubstituted quinoline as a heteroaromatic-type base [44], and Pd-catalyzed aminocarbonylation reactions: an expedient and unique alternative route giving access to both targets *using just one stone* [45,46,47].

The palladium-catalyzed Buchwald–Hartwig amination and Suzuki-coupling reaction have been described in the literature for the functionalization of the quinoline ring at position 6 [48,49]. The 6-iodoquinoline has also successfully been used as a substrate in several palladium-catalyzed carbonylative coupling reactions [50,51,52,53]. However, aminocarbonylation with 6-iodoquinoline derivatives has only been mentioned in a few articles: e.g., 6-iodoquinoline has been successfully converted to quinoline-6-carboxamide using formic acid as a CO surrogate and ammonium hydrogen carbonate as the ammonia source [54].

**Figure 1:**
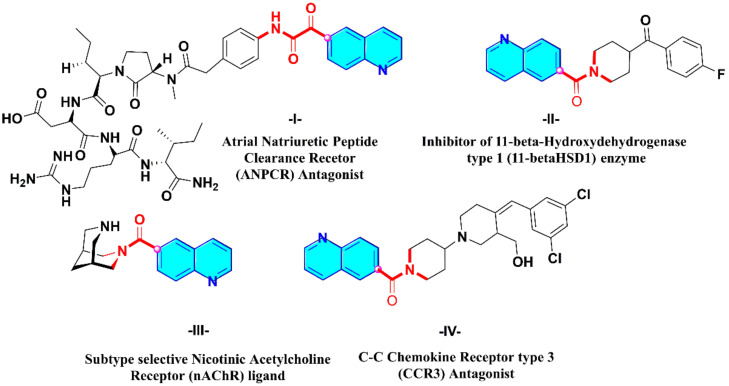
Importance of quinoline-6-carboxamide and quinoline-6-glyoxylamide derivatives: selected examples of biologically active molecules.

Surprisingly, only a few examples of 6-carboxamide and 6-glyoxamide-based quinoline scaffolds have been documented in the literature that makes them good targets for synthetic organic chemists. As examples, *bispidine-carboxamide-6-quinoline* derivative ((1*R*,5*S*)-3,7-diazabicyclo[3.3.1]nonan-3-yl(quinolin-6-yl)methanone) **III**, a selective ligand for nicotinic acetylcholine receptor, could be used in case of central nervous system (CNS) diseases such as depression and addiction. Furthermore, *piperidinylcarboxamide-6-quinolines* (e.g., (4-(4-fluorobenzoyl)piperidin-1-yl)(quinolin-6-yl)methanone (**II**) and (4-(3,5-dichlorobenzylidene)-3-(hydroxymethyl)-[1,4’-bipiperidin]-1’-yl)(quinolin-6-yl)methanone (**IV**)), showing interesting inhibitions of C-C chemokine type 3 and 11β-hydroxydehydrogenase type 1 enzyme, are described for the treatment of metabolic syndrome and as anti-asthmatic agent, respectively. On the other hand, a hybrid *peptido-based-phenylglyoxylamide-6-quinoline* **I** has been patented as a good antagonist to alleviate secondary pulmonary hypertension (Figure 1) [55,56,57,58].

In our research group, several iodoheteroaromatic model substrates have been successfully used in palladium-catalyzed aminocarbonylation to introduce an amide motif into their heteroaryl core, justifying the effectiveness of this homogeneous synthetic method [59,60,61,62,63,64,65]. As a part of our ongoing research, we were interested in the use of the homogeneous catalytic aminocarbonylation of 6-iodoquinoline substrate with various amines in order to design a library of new 6-functionalized quinoline derivatives, keeping in mind our main goal to access to scarce quinoline-6-glyoxylamides that could be the object of future biological investigations.

## 2. Results

### 2.1. Optimization Study

We previously reported [61,66,67] that iodoarene substrates provide a mixture of carboxamide and the 2-ketocarboxamide-type products in aminocarbonylation reactions using Pd(OAc)_2_/2 PPh_3_ catalysts. Therefore, our study began with the optimization of the aminocarbonylation of 6-iodoquinoline (**1**) to find the appropriate circumstances for the selective synthesis of the mono- and dicarbonylated derivatives (Table 1).

Performing the reactions with piperidine (**b**) and tert-butylamine (**a**) under atmospheric conditions (entries 1, 2), the conversion was complete in 24 h, and a mixture of carboxamide (**2a**, **2b**) and 2-ketocarboxamide (**3a**, **3b**) products were formed. Carrying out the reactions in the presence of piperidine (**b**) at 30 °C (entry 3), the product formation was slightly shifted toward the ketoamide derivative (**3b**). It can be seen by GC-MS analysis of the mixture that the reaction was complete in 6 h of reaction time (entry 3). It is also well known that carbon monoxide pressure has great influence on the double carbon monoxide insertion forming the expected 2-ketocarboxamides [68]. For this reason, the aminocarbonylation of 6-iodoquinoline (**1**) with piperidine (**b**) was investigated at elevated CO pressure (10 and 40 bar) (entries 4, 5), and the corresponding 2-ketocarboxamide (**3b**) was formed with high selectivity (75% and 82%) (entries 6, 7). Using primary amines (tert-butylamine (**a**), cyclohexylamine (**c**)) instead of piperidine (**b**) under optimized conditions (entries 8, 9), the expected ketoamide-type products (**3a**, **3c**) were formed with convincing selectivity (>84%).

Since the isolation of the quinoline-6-carboxamide derivatives cannot be accomplished in high yields under the above-mentioned optimized conditions toward the double carbonylated products, we wanted to establish appropriate conditions to achieve their selective and high-yielding synthesis. Carrying out the reactions with piperidine (**b**) in the presence of Pd(OAc)_2_/2 PPh_3_ catalysts under atmospheric conditions at 80 °C, the corresponding carboxamide (**2b**) was formed with 90% selectivity (entry 8). However, when cyclohexylamine (**c**) is used, the selectivity in favor of amide (**2c**) decreases to 70% (entry 9). Based on our previous results [69,70], the triphenylphosphine was replaced by the bidentate XantPhos to increase the selectivity toward the carboxamide. When the aminocarbonylation of 6-iodoquinoline (**1**) with piperidine (**b**) was conducted at 1 bar of CO pressure and at 50 °C in the presence of Pd(OAc)_2_/XantPhos catalysts, the corresponding carboxamide (**2b**) was formed almost exclusively (95%) (entry 10).

### 2.2. Synthesis of Quinoline-6-glyoxylamides Using Various Amine Nucleophiles

With the optimized conditions in hand, (40 bar of CO pressure, Et_3_N, 50 °C, DMF), 6-iodoquinoline (**1**) was reacted with various amine nucleophiles (**a–w**) in aminocarbonylation reactions, providing the expected quinoline-6-glyoxylamide derivatives (**3a–3w**, except aniline **3n**) (Table 2). The well-known Pd(OAc)_2_/PPh_3_ catalysts were used as an effective system for the selective synthesis of the double-carbonylated products. The low-ligated, highly active Pd(0) species was prepared in situ after the reduction of Pd(II) on the influence of the phosphine, while it was oxidized to phosphine oxide [71,72,73].

Using simple primary (**a**, **c**, **f**, **g**) and secondary (**b**, **d**, **e**) amines, the expected 2-ketocarboxamide-type products (**3a**–**3g**) were synthesized with high selectivity (70–94%) (entries 1–7). Although, the pyrrolidine (**d**) showed decreased selectivity (70%) towards the double-carbonylated product (**3d**) (entry 4), nevertheless, the isolation of the target **3d** in good yield (56%) was accomplished. This phenomenon was described by Ozawa et al. during the systematic investigation of the double carbonylation of iodo- and bromobenzene with secondary amines. It has been shown that the steric bulkiness of the secondary amines has a great influence on double carbonylation [74]. As expected, based on our previous results [61,66], the aminocarbonylation carried out in the presence of less basic aniline (**n**) showed perfect chemoselectivity, giving the 6-(N-phenylcarboxamido)quinoline (**2n**) (entry 14).

**Figure 2:**
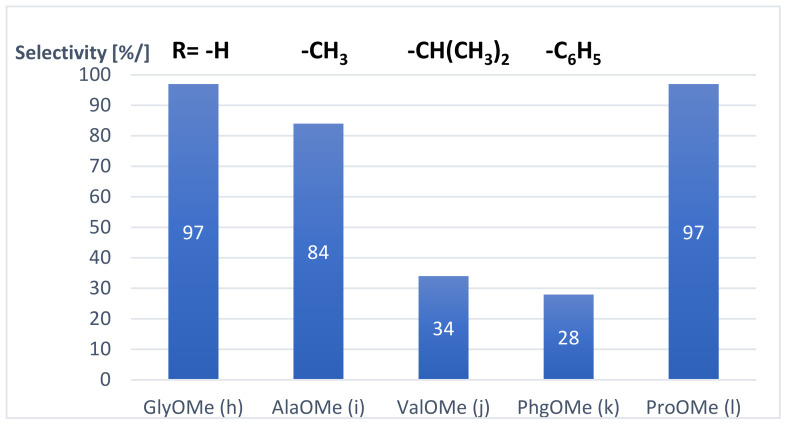
Influence of the side chains of amino acid methyl esters (**h**–**l**) on the selectivity towards the corresponding 2-ketocarboxamides in the aminocarbonylation of 6-iodoquinoline. (Reaction conditions: Pd(OAc)_2_/2 PPh_3_, 40 bar of CO, 50 °C, DMF, Et_3_N).

The effect of amino acid methyl esters (**h**–**;m**) in the aminocarbonylation of 6-iodoquinoline was also investigated. Most of them (except R-(-)-phenylglycine methyl ester (**k**)) reacted well with substrate **1** under optimized conditions, and they provided complete conversion in short reaction times (6–7 h) (entries 8–10, 11–13). It has to be noted that the chemoselectivity towards the 2-ketocarboxamides (**3h**–**3m**) was strongly influenced by the steric properties of the side chain of the amino acids (Figure 2).

While the glycine and alanine methyl ester (**h, i**) gave the expected 6-glyoxyamidoquinoline derivatives (**3h**, **3i**) with high selectivity (entries 8, 9), until then, the chemoselectivity was strikingly decreased (34% and 28%) towards the double-carbonylated products (**3j**, **3k**) by using the valine and the phenylglycine methyl esters (**j**, **k**) (entries 10, 11). This phenomenon can be explained by the steric properties of the side chain of the amino acid methyl esters used in our research: the bulky isopropyl and phenyl substituents bonded to amine **j** and **k** could inhibit the coordination of the second carbon monoxide to the acyl-amido-palladium(II) species, promoting the reductive elimination step of the carboxamide type product during the catalytic cycle. The proline methyl ester (**l**), the secondary amino acid, showed high reactivity, providing the target 2-ketocarboxamide derivative (**3l**) almost exclusively (entry 12). The good functional group tolerance of the palladium-catalyzed aminocarbonylation was also shown by using serine methyl ester (**m**) as an -OH containing amino acid. Complete conversion was observed in a short reaction time (6h) and the corresponding 2-ketocarboxamide (**3m**) was formed as a major product (85%) under 40 bar of carbon monoxide pressure (entry 13). It has to be noted that the -OH motif remained untouched, and no carboxylic-acid-ester-type product was detected by GC analysis.

Aliphatic amines containing a (hetero)aromatic moiety were also used and showed good selectivity towards the target 2-ketocarboxamides. First, benzylamine (**o**) was used, giving the double-carbonylated product (**3o**) in 8 h with 86% selectivity (entry 15). Picolylamines (**p**–**s**) bearing pyridyl moiety were also tested in the aminocarbonylation of 6-iodoquinoline (**1**). 3- and 4-picolylamine (**q**, **r**) showed similar behavior as nucleophile **o**, because high reactivity and perfect 2-ketocarboxamide selectivity were observed (entries 17, 18). As we described previously [75], 2-picolylamine (**p**) showed lower reactivity than **q** and **r**, because its good coordination ability to the central metal could decrease the reaction rate of the oxidative step of the catalytic cycle. In this way, complete conversion was achieved in just 14 h and the selectivity towards the corresponding ketoamide (**3p**) was decreased to 66% (entry 16).

4-(Ethylaminomethyl)pyridine (**s**) as a secondary amine bearing a 4-picolylamine moiety was also used in the aminocarbonylation with 6-iodoquinoline, and a 60/40 mixture of **2s**/**3s** was formed at 40 bar of carbon monoxide pressure. It can be seen that the bulkiness of nucleophile **s**, caused by the ethyl substituent on the N atom, decreased the chemoselectivity towards the expected double-carbonylated product. Additionally, furfurylamine (**t**) and 2-(aminomethyl)thiophene (**u**) were also used as nucleophiles, giving chemoselectively (>95%) the target 2-ketocarboxamide type derivatives (**3t**, **3u**) (entries 20, 21).

Finally, two further N-nucleophiles were chosen to verify the good functional group tolerance of the palladium-catalyzed aminocarbonylation. The -OH moiety of nortropine (**v**) and the alkynyl motif in propargylamine (**w**) did not have any influence on the reactivity, and complete conversion was observed under optimized conditions in a short reaction time (6–7h) in both cases (entries 22, 23). Although the target double-carbonylated products (**3v**, **3w**) were formed with lower selectivity (**3v**—63%, **3w**—78%), the isolation of them was successfully performed.

**Figure 3:**
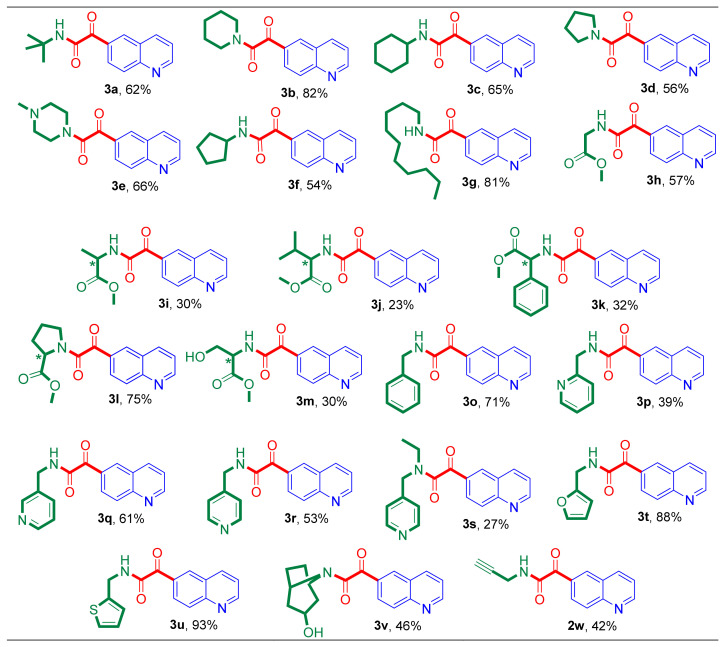
Nucleophile scope of aminocarbonylation: the quinoline-6-glyoxylamides synthesized in the aminocarbonylation of **1** at 40 bar CO pressure using a Pd(OAc)_2_/2 PPh_3_ catalysts (further conditions are mentioned in the footnote of Table 2). (The yields of the isolated target compounds (%), based on the substrate (**1**), are indicated below the structure of the synthesized 2-ketocarboxamide derivatives.)

Overall, our synthetic method showed good tolerance to diverse structures of amines and various functional groups, providing the desired quinoline-6-glyoxylamides with high selectivity. All of the double-carbonylated compounds synthesized in the reactions were isolated in moderate to high yields (Figure 3).

### 2.3. Selective Synthesis of Quinoline-6-carboxamide Derivatives

**Figure 4:**
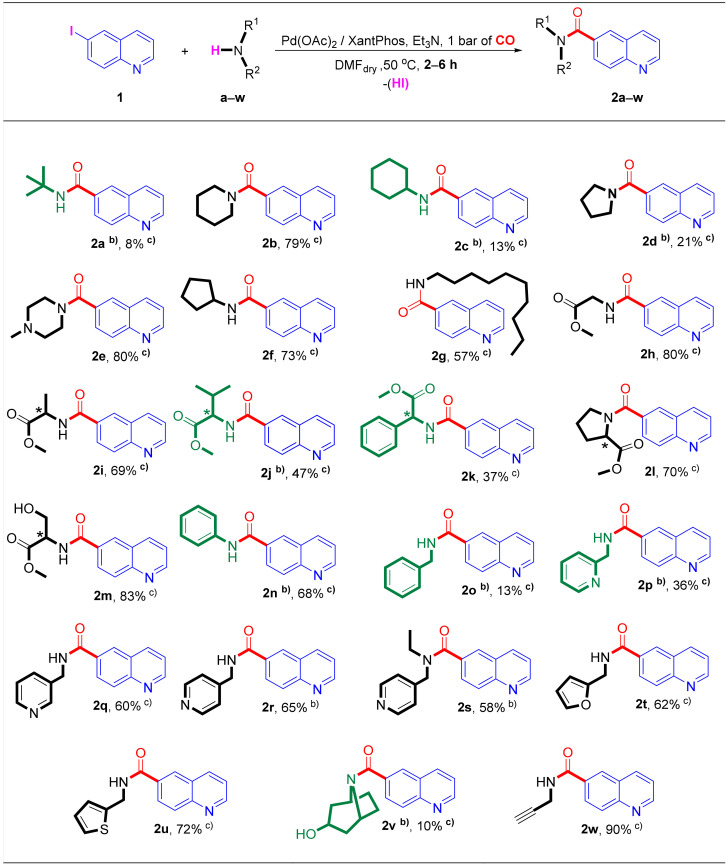
Selective synthesis ofquinoline-6-carboxamide derivatives in the aminocarbonylation of **1** by using Pd(OAc)_2_/XantPhos catalysts under atmospheric conditions. ^(^^a)^ Reaction conditions: 1 mmol of 6-iodoquinoline (**1**), amine nucleophile: 3 mmol of tert-butylamine (**a**) or 1.1 mmol of solid amines or 1.5 mmol of liquid amines, 0.025 mmol of Pd(OAc)_2_, 0.025 mmol of XantPhos, 0.5 mL of Et_3_N, 10 mL of dry DMF, and 1 bar of CO pressure. The reaction was purged with argon and then replaced by carbon monoxide. Complete conversion was detected by GC in most of cases after 6 h (unless specified otherwise). ^(b)^ The mentioned compounds (**2a**, **2j**, **2n**, **2o**, **2p**, and **2v**) were prepared following the first experimental procedure using PPh_3_ as ligand at 40 bar of carbon monoxide pressure. ^(c)^ The isolated yields of target compounds (%), based on the substrate (1), are indicated below the structure of the synthesized quinoline-6-carboxamide derivatives.

The carboxamide type compounds could also be valuable products. Considering the versatile structure of the amines used during our research project, only a few quinoline-6-carboxamide derivatives of them have been mentioned in the literature (**2b** [76], **2n** [77], **2o** [78], **2w** [79]). Although in some cases, the isolation of the 6-carboxamido-quinolines (**2a**, **2c**, **2d**, **2j**, **2n**, **2o**, **2p**, **2v**) was performed with low to moderate yields from the reaction mixture of the aminocarbonylation carried out at 40 bar of carbon monoxide pressure, optimized conditions had to be found for the selective and high yielding synthesis of the target carboxamides. According to our initial study described in Section 2.1, it can be seen that the use of bidentate XantPhos was more efficient than the monodentate triphenylphosphine to get selectively the target carboxamides. Performing the reactions under atmospheric CO pressure at 50 °C in the presence of Pd(OAc)_2_/XantPhos catalysts, the desired quinoline-6-carboxamide derivatives (**2a**–**2w**) were chemoselectively synthesized (Figure 4). It has to be also noted that the corresponding double-carbonylated compounds were not detected even by GC-MS or ^1^H NMR. Under optimized conditions, each amine showed high reactivity, and complete conversion was achieved in a short reaction time (2–6 h). All monocarbonylated derivatives (**2a**–**2w**) were isolated (13–90% yields), justifying that the palladium-catalyzed aminocarbonylation is a powerful method for introducing amide moieties into a heteroaromatic skeleton.

### 2.4. X-ray Cristallographic Study

The crystal and molecular structures of compounds **2c**, **2n**, and **3g** were established unequivocally by single-crystal X- ray diffraction analysis. X-ray quality crystals of each compound were grown from a CHCl_3_ solution upon standing at ambient temperature or from CDCl_3_ solution stored at 4–5 °C. Suitable crystals were chosen by size, habit, and polarized light microscopy and mounted on a loop using high density oil. Intensity data were collected at ambient temperature (294 K) on a Bruker-D8 Venture diffractometer (Bruker AXS GmbH, Karlsruhe, Germany) equipped with INCOATEC IµS 3.0 (Incoatec GmbH, Geesthacht, Germany) dual (Cu and Mo) sealed tube micro sources and a Photon II Charge-Integrating Pixel Array detector (Bruker AXS GmbH, Karlsruhe, Germany) using Mo Kα (λ = 0.71073 Å) radiation. High-multiplicity data collection and integration were performed using APEX3 (version 2017.3-0, Bruker AXS Inc., 2017, Madison, WI, USA) software. Data reduction and multiscan absorption correction were performed using SAINT (version 8.38A, Bruker AXS Inc., 2017, Madison, WI, USA). The structure was solved using direct methods and refined on F^2^ using the SHELXL program [80] incorporated into the APEX3 suite. Non-hydrogen atoms were refined with anisotropic thermal parameters. Hydrogen atoms were placed in idealized positions on parent atoms in the final refinement except N-H protons, which could be found on the difference electron density maps, and N-H distances should have been constrained. The CIF file was manually edited using Publcif software [81], while graphics were prepared using the Olex2 program [82]. The results for the X-ray diffraction structure determinations were very good according to the Checkcif functionality of PLATON software (Utrecht University, Utrecht, the Netherlands) [83]. Details of the crystal parameters, data collection, and structure refinement are given in Table 3. Selected geometric parameters for these structures are given in Table 4. The supplementary crystallographic data for each compound can be obtained free of charge from the Cambridge Crystallographic Data Centre via http://www.ccdc.cam.ac.uk/data_request/cif accessed on 12 October 2021, using reference deposition numbers: **2115317–2115319** for **2c, 2n**, and **3g**, respectively.

**Figure 5:**
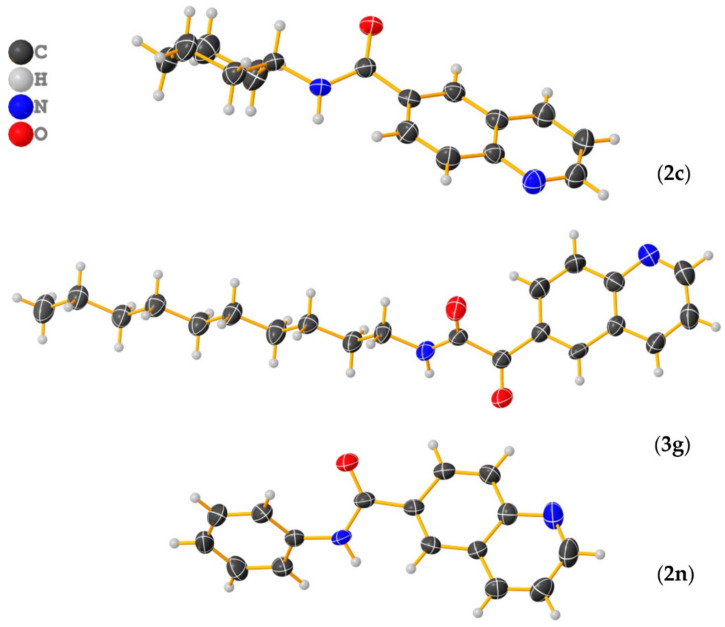
ORTEP style views of *N*-cyclohexylquinoline-6-carboxamide (**2c**), *N*-decyl-2-oxo-2-(quinolin-6-yl)acetamide (**3g**), and *N*-phenylquinoline-6-carboxamide (**2n**), showing thermal displacement ellipsoids, drawn at the 50% probability level (Graphics were designed using Olex2 program.).

The ORTEP style views of **2c**, **3g** and **2n** derivatives can be seen on Figure 5.

**Figure 6:**
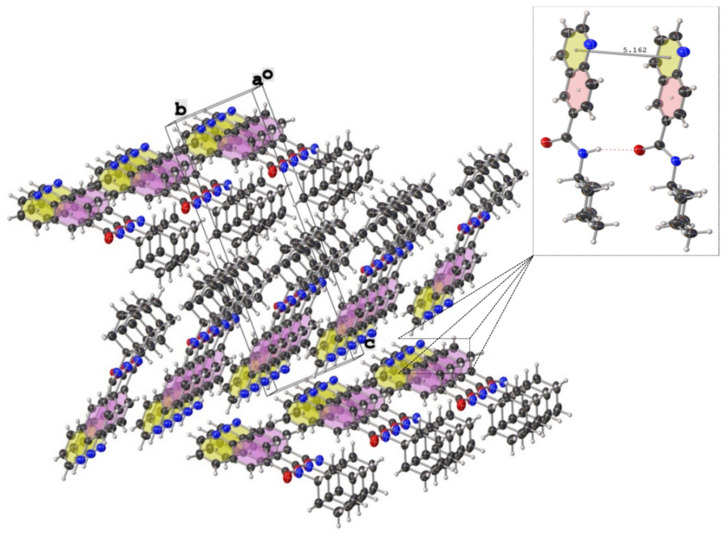
Crystal packing of *N*-cyclohexylquinoline-6-carboxamide (**2c**) showing thermal displacement ellipsoids, drawn at the 50% probability level (view normal to (011) plane).

**Figure 7:**
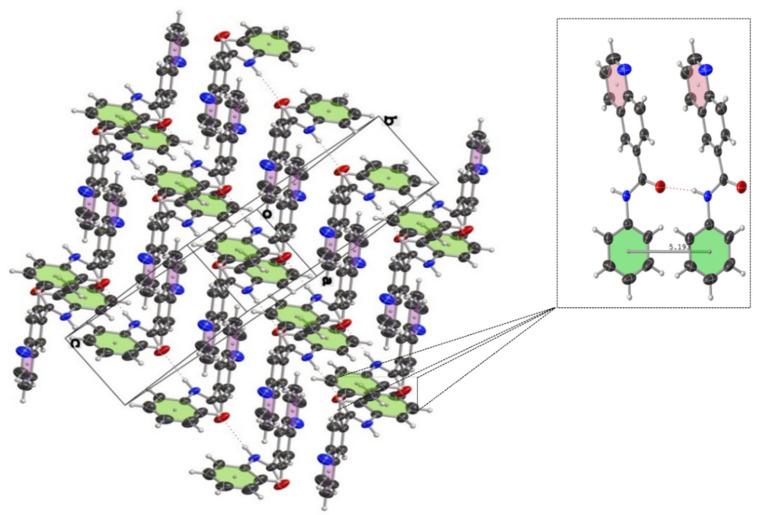
Crystal packing of *N*-phenylquinoline-6-carboxamide (**2n**) showing thermal displacement ellipsoids, drawn at the 50% probability level (view normal to (111) plane). Intermolecular hydrogen bonds are indicated with dashed lines.

As reported in Table 3, compound **2c** crystallized in non-enantiogenic (Sohncke) space group P2_1_. The Platon program and checkcif did not report missed symmetry, and this compound can be a candidate for investigating anisotropy in optical and other physical properties. The high uncertanity and value of the Flack parameter of F = 0.4 (9) may indicate that we have an inversion twin or racemic conglomerate. However, the applied MoKÎą radiation makes it impossible to distinguish between these cases. The *N*-phenylquinoline-6-carboxamide (**2n**) crystallized in the centrosymmetric group P ī with two molecules in the asymmetric unit (Table 3, Z = 4). Again, no additional symmetry was reported by checkcif, although the conformation of the two molecules is very similar. However, considering the packing and hydrogen bonds network (Table 4), minute differences can be detected in the geometric data. These results verifiy that the unit cell and space group originally suggested by the data collection software Apex3 are correct. Interestingly, all three compounds subjected to X-ray diffraction study exhibit pseudo-planar geometries with an important amount of twist of the carbonyl group in the range of 22–35°. At first glance, as it can be seen from the crystal packings for both carboxamidoquinoline derivatives (Figure 6 and Figure 7), the molecules are stacked in two non-equivalent stacks with a slight splitting of the arrangement. In case of (**2n**), the dihedral angle between the mean plane of the quinoline-ring and the mean plane of the phenyl group is 36.7°, while the same deformation is less remarkable in the case of (**2c**). The presence of intermolecular hydrogen bonds between amide fragments (average *d*_(O⋯N)_ ≈ 3.03 Å (Table 4)) gives rise to longer and rigid ‘***zigzag edges***’ in the skeletons.

The interplanar distance of 5.162 Å (**2c**) and 5.193 Å (**2n**) are too far from the ideal centroid-to-centroid distance (3.5 Å) that would enable parallel π⋯π-stacking between the aromatic platforms [84,85]; the arrangement of the rings is shown in Figure 6 and Figure 7.

**Figure 8:**
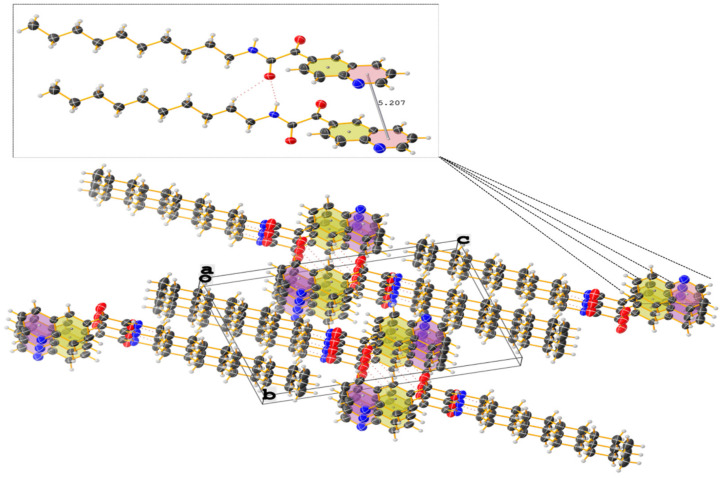
Crystal packing of *N*-decyl-2-oxo-2-(quinolin-6-yl)acetamide (**3g**) showing thermal displacement ellipsoids, drawn at the 50% probability level (view normal to (011) plane). Intermolecular hydrogen bonds are indicated with dashed lines.

Analogously, the crystal structure of the *N*-decyl-2-oxo-2-(quinolin-6-yl)acetamide (**3g**) reveals interesting structural features. The α-ketoamide moiety exhibits the prefered *s-trans*-conformation, where the two carbonyls are in the *trans* position (∠***O-CO-CO-O*** = 138.86°) and are in the same plane as the nitrogen atom. This important twist and the presence of the long, fully elongated hydrocarbon chain (Figure 5) consequently lead to the pyramidalization of the nitrogen center. The aromatic systems are even further from each other (***d_(centroid-to-centroid)_*** = 5.207 Å (Table 4)) (Figure 8). The presence of weak C-H⋯O and N-H⋯O hydrogen bonds (*d*_(CH⋯O)_ ≈ 2.07 Å, *d*_(NH⋯O)_ ≈ 2.24 Å) provides more stability to the solid-state structure.

## 3. Materials and Methods

### 3.1. General Procedures

The ^1^H and ^13^C NMR spectra were recorded in CDCl_3_ or DMSO-d_6_ on a Bruker Avance III 500 spectrometer (Bruker BioSpin Corp., Karlsruhe, Germany) at 500 and 125.7 MHz, respectively. Chemical shifts β are reported in ppm relative to CDCl_3_ (7.26 and 77.00 ppm for ^1^H and ^13^C, respectively) or DMSO-d_6_ (2.50 and 39.50 ppm for ^1^H and ^13^C, respectively). The FT-IR spectra were taken in KBr pellets using a Nicolet IMPACT 400 spectrometer (Thermo Fisher Scientific, Waltham, MA, USA) applying a DTGS detector in the region of 400–4000 cm^−1^; the resolution was 4 cm^−1^. The amount of the samples was ca. 0.5 mg. Shimadzu GC-2030 gas-chromatograph (Shimadzu, Tokyo, Japan) fitted with a capillary column coated with OV-1 (injector temp. 250 °C; oven: starting temp. 50 °C (hold-time 1 min), heating rate 15 °C min^−1^, final temp. 320 °C (hold-time 11 min); detector temp. 280 °C; carrier gas: helium (rate: 1 mL min^−1^)). Mass spectrometry data were recorded using a GC–MS-2020 system (Shimadzu, Tokyo, Japan) operated in EI mode (70 eV). TLC plates (silica gel on TLC Al foils with fluorescence indicator 254 nm) were purchased from Sigma-Aldrich (St. Louis, MO, USA). The eluents used in thin-layer chromatography are specified below.

The Pd(OAc)_2_, the ligands (PPh_3_ and XantPhos), and the solvents were used without further purification. 6-Iodoquinoline (**1**) and the amine nucleophiles (**a**–**w**) were purchased from Sigma-Aldrich and were used without any further purification. The nortropine (**v**) was purchased from TCI Chemicals and was used without further purification. The compounds **2b** [76], **2n** [77], **2o** [78], and **2w** [79] have been described in the literature. In the case of **2b**, **2n**, and **2w**, the spectral data were in accordance with the literature data. In the case of **2o**, the characterization was not complete, and the ^1^H or ^13^C NMR data for these compounds are provided here for the sake of completeness.

### 3.2. Aminocarbonylation of 6-Iodoquinoline (1) in the Presence of Various Amine Nucleophiles (a–w) under Atmospheric Carbon Monoxide Pressure

In a typical experiment, Pd(OAc)_2_ (5.6 mg, 0.025 mmol), triphenylphosphine (13.1 mg, 0.05 mmol) or XantPhos (14.5 mg, 0.025 mmol), 6-iodoquinoline (**1**) substrate (1 mmol), amine nucleophiles (amount of the amines are given in Table 2 and Table 4) and triethylamine (0.5 mL) were dissolved in DMF (10 mL) under argon in a 100 mL three-necked flask equipped with reflux condenser connected to a balloon filled with argon. The atmosphere was changed to carbon monoxide. The reaction was conducted for the given reaction time upon stirring at 50 °C and analyzed by GC and GC-MS. The cooled reaction mixture was then concentrated and evaporated to dryness under reduced pressure. The residue was dissolved in chloroform (20 mL) and washed twice with water (20 mL). The organic phase was dried over Na_2_SO_4_, filtered, and evaporated under reduced pressure to a solid material. All compounds were subjected to column chromatography (Silicagel 60 (Sigma), 0.063–0.200 mm), CHCl_3_/MeOH, or CHCl_3_/EtOAc eluent mixtures (the exact ratios are specified in the Characterization section found in the Supplementary file).

### 3.3. Aminocarbonylation of 6-Iodoquinoline (1) in the Presence of Various Amine Nucleophiles (a–w) under High Carbon Monoxide Pressure

In a typical experiment, Pd(OAc)_2,_ triphenylphosphine, 6-iodoquinoline (**1**), the amine nucleophiles (**a–w**), and triethylamine were used in the same amount as above and were dissolved in 10 mL of DMF under argon in a 100 mL autoclave. The atmosphere was changed to carbon monoxide and the autoclave was pressurized to the given pressure with carbon monoxide. (Caution: high-pressure carbon monoxide should only be used with adequate ventilation (hood) and while also using CO sensors.) The reaction was conducted for the given reaction time upon stirring at 50 °C. After the given reaction time, the reaction mixture was cooled to room temperature and the autoclave was carefully depressurized in a well-ventilated hood. The product mixture was analyzed using GC and GC-MS. The work-up of the reaction mixture was identical to that discussed for the atmospheric experiments.

## 4. Conclusions

In summary, the quinoline skeleton was functionalized at position 6 via palladium-catalyzed aminocarbonylation in the presence of amines with various structures, providing several quinoline-6-carboxamides and quinoline-6-glyoxylamides. The selectivity of the reaction was strikingly affected by the ligand and the carbon monoxide pressure used during the reactions.

It was shown that the aminocarbonylation of 6-iodoquinoline provides more than 82% selectivity of the target double-carbonylated derivatives in almost all cases under 40 bar of carbon monoxide pressure in the presence of a Pd(OAc)_2_/2 PPh_3_ catalyst. In some cases, the selectivity towards the 2-ketocarboxamides decreased (28–66%) due to the steric hindrance of the amine nucleophiles (**j**, **k**, **p**, **s**, **v**). It has to be noted that a primary aromatic amine (aniline (**n**)) provided the 6-(*N*-phenylcarboxamido)quinoline (2**n**) product exclusively and the ketoamide (**3n**) was not detected by GC-MS analysis. The 22 novel quinoline-6-glyoxylamide derivatives (**3a**–**3w**) synthesized in the aminocarbonylation of **1** were isolated and fully characterized. In this way, a novel one-pot synthetic approach regarding valuable quinoline-6-glyoxylamides was developed, the synthesis of which is quite difficult using conventional organic synthetic methods.

Using the bidentate XantPhos instead of triphenylphosphine under atmospheric carbon monoxide pressure, the synthesis of quinoline-6-carboxamide was also achieved. The target amides (**2a**–**2w**) were successfully produced via palladium-catalyzed aminocarbonylation. Under these conditions, the reactions showed extremely high chemoselectivity, exclusively giving the desired monocarbonylated products.

The molecular structure of some products (**2c**, **2n**, and **3g**) was unambiguously supported by single-crystal X-ray diffraction study. The novel 2-ketocarboxamides and carboxamides synthesized and isolated in this work could have biological and practical relevance. The perfect selectivity toward the target compounds and the good isolated yields mean the synthetic importance of these reactions.

## Figures and Tables

**Table 1:** Optimization of the aminocarbonylation of 6-iodoquinoline (**1**) ^(a)^.

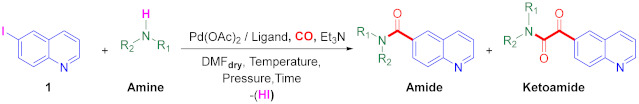						
Entry	Amine	Ligand	Temp. (°C)	pCO (bar)	Ratio of the Carbonylated Products ^(b)^	
					Amide	Ketoamide
**1**	Piperidine (**b**)	PPh_3_	50	1	63	37
**2**	*tert*-Butylamine ^(c)^ (**a**)	PPh_3_	50	1	40	60
**3**	Piperidine (**b**)	PPh_3_	30	1	33	66
**4**	Piperidine (**b**)	PPh_3_	80	1	90	10
**5**	Cyclohexylamine (**c**)	PPh_3_	80	1	70	30
**6**	Piperidine (**b**)	PPh_3_	50	10	25	75
**7**	Piperidine (**b**)	PPh_3_	50	40	18	82
**8**	*tert*-Butylamine (**a**)	PPh_3_	50	40	6	94
**9**	Cyclohexylamine (**c**)	PPh_3_	50	40	16	84
**10**	Piperidine (**b**)	XantPhos	50	1	95	5

^(a)^ Reaction conditions: 1 mmol of 6-iodoquinoline (**1**), 3.0 mmol of tert-butylamine (**a**) or 1.5 mmol of piperidine (**b**) and cyclohexylamine (**c**), 0.025 mmol of Pd(OAc)_2_, 0.05 mmol of PPh_3_ or 0.025 mmol of Xantphos, 0.5 mL of Et_3_N, 10 mL of DMF, at the mentioned temperature. The reaction was purged with argon and then replaced by carbon monoxide. Complete conversion was detected in 6 h. ^(b)^ Determined by GC analysis of the reaction mixture. ^(c)^ The conversion was complete in 19 h.

**Table 2:** Synthesis of quinoline-6-glyoxylamide derivatives ^(a)^.

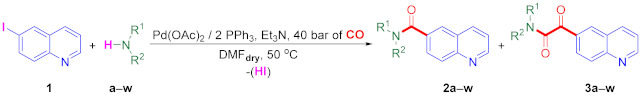				
Entry	Amine	R. Time (h) ^(b)^	Ratio of the Carbonylated Products ^(c)^	
			Amide	Ketoamide
**1**	*tert*-Butylamine (**a**)	19	6 (**2a**)	94 (**3a**)
**2**	Piperidine (**b**)	6	18 (**2b**)	82 (**3b**)
**3**	Cyclohexylamine (**c**)	6	16 (**2c**)	84(**3c**)
**4**	Pyrrolidine (**d**)	6	30 (**2d**)	70 (**3d**)
**5**	N-methylpiperazine (**e**)	6	6 (**2e**)	94 (**3e**)
**6**	Cyclopentylamine (**f**)	6	14 (**2f**)	86 (**3f**)
**7**	Decylamine (**g**)	6	13 (**2g**)	87 (**3g**)
**8**	L-Glycine methyl ester (**h**)	7	3 (**2h**)	97(**3h**)
**9**	L-Alanine methy lester (**i**)	6	16 (**2i**)	84 (**3i**)
**10**	L-Valine methyl ester (**j**)	7	66 (**2j**)	34 (**3j**)
**11**	R-(-)-2-Phenylglycine methyl ester (**k**)	24	72 (**2k**)	28 (**3k**)
**12**	L-Proline methyl ester (**l**)	6	7 (**2l**)	93 (**3l**)
**13**	(L/D)-Serine methyl ester (**m**)	6	15 (**2m**)	85 (**3m**)
**14**	Aniline (**n**)	8	100 (**2n**)	0(**3n**)
**15**	Benzylamine (**o**)	8	14 (**2o**)	86(**3o**)
**16**	2-(aminomethyl)pyridine (**p**)	14	33 (**2p**)	66(**3 p**)
**17**	3-(aminomethyl)pyridine (**q**)	6	3 (**2q**)	97(**3q**)
**18**	4-(aminomethyl)pyridine (**r**)	6	2 (**2r**)	98(**3r**)
**19**	4-(ethylaminomethyl)pyridine (**s**)	24	60 (**2s**)	40 (**3s**)
**20**	Furfyrlamine (**t**)	6	5 (**2t**)	95(**3t**)
**21**	2-(Aminomethyl)thiophene (**u**)	6	2(**2u**)	98(**3u**)
**22**	Nortropine (**v**)	7	37 (**2v**)	63(**3v**)
**23**	Propargylamine (**w**)	6	15 (**2w**)	85 (**3w**)

^(a)^ Reaction conditions: 1 mmol of 6-iodoquinoline (**1**), amine nucleophile: 3 mmol of tert-butylamine (**a**) or 1.1 mmol of solid amines or 1.5 mmol of liquid amines, 0.025 mmol of Pd(OAc)_2_, 0.05 mmol of PPh_3_, 0.5 mL of Et_3_N, 10 mL of dry DMF, and 40 bar of CO pressure. The reaction was purged with argon and then replaced by carbon monoxide.^(b)^ Complete conversion detected by GC in the given time.^(c)^ Ratio based on GC-MS analysis.

**Table 3:** Crystallographic parameters and refinement details for (**2c**), (**2n**), and (**3g**).

Compounds	(2n)	(2c)	(3g)
Chemical formula	C_16_H_18_N_2_O	2(C_16_H_12_N_2_O)	C_21_H_28_N_2_O_2_
M_r_	254.32	248.28	340.45
Crystal system	Monoclinic	Triclinic	Triclinic
Space group	P2_1_	P ī	P ī
Temperature (K)	294	294	300
a, b, c (Å)	5.1616 (4), 6.6616 (5),19.2655 (14)	5.1930 (4), 9.0314 (7), 27.180 (2)	5.2074 (9), 11.0289 (18), 16.859 (3)
α, β, γ (°)	90, 92.600 (3), 90	98.887 (4), 93.763 (4), 97.281 (4)	79.495 (10), 86.693 (11), 89.903 (9)
V (Å^3^)	661.75 (9)	1244.55 (17)	950.4 (3)
Z	2	4	2
Radiation type	Mo Kα		
μ (mm^−1^)	0.08	0.09	0.08
Crystal size(mm)	0.30 × 0.17 × 0.10	0.38 × 0.19 × 0.04	0.54 × 0.17 × 0.07
Data collection			
Diffractometer	Bruker D8 VENTURE		
Absorption correction	Multi-scan SADABS2016/2—Bruker AXS area detector scaling and absorption correction		
T_min_, T_max_	0.91, 0.99	0.74, 1.00	0.42, 0.99
No. of measured, independent and observed [I > 2σ(I)] reflections 7476, 2725, 2250 41129, 4550, 3249 20248, 3507, 2145			
R_int_	0.038	0.140	0.193
(sin θ/λ)_max_ (Å^−1^)	0.626	0.604	0.609
Refinement			
R[F^2^ > 2s(F^2^)], wR(F^2^), S	0.052, 0.141, 1.15	0.099, 0.208, 1.17	0.143, 0.359, 1.13
No. of reflections	2725	4550	3507
No. of parameters	176	350	232
No. of restraints	2	2	1
H-atom treatment	H atoms treated by a mixture of independent and constrained refinement		
Δ>_max_, Δ>_min_ (e Å^−3^)	0.51, −0.60	0.26, −0.22	0.73, −0.59

**Table 4:** Summary of some selected geometric parameters interatomic distances, interplanar spacing and torsion angles for (**2c**), (**2n**), and (**3g**).

Crystals		(2c)	(2n)	(3g)
***Intermolecular Hydrogen Bonds* (N-H)^i^⋯(O=C)^ii^**	**N⋯O**/Å	3.020	3.019	3.046
	**N-H⋯O**/Å	2.188	2.177	2.24
	**∠N-H⋯O**/°	161.23	161.81	158.34
***Dihedral Angles***	**∠C_Ar_-C_Ar_-C_co_-N**/°	153.30	−143.28	-
	**∠C_Ar_-C_Ar_-C_co_-O**/°	149.08	142.77	157.15
	**∠O-C_co_-C_co_-O**/°	-	-	138.86
***Interplanar distances* (Å)** ***I(d(centroid-to-centroid))***		5.162	5.193	5.207

Symmetry code: **2c**: (i) x + 1, y, z; **2n**: (i) x − 1, y, z and (ii) x + 1, y, z; **3g**: (i) x − 1, y, z.

## Data Availability

The data presented in this study are available in the Supplementary Materials.

## Supplementary Materials

The following are available online. ^1^H and ^13^C NMR spectra of the products synthesized in this work are available online. The X-ray data of products **2c**, **2n**, and **3g** are also available online.

## Sample Availability

Samples of the compounds are not available from the authors.
