# Supplementary material for: Highly Selective Synthesis of 6-Glyoxylamidoquinoline Derivatives via Palladium-Catalyzed Aminocarbonylation
Source: Molecules. 2021 Dec 21;27(1):4. doi: 10.3390/molecules27010004 (PMC8746719; doi:10.3390/molecules27010004)
Supplement: Supplementary file 1 [file molecules-27-00004-s001.zip › cif files&checkcif reports/checkcif_2c.pdf]

No syntax errors found.
Please wait while processing ....

CIF dictionary
Interpreting this report

Datablock: 2c

Bond precision: C-C = 0.0053 A Wavelength=0.71073

Cell: a=5.1616(4) b=6.6616(5) c=19.2655(14)
alpha=90 beta=92.600(3) gamma=90

Temperature: 294 K

	Calculated	Reported
Volume	661.75(9)	661.75(9)
Space group	P 21	P 1 21 1
Hall group	P 2yb	P 2yb
Moiety formula	C16 H18 N2 O	C16 H18 N2 O
Sum formula	C16 H18 N2 O	C16 H18 N2 O
Mr	254.32	254.32
Dx,g cm-3	1.276	1.276
Z	2	2
Mu (mm-1)	0.081	0.081
F000	272.0	272.0
F000'	272.10	
h,k,lmax	6,8,24	6,8,24
Nref	2737[1491]	2725
Tmin,Tmax	0.984,0.992	0.910,0.990
Tmin'	0.976	

Correction method= # Reported T Limits: Tmin=0.910 Tmax=0.990 AbsCorr = MULTI-SCAN

Data completeness= 1.83/1.00 Theta(max)= 26.410

R(reflections)= 0.0524(2250) wR2(reflections)= 0.1409(2725)

S = 1.149 Npar= 176

The following ALERTS were generated. Each ALERT has the format
test-name_ALERT_alert-type_alert-level.
Click on the hyperlinks for more details of the test.

Alert level A

PLAT939_ALERT_3_A Large Value of Not (SHELXL) Weight Optimized S . 1162.40 Check

Alert level C

STRVA01_ALERT_4_C Flack test results are ambiguous.
From the CIF: _refine_ls_abs_structure_Flack 0.400
From the CIF: _refine_ls_abs_structure_Flack_su 0.900
PLAT340_ALERT_3_C Low Bond Precision on C-C Bonds .... 0.00531 Ang.
PLAT906_ALERT_3_C Large K Value in the Analysis of Variance ...... 2.090 Check
PLAT918_ALERT_3_C Reflection(s) with I(obs) much Smaller I(calc) . 1 Check
PLAT934_ALERT_3_C Number of (Iobs-Icalc)/Sigma(W) > 10 Outliers .. 1 Check
PLAT977_ALERT_2_C Check Negative Difference Density on H10 -0.31 eA-3
PLAT977_ALERT_2_C Check Negative Difference Density on H14A -0.33 eA-3

Alert level G

PLAT002_ALERT_2_G Number of Distance or Angle Restraints on AtSite 2 Note
PLAT032_ALERT_4_G Std. Uncertainty on Flack Parameter Value High . 0.900 Report
PLAT172_ALERT_4_G The CIF-Embedded .res File Contains DFIX Records 1 Report
PLAT860_ALERT_3_G Number of Least-Squares Restraints .... 2 Note
PLAT910_ALERT_3_G Missing # of FCF Reflection(s) Below Theta(Min). 2 Note
PLAT978_ALERT_2_G Number C-C Bonds with Positive Residual Density. 0 Info

1 **ALERT level A** = Most likely a serious problem - resolve or explain
0 **ALERT level B** = A potentially serious problem, consider carefully
7 **ALERT level C** = Check. Ensure it is not caused by an omission or oversight
6 **ALERT level G** = General information/check it is not something unexpected

0 ALERT type 1 CIF construction/syntax error, inconsistent or missing data
4 ALERT type 2 Indicator that the structure model may be wrong or deficient
7 ALERT type 3 Indicator that the structure quality may be low

```
3 ALERT type 4 Improvement, methodology, query or suggestion
0 ALERT type 5 Informative message, check
```

It is advisable to attempt to resolve as many as possible of the alerts in all categories. Often the minor alerts point to easily fixed oversights, errors and omissions in your CIF or refinement strategy, so attention to these fine details can be worthwhile. In order to resolve some of the more serious problems it may be necessary to carry out additional measurements or structure refinements. However, the purpose of your study may justify the reported deviations and the more serious of these should normally be commented upon in the discussion or experimental section of a paper or in the "special_details" fields of the CIF. checkCIF was carefully designed to identify outliers and unusual parameters, but every test has its limitations and alerts that are not important in a particular case may appear. Conversely, the absence of alerts does not guarantee there are no aspects of the results needing attention. It is up to the individual to critically assess their own results and, if necessary, seek expert advice.

Publication of your CIF in IUCr journals

A basic structural check has been run on your CIF. These basic checks will be run on all CIFs submitted for publication in IUCr journals (*Acta Crystallographica*, *Journal of Applied Crystallography*, *Journal of Synchrotron Radiation*); however, if you intend to submit to *Acta Crystallographica Section C* or *E* or *IUCrData*, you should make sure that full publication checks are run on the final version of your CIF prior to submission.

Publication of your CIF in other journals

Please refer to the *Notes for Authors* of the relevant journal for any special instructions relating to CIF submission.

PLATON version of 13/07/2021; check.def file version of 13/07/2021

Datablock 2c - ellipsoid plot

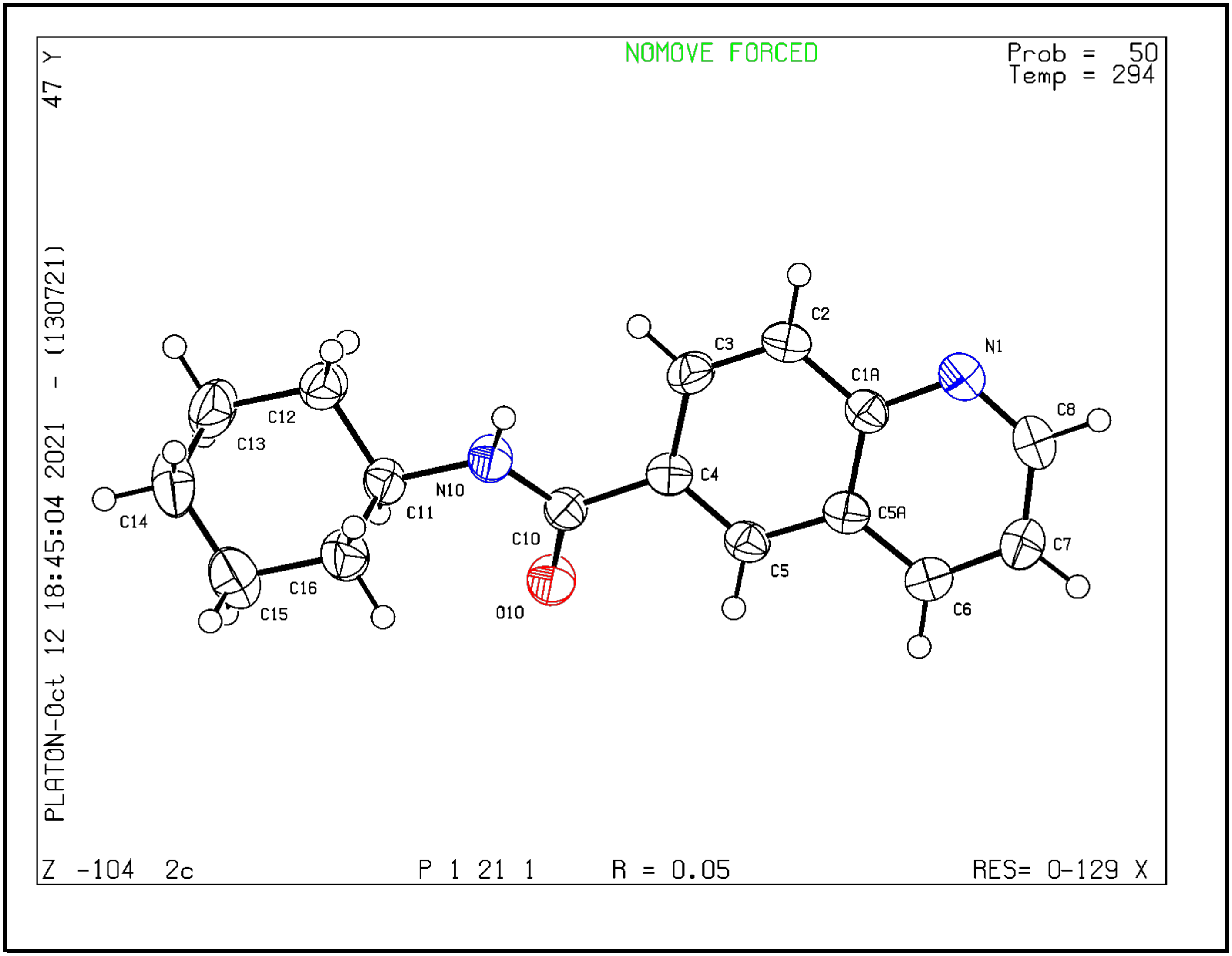

[Download CIF editor (publCIF) from the IUCr](#)
[Download CIF editor (enCIFer) from the CCDC](#)
[Test a new CIF entry](#)
