# Supplementary material for: Highly Selective Synthesis of 6-Glyoxylamidoquinoline Derivatives via Palladium-Catalyzed Aminocarbonylation
Source: Molecules. 2021 Dec 21;27(1):4. doi: 10.3390/molecules27010004 (PMC8746719; doi:10.3390/molecules27010004)
Supplement: Supplementary file 1 [file molecules-27-00004-s001.zip › cif files&checkcif reports/checkcif_2n.pdf]

No syntax errors found.
Please wait while processing ....

CIF dictionary
Interpreting this report

Datablock: 2n

Bond precision:	C-C = 0.0064 A		Wavelength=0.71073
Cell:	a=5.1930(4)	b=9.0314(7)	c=27.180(2)
	alpha=98.887(4)	beta=93.763(4)	gamma=97.281(4)
Temperature: 294 K			

	Calculated	Reported
Volume	1244.55(17)	1244.55(17)
Space group	P -1	P -1
Hall group	-P 1	-P 1
Moiety formula	C16 H12 N2 O	2(C16 H12 N2 O)
Sum formula	C16 H12 N2 O	C16 H12 N2 O
Mr	248.28	248.28
Dx,g cm-3	1.325	1.325
Z	4	4
Mu (mm-1)	0.085	0.085
F000	520.0	520.0
F000'	520.20	
h,k,lmax	6,10,32	6,10,32
Nref	4586	4550
Tmin,Tmax	0.981,0.997	0.740,1.000
Tmin'	0.968	

Correction method= # Reported T Limits: Tmin=0.740 Tmax=1.000 AbsCorr = MULTI-SCAN

Data completeness= 0.992 Theta(max)= 25.410

R(reflections)= 0.0991(3249) wR2(reflections)= 0.2079(4550)

S = 1.170 Npar= 350

The following ALERTS were generated. Each ALERT has the format
test-name_ALERT_alert-type_alert-level.
Click on the hyperlinks for more details of the test.

Alert level C

RINTA01_ALERT_3_C The value of Rint is greater than 0.12
Rint given 0.140
PLAT020_ALERT_3_C The Value of Rint is Greater Than 0.12 .... 0.140 Report
PLAT340_ALERT_3_C Low Bond Precision on C-C Bonds .... 0.00641 Ang.
PLAT480_ALERT_4_C Long H...A H-Bond Reported H8 ..N51 . 2.66 Ang.
PLAT906_ALERT_3_C Large K Value in the Analysis of Variance ...... 31.511 Check
And 2 other PLAT906 Alerts
More ...
PLAT911_ALERT_3_C Missing FCF Refl Between Thmin & STh/L= 0.600 23 Report

Alert level G

FORMU01_ALERT_1_G There is a discrepancy between the atom counts in the _chemical_formula_sum and _chemical_formula_moiety. This is usually due to the moiety formula being in the wrong format.
Atom count from _chemical_formula_sum: C16 H12 N2 O1
Atom count from _chemical_formula_moiety:C32 H24 N4 O2
PLAT002_ALERT_2_G Number of Distance or Angle Restraints on AtSite 4 Note
PLAT042_ALERT_1_G Calc. and Reported Moiety Formula Strings Differ Please Check
PLAT154_ALERT_1_G The s.u.'s on the Cell Angles are Equal ..(Note) 0.004 Degree
PLAT172_ALERT_4_G The CIF-Embedded .res File Contains DFIX Records 1 Report
PLAT860_ALERT_3_G Number of Least-Squares Restraints .... 2 Note
PLAT910_ALERT_3_G Missing # of FCF Reflection(s) Below Theta(Min). 2 Note
PLAT912_ALERT_4_G Missing # of FCF Reflections Above STh/L= 0.600 13 Note
PLAT933_ALERT_2_G Number of OMIT Records in Embedded .res File ... 23 Note
PLAT978_ALERT_2_G Number C-C Bonds with Positive Residual Density. 1 Info

0 **ALERT level A** = Most likely a serious problem - resolve or explain
0 **ALERT level B** = A potentially serious problem, consider carefully
8 **ALERT level C** = Check. Ensure it is not caused by an omission or oversight

```
10 ALERT level G = General information/check it is not something unexpected

 3 ALERT type 1 CIF construction/syntax error, inconsistent or missing data
 3 ALERT type 2 Indicator that the structure model may be wrong or deficient
 9 ALERT type 3 Indicator that the structure quality may be low
 3 ALERT type 4 Improvement, methodology, query or suggestion
 0 ALERT type 5 Informative message, check
```

It is advisable to attempt to resolve as many as possible of the alerts in all categories. Often the minor alerts point to easily fixed oversights, errors and omissions in your CIF or refinement strategy, so attention to these fine details can be worthwhile. In order to resolve some of the more serious problems it may be necessary to carry out additional measurements or structure refinements. However, the purpose of your study may justify the reported deviations and the more serious of these should normally be commented upon in the discussion or experimental section of a paper or in the "special_details" fields of the CIF. checkCIF was carefully designed to identify outliers and unusual parameters, but every test has its limitations and alerts that are not important in a particular case may appear. Conversely, the absence of alerts does not guarantee there are no aspects of the results needing attention. It is up to the individual to critically assess their own results and, if necessary, seek expert advice.

Publication of your CIF in IUCr journals

A basic structural check has been run on your CIF. These basic checks will be run on all CIFs submitted for publication in IUCr journals (*Acta Crystallographica*, *Journal of Applied Crystallography*, *Journal of Synchrotron Radiation*); however, if you intend to submit to *Acta Crystallographica Section C* or *E* or *IUCrData*, you should make sure that full publication checks are run on the final version of your CIF prior to submission.

Publication of your CIF in other journals

Please refer to the *Notes for Authors* of the relevant journal for any special instructions relating to CIF submission.

PLATON version of 13/07/2021; check.def file version of 13/07/2021

Datablock 2n - ellipsoid plot

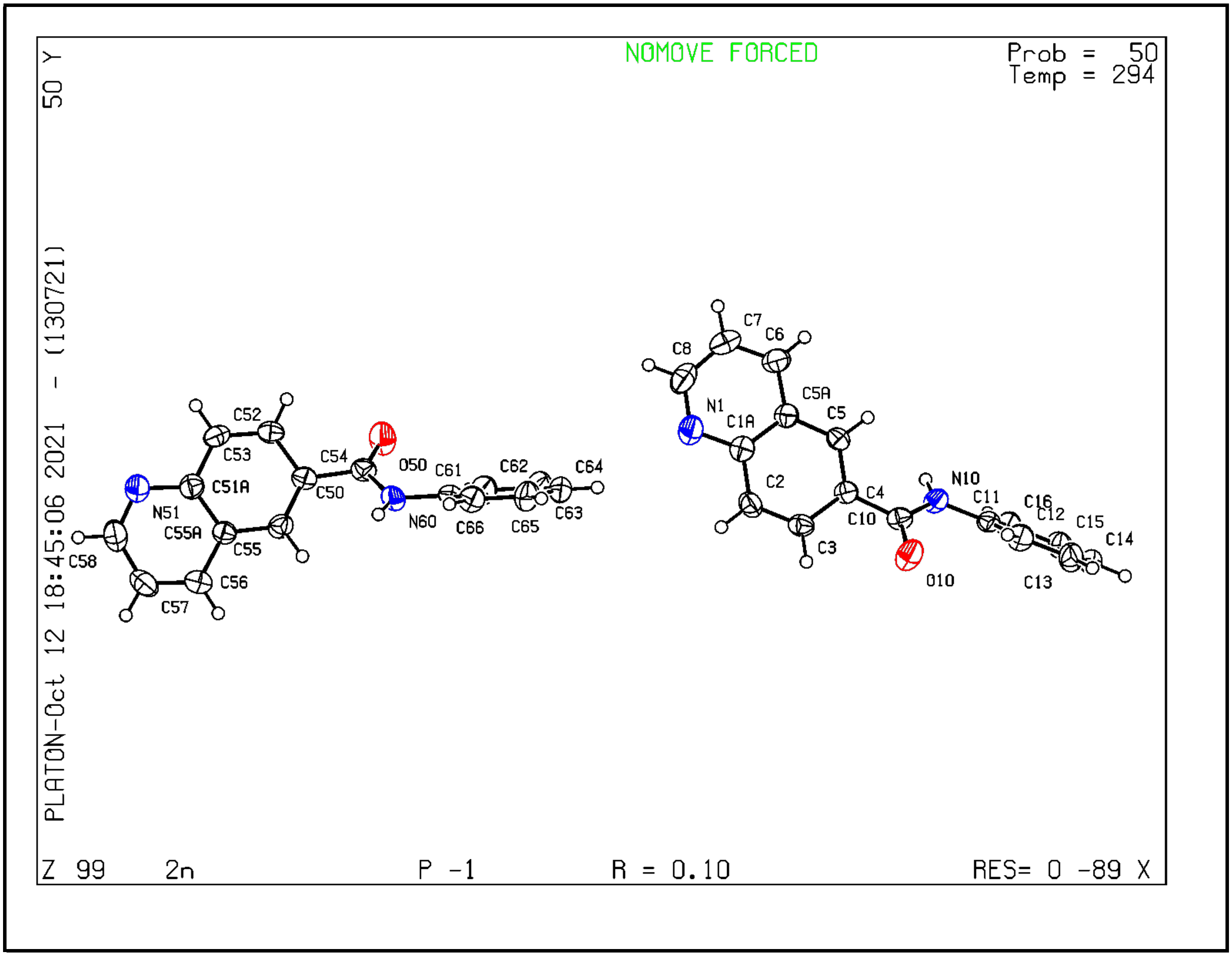

Download CIF editor (publCIF) from the IUCr
Download CIF editor (enCIFer) from the CCDC
Test a new CIF entry
