# Supplementary material for: Highly Selective Synthesis of 6-Glyoxylamidoquinoline Derivatives via Palladium-Catalyzed Aminocarbonylation
Source: Molecules. 2021 Dec 21;27(1):4. doi: 10.3390/molecules27010004 (PMC8746719; doi:10.3390/molecules27010004)
Supplement: Supplementary file 1 [file molecules-27-00004-s001.zip › cif files&checkcif reports/checkcif_3g.pdf]

No syntax errors found.
Please wait while processing ....

CIF dictionary
Interpreting this report

Datablock: 3g

Bond precision: C-C = 0.0070 A Wavelength=0.71073

Cell: a=5.2074(9) b=11.0289(18) c=16.859(3)
alpha=79.495(10) beta=86.693(11) gamma=89.903(9)

Temperature: 300 K

	Calculated	Reported
Volume	950.4(3)	950.4(3)
Space group	P -1	P -1
Hall group	-P 1	-P 1
Moiety formula	C21 H28 N2 O2	C21 H28 N2 O2
Sum formula	C21 H28 N2 O2	C21 H28 N2 O2
Mr	340.45	340.45
Dx,g cm-3	1.190	1.190
Z	2	2
Mu (mm-1)	0.076	0.076
F000	368.0	368.0
F000'	368.15	
h,k,lmax	6,13,20	6,13,20
Nref	3620	3507
Tmin,Tmax	0.985,0.995	0.420,0.990
Tmin'	0.960	

Correction method= # Reported T Limits: Tmin=0.420 Tmax=0.990 AbsCorr = MULTI-SCAN

Data completeness= 0.969 Theta(max)= 25.670

R(reflections)= 0.1429(2145) wR2(reflections)= 0.3591(3507)

S = 1.130 Npar= 232

The following ALERTS were generated. Each ALERT has the format
test-name_ALERT_alert-type_alert-level.
Click on the hyperlinks for more details of the test.

Alert level B

RINTA01_ALERT_3_B The value of Rint is greater than 0.18
Rint given 0.193
PLAT020_ALERT_3_B The Value of Rint is Greater Than 0.12 .... 0.193 Report
PLAT084_ALERT_3_B High wR2 Value (i.e. > 0.25) .... 0.36 Report

Alert level C

DIFMX02_ALERT_1_C The maximum difference density is > 0.1*ZMAX*0.75
The relevant atom site should be identified.
PLAT029_ALERT_3_C _diffrn_measured_fraction_theta_full value Low . 0.969 Why?
PLAT082_ALERT_2_C High R1 Value .... 0.14 Report
PLAT097_ALERT_2_C Large Reported Max. (Positive) Residual Density 0.73 eA-3
PLAT340_ALERT_3_C Low Bond Precision on C-C Bonds .... 0.00705 Ang.
PLAT369_ALERT_2_C Long C(sp2)-C(sp2) Bond C10 - C11 . 1.54 Ang.
PLAT906_ALERT_3_C Large K Value in the Analysis of Variance ...... 26.317 Check
And 2 other PLAT906 Alerts
More ...
PLAT911_ALERT_3_C Missing FCF Refl Between Thmin & STh/L= 0.600 26 Report
PLAT975_ALERT_2_C Check Calcd Resid. Dens. 1.00A From O10 0.58 eA-3
PLAT975_ALERT_2_C Check Calcd Resid. Dens. 0.92A From N1 0.58 eA-3
PLAT977_ALERT_2_C Check Negative Difference Density on H70A -0.36 eA-3

Alert level G

PLAT002_ALERT_2_G Number of Distance or Angle Restraints on AtSite 2 Note
PLAT072_ALERT_2_G SHELXL First Parameter in WGHT Unusually Large 0.20 Report
PLAT172_ALERT_4_G The CIF-Embedded .res File Contains DFIX Records 1 Report
PLAT860_ALERT_3_G Number of Least-Squares Restraints .... 1 Note
PLAT910_ALERT_3_G Missing # of FCF Reflection(s) Below Theta(Min). 3 Note
PLAT912_ALERT_4_G Missing # of FCF Reflections Above STh/L= 0.600 85 Note
PLAT913_ALERT_3_G Missing # of Very Strong Reflections in FCF .... 2 Note

```
PLAT933_ALERT_2_G Number of OMIT Records in Embedded .res File ...         25 Note
PLAT978_ALERT_2_G Number C-C Bonds with Positive Residual Density.          2 Info
```

```
  0 ALERT level A = Most likely a serious problem - resolve or explain
  3 ALERT level B = A potentially serious problem, consider carefully
 13 ALERT level C = Check. Ensure it is not caused by an omission or oversight
  9 ALERT level G = General information/check it is not something unexpected

  1 ALERT type 1 CIF construction/syntax error, inconsistent or missing data
 10 ALERT type 2 Indicator that the structure model may be wrong or deficient
 12 ALERT type 3 Indicator that the structure quality may be low
  2 ALERT type 4 Improvement, methodology, query or suggestion
  0 ALERT type 5 Informative message, check
```

It is advisable to attempt to resolve as many as possible of the alerts in all categories. Often the minor alerts point to easily fixed oversights, errors and omissions in your CIF or refinement strategy, so attention to these fine details can be worthwhile. In order to resolve some of the more serious problems it may be necessary to carry out additional measurements or structure refinements. However, the purpose of your study may justify the reported deviations and the more serious of these should normally be commented upon in the discussion or experimental section of a paper or in the "special_details" fields of the CIF. checkCIF was carefully designed to identify outliers and unusual parameters, but every test has its limitations and alerts that are not important in a particular case may appear. Conversely, the absence of alerts does not guarantee there are no aspects of the results needing attention. It is up to the individual to critically assess their own results and, if necessary, seek expert advice.

Publication of your CIF in IUCr journals

A basic structural check has been run on your CIF. These basic checks will be run on all CIFs submitted for publication in IUCr journals (*Acta Crystallographica*, *Journal of Applied Crystallography*, *Journal of Synchrotron Radiation*); however, if you intend to submit to *Acta Crystallographica Section C* or *E* or *IUCrData*, you should make sure that full publication checks are run on the final version of your CIF prior to submission.

Publication of your CIF in other journals

Please refer to the *Notes for Authors* of the relevant journal for any special instructions relating to CIF submission.

PLATON version of 13/07/2021; check.def file version of 13/07/2021

Datablock 3g - ellipsoid plot

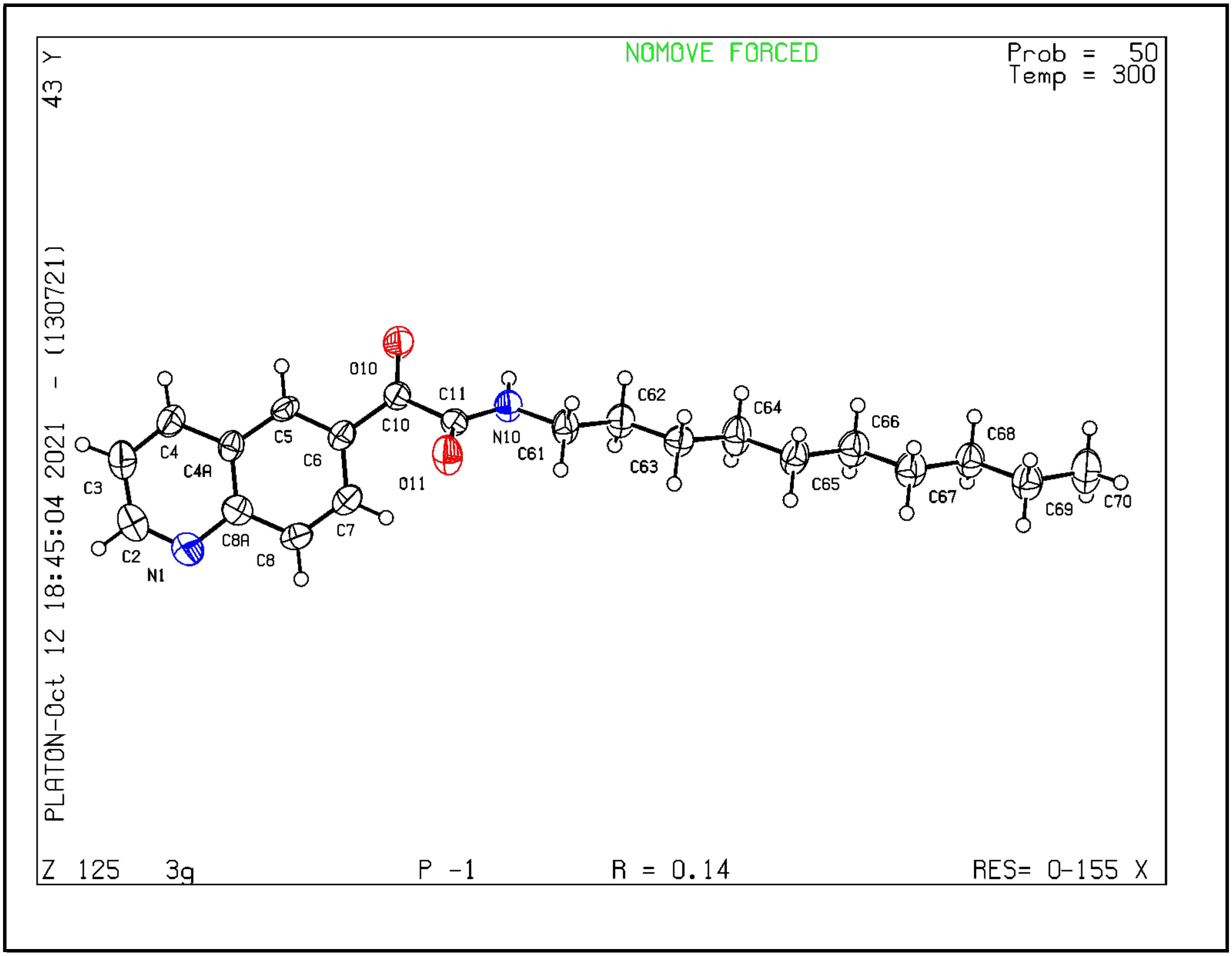

Download CIF editor (publCIF) from the IUCr
Download CIF editor (enCIFer) from the CCDC
Test a new CIF entry
