# Supplementary material for: Highly Selective Synthesis of 6-Glyoxylamidoquinoline Derivatives via Palladium-Catalyzed Aminocarbonylation
Source: Molecules. 2021 Dec 21;27(1):4. doi: 10.3390/molecules27010004 (PMC8746719; doi:10.3390/molecules27010004)
Supplement: Supplementary file 1 [file molecules-27-00004-s001.zip › 6-Iodoquinoline_Supplementary_file_Molecules.pdf]

Supporting Information

Highly Selective Synthesis of 6-Glyoxylamido-Quinoline Derivatives via Palladium-Catalyzed Aminocarbonylation

Sami Chniti [1], László Kollár [1, 2, 3], Attila Bényei [4] and Attila Takács [1, 2*]

[1]*Department of Inorganic Chemistry, University of Pécs, Ifjúság útja. 6., H-7624, Pécs, Hungary* [2]*János Szentágothai Research Centre, University of Pécs, Ifjúság útja. 20., H-7624 Pécs, Hungary*

[3]*MTA-PTE Research Group for Selective Chemical Syntheses, Ifjúság útja., 6., H-7624 Pécs, Hungary,*

[4]*University of Debrecen, Department of Pharmaceutical Chemistry, Egyetem tér 1, 4032, Hungary*

**Correspondence: takacsattila@gamma.ttk.pte.hu*

Contents

X-ray Crystallographic Data of compound **2c** 1.

X-ray Crystallographic Data of compound **2n** 5.

X-ray Crystallographic Data of compound **3g** 11.

Characterization of the synthesized compounds 16.

^{1}H and ^{13}C NMR spectra of the synthesized compounds 31.

X-ray Crystallography

Computing details

For all compounds, data collection: Bruker Instrument Service vV6.2.6; cell refinement: *APEX3* v2017.3-0 (Bruker AXS); data reduction: *SAINT* V8.38A (Bruker AXS Inc., 2017). Program(s) used to solve structure: SHELXT 2014/5 (Sheldrick, 2014) for (**2c**), (**3g**); SHELXT 2018/2 (Sheldrick, 2018) for (**2n**). Program(s) used to refine structure: *SHELXL2018*/3 (Sheldrick, 2018) for (**2c**), (**2n**); *SHELXL2016*/6 (Sheldrick, 2016) for (**3g**). For all compounds, molecular graphics: *Mercury*; software used to prepare material for publication: *WinGX*, *publCIF*.

X-ray Crystallographic Data of compound 2c

Crystal data

$C_{16}H_{18}N_2O$	$F(000) = 272$
$M_r = 254.32$	$D_x = 1.276$ Mg m^{-3}
Monoclinic, $P2_1$	Mo $K\alpha$ radiation, $\lambda = 0.71073$ Å
$a = 5.1616$ (4) Å	Cell parameters from 232 reflections
$b = 6.6616$ (5) Å	$\theta = 3.2$–$22.2°$
$c = 19.2655$ (14) Å	$\mu = 0.08$ mm^{-1}
$\beta = 92.600$ (3)°	$T = 294$ K
$V = 661.75$ (9) Å^3	Plate, colourless
$Z = 2$	0.30 × 0.17 × 0.10 mm

Data collection

Bruker D8 VENTURE diffractometer	2725 independent reflections
Radiation source: microfocus sealed tube, INCOATEC IμS 3.0	2250 reflections with $I > 2\sigma(I)$
Multilayer mirror INCOATEC monochromator	$R_{int} = 0.038$
Detector resolution: 7.3910 pixels mm^{-1}	$\theta_{max} = 26.4°$, $\theta_{min} = 3.2°$
ω and π scan	$h = -6\rightarrow6$
Absorption correction: multi-scan *SADABS2016*/2 - Bruker AXS area detector scaling and absorption correction	$k = -8\rightarrow8$
$T_{min} = 0.91$, $T_{max} = 0.99$	$l = -24\rightarrow23$
7476 measured reflections	

Refinement

Refinement on F^2	Hydrogen site location: mixed
Least-squares matrix: full	H atoms treated by a mixture of independent and constrained refinement
$R[F^2 > 2\sigma(F^2)] = 0.052$	$w = 1/[\sigma^2(F_o^2) + (0.0736P)^2]$ where $P = (F_o^2 + 2F_c^2)/3$
$wR(F^2) = 0.141$	$(\Delta/\sigma)_{max} < 0.001$
$S = 1.15$	$\Delta\rangle_{max} = 0.51$ e Å^{-3}
2725 reflections	$\Delta\rangle_{min} = -0.60$ e Å^{-3}
176 parameters	Extinction correction: *SHELXL2018*/3 (Sheldrick 2018)
2 restraints	Extinction coefficient: 0.16 (2)
Primary atom site location: structure-invariant direct methods	Absolute structure: Flack x determined using 783 quotients [(I+)-(I-)]/[(I+)+(I-)] (Parsons, Flack and Wagner, Acta Cryst. B69 (2013) 249-259).
Secondary atom site location: difference Fourier map	Absolute structure parameter: 0.4 (9)

Special details

Geometry. All esds (except the esd in the dihedral angle between two l.s. planes) are estimated using the full covariance matrix. The cell esds are taken into account individually in the estimation of esds in distances, angles and torsion angles; correlations between esds in cell parameters are only used when they are defined by crystal symmetry. An approximate (isotropic) treatment of cell esds is used for estimating esds involving l.s. planes.

*Geometric parameters (Å, º) for (**2c**)*

C2—C3	1.359 (5)	C11—N10	1.458 (4)
C2—C1A	1.411 (5)	C11—C16	1.511 (5)
C2—H2	0.93	C11—C12	1.513 (5)
C1A—N1	1.375 (4)	C11—H11	0.98
C1A—C5A	1.407 (4)	C12—C13	1.521 (5)
C3—C4	1.412 (5)	C12—H12A	0.97
C3—H3	0.93	C12—H12B	0.97
C4—C5	1.371 (5)	C13—C14	1.506 (7)
C4—C10	1.491 (5)	C13—H13A	0.97
C5—C5A	1.410 (5)	C13—H13B	0.97
C5—H5	0.93	C14—C15	1.504 (7)
C6—C7	1.356 (5)	C14—H14A	0.97

C6—C5A	1.412 (5)	C14—H14B	0.97
C6—H6	0.93	C15—C16	1.518 (6)
C7—C8	1.399 (6)	C15—H15A	0.97
C7—H7	0.93	C15—H15B	0.97
C8—N1	1.305 (5)	C16—H16A	0.97
C8—H8	0.93	C16—H16B	0.97
C10—O10	1.235 (4)	N10—H10	0.862 (14)
C10—N10	1.338 (4)		
C3—C2—C1A	120.7 (3)	C12—C11—H11	107.9
C3—C2—H2	119.6	C11—C12—C13	110.8 (3)
C1A—C2—H2	119.6	C11—C12—H12A	109.5
N1—C1A—C5A	122.7 (3)	C13—C12—H12A	109.5
N1—C1A—C2	118.5 (3)	C11—C12—H12B	109.5
C5A—C1A—C2	118.8 (3)	C13—C12—H12B	109.5
C2—C3—C4	121.1 (3)	H12A—C12—H12B	108.1
C2—C3—H3	119.4	C14—C13—C12	111.6 (3)
C4—C3—H3	119.4	C14—C13—H13A	109.3
C5—C4—C3	118.8 (3)	C12—C13—H13A	109.3
C5—C4—C10	119.1 (3)	C14—C13—H13B	109.3
C3—C4—C10	122.1 (3)	C12—C13—H13B	109.3
C4—C5—C5A	121.4 (3)	H13A—C13—H13B	108.0
C4—C5—H5	119.3	C15—C14—C13	112.0 (4)
C5A—C5—H5	119.3	C15—C14—H14A	109.2
C7—C6—C5A	119.4 (3)	C13—C14—H14A	109.2
C7—C6—H6	120.3	C15—C14—H14B	109.2
C5A—C6—H6	120.3	C13—C14—H14B	109.2
C1A—C5A—C5	119.2 (3)	H14A—C14—H14B	107.9
C1A—C5A—C6	117.4 (3)	C14—C15—C16	111.1 (4)
C5—C5A—C6	123.4 (3)	C14—C15—H15A	109.4
C6—C7—C8	118.8 (4)	C16—C15—H15A	109.4
C6—C7—H7	120.6	C14—C15—H15B	109.4
C8—C7—H7	120.6	C16—C15—H15B	109.4
N1—C8—C7	124.9 (4)	H15A—C15—H15B	108.0
N1—C8—H8	117.6	C11—C16—C15	111.6 (3)

C7—C8—H8	117.6	C11—C16—H16A	109.3
O10—C10—N10	122.4 (3)	C15—C16—H16A	109.3
O10—C10—C4	121.0 (3)	C11—C16—H16B	109.3
N10—C10—C4	116.6 (3)	C15—C16—H16B	109.3
N10—C11—C16	111.5 (3)	H16A—C16—H16B	108.0
N10—C11—C12	110.2 (3)	C8—N1—C1A	116.8 (3)
C16—C11—C12	111.2 (3)	C10—N10—C11	123.5 (3)
N10—C11—H11	107.9	C10—N10—H10	119 (3)
C16—C11—H11	107.9	C11—N10—H10	117 (3)
C3—C2—C1A—N1	-178.6 (3)	C3—C4—C10—O10	149.1 (4)
C3—C2—C1A—C5A	1.6 (5)	C5—C4—C10—N10	153.3 (3)
C1A—C2—C3—C4	-1.3 (6)	C3—C4—C10—N10	-29.8 (5)
C2—C3—C4—C5	-0.6 (5)	N10—C11—C12—C13	-179.5 (3)
C2—C3—C4—C10	-177.5 (3)	C16—C11—C12—C13	-55.4 (4)
C3—C4—C5—C5A	2.2 (5)	C11—C12—C13—C14	54.9 (5)
C10—C4—C5—C5A	179.2 (3)	C12—C13—C14—C15	-54.8 (5)
N1—C1A—C5A—C5	-179.8 (3)	C13—C14—C15—C16	54.4 (5)
C2—C1A—C5A—C5	-0.1 (5)	N10—C11—C16—C15	179.1 (4)
N1—C1A—C5A—C6	-1.2 (4)	C12—C11—C16—C15	55.7 (5)
C2—C1A—C5A—C6	178.6 (3)	C14—C15—C16—C11	-54.9 (5)
C4—C5—C5A—C1A	-1.9 (5)	C7—C8—N1—C1A	0.9 (6)
C4—C5—C5A—C6	179.5 (3)	C5A—C1A—N1—C8	0.6 (5)
C7—C6—C5A—C1A	0.2 (5)	C2—C1A—N1—C8	-179.1 (3)
C7—C6—C5A—C5	178.8 (3)	O10—C10—N10—C11	-0.3 (5)
C5A—C6—C7—C8	1.2 (6)	C4—C10—N10—C11	178.6 (3)
C6—C7—C8—N1	-1.8 (7)	C16—C11—N10—C10	91.8 (4)
C5—C4—C10—O10	-27.8 (5)	C12—C11—N10—C10	-144.2 (3)

Hydrogen-bond geometry (Å, º) for (2c)

D—H···*A*	*D*—H	H···*A*	*D*···*A*	*D*—H···*A*
N10—H10···O10^{i}	0.86 (1)	2.19 (2)	3.020 (4)	161 (4)

Symmetry code: (i) *x*+1, *y*, *z*.

X-ray Crystallographic Data of compound 2n

Crystal data

2($C_{16}H_{12}N_2O$)	Z = 4
M_r = 248.28	F(000) = 520
Triclinic, $P\bar{1}$	D_x = 1.325 Mg m^{-3}
a = 5.1930 (4) Å	Mo $K\alpha$ radiation, λ = 0.71073 Å
b = 9.0314 (7) Å	Cell parameters from 2891 reflections
c = 27.180 (2) Å	θ = 3.1–22.6°
α = 98.887 (4)°	μ = 0.09 mm^{-1}
β = 93.763 (4)°	T = 294 K
γ = 97.281 (4)°	Plate, colourless
V = 1244.55 (17) Å^3	0.38 × 0.19 × 0.04 mm

Data collection

Bruker D8 VENTURE diffractometer	4550 independent reflections
Radiation source: microfocus sealed tube, INCOATEC IμS 3.0	3249 reflections with $I > 2\sigma(I)$
Multilayer mirror INCOATEC monochromator	R_{int} = 0.140
Detector resolution: 7.3910 pixels mm^{-1}	θ_{max} = 25.4°, θ_{min} = 2.3°
ω and π scan	h = -6→6
Absorption correction: multi-scan *SADABS2016*/2 - Bruker AXS area detector scaling and absorption correction	k = -10→10
T_{min} = 0.74, T_{max} = 1.00	l = -32→32
41129 measured reflections	

Refinement

Refinement on F^2	Secondary atom site location: difference Fourier map
Least-squares matrix: full	Hydrogen site location: mixed
$R[F^2 > 2\sigma(F^2)]$ = 0.099	H atoms treated by a mixture of independent and constrained refinement
$wR(F^2)$ = 0.208	$w = 1/[\sigma^2(F_o^2) + (0.0442P)^2 + 2.1465P]$ where $P = (F_o^2 + 2F_c^2)/3$
S = 1.17	$(\Delta/\sigma)_{max}$ < 0.001

4550 reflections	$\Delta\rangle_{max}$ = 0.26 e Å^{-3}
350 parameters	$\Delta\rangle_{min}$ = -0.22 e Å^{-3}
2 restraints	Extinction correction: *SHELXL2018*/3 (Sheldrick 2018)
Primary atom site location: structure-invariant direct methods	Extinction coefficient: 0.0066 (17)

Special details

Geometry. All esds (except the esd in the dihedral angle between two l.s. planes) are estimated using the full covariance matrix. The cell esds are taken into account individually in the estimation of esds in distances, angles and torsion angles; correlations between esds in cell parameters are only used when they are defined by crystal symmetry. An approximate (isotropic) treatment of cell esds is used for estimating esds involving l.s. planes.

*Geometric parameters (Å, º) for (**2n**)*

C2—C3	1.363 (6)	C50—N60	1.349 (5)
C2—C1A	1.418 (6)	C50—C54	1.490 (6)
C2—H2	0.93	C52—C53	1.350 (6)
C1A—N1	1.363 (6)	C52—C51A	1.419 (6)
C1A—C5A	1.405 (6)	C52—H52	0.93
C3—C4	1.411 (6)	C51A—N51	1.361 (6)
C3—H3	0.93	C51A—C55A	1.417 (6)
C4—C5	1.362 (6)	C53—C54	1.430 (6)
C4—C10	1.486 (6)	C53—H53	0.93
C5—C5A	1.412 (6)	C54—C55	1.352 (6)
C5—H5	0.93	C55—C55A	1.406 (6)
C6—C7	1.352 (6)	C55—H55	0.93
C6—C5A	1.404 (6)	C56—C57	1.353 (6)
C6—H6	0.93	C56—C55A	1.409 (6)
C7—C8	1.395 (7)	C56—H56	0.93
C7—H7	0.93	C57—C58	1.397 (7)
C8—N1	1.317 (6)	C57—H57	0.93
C8—H8	0.93	C58—N51	1.313 (6)
C10—O10	1.230 (5)	C58—H58	0.93
C10—N10	1.352 (5)	C61—C62	1.370 (6)
C11—C12	1.381 (6)	C61—C66	1.387 (6)
C11—C16	1.385 (6)	C61—N60	1.429 (5)

C11—N10	1.420 (5)	C62—C63	1.385 (7)
C12—C13	1.378 (6)	C62—H62	0.93
C12—H12	0.93	C63—C64	1.368 (7)
C13—C14	1.361 (7)	C63—H63	0.93
C13—H13	0.93	C64—C65	1.370 (7)
C14—C15	1.381 (7)	C64—H64	0.93
C14—H14	0.93	C65—C66	1.381 (6)
C15—C16	1.388 (6)	C65—H65	0.93
C15—H15	0.93	C66—H66	0.93
C16—H16	0.93	N10—H10	0.861 (19)
C50—O50	1.217 (5)	N60—H60	0.86 (2)
C3—C2—C1A	120.1 (4)	C53—C52—H52	119.9
C3—C2—H2	120.0	C51A—C52—H52	119.9
C1A—C2—H2	120.0	N51—C51A—C55A	122.7 (4)
N1—C1A—C5A	122.9 (4)	N51—C51A—C52	117.9 (4)
N1—C1A—C2	117.7 (4)	C55A—C51A—C52	119.3 (4)
C5A—C1A—C2	119.4 (4)	C52—C53—C54	121.1 (4)
C2—C3—C4	121.1 (4)	C52—C53—H53	119.4
C2—C3—H3	119.5	C54—C53—H53	119.4
C4—C3—H3	119.5	C55—C54—C53	118.7 (4)
C5—C4—C3	119.0 (4)	C55—C54—C50	124.3 (4)
C5—C4—C10	123.9 (4)	C53—C54—C50	116.9 (4)
C3—C4—C10	117.0 (4)	C54—C55—C55A	122.3 (4)
C4—C5—C5A	121.9 (4)	C54—C55—H55	118.8
C4—C5—H5	119.1	C55A—C55—H55	118.8
C5A—C5—H5	119.1	C57—C56—C55A	118.9 (4)
C7—C6—C5A	119.4 (5)	C57—C56—H56	120.6
C7—C6—H6	120.3	C55A—C56—H56	120.6
C5A—C6—H6	120.3	C55—C55A—C56	123.9 (4)
C6—C5A—C1A	117.4 (4)	C55—C55A—C51A	118.3 (4)
C6—C5A—C5	124.1 (4)	C56—C55A—C51A	117.8 (4)
C1A—C5A—C5	118.5 (4)	C56—C57—C58	119.1 (5)
C6—C7—C8	119.4 (5)	C56—C57—H57	120.4
C6—C7—H7	120.3	C58—C57—H57	120.4

C8—C7—H7	120.3	N51—C58—C57	125.1 (5)
N1—C8—C7	123.9 (4)	N51—C58—H58	117.5
N1—C8—H8	118.1	C57—C58—H58	117.5
C7—C8—H8	118.1	C62—C61—C66	120.2 (4)
O10—C10—N10	123.4 (4)	C62—C61—N60	122.0 (4)
O10—C10—C4	120.2 (4)	C66—C61—N60	117.8 (4)
N10—C10—C4	116.3 (3)	C61—C62—C63	118.9 (5)
C12—C11—C16	120.0 (4)	C61—C62—H62	120.6
C12—C11—N10	122.1 (4)	C63—C62—H62	120.6
C16—C11—N10	117.8 (4)	C64—C63—C62	121.7 (5)
C13—C12—C11	118.9 (4)	C64—C63—H63	119.2
C13—C12—H12	120.6	C62—C63—H63	119.2
C11—C12—H12	120.6	C63—C64—C65	118.9 (5)
C14—C13—C12	122.1 (5)	C63—C64—H64	120.5
C14—C13—H13	118.9	C65—C64—H64	120.5
C12—C13—H13	118.9	C64—C65—C66	120.7 (5)
C13—C14—C15	119.1 (4)	C64—C65—H65	119.6
C13—C14—H14	120.5	C66—C65—H65	119.6
C15—C14—H14	120.5	C65—C66—C61	119.5 (4)
C14—C15—C16	120.2 (4)	C65—C66—H66	120.2
C14—C15—H15	119.9	C61—C66—H66	120.2
C16—C15—H15	119.9	C8—N1—C1A	117.1 (4)
C11—C16—C15	119.7 (4)	C10—N10—C11	125.3 (3)
C11—C16—H16	120.1	C10—N10—H10	120 (3)
C15—C16—H16	120.1	C11—N10—H10	114 (3)
O50—C50—N60	123.8 (4)	C58—N51—C51A	116.5 (4)
O50—C50—C54	120.9 (4)	C50—N60—C61	125.1 (3)
N60—C50—C54	115.2 (3)	C50—N60—H60	121 (3)
C53—C52—C51A	120.2 (4)	C61—N60—H60	112 (3)
C3—C2—C1A—N1	-178.7 (4)	O50—C50—C54—C53	-35.0 (6)
C3—C2—C1A—C5A	1.7 (6)	N60—C50—C54—C53	143.3 (4)
C1A—C2—C3—C4	-1.8 (7)	C53—C54—C55—C55A	0.1 (6)
C2—C3—C4—C5	0.7 (6)	C50—C54—C55—C55A	-177.7 (4)

C2—C3—C4—C10	179.1 (4)	C54—C55—C55A—C56	179.6 (4)
C3—C4—C5—C5A	0.5 (6)	C54—C55—C55A—C51A	-0.9 (6)
C10—C4—C5—C5A	-177.8 (4)	C57—C56—C55A—C55	179.1 (5)
C7—C6—C5A—C1A	-0.8 (7)	C57—C56—C55A—C51A	-0.4 (7)
C7—C6—C5A—C5	-179.9 (5)	N51—C51A—C55A—C55	-179.0 (4)
N1—C1A—C5A—C6	0.8 (7)	C52—C51A—C55A—C55	0.3 (6)
C2—C1A—C5A—C6	-179.6 (4)	N51—C51A—C55A—C56	0.5 (7)
N1—C1A—C5A—C5	179.9 (4)	C52—C51A—C55A—C56	179.8 (4)
C2—C1A—C5A—C5	-0.5 (6)	C55A—C56—C57—C58	0.0 (8)
C4—C5—C5A—C6	178.5 (4)	C56—C57—C58—N51	0.4 (9)
C4—C5—C5A—C1A	-0.6 (6)	C66—C61—C62—C63	-2.8 (7)
C5A—C6—C7—C8	0.3 (8)	N60—C61—C62—C63	179.8 (4)
C6—C7—C8—N1	0.3 (8)	C61—C62—C63—C64	1.2 (8)
C5—C4—C10—O10	143.7 (5)	C62—C63—C64—C65	0.6 (8)
C3—C4—C10—O10	-34.7 (6)	C63—C64—C65—C66	-0.7 (8)
C5—C4—C10—N10	-37.5 (6)	C64—C65—C66—C61	-0.9 (7)
C3—C4—C10—N10	144.1 (4)	C62—C61—C66—C65	2.7 (6)
C16—C11—C12—C13	-1.8 (7)	N60—C61—C66—C65	-179.8 (4)
N10—C11—C12—C13	-179.9 (4)	C7—C8—N1—C1A	-0.4 (8)
C11—C12—C13—C14	0.1 (7)	C5A—C1A—N1—C8	-0.2 (7)
C12—C13—C14—C15	1.1 (7)	C2—C1A—N1—C8	-179.8 (4)
C13—C14—C15—C16	-0.6 (7)	O10—C10—N10—C11	1.3 (7)
C12—C11—C16—C15	2.2 (6)	C4—C10—N10—C11	-177.5 (4)
N10—C11—C16—C15	-179.6 (4)	C12—C11—N10—C10	-38.8 (6)
C14—C15—C16—C11	-1.0 (7)	C16—C11—N10—C10	143.1 (4)
C53—C52—C51A—N51	-179.6 (4)	C57—C58—N51—C51A	-0.3 (8)
C53—C52—C51A—C55A	1.1 (7)	C55A—C51A—N51—C58	-0.2 (7)
C51A—C52—C53—	-1.9 (7)	C52—C51A—N51—	-179.4 (5)

C54		C58	
C52—C53—C54—C55	1.3 (7)	O50—C50—N60—C61	1.1 (7)
C52—C53—C54—C50	179.3 (4)	C54—C50—N60—C61	-177.2 (4)
O50—C50—C54—C55	142.8 (5)	C62—C61—N60—C50	-38.9 (6)
N60—C50—C54—C55	-38.9 (6)	C66—C61—N60—C50	143.7 (4)

*Hydrogen-bond geometry (Å, º) for (**2n**)*

D—H···*A*	*D*—H	H···*A*	*D*···*A*	*D*—H···*A*
C8—H8···N51^{i}	0.93	2.66	3.557 (6)	162
N10—H10···O10^{ii}	0.86 (2)	2.18 (3)	2.999 (4)	160 (5)
N60—H60···O50^{iii}	0.86 (2)	2.19 (2)	3.020 (4)	162 (5)

Symmetry codes: (i) -*x*+1, -*y*+1, -*z*+1; (ii) *x*-1, *y*, *z*; (iii) *x*+1, *y*, *z*.

X-ray Crystallographic Data of compound 3g

Crystal data

$C_{21}H_{28}N_2O_2$	$Z = 2$
$M_r = 340.45$	$F(000) = 368$
Triclinic, $P\bar{1}$	$D_x = 1.190$ Mg m^{-3}
$a = 5.2074$ (9) Å	Mo $K\alpha$ radiation, $\lambda = 0.71073$ Å
$b = 11.0289$ (18) Å	Cell parameters from 223 reflections
$c = 16.859$ (3) Å	$\theta = 2.5–21.1°$
$\alpha = 79.495$ (10)°	$\mu = 0.08$ mm^{-1}
$\beta = 86.693$ (11)°	$T = 300$ K
$\gamma = 89.903$ (9)°	Rod, colourless
$V = 950.4$ (3) Å^3	0.54 × 0.17 × 0.07 mm

Data collection

Bruker D8 VENTURE diffractometer	3507 independent reflections
Radiation source: microfocus sealed tube, INCOATEC IμS 3.0	2145 reflections with $I > 2\sigma(I)$
Multilayer mirror INCOATEC monochromator	$R_{int} = 0.193$
Detector resolution: 7.3910 pixels mm^{-1}	$\theta_{max} = 25.7°$, $\theta_{min} = 2.4°$
ω and π scan	$h = -6 \rightarrow 6$
Absorption correction: multi-scan *SADABS2016*/2 - Bruker AXS area detector scaling and absorption correction	$k = -13 \rightarrow 13$
$T_{min} = 0.42$, $T_{max} = 0.99$	$l = -20 \rightarrow 20$
20248 measured reflections	

Refinement

Refinement on F^2	Secondary atom site location: difference Fourier map
Least-squares matrix: full	Hydrogen site location: mixed
$R[F^2 > 2\sigma(F^2)] = 0.143$	H atoms treated by a mixture of independent and constrained refinement
$wR(F^2) = 0.359$	$w = 1/[\sigma^2(F_o^2) + (0.198P)^2 + 0.0785P]$ where $P = (F_o^2 + 2F_c^2)/3$
$S = 1.13$	$(\Delta/\sigma)_{max} < 0.001$
3507 reflections	$\Delta\rangle_{max} = 0.73$ e Å^{-3}

232 parameters	Δ⟩$_{min}$ = -0.59 e Å^{-3}
1 restraint	Extinction correction: *SHELXL2018*/3 (Sheldrick 2018)
Primary atom site location: structure-invariant direct methods	Extinction coefficient: 0.55 (8)

Special details

Geometry. All esds (except the esd in the dihedral angle between two l.s. planes) are estimated using the full covariance matrix. The cell esds are taken into account individually in the estimation of esds in distances, angles and torsion angles; correlations between esds in cell parameters are only used when they are defined by crystal symmetry. An approximate (isotropic) treatment of cell esds is used for estimating esds involving l.s. planes.

*Geometric parameters (Å, º) for (**3g**)*

C2—N1	1.323 (6)	C62—H62A	0.97
C2—C3	1.384 (7)	C62—H62B	0.97
C2—H2	0.93	C63—C64	1.506 (6)
C3—C4	1.348 (7)	C63—H63A	0.97
C3—H3	0.93	C63—H63B	0.97
C4—C4A	1.415 (6)	C64—C65	1.509 (6)
C4—H4	0.93	C64—H64A	0.97
C5—C6	1.363 (6)	C64—H64B	0.97
C5—C4A	1.421 (6)	C65—C66	1.513 (6)
C5—H5	0.93	C65—H65A	0.97
C4A—C8A	1.400 (6)	C65—H65B	0.97
C6—C7	1.405 (6)	C66—C67	1.498 (7)
C6—C10	1.479 (6)	C66—H66A	0.97
C7—C8	1.356 (7)	C66—H66B	0.97
C7—H7	0.93	C67—C68	1.511 (6)
C8—C8A	1.409 (6)	C67—H67A	0.97
C8—H8	0.93	C67—H67B	0.97
C8A—N1	1.369 (6)	C68—C69	1.503 (7)
C10—O10	1.216 (5)	C68—H68A	0.97
C10—C11	1.536 (6)	C68—H68B	0.97
C11—O11	1.216 (5)	C69—C70	1.507 (7)
C11—N10	1.317 (5)	C69—H69A	0.97
C61—N10	1.465 (5)	C69—H69B	0.97

C61—C62	1.488 (6)	C70—H70A	0.96
C61—H61A	0.97	C70—H70B	0.96
C61—H61B	0.97	C70—H70C	0.96
C62—C63	1.511 (6)	N10—H10	0.85 (2)
N1—C2—C3	125.1 (4)	C62—C63—H63B	108.6
N1—C2—H2	117.5	H63A—C63—H63B	107.5
C3—C2—H2	117.5	C63—C64—C65	114.9 (4)
C4—C3—C2	119.3 (5)	C63—C64—H64A	108.5
C4—C3—H3	120.4	C65—C64—H64A	108.5
C2—C3—H3	120.4	C63—C64—H64B	108.5
C3—C4—C4A	118.9 (4)	C65—C64—H64B	108.5
C3—C4—H4	120.6	H64A—C64—H64B	107.5
C4A—C4—H4	120.6	C64—C65—C66	115.6 (4)
C6—C5—C4A	120.5 (4)	C64—C65—H65A	108.4
C6—C5—H5	119.7	C66—C65—H65A	108.4
C4A—C5—H5	119.7	C64—C65—H65B	108.4
C8A—C4A—C4	117.9 (4)	C66—C65—H65B	108.4
C8A—C4A—C5	119.0 (4)	H65A—C65—H65B	107.4
C4—C4A—C5	123.1 (4)	C67—C66—C65	114.9 (4)
C5—C6—C7	119.6 (4)	C67—C66—H66A	108.5
C5—C6—C10	119.5 (4)	C65—C66—H66A	108.5
C7—C6—C10	120.8 (4)	C67—C66—H66B	108.5
C8—C7—C6	121.1 (4)	C65—C66—H66B	108.5
C8—C7—H7	119.4	H66A—C66—H66B	107.5
C6—C7—H7	119.4	C66—C67—C68	115.8 (4)
C7—C8—C8A	120.2 (4)	C66—C67—H67A	108.3
C7—C8—H8	119.9	C68—C67—H67A	108.3
C8A—C8—H8	119.9	C66—C67—H67B	108.3
N1—C8A—C4A	122.7 (4)	C68—C67—H67B	108.3
N1—C8A—C8	117.9 (4)	H67A—C67—H67B	107.4
C4A—C8A—C8	119.4 (4)	C69—C68—C67	115.2 (4)
O10—C10—C6	123.5 (4)	C69—C68—H68A	108.5
O10—C10—C11	118.7 (4)	C67—C68—H68A	108.5
C6—C10—C11	117.6 (3)	C69—C68—H68B	108.5

O11—C11—N10	124.9 (4)	C67—C68—H68B	108.5
O11—C11—C10	118.8 (3)	H68A—C68—H68B	107.5
N10—C11—C10	116.4 (3)	C68—C69—C70	114.7 (4)
N10—C61—C62	113.7 (4)	C68—C69—H69A	108.6
N10—C61—H61A	108.8	C70—C69—H69A	108.6
C62—C61—H61A	108.8	C68—C69—H69B	108.6
N10—C61—H61B	108.8	C70—C69—H69B	108.6
C62—C61—H61B	108.8	H69A—C69—H69B	107.6
H61A—C61—H61B	107.7	C69—C70—H70A	109.5
C61—C62—C63	112.5 (4)	C69—C70—H70B	109.5
C61—C62—H62A	109.1	H70A—C70—H70B	109.5
C63—C62—H62A	109.1	C69—C70—H70C	109.5
C61—C62—H62B	109.1	H70A—C70—H70C	109.5
C63—C62—H62B	109.1	H70B—C70—H70C	109.5
H62A—C62—H62B	107.8	C2—N1—C8A	116.1 (4)
C64—C63—C62	114.8 (4)	C11—N10—C61	120.2 (4)
C64—C63—H63A	108.6	C11—N10—H10	119 (4)
C62—C63—H63A	108.6	C61—N10—H10	120 (4)
C64—C63—H63B	108.6		
N1—C2—C3—C4	-0.4 (8)	C7—C6—C10—C11	-28.4 (6)
C2—C3—C4—C4A	-0.7 (8)	O10—C10—C11—O11	138.8 (5)
C3—C4—C4A—C8A	0.8 (7)	C6—C10—C11—O11	-35.9 (6)
C3—C4—C4A—C5	-178.3 (4)	O10—C10—C11—N10	-40.2 (6)
C6—C5—C4A—C8A	-2.1 (7)	C6—C10—C11—N10	145.0 (4)
C6—C5—C4A—C4	177.0 (4)	N10—C61—C62—C63	178.5 (4)
C4A—C5—C6—C7	3.3 (7)	C61—C62—C63—C64	-179.8 (4)
C4A—C5—C6—C10	-177.2 (4)	C62—C63—C64—C65	-179.3 (4)
C5—C6—C7—C8	-1.7 (8)	C63—C64—C65—C66	-176.9 (4)
C10—C6—C7—C8	178.7 (5)	C64—C65—C66—C67	179.8 (4)
C6—C7—C8—C8A	-1.0 (9)	C65—C66—C67—C68	-178.4 (4)
C4—C4A—C8A—N1	0.1 (7)	C66—C67—C68—C69	-179.9 (4)
C5—C4A—C8A—N1	179.3 (4)	C67—C68—C69—C70	-179.2 (5)
C4—C4A—C8A—C8	-179.8 (5)	C3—C2—N1—C8A	1.3 (8)
C5—C4A—C8A—C8	-0.6 (7)	C4A—C8A—N1—C2	-1.1 (7)

C7—C8—C8A—N1	-177.7 (5)	C8—C8A—N1—C2	178.8 (5)
C7—C8—C8A—C4A	2.1 (8)	O11—C11—N10—C61	-1.4 (7)
C5—C6—C10—O10	-22.4 (7)	C10—C11—N10—C61	177.6 (4)
C7—C6—C10—O10	157.2 (5)	C62—C61—N10—C11	163.3 (4)
C5—C6—C10—C11	152.1 (4)		

*Hydrogen-bond geometry (Å, º) for (**3g**)*

D—H···*A*	*D*—H	H···*A*	*D*···*A*	*D*—H···*A*
N10—H10···O11^{i}	0.85 (2)	2.24 (3)	3.046 (5)	159 (5)

Symmetry code: (i) *x*-1, *y*, *z*.

Document origin: *publCIF* [Westrip, S. P. (2010). *J. Apply. Cryst.*, **43**, 920-925].

Characterization of the synthesized compounds

N-(tert-Butyl)-quinoline-6-carboxamide **(2a)**

Yield: 44 mg (8%); brown solid, m.p: 139-141 °C, R_f (80 % $CHCl_3$, 20 % EtOAc) 0.25; IR (KBr, ν (cm^{-1})): 3336 *m*, 2978 *w*, 2963 *w*, 1649 *vs*, 1637 *vs*, 1621 *m*, 1543 *m*, 800 *m*. ^{1}H NMR (500 *MHz*, $CDCl_3$) δ 8.94 (H_{Ar}, d, *J* = 3.7 *Hz*, 1H), 8.20 (H_{Ar}, *s*, 1H), 8.16 (H_{Ar}, *d*, *J* = 8.2 *Hz*, 1H), 8.08 (H_{Ar}, *d*, *J* = 8.7 *Hz*, 1H), 7.98 (H_{Ar}, *d*, *J* = 8.7 *Hz*, 1H), 7.42 (H_{Ar}, *dd*, *J* = 8.2, 4.2 *Hz*, 1H), 6.26 (NH, *br s*, 1H,), 1.51 ($C(\underline{CH_3})_3$, *s*, 9H). ^{13}C NMR (126 *MHz*, $CDCl_3$) δ 166.3 (CO), 151.7, 149.1, 136.9, 133.8, 129.8, 127.51, 127.2, 127.1, 121.8, 51.9 ($\underline{C}(CH_3)_3$), 28.9 ($C(\underline{C}H_3)_3$). MS (EI): *m/z* (rel. int, %): 228 (25, [M^+]), 156 (100), 128 (35), 101 (8), 77 (6), 51 (4).

N-(tert-Butyl)-2-oxo-2-(quinolin-6-yl)acetamide **(3a)**

Yield: 159 mg (62%); pale-yellow solid, m.p: 119-122 °C, R_f (85 % $CHCl_3$, 15 % EtOAc) 0.55. IR (KBr, ν (cm-1)): 3201 *w*, 3042 *w*, 2993 *w* 2969 *m*, 2928 *w*, 2869 *w*, 1688 *vs*, 1664 *vs*, 1622 *s*, 1574 *m*, 1565 *m*, 1459 *m*, 1435 *m*, 1364 *m*, 1241 *w*, 1226 *s*, 1171 *s*, 845 *m*. ^{1}H NMR (500 *MHz*, $CDCl_3$) δ 9.19 (H_{Ar}, *s*, 1H), 9.00 (H_{Ar}, dd, *J* = 4, 1.5 *Hz*, 1H), 8.40 (H_{Ar} , *dd*, *J* = 8.9, 1.2 *Hz*, 1H), 8.30 (H_{Ar}, *d*, *J* = 8.2 *Hz*, 1H), 8.14 (H_{Ar}, *d*, *J* = 8.9 *Hz*, 1H), 7.47 (H_{Ar}, *dd*, *J* = 8.2, 4.2 *Hz*, 1H), 7.11 (NH, *br s*, 1H), 1.49 ($C(\underline{CH_3})_3$, *s*, 9H). ^{13}C NMR (126 *MHz*, $CDCl_3$) δ 187.4 (CO), 160.9 (CO), 153.2, 150.5, 138.1, 134.7, 131.1, 129.9, 129.3, 127.3, 122.0, 51.8, 28.4. MS (EI): *m/z* (rel. int, %): 256 (1, [M^+]), 200 (12), 156 (100), 128 (38), 101 (10), 77 (5), 57 (35).

Piperidin-1-yl-(quinolin-6-yl)methanone **(2b)**

Yield: 39 mg (16%); light oil, R_f (70 % $CHCl_3$, 30 % EtOAc) 0.25; IR (KBr, ν (*cm^{-1}*)): 3456 *m/br*, 2938 *w*, 2855 *w*, 1630 *s*, 1620 *s*, 1475 *m*, 1442 *m*, 1275 *m*, 854 *m*. ^{1}H NMR (500 *MHz*, $CDCl_3$) δ 8.91(H_{Ar}, *dd*, *J* = 3.5, 1 *Hz*, 1H), 8.15 (H_{Ar}, *d*, *J* = 8.3 *Hz*, 1H), 8.10 (H_{Ar}, *d*, *J* = 8.6 *Hz*, 1H), 7.85 (H_{Ar}, s, 1H), 7.68 (H_{Ar}, *d*, *J* = 8.6 *Hz*, 1H), 7.40 (H_{Ar}, *dd*, *J* = 8.2, 4.2 *Hz*, 1H), 3.73 ($(CH_2)_{piperidinyl}$, *br s*, 2H), 3.34 ($(CH_2)_{piperidinyl}$, *br s*, 2H), 1.66 ($((CH_2)_2)_{piperidinyl}$, *br s*, 4H), 1.50 ($(CH_2)_{piperidinyl}$, *br s*, 2H). ^{13}C NMR (126 *MHz*, $CDCl_3$) δ 169.5 (CO), 151.4, 148.2, 136.5, 134.7, 129.7, 127.8, 127.8, 126.5, 121.8, 48.8, 43.2$(CH_2)_{piperidinyl}$, 26.5 $(CH_2)_{piperidinyl}$, 25.6 $(CH_2)_{piperidinyl}$, 24.5 $((\underline{C}H_2)_{\underline{2}})_{piperidinyl}$. MS (EI): *m/z* (rel. int, %): 240 (52, [M^+]), 239 (100), 207 (2), 156 (7), 128 (56), 101 (20), 44 (4).

1-(Piperidin-1-yl)-2-(quinolin-6-yl)ethane-1,2-dione **(3b)**

Yield: 220 mg (82%); orange waxy solid, R_f (70 % $CHCl_3$, 30 %EtOAc) 0.45; IR (KBr, ν (cm-1)): 2944 *w*, 2866 *w*, 2847 *w*, 1673 *vs*, 1641 *vs*, 1621 *s*, 1446 *w*, 1321 *m*, 1294 *m*, 1246 *m*, 1171 *m*, 1121 *m*, 976 *w*, 841 *m*. ^{1}H NMR (500 *MHz*, $CDCl_3$) δ 9.06 (H_{Ar}, *dd*, *J* = 4.3, 1.7 *Hz*, 1H), 8.48 (H_{Ar}, *d*, *J* = 1.5 *Hz*, 1H), 8.34 (H_{Ar}, *d*, *J* = 8.3 *Hz*, 1H), 8.29 (H_{Ar}, *dd*, *J* = 8.8, 1.8 *Hz*, 1H), 8.25 (H_{Ar}, *d*, *J* = 8.8 *Hz*, 1H), 7.55 (H_{Ar}, *dd*, *J* = 8.3, 4.3 *Hz*, 1H), 3.77 ($(CH_2)_{piperidinyl}$,

m, 2H), 3.37 – 3.33 ((CH$_2$)$_{piperidinyl}$, *t*, *J* = 5.5 *Hz*, 2H), 1.76 – 1.72 (((CH$_2$)$_2$)$_{piperidinyl}$, *m*, 4H), 1.59-1.74 ((CH$_2$)$_{piperidinyl}$, *m*, 2H). ^{13}C NMR (126 *MHz*, CDCl$_3$) δ 191.1 (CO), 165.1 (CO), 153.0, 150.3, 138.2, 132.4, 131.3, 130.4, 127.8, 127.6, 122.3, 47.2 (CH$_2$)$_{piperidinyl}$, 42.4 (CH$_2$)$_{piperidinyl}$, 26.3 (CH$_2$)$_{piperidinyl}$, 25.5 (CH$_2$)$_{piperidinyl}$, 24.4 (CH$_2$)$_{piperidinyl}$. MS (EI): *m/z* (rel. int, %): 268 (7, [M$^+$]), 240 (47), 207 (5), 156 (85), 128 (47), 112 (100), 101 (21), 69 (72) 41 (35).

N-Cyclohexylquinoline-6-carboxamide **(2c)**

Yield: 33 mg (13%); colorless crytals, m.p: 213-215 °C, R$_f$ (95 % CHCl$_3$, 5 %EtOAc) 0.25; IR (KBr, ν (cm^{-1})): 3195 *w*, 3037 *w*, 2923 *m*, 2853 *w*, 1684 *s*, 1657 v*s*, 1622 *m*, 1559 *w*, 1173 *m*, 803 *m*. ^{1}H NMR (500 *MHz*, CDCl$_3$) δ 8.96 (H$_{Ar}$, *dd*, *J* = 4.2, 1.5 *Hz*, 1H), 8.29 (H$_{Ar}$, *d*, *J* = 1.7 *Hz*, 1H), 8.22 (H$_{Ar}$, *d*, *J* = 7.6 *Hz*, 1H), 8.14 (H$_{Ar}$, d, *J* = 8.8 *Hz*, 1H), 8.05 (H$_{Ar}$, *dd*, *J* = 8.8, 1.9 *Hz*, 1H), 7.46 (H$_{Ar}$, *dd*, *J* = 8.3, 4.2 *Hz*, 1H), 6.36 (NH, *d*, *J* = 7.4 *Hz*, 1H), 4.04 (C$\underline{H}_{Cy}$, *tdt*, *J* = 11.7, 8.0, 3.9 *Hz*, 1H), 2.08 ((C$\underline{H_2}$)$_{Cy}$, *dd*, *J* = 12.2, 3.1 *Hz*, 2H), 1.81 – 1.76 ((C$\underline{H_2}$)$_{Cy}$, *dt*, J = 14.0, 3.5 *Hz*, 2H), 1.70 – 1.65 ((-H$_{\underline{b}}$C$\underline{H_a}$-)$_{Cy}$, *dt*, *J* = 13.0, 3.5 *Hz*, 1H), 1.50 – 1.39 ((CH$_2$)$_{Cy}$, m, 2H), 1.35 – 1.16 ((-H$_{\underline{b}}$C$\underline{H_a}$-C$\underline{H_2}$)$_{Cy}$, m, 3H). ^{13}C NMR (126 *MHz*, CDCl$_3$) δ 165.9 (CO), 151.6, 148.9, 137.3, 133.1, 129.6, 127.6, 127.5, 127.4, 121.9, 49.1, 33.2 (2×($\underline{C}$H$_2$)$_{Cy}$), 25.6, 25.0 (2×($\underline{C}$H$_2$)$_{Cy}$). MS (EI): *m/z* (rel. int, %) : 254 (40, [M$^+$]), 173 (65), 156 (100), 128 (58), 101 (20), 40 (11).

N-Cyclohexyl-2-oxo-2-(quinolin-6-yl)acetamide **(3c)**

Yield: 184 mg (65%); pale-yellow waxy solid, R$_f$ (95 % CHCl$_3$, 5 % EtOAc) 0.40; IR (KBr, ν (cm^{-1})): 3195 *w*, 3037 *w*, 2923 *m*, 2853 *w*, 1684 *s*, 1657 vs, 1622 *m*, 1559 *w*, 1173 *m*, 803 *m*. ^{1}H NMR (500 *MHz*, CDCl$_3$) δ 9.24 (H$_{Ar}$, *d*, *J* = 1.7 *Hz*, 1H), 9.03 (H$_{Ar}$, *dd*, *J* = 4.2, 1.6 *Hz*, 1H), 8.43 (H$_{Ar}$, *dd*, *J* = 8.9, 1.9 *Hz*, 1H), 8.33 (H$_{Ar}$, *d*, *J* = 7.7 *Hz*, 1H), 8.17 (H$_{Ar}$, *d*, *J* = 8.9 *Hz*, 1H), 7.51 (H$_{Ar}$, *dd*, *J* = 8.3, 4.3 *Hz*, 1H), 7.15 (NH, *d*, *J* = 7.3 *Hz*, 1H), 3.94 – 3.86 (C$\underline{H}_{Cy}$, *m*, 1H), 2.03 ((C$\underline{H_2}$)$_{Cy}$, *dd*, *J* = 12.5, 3.4 *Hz*, 2H), 1.85 – 1.75 ((C$\underline{H_2}$)$_{Cy}$, *m*, 2H), 1.71 – 1.64 ((-H$_a$C$\underline{H_b}$-)$_{Cy}$ *m*, 1H), 1.50 – 1.38 ((C$\underline{H_2}$)$_{Cy}$ *m*, 2H), 1.38 – 1.18 ((-H$_b$C$\underline{H_a}$-C$\underline{H_2}$)$_{Cy}$ *m*, 3H). ^{13}C NMR (126 *MHz*, CDCl$_3$) δ 187.0 (CO), 160.7 (CO), 153.0, 150.3, 138.4, 134.8, 131.2, 129.7, 129.3, 127.4, 122.0, 48.7, 32.7 (2×($\underline{C}$H$_2$)$_{Cy}$), 25.4, 24.8 (2×($\underline{C}$H$_2$)$_{Cy}$). MS (EI): *m/z* (rel. int, %): 282 (13, [M$^+$]), 156 (100), 129 (68), 101 (14), 83 (30), 55 (26).

Pyrrolidin-1-yl(quinolin-6-yl)methanone **(2d)**

Yield: 57 mg (25%); brownish oil, R$_f$ (70 % CHCl$_3$, 30 % EtOAc , 5 % MeOH) 0.60; IR (KBr, ν (cm^{-1})): 3449 *w*/*br*, 2973 *w*, 2877 *m*, 1628 *vs*, 1612 *vs*, 1588 *m*, 1468 *m*, 1435 *m*, 847 *m*. ^{1}H NMR (500 *MHz*, CDCl$_3$) δ 8.94 (H$_{Ar}$, *dd*, *J* = 4.2, 1.6 *Hz*, 1H), 8.18 (H$_{Ar}$, *d*, *J* = 7.9 *Hz*, 1H), 8.12 (H$_{Ar}$, *d*, *J* = 8.7 *Hz*, 1H), 7.99 (H$_{Ar}$, *d*, *J* = 1.5 *Hz*, 1H), 7.83 (H$_{Ar}$, *dd*, *J* = 8.7, 1.8 *Hz*, 1H), 7.43 (H$_{Ar}$, *dd*, *J* = 8.3, 4.2 *Hz*, 1H), 3.69 ((C$\underline{H_2}$)$_{Pyr}$, *t*, *J* = 7.0 *Hz*, 2H), 3.46 ((C$\underline{H_2}$)$_{Pyr}$, *t*, *J* = 6.6 *Hz*, 2H), 2.03 – 1.94 ((C$\underline{H_2}$)$_{Pyr}$, *qt*, 2H), 1.93 – 1.83 ((C$\underline{H_2}$)$_{Pyr}$, *qt*, 2H). ^{13}C NMR (126 *MHz*, CDCl$_3$) δ 169.0

(CO), 151.5, 148.4, 136.7, 135.3, 129.6, 128.0, 127.6, 127.0, 121.8, 49.7 $(\underline{C}H_2)_{Pyr}$, 46.4 $(\underline{C}H_2)_{Pyr}$, 26.4 $(\underline{C}H_2)_{Pyr}$, 24.5 $(\underline{C}H_2)_{Pyr}$. MS (EI): *m/z* (rel. int, %): 226 (58, [M$^+$]), 197 (19), 156 (100), 128 (56), 101 (25), 77 (11), 51 (6).

1-(Pyrrolidin-1-yl)-2-(quinolin-6-yl)ethane-1,2-dione **(3d)**

Yield: 143 mg (56%); brownish waxy solid, R$_f$ (70 % $CHCl_3$, 30 % EtOAc , 5 % MeOH) 0. 40; IR (KBr, ν (cm^{-1})): 2964 *w,* 1635 *s,* 1623 *s,* 1457 *w,* 1262 *s,* 1098 *m,* 1019 *m,* 803 *m.* ^{1}H NMR (500 *MHz*, $CDCl_3$) δ 9.08 (H$_{Ar}$, *dd, J* = 4.2, 1.3 *Hz*, 1H), 8.61 (H$_{Ar}$, *s,* 1H), 8.44 – 8.33 (H$_{Ar}$, *t, J* = 10 *Hz*, 2H), 8.28 (H$_{Ar}$, *d, J* = 8.9 *Hz*, 1H), 7.57 (H$_{Ar}$, *dd, J* = 8.3, 4.3 *Hz*, 1H), 3.74 ($(C\underline{H}_2)_{Pyr}$, *t, J* = 6.7 *Hz*, 1H), 3.53 ($(C\underline{H}_2)_{Pyr}$, *t, J* = 6.4 *Hz*, 1H), 2.07 – 1.94 ($((C\underline{H}_2)_2)_{Pyr}$, *m,* 4H). ^{13}C NMR (126 *MHz*, $CDCl_3$) δ 190.3 (CO), 164.2 (CO), 152.2, 149.4, 139.1, 132.9, 131.3, 129.7, 128.6, 127.7, 122.2, 46.9 $(\underline{C}H_2)_{Pyr}$, 45.6 $(\underline{C}H_2)_{Pyr}$, 26.0 $(\underline{C}H_2)_{Pyr}$, 24.0 $(\underline{C}H_2)_{Pyr}$. MS (EI): *m/z* (rel. int, %): 254 (5, [M$^+$]), 226 (44), 156 (100), 128 (50), 98 (100), 77 (11), 55 (75).

(4-Methylpiperazin-1-yl)(quinolin-6-yl)methanone **(2e)**

Yield: 202 mg (79%); light orange oil, R$_f$ (40 % $CHCl_3$, 40 % EtOAc , 20 % MeOH) 0.30; IR (KBr, ν (cm^{-1})): 3447 *m/br*, 2956 *w,* 2862 *m,* 2853 *w,* 1635 *m,* 1629 *vs,* 1620 *vs,* 1472 *m,* 1261 *m,* 803 *m.* ^{1}H NMR (500 *MHz*, $CDCl_3$) δ 9.00 (H$_{Ar}$, *dd, J* = 4.2, 1.6 *Hz*, 1H), 8.22 (H$_{Ar}$, *d, J* = 7.9 *Hz*, 1H), 8.17 (H$_{Ar}$, *d, J* = 8.6 *Hz*, 1H), 7.94 (H$_{Ar}$, *d, J* = 1.6 *Hz*, 1H), 7.75 (H$_{Ar}$, *dd, J* = 8.6, 1.8 *Hz*, 1H), 7.49 (H$_{Ar}$, *dd, J* = 8.3, 4.2 *Hz*, 1H), 3.97 ($(C\underline{H}_2)_{Pyz}$, *br s,* 2H), 3.63 ($(C\underline{H}_2)_{Pyz}$, *br s,* 2H), 2.67 ($(C\underline{H}_2)_{Pyz}$, *br s,* 2H), 2.55 ($(C\underline{H}_2)_{Pyz}$, *br s,* 2H), 2.46 (N-CH_3, *s,* 3H). ^{13}C NMR (126 *MHz*, $CDCl_3$) δ 169.7 (CO), 151.7, 148.5, 136.48, 133.5, 130.1, 127.8, 127.8, 127.1, 122.00, 54.7 (*br,* $(\underline{C}H_2)_{Pyz}$), 54.7 (*br,* $(\underline{C}H_2)_{Pyz}$), 47.1 (*br,* $C\underline{H_2})_{Pyz}$), 45.5 (N-$\underline{C}H_3$), 41.6 (*br,* $(\underline{C}H_2)_{Pyz}$). MS (EI): *m/z* (rel. int, %): 255 (5, [M$^+$]), 198 (32), 156 (15), 128 (19), 99 (20), 83 (39), 70 (100), 56 (26).

1-(4-Methylpiperazin-1-yl)-2-(quinolin-6-yl)ethane-1,2-dione **(3e)**

Yield: 187 mg (66%); pale yellow solid, m.p: 140-142 °C, R$_f$ (33 % $CHCl_3$, 33 % EtOAc , 33 % MeOH) 0.50; IR (KBr, ν (cm^{-1})): 2059 *w,* 2801 *w,* 1670 *s,* 1636 *vs,* 1621*m,* 1447 *m,* 1219 *m,* 1176 *m,* 1120 *m,* 830 *w,* 785 *w.* ^{1}H NMR (500 *MHz*, $CDCl_3$) δ 9.06 (H$_{Ar}$, *dd, J* = 4.2, 1.6 *Hz*, 1H), 8.47 (H$_{Ar}$, *d, J* = 1.6 *Hz*, 1H), 8.31 (H$_{Ar}$, *d, J* = 7.5 *Hz*, 1H), 8.27 (H$_{Ar}$, *dd, J* = 8.8, 1.9 *Hz*, 1H), 8.21 (H$_{Ar}$, *d, J* = 8.8 *Hz*, 1H), 7.53 (H$_{Ar}$, *dd, J* = 8.3, 4.2 *Hz*, 1H). 3.89 ($(C\underline{H}_2)_{Pyz}$, *br s,* 2H), 3.47 ($(C\underline{H}_2)_{Pyz}$, *t, J* = 5.0 *Hz*, 2H), 2.60 ($(C\underline{H}_2)_{Pyz}$, *br s,* 2H), 2.46 ($(C\underline{H}_2)_{Pyz}$, *br s,* 2H), 2.37 ((N-$\underline{C}H_3$), *s,* 3H). ^{13}C NMR (126 *MHz*, $CDCl_3$) δ 190.7 (CO), 165.1 (CO), 153.5, 150.8, 137.8, 132.7, 130.9, 130.9, 127.5, 127.5, 122.3, 54.98 ($(\underline{C}H_2)_{Pyz}$), 54.4 ($(\underline{C}H_2)_{Pyz}$), 45.9 (N-$\underline{C}H_3$), 45.8 ($(\underline{C}H_2)_{Pyz}$), 41.2($(\underline{C}H_2)_{Pyz}$). MS (EI): *m/z* (rel. int, %): 283 (49, [M$^+$]), 156 (43), 128 (28), 99 (100), 70 (57), 56 (35), 42 (17).

N-Cyclopentylquinoline-6-carboxamide **(2f)**

Yield: 175 mg (73%); yellow solid, m.p: 180-182 °C, R_f (70 % $CHCl_3$, 30 % EtOAc , 2 % MeOH) 0.30; IR (KBr, ν (cm^{-1})): 3251 *m*, 3075 *w*, 2959 *w*, 2868 *w*, 1641 *w*, 1629 *s*, 1616 *m*, 1554 *m*, 802 *m*. ^{1}H NMR (500 *MHz*, $CDCl_3$) δ 8.92 (H_{Ar}, *dd*, *J* = 4.1, 1.4 *Hz*, 1H), 8.25 (H_{Ar}, *d*, *J* = 1.4 *Hz*, 1H), 8.14 (H_{Ar}, *d*, *J* = 8.1 *Hz*, 1H), 8.08 (H_{Ar}, *d*, *J* = 8.8 *Hz*, 1H), 8.02 (H_{Ar}, *dd*, *J* = 8.8, 1.8 *Hz*, 1H), 7.41 (H_{Ar}, *dd*, *J* = 8.3, 4.2 *Hz*, 1H), 6.64 (NH, *d*, *J* = 6.6 *Hz*, 1H), 4.48 – 4.39 ($(\text{-C}\underline{\text{H}}\text{-})_{Cp}$, *m*, 1H), 2.16 – 2.03 ($(\text{C}\underline{\text{H}}_2)_{Cp}$, *m*, 2H), 1.80 – 1.69 ($(\text{C}\underline{\text{H}}_2)_{Cp}$, *m*, 2H), 1.69 – 1.60 ($(\text{C}\underline{\text{H}}_2)_{Cp}$, *m*, 2H), 1.60 – 1.49 ($(\text{C}\underline{\text{H}}_2)_{Cp}$, *m*, 2H). ^{13}C NMR (126 *MHz*, $CDCl_3$) δ 166.5 (CO), 151.6, 148.9, 137.1, 132.9, 129.6, 127.5, 127.5, 127.4, 121.8, 52.0 $(\text{-}\underline{\text{C}}\text{H-})_{Cp}$, 33.2 (2×$(\underline{\text{C}}\text{H}_2)_{Cp}$), 23.9 (2×$(\underline{\text{C}}\text{H}_2)_{Cp}$). MS (EI): *m/z* (rel. int, %): 240 (44, [M^+]), 173 (45), 156 (100), 128 (61), 101 (22), 77 (10), 40 (10).

N-Cyclopentyl-2-oxo-2-(quinolin-6-yl)acetamide **(3f)**

Yield: 145 mg (54%); light oil, R_f (70 % $CHCl_3$, 30 % EtOAc , 5 % MeOH) 0.40; IR (KBr, ν (cm^{-1})): 3292 *m*, 3063 *w*, 2956 *w*, 2870 *w*, 1684 *w*, 1675 *vs*, 1649 *vs*, 1621 *w*, 1539 *w*, 1169 *m*, 810 *w*. ^{1}H NMR (500 *MHz*, $CDCl_3$) δ 9.32 (H_{Ar}, *d*, *J* = 1.5 *Hz*, 1H), 9.07 (H_{Ar}, *dd*, *J* = 4.3, 1.6 *Hz*, 1H), 8.49 (H_{Ar}, *dd*, *J* = 8.9, 1.8 *Hz*, 1H), 8.42 (H_{Ar}, *d*, *J* = 8.2 *Hz*, 1H), 8.27 (H_{Ar}, *d*, *J* = 8.9 *Hz*, 1H), 7.57 (H_{Ar}, *dd*, *J* = 8.2, 4.3 *Hz*, 1H), 7.18 (NH, *d*, *J* = 4.8 *Hz*, 1H), 4.40 – 4.31 ($(\text{-C}\underline{\text{H}}\text{-})_{Cp}$, *m*, 1H), 2.13 ($(\text{C}\underline{\text{H}}_2)_{Cp}$, *td*, *J* = 12.8, 7.1 *Hz*, 2H), 1.80 ($(\text{C}\underline{\text{H}}_2)_{Cp}$, *dt*, *J* = 17.3, 9.0 *Hz*, 2H), 1.76 – 1.66 ($(\text{C}\underline{\text{H}}_2)_{Cp}$, *m*, 2H), 1.59 (*dt*, *J* = 20.3, 7.1 *Hz*, 2H). ^{13}C NMR (126 *MHz*, $CDCl_3$) δ 186.2 (CO), 160.7 (CO), 150.1, 141.6, 134.7, 132.3, 131.3, 127.7, 127.4, 127.3, 121.8, 51.5 $(\text{-}\underline{\text{C}}\text{H-})_{Cp}$, 33.0 (2×$(\underline{\text{C}}\text{H}_2)_{Cp}$), 23.8 (2×$(\underline{\text{C}}\text{H}_2)_{Cp}$). MS (EI): *m/z* (rel. int, %): 268 (19, [M^+]), 156 (100), 129 (52), 101 (15), 77 (7), 69 (30), 41 (14).

N-Decylquinoline-6-carboxamide **(2g)**

Yield: 178 mg (57%); pale yellow crystals, m.p: 76-78 °C, R_f (90 % $CHCl_3$, 10 % EtOAc) 0.30; IR (KBr, ν (cm^{-1})): 3320 *s*, 2922 *s*, 2850 *m*, 1637 *vs*, 1617 *m*, 1534 *vs*, 1494 *s*, 845 *s*. ^{1}H NMR (500 *MHz*, $CDCl_3$) δ 8.99 – 8.94 (H_{Ar}, *dd*, *J* = 3.5, 1.0 *Hz*, 1H), 8.30 (H_{Ar}, *s*, 1H), 8.20 (H_{Ar}, *d*, *J* = 8.2 *Hz*, 1H), 8.13 (H_{Ar}, *d*, *J* = 8.8 *Hz*, 1H), 8.05 (H_{Ar}, *dd*, *J* = 8.8, 1.6 *Hz*, 1H), 7.45 (H_{Ar}, *dd*, *J* = 8.3, 4.2 *Hz*, 1H), 6.58 (NH, *br s*, 1H), 3.51 ($(\text{C}\underline{\text{H}}_2)_{Hex}$, *qd*, *J* = 6.8 *Hz*, 2H), 1.70 – 1.61 ($(\text{C}\underline{\text{H}}_2)_{Hex}$, *m*, 2H), 1.44 – 1.19 (7×$(\text{C}\underline{\text{H}}_2)_{Hex}$, *m*, 14H), 0.88 ($(\text{C}\underline{\text{H}}_3)_{Hex}$, *t*, *J* = 6.8 *Hz*, 3H). ^{13}C NMR (126 *MHz*, $CDCl_3$) δ 166.9 (CO), 151.9, 149.2, 137.0, 132.8, 129.9, 127.6, 127.5, 127.1, 121.9, 40.4 $(\underline{\text{C}}\text{H}_2)_{Hex}$, 31.9 $(\underline{\text{C}}\text{H}_2)_{Hex}$, 29.7 $(\underline{\text{C}}\text{H}_2)_{Hex}$, 29.6 (2×$(\underline{\text{C}}\text{H}_2)_{Hex}$), 29.4 $(\underline{\text{C}}\text{H}_2)_{Hex}$, 29.3 $(\underline{\text{C}}\text{H}_2)_{Hex}$, 27.1 $(\underline{\text{C}}\text{H}_2)_{Hex}$, 22.7 $(\underline{\text{C}}\text{H}_2)_{Hex}$, 14.1 $(\underline{\text{C}}\text{H}_3)_{Hex}$. MS (EI): *m/z* (rel. int, %): MS m/z (rel. int.): 312 (12, [M^+]), 269 (2), 227 (14), 199 (10), 156 (100).

N-Decyl-2-oxo-2-(quinolin-6-yl)acetamide **(3g)**

Yield: 275 mg (81%); pale yellow solid, m.p: 89-92 °C, R_f (90 % $CHCl_3$, 10 % EtOAc) 0.50; IR (KBr, ν (cm^{-1})): 3313 *m*, 2954 *w*, 2919 *s*, 2850 *s*, 1689 *m*, 1643 *vs*, 1617 *m*, 1572 *w*, 1527 *m*, 1474 *w*, 1461 *w*, 1170 *m*, 1120 *m*, 921 *w*, 817 *m*, 800 *w*. ^{1}H NMR (500 *MHz*, $CDCl_3$) δ 9.16 (H_{Ar}, *d*, *J* = 1.7 *Hz*, 1H), 8.98 (H_{Ar}, *dd*, *J* = 4.2, 1.6 *Hz*, 1H), 8.37 (H_{Ar}, *dd*, *J* = 8.9, 1.8 *Hz*, 1H), 8.25 (H_{Ar}, *d*, *J* = 8.2 *Hz*, 1H), 8.10 (H_{Ar}, *d*, *J* = 8.9 *Hz*, 1H), 7.43 (H_{Ar}, *dd*, *J* = 8.3, 4.2 *Hz*, 1H), 7.41 (NH, *br s*, 1H), 3.40 ($(C\underline{H}_2)_{Hex}$, *qd*, *J* = 6.9 *Hz*, 2H), 1.68 – 1.53 ($(C\underline{H}_2)_{Hex}$, *qt*, 2H), 1.33 – 1.16 ($(C\underline{H}_2)_{Hex}$, *m*, 14H), 0.84 ($(C\underline{H}_3)_{Hex}$, *t*, *J* = 6.9 *Hz*, 3H). ^{13}C NMR (126 *MHz*, $CDCl_3$) δ 186.9 (CO), 161.7 (CO), 153.2, 150.5, 138.1, 134.7, 131.1, 129.9, 129.0, 127.328, 122.0, 39.6 $(\underline{C}H_2)_{Hex}$, 31.9 $(\underline{C}H_2)_{Hex}$, 29.6 $(\underline{C}H_2)_{Hex}$, 29.5 $(\underline{C}H_2)_{Hex}$, 29.3 $(\underline{C}H_2)_{Hex}$, 29.3 $(\underline{C}H_2)_{Hex}$, 29.2 $(\underline{C}H_2)_{Hex}$, 26.9 $(\underline{C}H_2)_{Hex}$, 22.7 $(\underline{C}H_2)_{Hex}$, 14.1 $(\underline{C}H_3)_{Hex}$. MS (EI): *m/z* (rel. int, %): 340 (7, [M^+]), 156 (100), 129 (23), 101(7), 43 (9).

Methyl (quinoline-6-carbonyl)glycinate **(2h)**

Yield: 194 mg (80%); yellow solid, m.p: 125-127 °C, R_f (70 % $CHCl_3$, 30 % EtOAc) 0.30; IR (KBr, ν (cm^{-1})): 3277 *m/br*, 2920 *w*, 1751 *vs*, 1749 *vs*, 1650 *s*, 1622 *m*, 1541 *s*, 1498 *m*, 1205 *s*, 838 *m*. ^{1}H NMR (500 *MHz*, $CDCl_3$) δ 8.98 (H_{Ar}, *dd*, *J* = 4.2, 1.7 *Hz*, 1H), 8.33 (H_{Ar}, *d*, *J* = 1.6 *Hz*, 1H), 8.21 (H_{Ar}, *dd*, *J* = 8.3, 0.9 *Hz*, 1H), 8.12 (H_{Ar}, *d*, *J* = 8.8 *Hz*, 1H), 8.08 (H_{Ar}, *dd*, *J* = 8.8, 1.9 *Hz*, 1H), 7.46 (H_{Ar}, *dd*, *J* = 8.3, 4.3 *Hz*, 1H), 7.25 (NH, *br s*, 1H), 4.31 ($(C\underline{H}_2)_{Gly}$, *d*, *J* = 5.2 *Hz*, 2H), 3.82 (O-C$\underline{H}_3$, *s*, 3H). ^{13}C NMR (126 *MHz*, $CDCl_3$) δ 170.6 (CO), 166.9 (CO), 151.8, 149.0, 137.4, 131.7, 129.7, 127.9, 127.5, 127.3, 122.0, 52.6 $(\underline{C}H_2)_{Gly}$, 41.9 (O-$\underline{C}H_3$). MS (EI): *m/z* (rel. int, %): 244 (25, [M^+]), 207 (13) 185 (16), 156 (100), 128 (46), 101(18), 40 (45).

Methyl (2-oxo-2-(quinolin-6-yl)acetyl)glycinate **(3h)**

Yield: 155 mg (57%); pale yellow solid, m.p: 77-80 °C, R_f (90 % $CHCl_3$, 10 % EtOAc , 1 % MeOH) 0.30; IR (KBr, ν (cm^{-1})): 3428 *m/br*, 3031 *w*, 2923 *w*, 2919 *w*, 2844 *w*, 1757 *vs*, 1680 *w*, 1652 *m*, 1454 *w*, 1215 *m*, 1174 *s*, 1119 *s*, 921 *w*, 851 *w*. ^{1}H NMR (500 *MHz*, $CDCl_3$) δ 9.00 (H_{Ar}, *s*, 1H), 8.91 (H_{Ar}, *dd*, *J* = 4.0, 1.0 *Hz*, 1H), 8.28 (H_{Ar}, *dd*, *J* = 8.9, 1.3 *Hz*, 1H), 8.18 (H_{Ar}, *d*, *J* = 8.2 *Hz*, 1H), 8.06 (NH, *br s*, 1H), 8.02 (H_{Ar}, *d*, *J* = 8.9 *Hz*, 1H), 7.38 (H_{Ar}, *dd*, *J* = 8.2, 4.2 *Hz*, 1H), 4.18 ($(C\underline{H}_2)_{Gly}$, *d*, *J* = 5.8 *Hz*, 2H), 3.73 ($(OC\underline{H}_3)_{Gly}$, *s*, 3H). ^{13}C NMR (126 *MHz*, $CDCl_3$) δ 186.0 (CO), 169.4 (CO), 162.1 (CO), 153.3, 150.4, 138.1, 134.6, 130.7, 129.8, 128.8, 127.2, 122.0, 52.5 $(\underline{C}H_2)_{Gly}$, 41.1 (O-$\underline{C}H_3$). MS (EI): *m/z* (rel. int, %): 272 (10, [M^+]), 156 (100), 128 (50), 101(18).

*Methyl (quinoline-6-carbonyl)-*L*-alaninate* **(2i)**

Yield: 178 mg (69%); yellow waxy solid, R_f (70 % $CHCl_3$, 30 % EtOAc , 2 % MeOH) 0.20; IR (KBr, ν (cm^{-1})): 3266 *m/br*, 2953 *w*, 1751 *vs*, 1658 *s*, 1539 *s*, 1501 *s*, 1300 *m*, 1050 *m*, 843 *m*. ^{1}H NMR (500 *MHz*, $CDCl_3$) δ 8.95

(H_{Ar}, *dd*, *J* = 4.2, 1.5 *Hz*, 1H), 8.30 (H_{Ar}, *d*, *J* = 1.5 *Hz*, 1H), 8.18 (H_{Ar}, *dd*, *J* = 8.2, 0.9 *Hz*, 1H), 8.09 (H_{Ar}, *d*, *J* = 8.8 *Hz*, 1H), 8.06 (H_{Ar}, *dd*, *J* = 8.8, 1.8 *Hz*, 1H), 7.43 (H_{Ar}, *dd*, *J* = 8.3, 4.2 *Hz*, 1H), 7.26 (NH, *s*, 1H), 4.85 ((-*C$\underline{H}$-)$_{Ala}$, *p*, *J* = 7.2 Hz, 1H), 3.80 ((OC$\underline{H}_3$)$_{Ala}$, *s*, 3H), 1.56 ((C$\underline{H}_3$)$_{Ala}$, *d*, *J* = 7.2 Hz, 3H). ^{13}C NMR (126 *MHz*, $CDCl_3$) δ 173.7 (CO), 166.3 (CO), 151.8, 149.0, 137.3, 131.8, 129.6, 127.9, 127.5, 127.4, 121.9, 52.7 (-*$\underline{C}$H-)$_{Ala}$, 48.7 (O$\underline{C}H_3$)$_{Ala}$, 18.4 ($\underline{C}H_3$)$_{Ala}$. MS (EI): *m/z* (rel. int, %): 258 (6, [M^+]), 199 (42), 156 (100), 128 (40), 101(16), 77 (7), 44 (14), 40 (16).

*Methyl (2-oxo-2-(quinolin-6-yl)acetyl)-*L*-alaninate* **(3i)**

Yield: 86 mg (30%); light oil, R_f (70 % $CHCl_3$, 30 % EtOAc) 0.30; IR (KBr, ν (cm^{-1})): 3369 *w/br*, 2956 *w*, 1745 *s*, 1667 *vs*, 1620 *m*, 1522 *m*, 1458 *m*, 1215 *m*, 1172 *s*, 850 *w*. ^{1}H NMR (500 *MHz*, $CDCl_3$): δ 9.17 (H_{Ar}, *dd*, *J* = 7.0, 1.9 *Hz*, 1H), 9.08 – 8.97 (H_{Ar}, *m*, 1H), 8.48 – 8.36 (H_{Ar}, *m*, 1H), 8.32 (H_{Ar}, *t*, *J* = 6.1 *Hz*, 1H), 8.16 (H_{Ar}, *dd*, *J* = 10.1, 5.5 *Hz*, 1H), 7.78 (NH, *s*, 1H), 7.51 (H_{Ar}, *d*, *J* = 10.8 *Hz*, 1H), 4.72 ((-*C$\underline{H}$-)$_{Ala}$, *pd*, *J* = 7.3, 1.5 *Hz*, 1H), 3.82 ((C$\underline{H}_3$)$_{Ala}$, *d*, *J* = 2.2 *Hz*, 3H), 1.57 ((OC$\underline{H}_3$)$_{Ala}$, *dd*, *J* = 7.1, 2.2 *Hz*, 3H). ^{13}C NMR (126 *MHz*, $CDCl_3$): δ 185.9 (CO), 172.4 (CO), 161.1 (CO), 153.1, 150.3, 138.4, 134.7, 130.9, 129.8, 129.1, 127.3, 122.1, 52.7 (-*$\underline{C}$H-)$_{Ala}$, 48.3 (O$\underline{C}H_3$)$_{Ala}$, 18.0 ($\underline{C}H_3$)$_{Ala}$. MS (EI): *m/z* (rel. int, %): 286 (7, [M^+]), 199 (42), 156 (100), 128 (44), 101(18), 77 (7), 44 (6), 40 (6).

*Methyl (quinoline-6-carbonyl)-*L*-valinate* **(2j)**

Yield: 135 mg (47%); pale yellow solid, m.p: 99-101 °C, R_f (85 % $CHCl_3$, 15 % EtOAc) 0.20; IR (KBr, ν (cm^{-1})): 3447 *s/br*, 2965 *m*, 1743 *vs*, 1653 *vs*, 1647 *s*, 1539 *s*, 1497 *m*, 1202 *m*, 1050 *m*, 801 *m*. ^{1}H NMR (500 *MHz*, $CDCl_3$) δ 8.94 (H_{Ar}, *dd*, *J* = 4.1, 1.5 *Hz*, 1H), 8.27 (H_{Ar}, *d*, *J* = 1.4 *Hz*, 1H), 8.16 (H_{Ar}, *d*, *J* = 8.2 *Hz*, 1H), 8.09 (H_{Ar}, *d*, *J* = 8.8 *Hz*, 1H), 8.05 (H_{Ar}, *dd*, *J* = 8.8, 1.8 *Hz*, 1H), 7.41 (H_{Ar}, *dd*, *J* = 8.3, 4.2 *Hz*, 1H), 7.03 (H_{Ar}, *d*, *J* = 8.4 *Hz*, 1H), 4.81 ((-*C$\underline{H}$-)$_{Val}$, *dd*, *J* = 8.6, 5.1 *Hz*, 1H), 3.77 ((OC$\underline{H}_3$)$_{Val}$, *s*, 3H), 2.30 ((C$\underline{H}$(CH_3)$_{a,b}$)$_{Val}$, *m*, 1H), 1.03 (((CH_3)$_b$-CH($\underline{CH_3}$)$_a$)$_{Val}$, *d*, *J* = 6.5 *Hz*, 3H), 1.00 ((($\underline{CH_3}$)$_b$CH(CH_3)$_a$)$_{Val}$, *d*, *J* = 7.0 *Hz*, 3H). ^{13}C NMR (126 *MHz*, $CDCl_3$) δ 172.7 (CO), 166.8 (CO), 152.0, 149.3, 137.0, 132.0, 129.9, 127.8, 127.5, 127.2, 121.9, 57.7 (O$\underline{C}H_3$)$_{Val}$, 52.3 (-*$\underline{C}$H-)$_{Val}$, 31.6 ($\underline{C}$H(CH_3)$_{a,b}$)$_{Val}$, 19.1 ((CH_3)$_b$-CH($\underline{C}H_3$)$_a$)$_{Val}$, 18.1 (($\underline{C}H_3$)$_b$-CH(CH_3)$_a$)$_{Val}$. MS (EI): *m/z* (rel. int, %): 286 (4, [M^+]), 172 (20), 156 (100), 128 (35), 101(13).

*Methyl (2-oxo-2-(quinolin-6-yl)acetyl)-*L*-valinate* **(3j)** (Title compound not completely separated from byproduct).

Yield: 73 mg (23%); light oil, R_f (85 % $CHCl_3$, 15 % EtOAc) 0.30; IR (KBr, ν (cm^{-1})): 3401 *w/br*, 2965 *w*, 1743 *s*, 1669 *vs*, 1620 *m*, 1540 *m*, 1459 *w*, 1436 *w*, 1274 *w*, 1211 *w*, 1169 *m*, 813 *w*. ^{1}H NMR (500 *MHz*, $CDCl_3$) δ 9.24 (NH, *d*, *J* = 1.5 *Hz*, 1H), 9.06 (H_{Ar}, *dd*, *J* = 4.2, 1.4 *Hz*, 1H), 8.47 (H_{Ar}, *dd*, *J* = 8.9, 1.8 *Hz*, 1H), 8.38 (H_{Ar}, *d*, *J* = 8.2 *Hz*, 1H), 8.23 (H_{Ar}, *d*, *J* = 8.9 *Hz*, 1H), 7.69 (H_{Ar}, *d*, *J* = 8.8 *Hz*, 1H), 7.55 (H_{Ar}, *dd*, *J* = 8.3, 4.3 *Hz*, 1H), 4.66 ((-*C$\underline{H}$-)$_{Val}$, *dd*, *J* = 9.0, 5.0 *Hz*, 1H), 3.82 ((OC$\underline{H}_3$)$_{Val}$, *s*, 3H), 2.41 – 2.28 ((C$\underline{H}$(CH_3)$_{a,b}$)$_{Val}$, *m*, 1H), 1.06 (((CH_3)$_b$-

CH($\underline{CH_3}$)$_a$)$_{Val}$, *d*, *J* = 6.9 *Hz*, 3H), 1.03 ((($\underline{CH_3}$)$_b$CH(CH_3)$_a$)$_{Val}$, *d*, *J* = 6.9 *Hz*, 3H). ^{13}C NMR (126 *MHz*, $CDCl_3$) δ 185.9 (CO), 171.4 (CO), 161.3 (CO), 152.9, 140.0, 138.8, 134. 8, 131.2, 129.6, 129.5, 127.4, 122.1, 57.5 (O$\underline{C}H_3$)$_{Val}$, 52.4 (-*$\underline{C}$H-)$_{Val}$, 31.5 ($\underline{C}$H(CH_3)$_{a,b}$)$_{Val}$, 19.1 ((CH_3)$_b$-CH($\underline{C}H_3$)$_a$)$_{Val}$, 17.8 (($\underline{C}H_3$)$_b$-CH(CH_3)$_a$)$_{Val}$. MS (EI): *m/z* (rel. int, %): 314 (14, [M^+]), 277 (16), 207 (8), 156 (100), 128 (40), 101(14).

*Methyl (*L*)-2-phenyl-2-(quinoline-6-carboxamido)acetate* **(2k)**

Yield: 118 mg (37%); light oil, R_f (85 % $CHCl_3$, 15 % EtOAc) 0.20; IR (KBr, ν (cm^{-1})): 3247 *w/br*, 2954 *w*, 1741 *vs*, 1641 *s*, 1621 *m*, 1540 *s*, 1501 *m*, 1318 *m*, 1095 *m*, 739 *m*. ^{1}H NMR (500 *MHz*, $CDCl_3$) δ 8.93 (H_{Ar}, *dd*, *J* = 4.1, 1.4 *Hz*, 1H), 8.31 (H_{Ar}, *s*, 1H), 8.14 (H_{Ar}, *d*, *J* = 8.2 *Hz*, 1H), 8.09 – 8.05 (H_{Ar}, *m*, 2H), 7.62 (NH, *d*, *J* = 6.8 *Hz*, 1H), 7.47 (H_{Ar}, *d*, *J* = 7.1 *Hz*, 2H), 7.41 (H_{Ar}, *dd*, *J* = 8.3, 4.2 *Hz*, 1H), 7.38-7.43 (H_{Ar}, *m*, 3H), 5.84 ((-*C$\underline{H}$-)$_{Phe}$, *d*, *J* = 7.0 *Hz*, 1H), 3.77 ((OC$\underline{H}_3$)$_{Phe}$, *s*, 3H). ^{13}C NMR (126 *MHz*, $CDCl_3$) δ 171.5 (CO), 166.1 (CO), 152.1, 149.4, 137.0, 136.4, 131.5, 129.9, 129.1 (2×C_{Ar}), 128.7, 128.0, 127.5 (2×C_{Ar}), 127.4, 127.3, 121.9, 57.1 (OC$\underline{H}_3$)$_{Phe}$, 53.0 (-*C$\underline{H}$-)$_{Phe}$. MS (EI): *m/z* (rel. int, %): 320 (4, [M^+]), 281 (10), 261 (35), 207 (26), 191 (4), 156 (100), 128 (43), 101(14), 77(10), 44 (33).

*Methyl (*L*)-2-(2-oxo-2-(quinolin-6-yl)acetamido)-2-phenylacetate* **(3k)**

Yield: 112 mg (32%); pale yellow solid, m.p: 133-136 °C, R_f (85 % $CHCl_3$, 15 % EtOAc) 0.40 ; IR (KBr, ν (cm^{-1})): 3393 *m*, 3028*w*, 2958 *w*, 2847 *w*, 1746 *vs*, 1667 *vs*, 1620 *m*, 1522 *m*, 1169 *s*, 812 *w*, 700 *m*. ^{1}H NMR (500 *MHz*, $CDCl_3$) δ 9.23 (H_{Ar}, d, *J* = 1.7 *Hz*, 1H), 9.06 (H_{Ar}, *dd*, *J* = 4.2, 1.4 *Hz*, 1H), 8.47 (H_{Ar}, *dd*, *J* = 8.9, 1.9 *Hz*, 1H), 8.36 (H_{Ar}, *d*, *J* = 8.1 *Hz*, 1H), 8.23 (H_{Ar}, *d*, *J* = 8.9 *Hz*, 1H), 8.17 (NH, *d*, *J* = 7.0 *Hz*, 1H), 7.54 (*dd*, *J* = 8.3, 4.3 *Hz*, 1H), 7.49 – 7.38 (*m*, 5H), 5.67 ((-*C$\underline{H}$-)$_{Phenylglycine}$, *d*, *J* = 7.2 *Hz*, 1H), 3.82 ((O$\underline{CH_3}$)$_{Phenylglycine}$, *s*, 3H). ^{13}C NMR (126 *MHz*, $CDCl_3$) δ 185.4 (CO), 170.4 (CO), 160.6 (CO), 152.5, 149.5, 139.3, 135.5, 134.8, 131.2, 129.7, 129.6, 129.2 (2×C_{Ar}), 129.0 (2×C_{Ar}), 127.4 (2×C_{Ar}), 122.1, 56.7 (OC$\underline{H}_3$)$_{Phe}$, 53.1 (-*C$\underline{H}$-)$_{Phe}$. MS (EI): *m/z* (rel. int, %): 348 (1, [M^+]), 289 (13), 281 (32), 253 (14), 207 (82), 191 (11), 156 (87), 128 (44), 44 (100).

*Methyl (quinoline-6-carbonyl)-*L*-prolinate* **(2l)**

Yield: 199 mg (70%); light yellowish oil, R_f (80 % $CHCl_3$, 20 % EtOAc , 1 % MeOH) 0.20; ((80:20) *Mixture of trans–cis Amide Rotamers*); IR (KBr, ν (cm^{-1})): 3447 *w/br*, 2954 *w*, 1743 *s*, 1633 *vs*, 1614 *s*, 1594 *w*, 1465 *m*, 1433 *m*, 1414 *m*, 1175 *m*, 850 *w*. ^{1}H NMR (500 *MHz*, $CDCl_3$) (*Data for Major Rotamer*) δ 8.90 (H_{Ar}, *d*, *J* = 4.0 *Hz*, 1H), 8.15 (H_{Ar}, *d*, *J* = 8.2 *Hz*, 1H), 8.09 (H_{Ar}, *d*, *J* = 8.7 *Hz*, 1H), 8.02 (H_{Ar}, *s*, 1H), 7.85 (H_{Ar}, *dd*, *J* = 8.5, 1.5 *Hz*, 1H), 7.40 (H_{Ar}, *dd*, *J* = 8.3, 4.2 *Hz*, 1H), 4.67 ((-*C$\underline{H}$-)$_{Pro}$, *dd*, *J* = 8.3, 5.1 *Hz*, 1H), 3.74 ((OC$\underline{H}_3$)$_{Pro}$, *s*, 3H), 3.65 ((-C$\underline{H}_a$-)$_{Pro}$, *m*, 1H), 3.55 – 3.49 ((-C$\underline{H}_b$-)$_{Pro}$, *m*, 1H), 2.26 – 2.33 ((-C$\underline{H}_{a'}$-)$_{Pro}$, *m*, 1H), 1.95–2.09 ((-C$\underline{H}_2$-)$_{Pro}$, *m*, 2H), 1.88 – 1.85 ((-C$\underline{H}_{b'}$-)$_{Pro}$, *m*, 1H). ^{13}C NMR (126 *MHz*, $CDCl_3$) (*Data for Major Rotamer*) δ 172.6 (CO), 168.9 (CO), 151.5, 148.4,

136.8, 134.3, 129.5, 128.0, 127.5, 127.3, 121.8, 59.2 (-*C̲H-)$_{Pro}$, 52.3 (OC̲H3)$_{Pro}$, 50.00 (-C̲H2-)$_{Pro}$, 29.4 (-C̲H2-)$_{Pro}$, 25.4 (-C̲H2-)$_{Pro}$.

^{1}H NMR (500 *MHz*, $CDCl_3$) (*Data for Minor Rotamer*) δ 8.90 (H_{Ar}, *br s, overlapped*, 1H), 8.10 (H_{Ar}, *br s, overlapped*, 1H), 8.05 (H_{Ar}, *br s, overlapped*, 1H), 7.80 (H_{Ar}, *br s*, 1H), 7.66 (H_{Ar}, *d, J* = 8.5 *Hz*, 1H), 7.38 (H_{Ar}, *br s, overlapped*, 1H), 4.34 ((-*CH̲-)$_{Pro}$, *d, J* = 6.5 *Hz*, 1H), 3.77-3.79 ((-$\underline{H}_a$C$\underline{H}_b$-)$_{Pro}$, *m*, 2H), 3.44 ((OC$\underline{H_3}$)$_{Pro}$, *s*, 3H), 2.18–2.23 ((-C$\underline{H}_{a'}$-)$_{Pro}$, *m*, 1H), 1.95 – 2.09 ((-C$\underline{H}_{b'}$-)$_{Pro}$ + (-C$\underline{H_2}$-)$_{Pro}$, *m, overlapped,* 3H). ^{13}C NMR (126 *MHz*, $CDCl_3$) (*Data for Minor Rotamer*) δ 172.7 (CO), 169.7 (CO), 151.3, 148.1, 136.6, 135.0, 129.8, 128.1, 127.7, 127.4, 126.2, 61.4 (-*C̲H-)$_{Pro}$, 52.1 (-OC̲H3-)$_{Pro}$, 46.7 (-C̲H2-)$_{Pro}$, 31.4 (-C̲H2-)$_{Pro}$, 22.7 (-C̲H2-)$_{Pro}$. MS (EI): *m/z* (rel. int, %) : 284 (8, [M^+]), 225 (37), 207 (18), 156 (100), 128 (37), 101(14), 44 (25).

*Methyl (2-oxo-2-(quinolin-6-yl)acetyl)-*L*-prolinate* **(3l)**, ((52:48*) Mixture of trans–cis Amide Rotamers*).

Yield: 235 mg (75%); brownish oil, R_f (80 % $CHCl_3$, 20 % EtOAc , 2 % MeOH) 0.40; IR (KBr, ν (cm^{-1})): 3447 *w/br*, 2984 *w*, 2957 *w*, 2883 *w*, 1744 *s*, 1684 *m*, 1643 *vs*, 1621 *m*, 1447 *m*, 1360 *w*, 1323 *w*, 1170 *m*, 1118 *w*, 805 *w*. ^{1}H NMR (500 *MHz*, $CDCl_3$) (*Data for Major Rotamer*) δ 8.98 (H_{Ar}, *br s*, 1H), 8.63 (H_{Ar}, *d, J* = 1.7 *Hz*, 1H), 8.30 (H_{Ar}, *d, J* = 9.0 *Hz*, 1H), 8.26 (H_{Ar}, *dd, J* = 4.6, 2.3 *Hz*, 1H), 8.15 (H_{Ar}, *d, J* = 8.8 *Hz*, 1H), 7.46 (H_{Ar}, *dd, J* = 8.2, 4.2 *Hz*, 1H), 4.67 ((-*CH̲-)$_{Pro}$, *dd, J* = 8.9, 4.1 *Hz*, 1H), 3.81 ((OC$\underline{H_3}$)$_{Pro}$, *s*, 3H), 3.55 – 3.62 ((-C$\underline{H}_a$-)$_{Pro}$, *m*, 1H), 3.47 – 3.53 ((-C$\underline{H}_b$-)$_{Pro}$, *m*, 1H), 2.31 – 2.36 ((-C$\underline{H}_{a'}$-)$_{Pro}$, *m*, 1H), 2.13 – 2.21 ((-C$\underline{H}_{b'}$-)$_{Pro}$, *m*, 1H), 1.93 – 2.01 ((-C$\underline{H_2}$-)$_{Pro}$, *m*, 2H). ^{13}C NMR (126 *MHz*, $CDCl_3$) (*Data for Major Rotamer*) δ 190.4 (CO), 171.8 (CO), 165.0 (CO), 153.3, 150.6, 138.2, 133.8, 130.7, 130.5, 127.6, 127.6, 122.2, 58.4 (-*C̲H-)$_{Pro}$, 52.6 (-OC̲H3-)$_{Pro}$, 47.3 (-C̲H2-)$_{Pro}$, 29.1 (-C̲H2-)$_{Pro}$, 24.7 (-C̲H2-)$_{Pro}$.

^{1}H NMR (500 *MHz*, $CDCl_3$) (*Data for Minor Rotamer*) δ 8.95 (H_{Ar}, *br s*, 1H), 8.63 (H_{Ar}, *s*, 1H), 8.28 (H_{Ar}, *d, J* = 4.3 *Hz*, 1H), 8.25 (H_{Ar}, *dd, J* = 9.0, 2.0 *Hz*, 1H), 8.12 (H_{Ar}, *d, J* = 8.9 *Hz*, 1H), 7.43 (H_{Ar}, *d, J* = 4.3 *Hz*, 1H), 4.79 ((-*CH̲-)$_{Pro}$, *dd, J* = 8.5, 3.8 *Hz*, 1H), 3.82 – 3.88 ((-C$\underline{H}_a$-)$_{Pro}$, *m*, 1H), 3.67 – 3.74 ((-C$\underline{H}_b$-)$_{Pro}$, *m*, 1H), 3.41 ((OC$\underline{H_3}$)$_{Pro}$, *s*, 3H), 2.24 – 2.30 ((-C$\underline{H}_{a'}$-)$_{Pro}$, *m*, 1H), 2.02 – 2.09 ((-C$\underline{H}_{b'}$-)$_{Pro}$, *m*, 1H), 1.82 – 1.92 ((-C$\underline{H_2}$-)$_{Pro}$, *m*, 2H). ^{13}C NMR (126 *MHz*, $CDCl_3$) (*Data for Minor Rotamer*) δ 189.4 (CO), 172.1 (CO), 164.0 (CO), 152.9, 150.2, 138.1, 133.5, 131.1, 129.8, 128.5, 127.3, 122.0, 59.4 (-*C̲H-)$_{Pro}$, 52.5 (-OC̲H3-)$_{Pro}$, 46.8 (-C̲H2-)$_{Pro}$, 31.0 (-C̲H2-)$_{Pro}$, 22.5 (C̲H2-)$_{Pro}$. MS (EI): *m/z* (rel. int, %): 312 (1, [M^+]), 284 (20), 207 (10), 156 (88), 128 (100), 101(15), 44 (25).

Methyl (quinoline-6-carbonyl)-(L/D)*-serinate* **(2m)**

Yield: 228 mg (83%); white solid, m.p: 174-177 °C, R_f (70 % $CHCl_3$, 30 % EtOAc , 2 % MeOH) 0.20; IR (KBr, ν (cm^{-1})): 3449 *w/br*, 3280 *m*, 3180 *w/br*, 2954 *vw*, 1740 *s*, 1642 *vs*, 1632 *m*, 1540 *m*, 1233 *w*, 1074 *m*, 1414 *m*, 847 *w*. ^{1}H NMR (500 *MHz*, DMSO-d_6) δ 9.01 (H_{Ar}, *dd, J* = 4.2, 1.6 *Hz*, 1H), 8.87 (H_{Ar}, *d, J* = 7.4 *Hz*, 1H), 8.59 (NH, *d, J*

= 1.7 *Hz*, 1H), 8.51 (H_{Ar}, *d*, *J* = 7.7 *Hz*, 1H), 8.22 (H_{Ar}, *dd*, *J* = 8.8, 1.9 *Hz*, 1H), 8.11 (H_{Ar}, *d*, *J* = 8.8 *Hz*, 1H), 7.63 (H_{Ar}, *dd*, *J* = 8.3, 4.2 *Hz*, 1H), 5.14 ($(\text{-OH})_{Ser}$, *t*, *J* = 6.1 *Hz*, 1H), 4.63 ($(\text{-*C}\underline{H}\text{-})_{Ser}$, *qd*, *J* = 5.0 *Hz*, 1H), 3.79-3.93 ($(\text{-}\underline{H}_b\text{C}\underline{H}_a\text{-})_{Ser}$, *m*, 2H), 3.69 ($(\text{OC}\underline{H_3})_{Ser}$, *s*, 3H). ^{13}C NMR (126 *MHz*, DMSO-d6) δ 171.5 (CO), 166.6 (CO), 152.7, 149.2, 137.6, 132.0, 129.5, 128.8, 128.3, 127.5, 122.7, 61.5 $(\text{-*}\underline{C}\text{H-})_{Ser}$, 56.3 $(\text{-}\underline{C}\text{H}_2\text{-OH})_{Ser}$, 52.4 $(\text{O}\underline{C}\text{H}_3)_{Ser}$. MS (EI): *m/z* (rel. int, %): 274 (1, [M$^+$]), 256 (20), 224 (10), 197 (20), 156 (100), 128 (50), 101(16), 51 (5).

Methyl (2-oxo-2-(quinolin-6-yl)acetyl)-(L/D)*-serinate* **(3m)**

Yield: 91 mg (30%); orange solid, m.p: 144-146 °C, R_f (70 % $CHCl_3$, 30 % EtOAc , 2 % MeOH) 0.40; IR (KBr, ν (cm^{-1})): 3398 *w*, 3199 *w/br*, 2968 *w*, 1744 *s*, 1684 *w*, 1656 *vs*, 1617 *m*, 1516 *m*, 1464 *w*, 1431 *w*, 1325 *m*, 1223 *m*, 1171 *m*, 1084 *m*, 815 *m*. ^{1}H NMR (500 *MHz*, $CDCl_3$) δ 9.14 (NH, *d*, *J* = 1.6 *Hz*, 1H), 9.06 (H_{Ar}, *dd*, *J* = 4.3, 1.6 *Hz*, 1H), 8.39 – 8.34 (H_{Ar}, *m*, 2H), 8.18 (H_{Ar}, *d*, *J* = 8.9 *Hz*, 1H), 8.10 (H_{Ar}, *d*, *J* = 7.7 *Hz*, 1H), 7.57 (H_{Ar}, *dd*, *J* = 8.3, 4.4 *Hz*, 1H), 4.81 ($(\text{-*C}\underline{H}\text{-})_{Ser}$, *dt*, J = 7.4, 3.5 *Hz*, 1H), 4.21 ($(\text{-H}_b\text{C}\underline{H}_a\text{-})_{Ser}$, *dd*, *J* = 11.5, 3.7 *Hz*, 1H), 4.12 ($(\text{-}\underline{H}_b\text{CH}_a\text{-})_{Ser}$, *dd*, *J* = 11.5, 3.4 *Hz*, 1H), 3.87 ($(\text{OC}\underline{H_3})_{Ser}$, *s*, 3H). ^{13}C NMR (126 *MHz*, $CDCl_3$) δ 185.6 (CO), 170.1 (CO), 161.6, 152.6, 149.5, 139.2, 134.7, 131.1, 129.5, 129.2, 127.4, 122.1, 62.69 $(\text{-}\underline{C}\text{H}_2\text{-OH})_{Ser}$, 54.8 $(\text{O}\underline{C}\text{H}_3)_{Ser}$, 53.0 $(\text{-*}\underline{C}\text{H-})_{Ser}$. MS (EI): *m/z* (rel. int, %): 302 (1, [M$^+$]), 241 (6), 207 (13), 183 (54), 156 (100), 128 (36), 101(10), 59 (6).

N-Phenylquinoline-6-carboxamide **(2n)**

Yield: 169 mg (68%); pale yellow solid, m.p: 155-156 °C, R_f (85 % $CHCl_3$, 15 % EtOAc , 1 % MeOH) 0.25; IR (KBr, ν (cm^{-1})): 3315 *w/br*, 3180 *w/br*, 1652 *vs*, 1598 *m*, 1529 *m*, 1497 *w*, 1444 *w*, 1330 *w*, 1112 *w*, 889 *m*, 757 *m*. ^{1}H NMR (500 *MHz*, $CDCl_3$) δ 8.96 (H_{Ar}, *d*, *J* = 3.8 *Hz*, 1H), 8.53 (H_{Ar}, *s*, 1H), 8.31 (H_{Ar}, *s*, 1H), 8.11 (NH, *s*, 1H), 8.09 (H_{Ar}, *s*, 2H), 7.71 (H_{Ar}, *d*, *J* = 7.9 *Hz*, 2H), 7.42 (H_{Ar}, *dd*, *J* = 8.2, 4.2 *Hz*, 1H), 7.37 (H_{Ar}, *t*, *J* = 7.7 *Hz*, 2H), 7.17 (H_{Ar}, *t*, *J* = 7.4 *Hz*, 1H). ^{13}C NMR (126 *MHz*, $CDCl_3$) δ 165.43(CO), 152.11, 149.30, 137.92, 136.97, 132.91, 130.05, 129.13 (2×C_{Ar}), 127.87, 127.51, 127.18, 124.84, 122.00, 120.54 (2×C_{Ar}). MS (EI): *m/z* (rel. int, %) : 248 (38, [M$^+$]), 156 (100), 128 (52), 101 (20), 77 (10).

N-Benzylquinoline-6-carboxamide **(2o)**

Yield: 34 mg (13%); orange solid, m.p: 124-126 °C, R_f (85 % $CHCl_3$, 15 % EtOAc , 1 % MeOH) 0.20; IR (KBr, ν (cm^{-1})): 3291 *m*, 1638 *vs*, 1558 *s*, 1545 *s*, 1496 *s*, 1416 *m*, 1326 *m*, 1313 *w*, 908 *m*, 858 *m*, 720 *m*. ^{1}H NMR (500 *MHz*, $CDCl_3$) δ 8.98 (H_{Ar}, *dd*, *J* = 4.2, 1.6 *Hz*, 1H), 8.34 (H_{Ar}, *d*, *J* = 1.6 *Hz*, 1H), 8.21 (H_{Ar}, *d*, *J* = 7.8 *Hz*, 1H), 8.14 (H_{Ar}, *d*, *J* = 8.8 *Hz*, 1H), 8.08 (H_{Ar}, *dd*, *J* = 8.8, 1.9 *Hz*, 1H), 7.46 (*dd*, *J* = 8.3, 4.2 *Hz*, 1H), 7.39 (H_{Ar}, *q*, *J* = 7.9 *Hz*, 4H), 7.35 – 7.30 (H_{Ar}, *m*, 1H), 6.82 (NH, *br s*, 1H), 4.72 ($(\text{C}\underline{H_2})_{Bn}$, *d*, *J* = 5.6 *Hz*, 2H). ^{13}C NMR (126 *MHz*, $CDCl_3$) δ 173.3 (CO), 166.8, 152.0, 149.4, 138.0, 137.0, 132.3, 130.0, 128.9, 128.9, 128.0 (2×C_{Ar}), 128.0 (2×C_{Ar}), 127.8,

127.6, 127.1, 122.0, 44.4 ($(\underline{C}H_2)_{Bn}$. MS (EI): *m/z* (rel. int, %): 262 (45, [M^+]), 207 (46), 156 (60), 128 (40), 101 (14), 73 (15), 44 (100).

N-Benzyl-2-oxo-2-(quinolin-6-yl)acetamide **(3o)**

Yield: 206 mg (71%); pale yellow solid, m.p: 109-111 °C, R_f (85 % $CHCl_3$, 15 % EtOAc , 1 % MeOH) 0.30; IR (KBr, ν (cm^{-1})): 3331 *w*, 3078 *w*, 2953 *w*, 1683 *w*, 1652 *vs*, 1618 *w*, 1535 *w*, 1460 *w*, 1455 *w*, 1241 *w*, 1176 *m*, 843 *m*. ^1H NMR (500 *MHz*, $CDCl_3$) δ 9.28 (H_{Ar}, *d*, *J* = 1.6 *Hz*, 1H), 9.04 (H_{Ar}, *d*, *J* = 4.0 *Hz*, 1H), 8.45 (H_{Ar}, *dd*, *J* = 8.9, 1.8 *Hz*, 1H), 8.36 (H_{Ar}, *d*, *J* = 8.2 *Hz*, 1H), 8.20 (H_{Ar}, *d*, *J* = 8.9 *Hz*, 1H), 7.67 (NH, *br s*, 1H), 7.53 (H_{Ar}, *dd*, *J* = 8.3, 4.3 *Hz*, 1H), 7.42 – 7.36 (H_{Ar}, *m*, 4H), 7.34 (H_{Ar}, *dd*, *J* = 11.6, 3.1 *Hz*, 1H), 7.31 – 7.19 (H_{Ar}, *m*, 1H), 4.63 (($C\underline{H}_2)_{Bn}$, *d*, *J* = 6.1 *Hz*, 2H). ^{13}C NMR (126 *MHz*, $CDCl_3$) δ 186.4 (CO), 161.4 (CO), 152.9, 150.1, 138.7, 137.0, 134.9, 131.2, 129.7, 129.4, 128.9, 128.6, 127.9, 127.4, 127.4, 127.2, 122.1, 43.6 ($(\underline{C}H_2)_{Bn}$. MS (EI): *m/z* (rel. int, %): 290 (2, [M^+]), 207 (10), 156 (100), 128 (47), 101 (15), 91 (36), 44 (17).

N-(Pyridin-2-ylmethyl)quinoline-6-carboxamide **(2p)**

Yield: 95 mg (36%); brownish waxy solid, R_f (80 % $CHCl_3$, 20 % EtOAc , 2 % MeOH) 0.20; IR (KBr, ν (cm^{-1})): 3471 *m/br*, 1660 *vs*, 1654 *vs*, 1595 *m*, 1539 *s*, 1533 *m*, 1495 *m*, 1306 *m*, 850 *w*, 785 *m*, 668 *s*. ^1H NMR (500 *MHz*, $CDCl_3$) δ 8.96 (H_{Ar}, *dd*, *J* = 4.2, 1.6 *Hz*, 1H), 8.57 (H_{Ar}, *d*, *J* = 4.8 *Hz*, 1H), 8.41 (H_{Ar}, *d*, *J* = 1.4 *Hz*, 1H), 8.23 (H_{Ar}, *dd*, *J* = 8.3, 1.2 *Hz*, 1H), 8.18 – 8.12 (H_{Ar}, *m*, 2H), 8.07 (NH, *br s*, 1H), 7.70 (H_{Ar}, *td*, *J* = 7.7, 1.7 *Hz*, 1H), 7.45 (H_{Ar}, *dd*, *J* = 8.3, 4.2 *Hz*, 1H), 7.37 (H_{Ar}, *d*, *J* = 7.8 *Hz*, 1H), 7.27 – 7.19 (H_{Ar}, *m*, 1H), 4.82 (($C\underline{H}_2)_{Pico}$, *d*, *J* = 4.9 *Hz*, 2H). ^{13}C NMR (126 *MHz*, $CDCl_3$) δ 166.7 (CO), 156.1, 151.9, 149.3, 149.0, 137.1, 137.0, 132.2, 129.9, 127.9, 127.6, 127.3, 122.6, 122.4, 121.9, 44.9 ($(\underline{C}H_2)_{Pico}$. MS (EI): *m/z* (rel. int, %): 263 (16, [M^+]), 207 (30), 156 (19), 128 (36), 107 (100), 109 (14), 44 (37), 40 (35).

2-Oxo-N-(pyridin-2-ylmethyl)-2-(quinolin-6-yl)acetamide **(3p)**

Yield: 114 mg (39%); green solid, m.p: 129-131 °C, R_f (80 % $CHCl_3$, 20 % EtOAc , 2 % MeOH) 0.30; IR (KBr, ν (cm^{-1})): 3180 *w*, 3095 *w*, 2943 *w*, 1679 *m*, 1659 *vs*, 1616 *m*, 1572 *m*, 1509 *w*, 1437 *w*, 1234 *w*, 1171 *m*, 922 *w*, 783 *m*. ^1H NMR (500 *MHz*, $CDCl_3$) δ 9.19 (NH, *d*, *J* = 1.7 *Hz*, 1H), 9.02 (H_{Ar}, *dd*, *J* = 4.2, 1.6 *Hz*, 1H), 8.59 (H_{Ar}, *d*, *J* = 4.8 Hz, 1H), 8.43 (H_{Ar}, *dd*, *J* = 8.9, 1.8 *Hz*, 2H), 8.31 (H_{Ar}, *d*, *J* = 8.2 *Hz*, 1H), 8.15 (H_{Ar}, *d*, *J* = 8.9 *Hz*, 1H), 7.71 (H_{Ar}, *td*, *J* = 7.7, 1.7 *Hz*, 1H), 7.49 (H_{Ar}, *dd*, *J* = 8.3, 4.3 *Hz*, 1H), 7.35 (H_{Ar}, *d*, *J* = 7.8 *Hz*, 1H), 7.25 (H_{Ar}, *dd*, *J* = 7.2, 5.2 *Hz*, 1H), 4.75 (($C\underline{H}_2)_{Pico}$, *d*, *J* = 5.4 *Hz*, 2H). ^{13}C NMR (126 *MHz*, $CDCl_3$) δ 186.5 (CO), 161.9 (CO), 155.4, 153.2, 150.4, 149.2, 138.3, 137.1, 134.7, 131.1, 129.9, 129.2, 127.3, 122.8, 122.1, 122.0, 44.3 ($(\underline{C}H_2)_{Pico}$. MS (EI): *m/z* (rel. int, %): 291 (1, [M^+]), 248 (26), 207 (32), 156 (43), 135 (100), 128 (43), 101 (18), 92 (94), 65 (20), 44 (32), 40 (28).

*N-(Pyridin-**3**-ylmethyl)-quinoline-6-carboxamide* **(2q)**

Yield: 159 mg (60%); pale yellow waxy solid, R_f (80 % $CHCl_3$, 20 % EtOAc , 1 % MeOH) 0.20; IR (KBr, ν (cm^{-1})): 3243 *w/br*, 3060 *w* 2925 *w*, 1647 *vs*, 1622 *w*, 1558 *m*, 1430 *w*, 1309 *m*, 824 *w*. ^{1}H NMR (500 *MHz*, $CDCl_3$) δ 8.92 (H_{Ar}, *dd*, *J* = 4.2, 1.6 *Hz*, 1H), 8.56 (H_{Ar}, *s*, 1H), 8.46 (H_{Ar}, *d*, *J* = 4.4 *Hz*, 1H), 8.35 (H_{Ar}, *d*, *J* = 1.6 *Hz*, 1H), 8.14 – 8.03 (H_{Ar}, *m*, 3H), 7.82 (NH, *br s*, 1H), 7.75 (H_{Ar}, *d*, *J* = 7.9 *Hz*, 1H), 7.40 (H_{Ar}, *dd*, *J* = 8.3, 4.2 *Hz*, 1H), 7.25 (H_{Ar}, *dd*, *J* = 7.8, 4.9 *Hz*, 1H), 4.67 ((C$\underline{H}_2$)$_{Pico}$, *d*, *J* = 5.8 *Hz*, 1H). ^{13}C NMR (126 *MHz*, $CDCl_3$) δ 167.1 (CO), 152.0, 149.3, 148.8, 148.4, 137.1, 136.4, 134.4, 131.9, 129.9, 128.0, 127.5, 127.3, 123.9, 122.0, 41.6 ($\underline{C}H_2$)$_{Pico}$. MS (EI): *m/z* (rel. int, %): 263 (77, [M$^+$]), 246 (15), 207 (81), 191(10), 156 (100), 128 (83), 101 (28), 73 (25), 44 (75), 40 (73).

*2-Oxo-N-(pyridin-**3**-ylmethyl)-2-(quinolin-6-yl)acetamide* **(3q)**

Yield: 178 mg (61%); pale yellow solid, m.p: 132-135 °C, R_f (80 % $CHCl_3$, 20 % EtOAc , 5 % MeOH) 0.30; IR (KBr, ν (cm^{-1})): 3218 *w*, 1656 *m*, 1654 *vs*, 1619 *w*, 1576 *w*, 1526 *w*, 1426 *w*, 1256 *w*, 1171 *m*, 914 *w*, 813 *w*, 707 *m*. ^{1}H NMR (500 *MHz*, $CDCl_3$) δ 9.21 (H_{Ar}, *d*, *J* = 1.7 *Hz*, 1H), 9.03 (H_{Ar}, *dd*, *J* = 4.2, 1.7 *Hz*, 1H), 8.67 (H_{Ar}, *d*, *J* = 1.7 *Hz*, 1H), 8.57 (H_{Ar}, *dd*, *J* = 4.8, 1.3 *Hz*, 1H), 8.41 (H_{Ar}, *dd*, *J* = 8.9, 1.9 *Hz*, 1H), 8.32 (H_{Ar}, *d*, *J* = 8.3 *Hz*, 1H), 8.15 (H_{Ar}, *d*, *J* = 8.9 *Hz*, 1H), 7.98 (NH, *s*, 1H), 7.76 (H_{Ar}, *d*, *J* = 7.9 *Hz*, 1H), 7.50 (H_{Ar}, *dd*, *J* = 8.3, 4.3 *Hz*, 1H), 7.34 (H_{Ar}, *dd*, *J* = 7.8, 4.9 *Hz*, 1H), 4.66 ((C$\underline{H}_2$)$_{Pico}$, *d*, *J* = 6.2 *Hz*, 2H). ^{13}C NMR (126 *MHz*, $CDCl_3$) δ 186.2 (CO), 161.7 (CO), 153.4, 150.6, 148.9, 148.8, 138.2, 136.2, 134.9, 133.2, 130.9, 130.1, 129.0, 127.3, 123.9, 122.1, 41.0 ($\underline{C}H_2$)$_{Pico}$. MS (EI): *m/z* (rel. int, %): 291 (1, [M$^+$]), 248 (26), 207 (10), 191(1), 156 (100), 128 (51), 101 (17), 65 (9), 44 (8), 40 (8).

*N-(Pyridin-**4**-ylmethyl)quinoline-6-carboxamide* **(2r)**

Yield: 171 mg (65%); yellow solid, m.p: 140-141 °C, R_f (80 % $CHCl_3$, 20 % EtOAc , 5 % MeOH) 0.30; IR (KBr, ν (cm^{-1})): 3296 *m*, 1637 *s*, 1617 *w*, 1596 *m*, 1543 *s*, 1309 *m*, 856 *w*, 791 *w*. ^{1}H NMR (500 *MHz*, $CDCl_3$) δ 8.97 (H_{Ar}, *dd*, *J* = 4.2, 1.6 *Hz*, 1H), 8.51 (H_{Ar}, *d*, *J* = 6.0 *Hz*, 2H), 8.41 (H_{Ar}, *s*, 1H), 8.19 (H_{Ar}, *dd*, *J* = 8.3, 1.2 *Hz*, 1H), 8.16 – 8.10 (H_{Ar}, *m*, 2H), 7.68 (NH, *br s*, 1H), 7.46 (H_{Ar}, *dd*, *J* = 8.3, 4.2 *Hz*, 1H), 7.31 (H_{Ar}, *d*, *J* = 5.8 *Hz*, 2H), 4.71 ((C$\underline{H}_2$)$_{Pico}$, *d*, *J* = 5.9 *Hz*, 2H). ^{13}C NMR (126 *MHz*, $CDCl_3$) δ 167.2 (CO), 152.1, 149.3, 149.2 (2×$C_{AA'BB'}$), 148.5, 137.1, 131.7, 130.0, 128.1, 127.6, 127.2, 122.7 (2×$C_{AA'BB'}$), 122.1, 43.0 ($\underline{C}H_2$)$_{Pico}$. MS (EI): *m/z* (rel. int, %): 263 (45, [M$^+$]), 207 (83), 156 (67), 128 (46), 101 (16), 73 (28), 44 (100), 40 (50).

2-Oxo-N-(pyridin-4-ylmethyl)-2-(quinolin-6-yl)acetamide **(3r)**

Yield: 154 mg (53%); pale yellow solid, m.p: 120-122 °C, R_f (85 % $CHCl_3$, 15 % EtOAc , 1 % MeOH) 0.40; IR (KBr, ν (cm^{-1})): 3309 *m*, 3031 *w*, 1680 *s*, 1652 *vs*, 1621 *m*, 1521 *m*, 1518 *m*, 1457 *w*, 1252 *w*, 1168 *m*, 1118 *w*, 809

m, 785 *w*. ^{1}H NMR (500 *MHz*, $CDCl_3$) δ 9.07 (NH, *s*, 1H), 8.94 (H_{Ar}, *d*, *J* = 3.8 *Hz*, 1H), 8.47 (H_{Ar}, *dd*, *J* = 10.9, 5.2 *Hz*, 3H), 8.32 (H_{Ar}, *d*, *J* = 8.9 *Hz*, 1H), 8.21 (H_{Ar}, *d*, *J* = 8.3 *Hz*, 1H), 8.07 (H_{Ar}, *d*, *J* = 8.9 *Hz*, 1H), 7.41 (H_{Ar}, *dd*, *J* = 8.2, 4.2 *Hz*, 1H), 7.20 (H_{Ar}, *d*, *J* = 4.9 *Hz*, 2H), 4.57 ((C$\underline{H_2}$)$_{Pico}$, *d*, *J* = 6.3 *Hz*, 2H). ^{13}C NMR (126 *MHz*, $CDCl_3$) δ 186.5 (CO), 162.3 (CO), 153.4, 150.5, 150.0 (2×$C_{AA'BB'}$), 146.4, 138.1, 134.7, 130.8, 130.0, 128.8, 127.3, 122.3, 122.1 (2×$C_{AA'BB'}$), 42.2 ($\underline{C}H_2$)$_{Pico}$. MS (EI): *m/z* (rel. int, %): 291 (1, [M$^+$]), 248 (32), 207 (12), 156 (100), 128 (51), 44 (12).

N-Ethyl-N-(pyridin-4-ylmethyl)quinoline-6-carboxamide **(2s)**

Yield: 169 mg (58%); brownish oil, R_f (80 % $CHCl_3$, 20 % EtOAc , 2 % MeOH) 0.3; IR (KBr, ν (cm^{-1})): 3426 *m/br*, 2996 *w*, 1635 *vs*, 1602 *w*, 1476 *m*, 1415 *m*, 1283 *m*, 844 *w*, 790 *w*. ^{1}H NMR (500 *MHz*, $CDCl_3$) δ 8.94 (H_{Ar}, *br s*, 1H), 8.59 (H_{Ar}, *d*, *J* = 4.5 *Hz*, 2H), 8.13 (H_{Ar}, *br s*, 1H), 7.89 (H_{Ar}, *br s*, 1H), 7.75 (H_{Ar}, *br s*, 1H), 7.44 (H_{Ar}, *br s*, 1H), 7.31 (H_{Ar}, *br s*, 1H), 7.13 (H_{Ar}, *br s*, 1H), 4.66 ((C$\underline{H_2}$)$_{Pico}$, *br*, 2H), 3.37 ((C$\underline{H_2}$-CH_3)$_{Pico}$, *br*, 2H), 1.18 ((CH_2-C$\underline{H_3}$)$_{Pico}$, *br*, 3H). ^{13}C NMR (126 *MHz*, $CDCl_3$) δ 171.4 (CO), 151.5, 150.0 (*br*), 149.8 (*br*), 148.2, 146.9 (*br*), 136.5, 134.2, 130.1, 127.8, 127.3, 126.2, 122.7 (*br*), 122.0, 121.6 (*br*), 46.9 (-CH_2-)$_{Pico}$, 44.0 ($\underline{C}H_2$-CH_3)$_{Pico}$, 14.0 (CH_2-$\underline{C}H_3$)$_{Pico}$. MS (EI): *m/z* (rel. int, %): 291 (40, [M$^+$]), 207 (12), 199 (5), 156 (100), 128 (55), 101 (18), 92 (34), 65 (8).

N-Ethyl-2-oxo-N-(pyridin-4-ylmethyl)-2-(quinolin-6-yl)acetamide **(3s)** ((57:43*) Mixture of trans–cis Amide Rotamers*).

Yield: 86 mg (27%); marron oil, R_f (80 % $CHCl_3$, 20 % EtOAc , 2 % MeOH) 0.2; IR (KBr, ν (cm^{-1})): 3436 *w*, 3050 *w*, 2974 *w*, 2930*w*, 1686 *w*, 1678 *m*, 1643 *vs*, 14016 *m*, 1169 *m*, 1115 *m*, 786 *w*. ^{1}H NMR (500 *MHz*, $CDCl_3$) (*Data for Major Rotamer*) δ 9.08 (H_{Ar}, *dd*, *J* = 4.0, 1.7 *Hz*, 1H), 8.68 (H_{Ar}, *d*, *J* = 6.5 *Hz*, 2×$H_{AA'BB'}$), 8.63 – 8.56 (H_{Ar}, *m*, 1H), 8.51 (H_{Ar}, *d*, *J* = 1.7 *Hz*, 1H), 8.35 – 8.20 (H_{Ar}, *m*, 2H), 7.55 (H_{Ar}, *t*, *J* = 8.1*Hz*, 1H), 7.38 (H_{Ar}, *d*, *J* = 6.0 *Hz*, 2×$H_{AA'BB'}$), 4.82 ((C$\underline{H_2}$)$_{Pico}$, *s*, 2H), 3.34 (C$\underline{H_2}$-CH_3)$_{Pico}$, *qd*, *J* = 7.1 *Hz*, 2H), 1.19 (CH_2-C$\underline{H_3}$)$_{Pico}$ *t*, *J* = 7 *Hz*, 3H). ^{13}C NMR (126 *MHz*, $CDCl_3$) δ 190.2 (CO), 167.3 (CO), 153.6, 150.8, 149.8, 149.5 (2×$C_{AA'BB'}$), 146.6, 137.9, 132.6, 130.91, 127.6, 122.9 (2×$C_{AA'BB'}$), 122.6, 122.4, 46.5 (-$\underline{C}H_2$-), 43.0 ($\underline{C}H_2$-CH_3)$_{Pico}$, 14.0 (CH_2-$\underline{C}H_3$)$_{Pico}$.

^{1}H NMR (500 *MHz*, $CDCl_3$) (*Data for Minor Rotamer*) δ 9.08 (H_{Ar}, *dd*, *J* = 4.0, 1.7 *Hz*, 1H), 8.68 (H_{Ar}, *d*, *J* = 6.5 *Hz*, 2×$H_{AA'BB'}$), 8.63 – 8.56 (H_{Ar}, m, 1H), 8.48 (H_{Ar}, *d*, *J* = 1.7 *Hz*, 1H), 8.35 – 8.20 (H_{Ar}, *m*, 2H), 7.55 (H_{Ar}, *t*, *J* = 8.1*Hz*, 1H), 7.29 (H_{Ar}, *d*, *J* = 6.0 *Hz*, 2×$H_{AA'BB'}$ (+ *s*, 1H, C$\underline{H}Cl_3$)), 4.49 ((CH_2)$_{Pico}$, *s*, 1H), 3.59 (C$\underline{H_2}$-CH_3)$_{Pico}$, *qd*, J = 7.2 *Hz*, 2H), 1.31 (CH_2-C$\underline{H_3}$)$_{Pico}$ *t*, *J* = 7 *Hz*, 3H). ^{13}C NMR (126 *MHz*, $CDCl_3$) δ 190.3 (CO), 166.7 (CO), 153.5, 150.7 (2×$C_{AA'BB'}$), 147.2, 145.2, 137.8, 132.7, 130.92, 130.8, 127.7, 123.3, 122.38 (2×$C_{AA'BB'}$), 121.4, 50.0 (-$\underline{C}H_2$-)$_{Pico}$, 39.9 ($\underline{C}H_2$-CH_3)$_{Pico}$, 12.4 (CH_2-$\underline{C}H_3$)$_{Pico}$. MS (EI): *m/z* (rel. int, %): 319 (1, [M$^+$]), 281(8), 248 (36), 207 (21), 191(3), 156 (100), 128 (38), 101 (14), 92 (60), 65 (11), 44 (18).

N-(Furan-2-ylmethyl)quinoline-6-carboxamide **(2t)**

Yield: 156 mg (62%); orange solid, m.p: 164-166 °C, R_f (80 % $CHCl_3$, 20 % EtOAc , 1 % MeOH) 0.20; IR (KBr, ν (cm^{-1})): 3228 *m*, 1633 *vs*, 1619 *m*, 1539 *s*, 1493 *m*, 1323 *w*, 1307 *m*, 1198 *w*, 910 *w*, 850 *w*, 726 *m*. ^1H NMR (500 *MHz*, $CDCl_3$) δ 8.96 (H_{Ar}, *dd*, *J* = 4.2, 1.6 *Hz*, 1H), 8.34 (H_{Ar}, *d*, *J* = 1.6 *Hz*, 1H), 8.23 (H_{Ar}, *d*, *J* = 8.2 *Hz*, 1H), 8.15 (H_{Ar}, *d*, *J* = 8.8 *Hz*, 1H), 8.09 (H_{Ar}, *dd*, *J* = 8.8, 1.9 *Hz*, 1H), 7.47 (H_{Ar}, *dd*, *J* = 8.3, 4.3 *Hz*, 1H), 7.39 ($(\text{-C=H}_a)_{Furfuryl}$, *J* = 2 *Hz d*, 1H), 7.00 (NH, *br s*, 1H), 6.36 ($(\text{-C=H}_b)_{Furfuryl}$, *t*, *J* = 5 *Hz*, 1H), 6.34 ($(\text{-C=H}_c)_{Furfuryl}$, *d*, *J* = 7 *Hz*, 1H), 4.71 ($(CH_2)_{Furfuryl}$, *d*, *J* = 5.4 *Hz*, 2H). ^{13}C NMR (126 *MHz*, $CDCl_3$) δ 166.5 (CO), 151.5, 151.0, 148.7, 142.4, 137.6, 132.3, 129.5, 127.9, 127.6, 127.5, 121.9, 110.6, 107.9, 37.2 $(CH_2)_{Furfuryl}$. MS (EI): *m/z* (rel. int, %): 252 (50, [M^+]), 207 (23), 156 (100), 128 (58), 101 (17), 44 (50), 40 (69), 53 (12).

N-(Furan-2-ylmethyl)-2-oxo-2-(quinolin-6-yl)acetamide **(3t)**

Yield: 247 mg (88%); orange solid, m.p: 109-111 °C, R_f (85 % $CHCl_3$, 15 % EtOAc, 2 % MeOH) 0.50; IR (KBr, ν (cm^{-1})): 3256 *w*, 1661 *vs*, 1618 *m*, 1517 *w*, 1425 *w*, 1277 *w*, 1168 *m*, 1144 *w*, 814 *m*, 744 *w*. ^1H NMR (500 *MHz*, $CDCl_3$) δ 9.28 (H_{Ar}, *d*, *J* = 1.7 *Hz*, 1H), 9.05 (H_{Ar}, *dd*, *J* = 4.3, 1.6 *Hz*, 1H), 8.46 (H_{Ar}, *dd*, *J* = 8.9, 1.8 *Hz*, 1H), 8.38 (H_{Ar}, *d*, *J* = 8.0 *Hz*, 1H), 8.22 (H_{Ar}, *d*, *J* = 8.9 *Hz*, 1H), 7.61 (NH, *br s*, 1H), 7.55 (H_{Ar}, *dd*, *J* = 8.3, 4.3 *Hz*, 1H), 7.43 – 7.41 ($(\text{-C=}\underline{H_a})_{Furfuryl}$, *dd*, *J* = 1.5, 0.5 *Hz* , 1H), 6.38 ($(\text{-C=}\underline{H_b})_{Furfuryl}$, *t*, *J* = 5 *Hz*, 1H), 6.36 ($(\text{-C=}\underline{H_c})_{Furfuryl}$, *d*, *J* = 3 *Hz*, 1H), 4.63 ($(C\underline{H_2})_{Furfuryl}$, *d*, *J* = 5.9 *Hz*, 2H). ^{13}C NMR (126 *MHz*, $CDCl_3$) δ 186.1 (CO), 161.1 (CO), 152.8, 150.0, 142.7, 141.9, 138.9, 134.9, 131.2, 129.5, 129.5, 127.4, 122.1, 110.6, 108.2, 36.5 $(\underline{C}H_2)_{Furfuryl}$. MS (EI): *m/z* (rel. int, %): 280 (2, [M^+]), 207 (3), 156 (100), 128 (60), 101 (19), 96 (56), 81 (35), 53 (12).

N-(Thiophen-2-ylmethyl)quinoline-6-carboxamide **(2u)**

Yield: 193 mg (72%); yellow brownish crystals, m.p: 143-145 °C, R_f (80 % $CHCl_3$, 20 % EtOAc , 1 % MeOH) 0.30; IR (KBr, ν (cm^{-1})): 3331 *m/br*, 1640 *vs*, 1540 *vs*, 1526 *s*, 1495 *s*, 1419 *m*, 1301 *m*, 858 *m*, 789 *w*, 700 *s*. ^1H NMR (500 *MHz*, $CDCl_3$) δ 8.96 – 8.92 (H_{Ar}, *dd*, *J* = 4.5 , 1.5 *Hz*, 1H), 8.33 (H_{Ar}, *d*, *J* = 1.6 *Hz*, 1H), 8.20 (H_{Ar}, *d*, *J* = 8.2 *Hz*, 1H), 8.13 (H_{Ar}, *d*, *J* = 8.8 *Hz*, 1H), 8.07 (H_{Ar}, *dd*, *J* = 8.8, 1.9 *Hz*, 1H), 7.46 (H_{Ar}, *dd*, *J* = 8.3, 4.3 *Hz*, 1H), 7.26 ($(\text{-C=}\underline{H_a})_{Thiophene}$, *dd*, *J* = 5.1, 0.9 *Hz*, 1H), 7.08 ($(\text{-C=}\underline{H_b})_{Thiophene}$, *s*, *overlapping*, 1H), 7.08 (NH, *s*, *overlapping*, 1H) 6.98 ($(\text{-C=}\underline{H_c})_{Thiophene}$, *dd*, *J* = 5.0, 3.5 *Hz*, 1H), 4.88 ($(C\underline{H_2})_{Thiophene}$, *d*, *J* = 5.5 *Hz*, 2H). ^{13}C NMR (126 *MHz*, $CDCl_3$) δ 166.5 (CO), 151.7, 148.9, 140.6, 137.4, 132.3, 129.6, 127.9, 127.6, 127.4, 127.0, 126.4, 125.5, 121.9, 39.0 $(C\underline{H_2})_{Thiophene}$. MS (EI): *m/z* (rel. int, %): 268 (61, [M^+]), 207 (28), 191(4), 156 (100), 128 (55), 101 (19), 40 (37).

2-Oxo-2-(quinolin-6-yl)-N-(thiophen-2-ylmethyl)acetamide **(3u)**

Yield: 275 mg (93%); pale yellow waxy solid, R_f (90 % $CHCl_3$, 10 % EtOAc , 1 % MeOH) 0.30; IR (KBr, ν (cm^{-1})): 3353 *w*, 2943 *w*, 1684 *w*, 1659 *s*, 1653 *vs*, 1616 *s*, 1558 *m*, 1506 *s*, 1425 *w*, 1175 *m*, 1145 *w*. ^{1}H NMR (500 *MHz*, $CDCl_3$) δ 9.27 (H_{Ar}, *d*, *J* = 1.7 *Hz*, 1H), 9.05 (H_{Ar}, *dd*, *J* = 4.3, 1.6 *Hz*, 1H), 8.44 (H_{Ar}, *dd*, *J* = 8.9, 1.9 *Hz*, 1H), 8.36 (H_{Ar}, *d*, *J* = 7.8 *Hz*, 1H), 8.20 (H_{Ar}, *d*, *J* = 8.9 *Hz*, 1H), 7.68 (NH, *br s*, 1H), 7.53 (H_{Ar}, *dd*, *J* = 8.3, 4.3 *Hz*, 1H), 7.29 ((-C=$\underline{H}_a$)$_{Thiophene}$, *dd*, *J* = 5.0, 1.1 *Hz*, 1H), 7.09 ((-C=$\underline{H}_b$)$_{Thiophene}$, *d*, *J* = 3.1 *Hz*, 1H), 7.00 ((-C=$\underline{H}_c$)$_{Thiophene}$, *dd*, *J* = 5.1, 3.5 *Hz*, 1H), 4.80 ((C$\underline{H}_2$)$_{Thiophene}$, *d*, *J* = 6.0 *Hz*, 2H). ^{13}C NMR (126 *MHz*, $CDCl_3$) δ 186.2 (CO), 161.1 (CO), 153.0, 150.1, 139.3, 138.7, 134.9, 131.1, 129.7, 129.3, 127.4, 127.1, 126.7, 125.7, 122.1, 38.2 ($\underline{C}H_2$)$_{Thiophene}$. MS (EI): *m/z* (rel. int, %): 296 (2, [M^+]), 207 (21), 156 (100), 128 (57), 112 (41), 97 (45), 77 (8), 45 (6).

(3-Hydroxy-8-azabicyclo[3.2.1]octan-8-yl)(quinolin-6-yl)methanone **(2v)**

Yield: 28 mg (10%); brownish light oil, R_f (70 % $CHCl_3$, 30 % EtOAc , 5 % MeOH) 0.20; IR (KBr, ν (cm^{-1})): 3420 *m/br*, 2962 *w*, 2937 *w*, 2913 *w*, 1628 *s*, 1606 *vs*, 1470 *m*, 1437 *m*, 1265 *w*, 1088 *s*, 1046 *s*, 803 *s*. ^{1}H NMR (500 *MHz*, $CDCl_3$) δ 8.94 (H_{Ar}, *d*, *J* = 2.8 *Hz*, 1H), 8.20 (H_{Ar}, *d*, *J* = 8.1 *Hz*, 1H), 8.13 (H_{Ar}, *d*, *J* = 8.6 *Hz*, 1H), 7.94 (H_{Ar}, *s*, 1H), 7.75 (H_{Ar}, *dd*, *J* = 8.6, 1.1 *Hz*, 1H), 7.45 (H_{Ar}, *dd*, *J* = 8.2, 4.2 *Hz*, 1H), 4.80 ((-$\underline{C}H_a$-OH)$_{Nortropine}$, *br s*, 1H), 4.19 ((-C$\underline{H}_b$-)$_{Nortropine}$, *br s*, 1H), 4.05 ((-C$\underline{H}_c$-)$_{Nortropine}$, *br s*, 1H), 3.07 ((OH)$_{Nortropine}$, *br s*, 1H), 2.37 – 2.18 ((-C$\underline{H}_d$-)$_{Nortropine}$ + (-C$\underline{H}_2$-)$_{Nortropine}$, *m*, 3H), 2.08 – 1.96 ((-C$\underline{H}_e$-)$_{Nortropine}$, *m*, 1H), 1.99 – 1.83 ((-C$\underline{H}_f$-)$_{Nortropine}$ + (-C$\underline{H}_2$-)$_{Nortropine}$, *m*, 3H), 1.76 ((-C$\underline{H}_g$-)$_{Nortropine}$, *d*, *J* = 14.2 *Hz*, 1H). ^{13}C NMR (126 *MHz*, $CDCl_3$) δ 167.0 (CO), 151.4, 148.3, 136.8, 134.6, 129.6, 128.0, 127.8, 126.8, 121.9, 64.6 (-$\underline{C}H_a$-OH)$_{Nortropine}$, 56.2 (-CH_a(OH)-$\underline{C}H_{b,c}$-)$_{Nortropine}$, 51.4 (-$\underline{C}H_{d,e}$-CH_a(OH)-$CH_{b,c}$-)$_{Nortropine}$, 40.3 (-$\underline{C}H_f$-)$_{Nortropine}$, 38.6 (-$\underline{C}H_g$-)$_{Nortropine}$, 28.6 (-$\underline{C}H_2$-)$_{Nortropine}$, 27.3 (-$\underline{C}H_2$-)$_{Nortropine}$. MS (EI): *m/z* (rel. int, %): 282 (24, [M^+]), 207 (23), 156 (100), 128 (60), 101 (18), 93 (17), 68 (14), 40 (19).

(3-Hydroxy-8-azabicyclo[3.2.1]octan-8-yl)-2-(quinolin-6-yl)ethane-1,2-dione **(3v)**

Yield: 143 mg (46%); yellow waxy solid, R_f (70 % $CHCl_3$, 30 % EtOAc , 5 % MeOH) 0.30; IR (KBr, ν (cm^{-1})): 2941 *w*, 1687 *m*, 1623 *vs*, 1622 *vs*, 1251 *w*, 1173 *w*, 1087 *w*, 1050 *w*, 825 *w*, 780 *w*. ^{1}H NMR (500 *MHz*, $CDCl_3$) δ 9.02 (H_{Ar}, *dd*, *J* = 4.1, 1.3 *Hz*, 1H), 8.52 (H_{Ar}, *d*, *J* = 1.6 *Hz*, 1H), 8.31 (H_{Ar}, *d*, *J* = 8.1 *Hz*, 1H), 8.26 (H_{Ar}, *dd*, *J* = 8.8, 1.8 *Hz*, 1H), 8.20 (H_{Ar}, *d*, *J* = 8.8 *Hz*, 1H), 7.51 (H_{Ar}, *dd*, *J* = 8.3, 4.3 *Hz*, 1H), 4.85 – 4.80 ((-C$\underline{H}_a$-OH)$_{Nortropine}$, *m*, 1H), 4.21 ((-C$\underline{H}_b$-)$_{Nortropine}$, *t*, *J* = 4.5 *Hz*, 1H), 4.02 – 3.97 ((-C$\underline{H}_c$-)$_{Nortropine}$, *m*, 1H), 2.73 ((OH)$_{Nortropine}$, *br s*, 1H), 2.44 – 2.34 ((-C$\underline{H}_f$-)$_{Nortropine}$, *m*, 1H), 2.32 – 2.17 ((-C$\underline{H}_d$-)$_{Nortropine}$ + (-C$\underline{H}_2$-)$_{Nortropine}$, *m*, 3H), 2.04 – 1.87 ((-C$\underline{H}_e$-)$_{Nortropine}$ + (-C$\underline{H}_2$-)$_{Nortropine}$, *m*, 3H), 1.82 ((-C$\underline{H}_g$-)$_{Nortropine}$, *d*, *J* = 14.3 *Hz*, 1H). ^{13}C NMR (126 *MHz*, $CDCl_3$) δ 190.5 (CO), 161.2 (CO), 153.1, 150.4, 138.2, 132.7, 131.1, 130.3, 127.9, 127.6, 122.3, 64.6 (-$\underline{C}H_a$-OH)$_{Nortropine}$, 54.8 (-CH_a(OH)-$\underline{C}H_{b,c}$-)$_{Nortropine}$, 51.2 (-$\underline{C}H_{d,e}$-CH_a(OH)-$CH_{b,c}$-)$_{Nortropine}$, 40.4 (-$\underline{C}H_f$-)$_{Nortropine}$, 39.1 (-$\underline{C}H_g$-)$_{Nortropine}$, 28.6 (-$\underline{C}H_2$-)$_{Nortropine}$, 27.1 (-

$\underline{C}H_2$-)$_{Nortropine}$. MS (EI): *m/z* (rel. int, %): 310 (2, [M$^+$]), 207 (19), 156 (65), 128 (49), 101 (18), 93 (100), 67 (35), 41 (17).

N-(Prop-2-yn-1-yl)quinoline-6-carboxamide **(2w)**

Yield: 189 mg (90%); pale yellow solid, m.p: 194-195 °C, R_f (90 % $CHCl_3$, 10 % EtOAc , 1 % MeOH) 0.30; IR (KBr, ν (cm^{-1})): 3300 *w/br*, 3196 *w*, 1646 *vs*, 1497 *m*, 1415 *w*, 1311 *w*, 898 *w*, 843 *w*, 785 *w*, 668 *s*. ^{1}H NMR (500 *MHz*, DMSO-d6) δ 9.21 (NH, *t*, *J* = 5.4 *Hz*, 1H), 9.00 (H_{Ar}, *dd*, *J* = 4.2, 1.7 *Hz*, 1H), 8.54 (H_{Ar}, *d*, *J* = 1.8 *Hz*, 1H), 8.49 (H_{Ar}, *d*, *J* = 7.6 *Hz*, 1H), 8.18 (H_{Ar}, *dd*, *J* = 8.8, 1.9 *Hz*, 1H), 8.10 (H_{Ar}, *d*, *J* = 8.8 *Hz*, 1H), 7.62 (H_{Ar}, *dd*, *J* = 8.3, 4.2 *Hz*, 1H), 4.14 ((-$\underline{H}_bC\underline{H}_a$-C≡$CH_c$), *dd*, *J* = 5.5, 2.5 *Hz*, 2H), 3.18 ((-H_bCH_a-C≡$C\underline{H}_c$), *t*, *J* = 2.4 *Hz*, 1H). ^{13}C NMR (126 *MHz*, DMSO-d6) δ 166.0 (CO), 152.6, 149.2, 137.6, 132.1, 129.6, 128.7, 128.1, 127.6, 122.7, 81.7 (-H_bCH_a-$\underline{C}$≡CH_c), 73.5 (-H_bCH_a-C≡$\underline{C}H_c$), 29.2 (-$H_b\underline{C}H_a$-C≡CH_c). MS (EI): *m/z* (rel. int, %): 210 (1, [M$^+$]), 200 (8), 156 (100), 128 (58), 101 (16), 51 (8).

2-Oxo-N-(prop-2-yn-1-yl)-2-(quinolin-6-yl)acetamide **(3w)**

Yield: 100 mg (42%); orange solid, m.p: 129-131 °C, R_f (90 % $CHCl_3$, 10 % EtOAc , 1 % MeOH) 0.50; IR (KBr, ν (cm^{-1})): 3280 *m*, 1684 *m*, 1671 *vs*, 1614 *m*, 1507 *m*, 1419 *m*, 1325 *w*, 1265 *m*, 1173 *m*, 815 *m*, 680 *w*. ^{1}H NMR (500 *MHz*, $CDCl_3$) δ 9.24 (H_{Ar}, *d*, *J* = 1.6 *Hz*, 1H), 9.05 (H_{Ar}, *dd*, *J* = 4.2, 1.6 *Hz*, 1H), 8.44 (H_{Ar}, *dd*, J = 8.9, 1.8 *Hz*, 1H), 8.35 (H_{Ar}, *d*, *J* = 8.1 *Hz*, 1H), 8.20 (H_{Ar}, *d*, *J* = 8.9 *Hz*, 1H), 7.57 (NH, *br s*, 1H), 7.53 (H_{Ar}, *dd*, *J* = 8.3, 4.3 *Hz*, 1H), 4.25 ((-$\underline{H}_bC\underline{H}_a$-C≡$CH_c$), *dd*, *J* = 5.6, 2.5 *Hz*, 2H), 2.34 ((-H_bCH_a-C≡$C\underline{H}_c$), *t*, *J* = 2.5 *Hz*, 1H). ^{13}C NMR (126 *MHz*, $CDCl_3$) δ 185.7 (CO), 161.1 (CO), 153.1, 150.2, 138.6, 134.9, 130.9, 129.8, 129.3, 127.4, 122.1, 78.6 (-H_bCH_a-$\underline{C}$≡CH_c), 72.5 (-H_bCH_a-C≡$\underline{C}H_c$), 29.3 (-$H_b\underline{C}H_a$-C≡CH_c). MS (EI): *m/z* (rel. int, %): 238 (2, [M$^+$]), 156 (100), 128 (45), 101 (15), 75 (8), 51 (4).

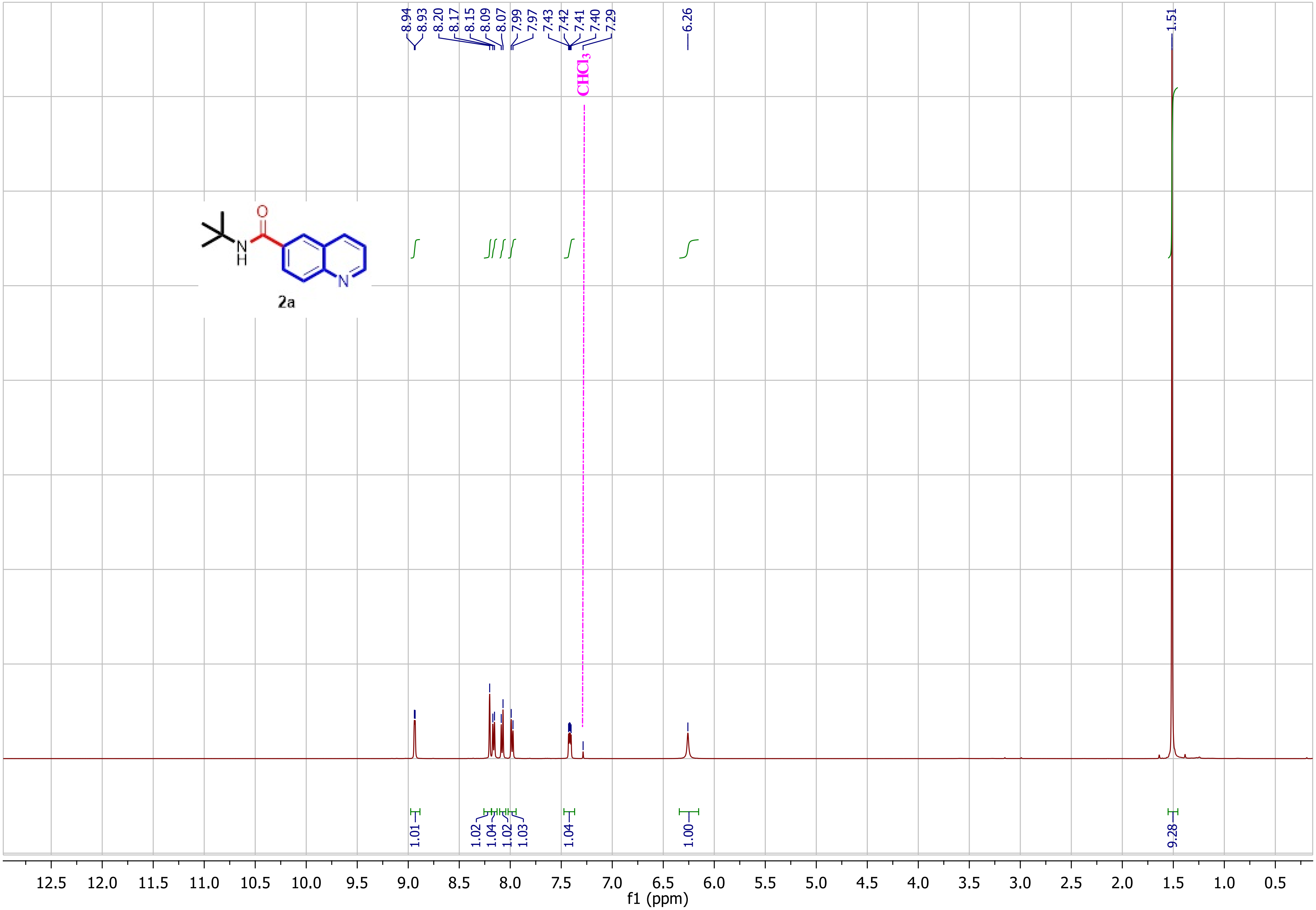

8.94
8.93
8.20
8.17
8.15
8.09
8.07
7.99
7.97
7.43
7.42
7.41
7.40
7.29
6.26
1.51
CHCl3
O
N
H
N
2a
1.01
1.02
1.04
1.02
1.03
1.04
1.00
9.28
12.5 12.0 11.5 11.0 10.5 10.0 9.5 9.0 8.5 8.0 7.5 7.0 6.5 6.0 5.5 5.0 4.5 4.0 3.5 3.0 2.5 2.0 1.5 1.0 0.5
f1 (ppm)

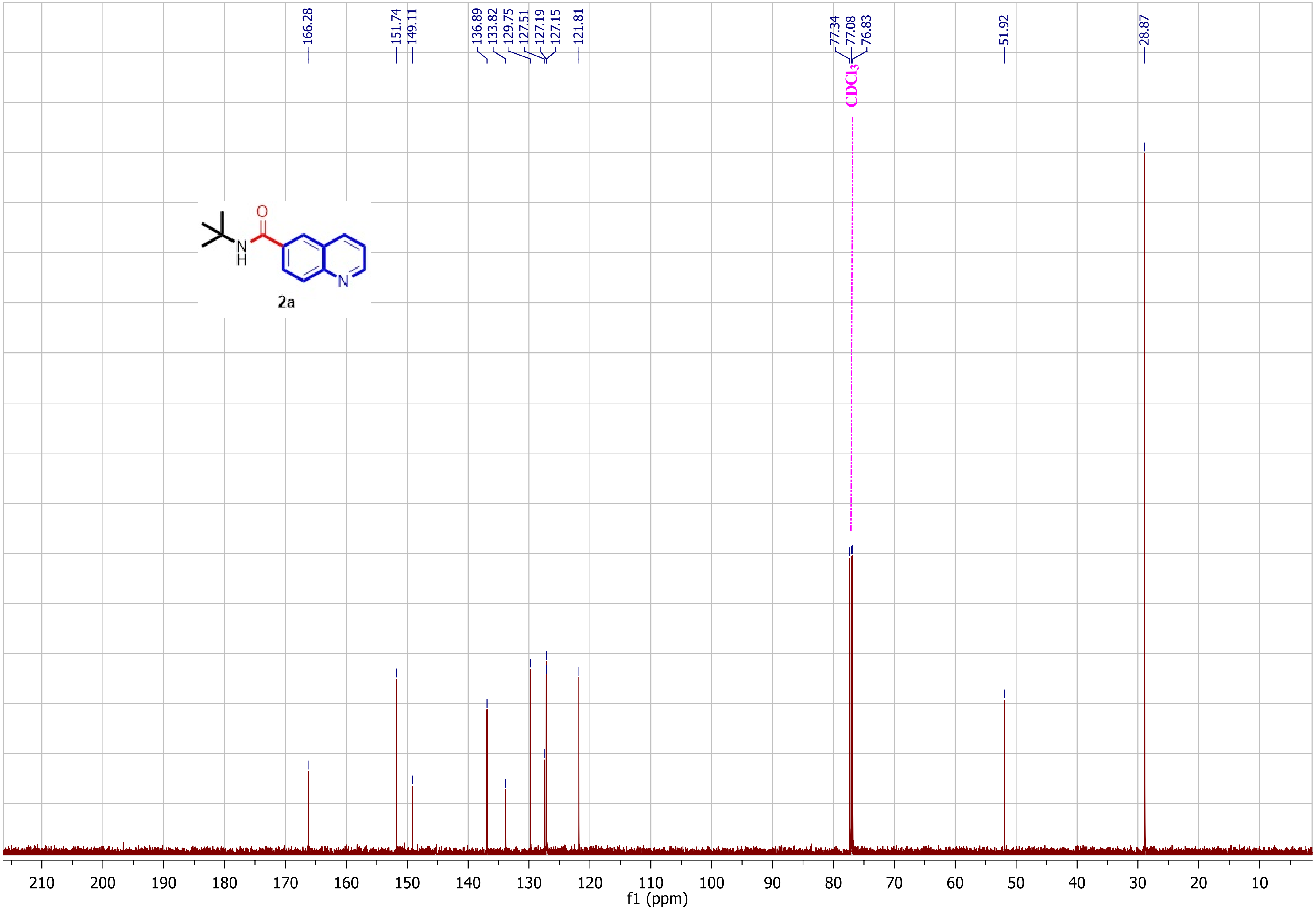
166.28
151.74
149.11
136.89
133.82
129.75
127.51
127.19
127.15
121.81
77.34
77.08
76.83
CDCl3
51.92
28.87
O
N
H
N
2a
210 200 190 180 170 160 150 140 130 120 110 100 90 80 70 60 50 40 30 20 10
f1 (ppm)

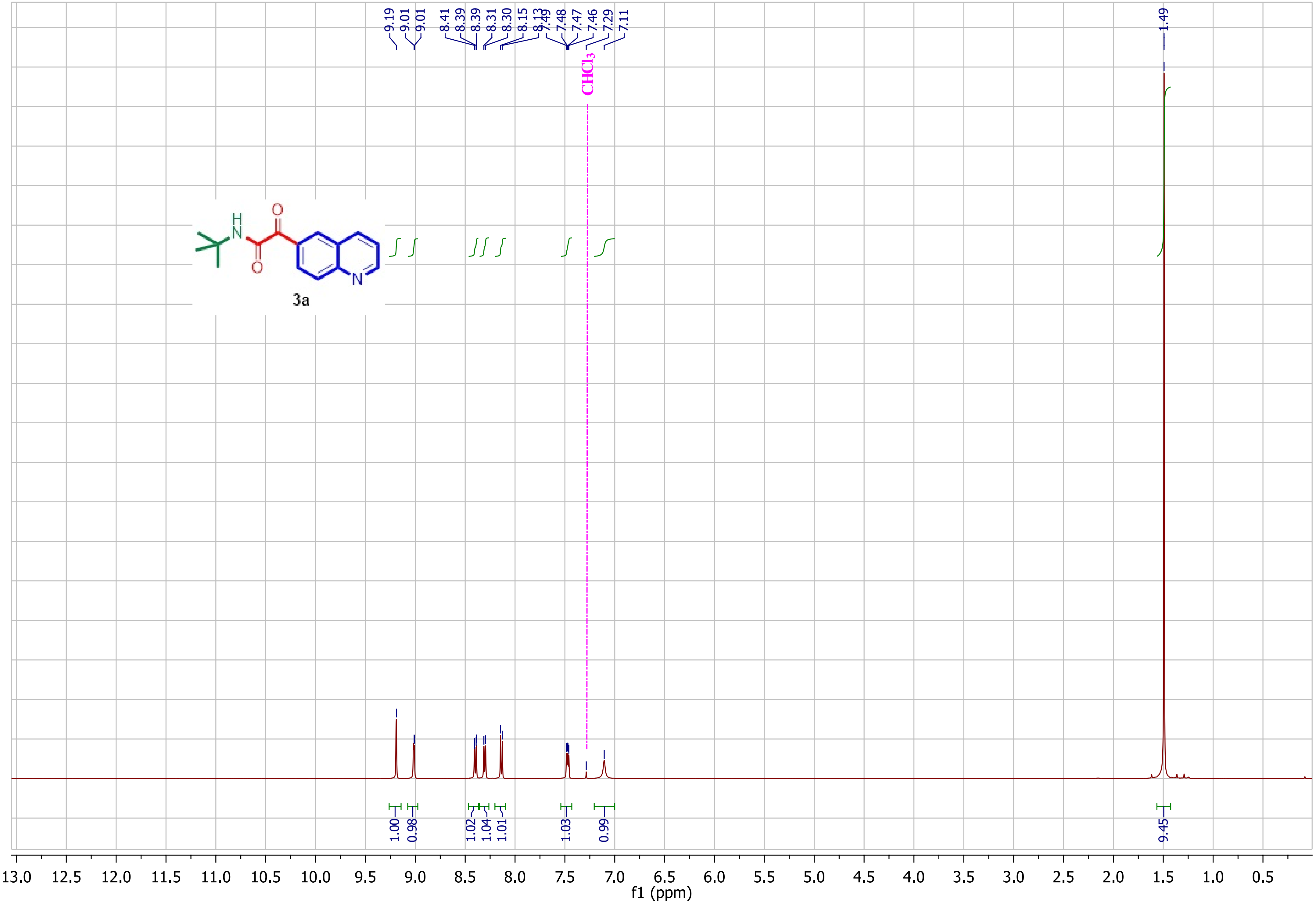

9.19
9.01
9.01
8.41
8.39
8.39
8.31
8.30
8.15
8.13
7.49
7.48
7.47
7.46
7.29
7.11
1.49
CHCl3
3a
1.00
0.98
1.02
1.04
1.01
1.03
0.99
9.45
f1 (ppm)

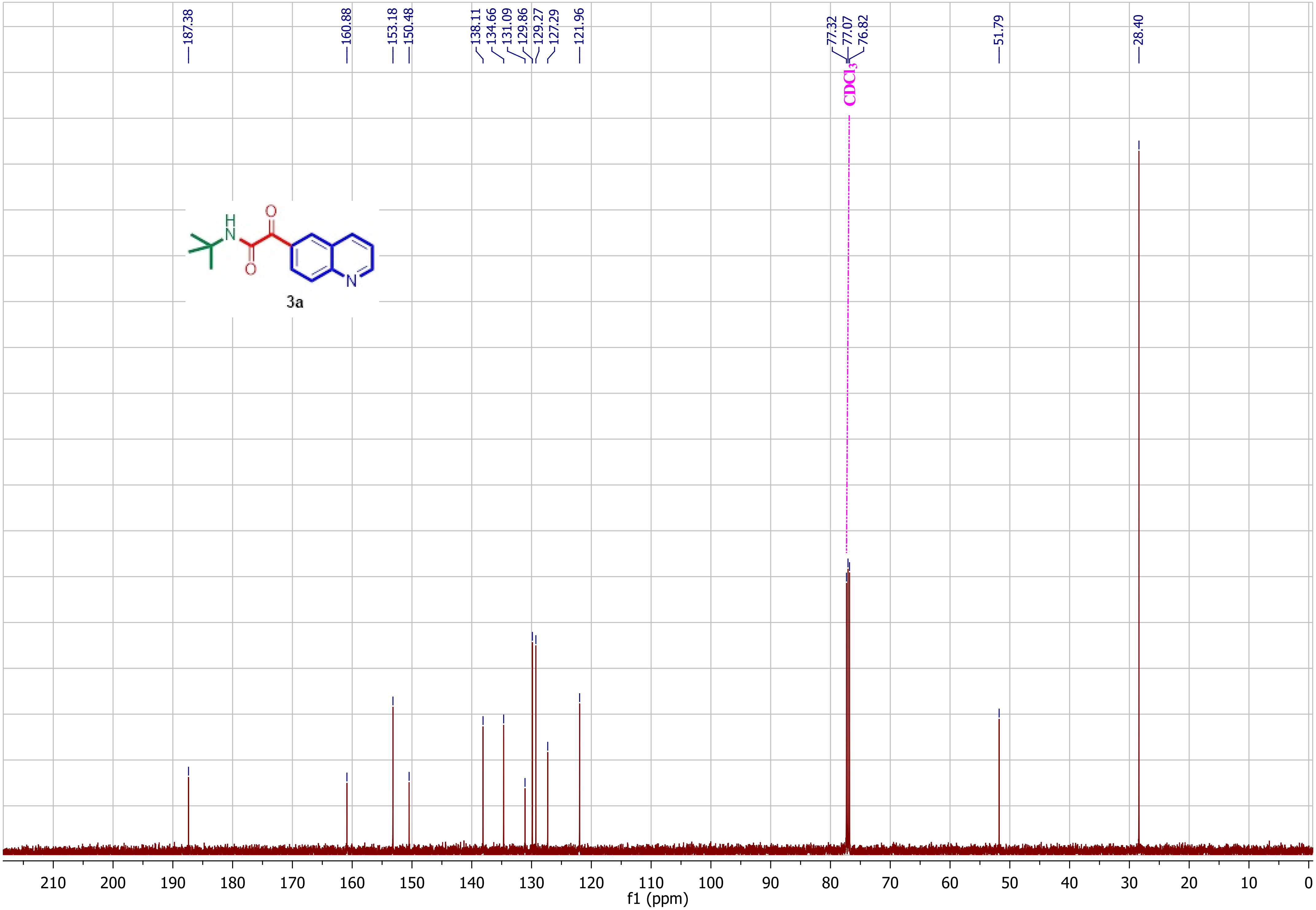
187.38
160.88
153.18
150.48
138.11
134.66
131.09
129.86
129.27
127.29
121.96
77.32
77.07
76.82
51.79
28.40
CDCl3
H
N
O
O
N
3a
210 200 190 180 170 160 150 140 130 120 110 100 90 80 70 60 50 40 30 20 10 0
f1 (ppm)

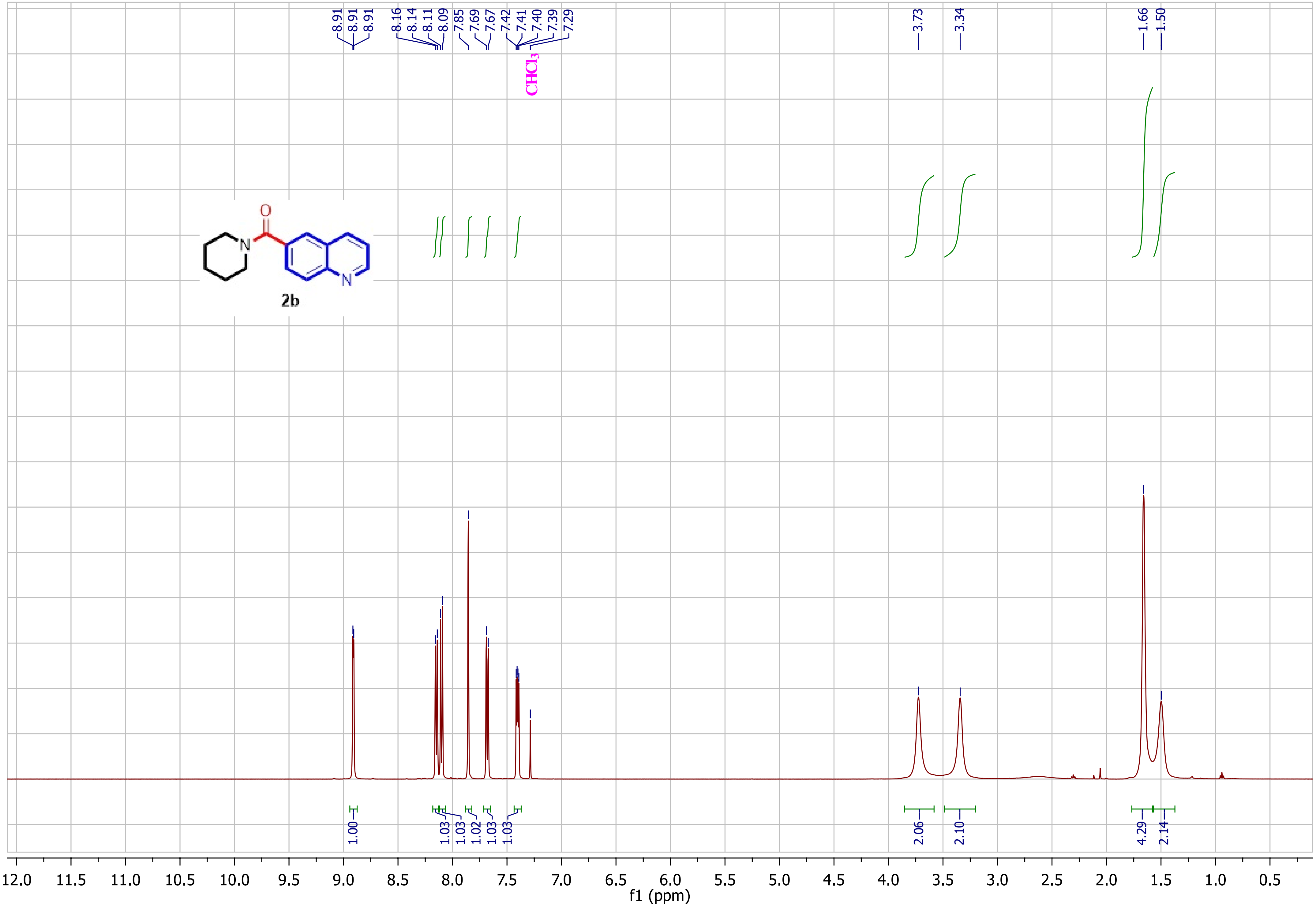
8.91
8.91
8.91
8.16
8.14
8.11
8.09
7.85
7.69
7.67
7.42
7.41
7.40
7.39
7.29
CHCl3
3.73
3.34
1.66
1.50
2b
1.00
1.03
1.03
1.02
1.03
1.03
2.06
2.10
4.29
2.14
f1 (ppm)

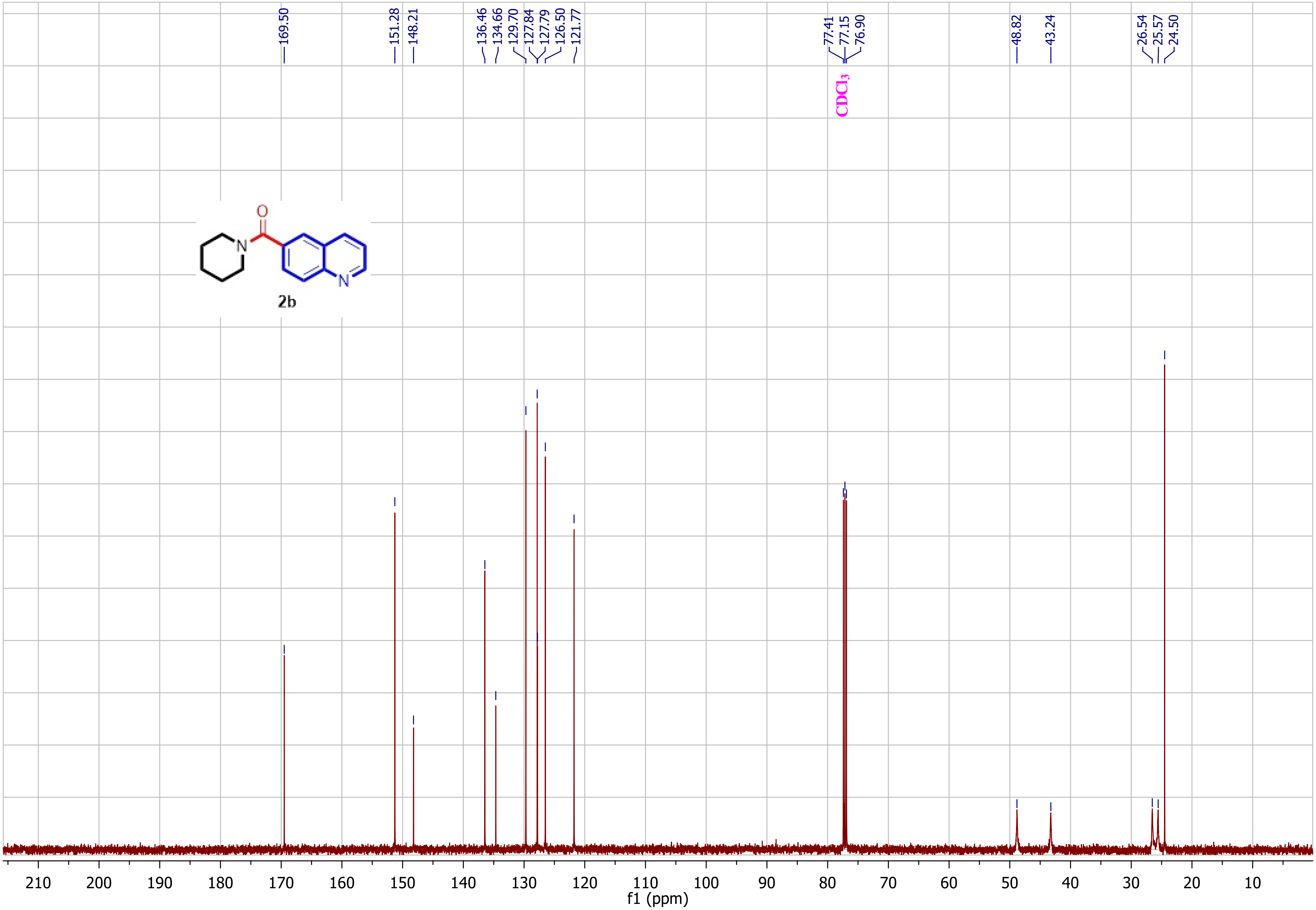
169.50
151.28
148.21
136.46
134.66
129.70
127.84
127.79
126.50
121.77
77.41
77.15
76.90
CDCl3
48.82
43.24
26.54
25.57
24.50
O
N
N
2b
210 200 190 180 170 160 150 140 130 120 110 100 90 80 70 60 50 40 30 20 10
f1 (ppm)

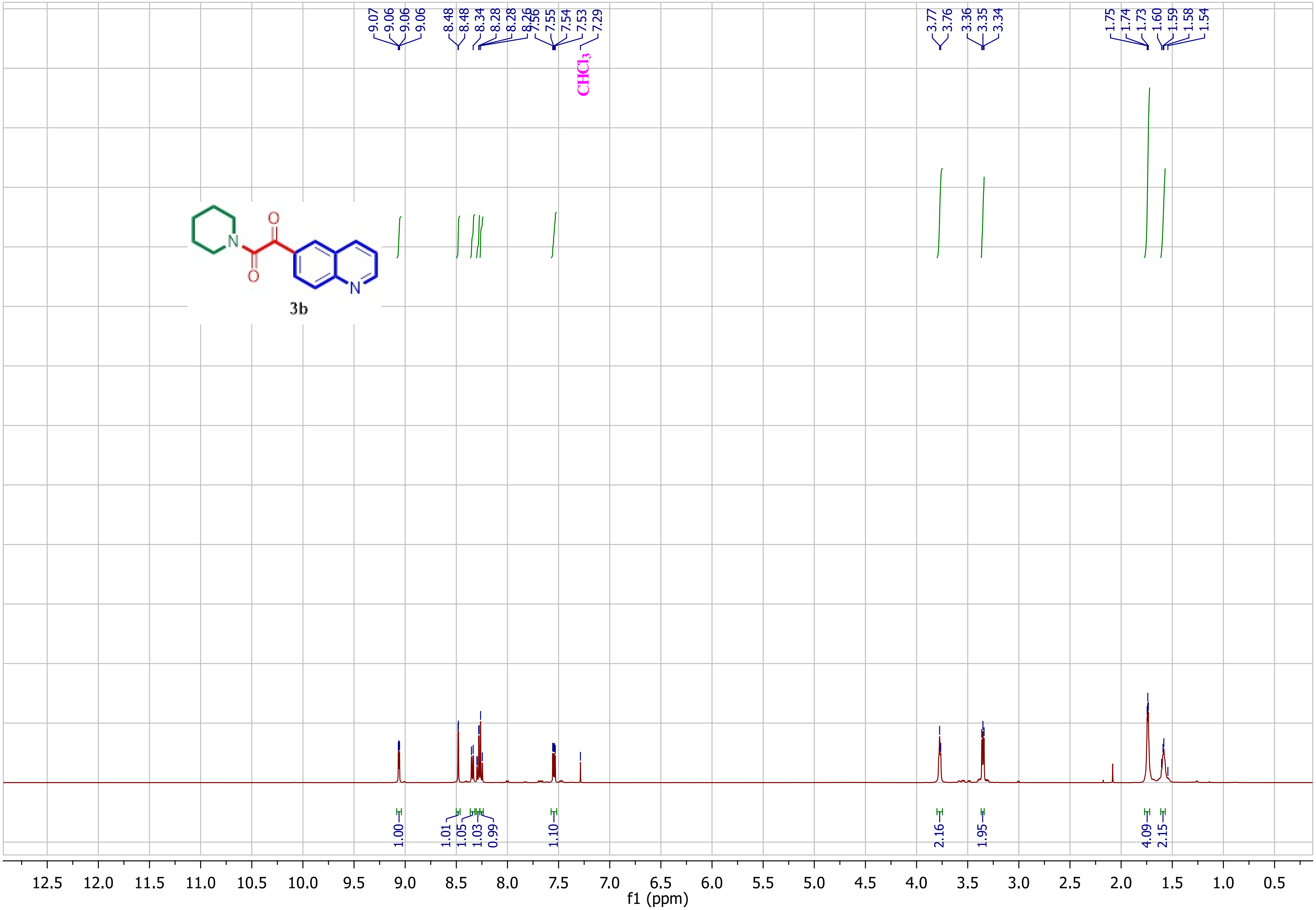
9.07
9.06
9.06
9.06
8.48
8.48
8.34
8.28
8.28
8.26
7.56
7.55
7.54
7.53
7.29
CHCl3
3.77
3.76
3.36
3.35
3.34
1.75
1.74
1.73
1.60
1.59
1.58
1.54
3b
1.00
1.01
1.05
1.03
0.99
1.10
2.16
1.95
4.09
2.15
12.5 12.0 11.5 11.0 10.5 10.0 9.5 9.0 8.5 8.0 7.5 7.0 6.5 6.0 5.5 5.0 4.5 4.0 3.5 3.0 2.5 2.0 1.5 1.0 0.5
f1 (ppm)

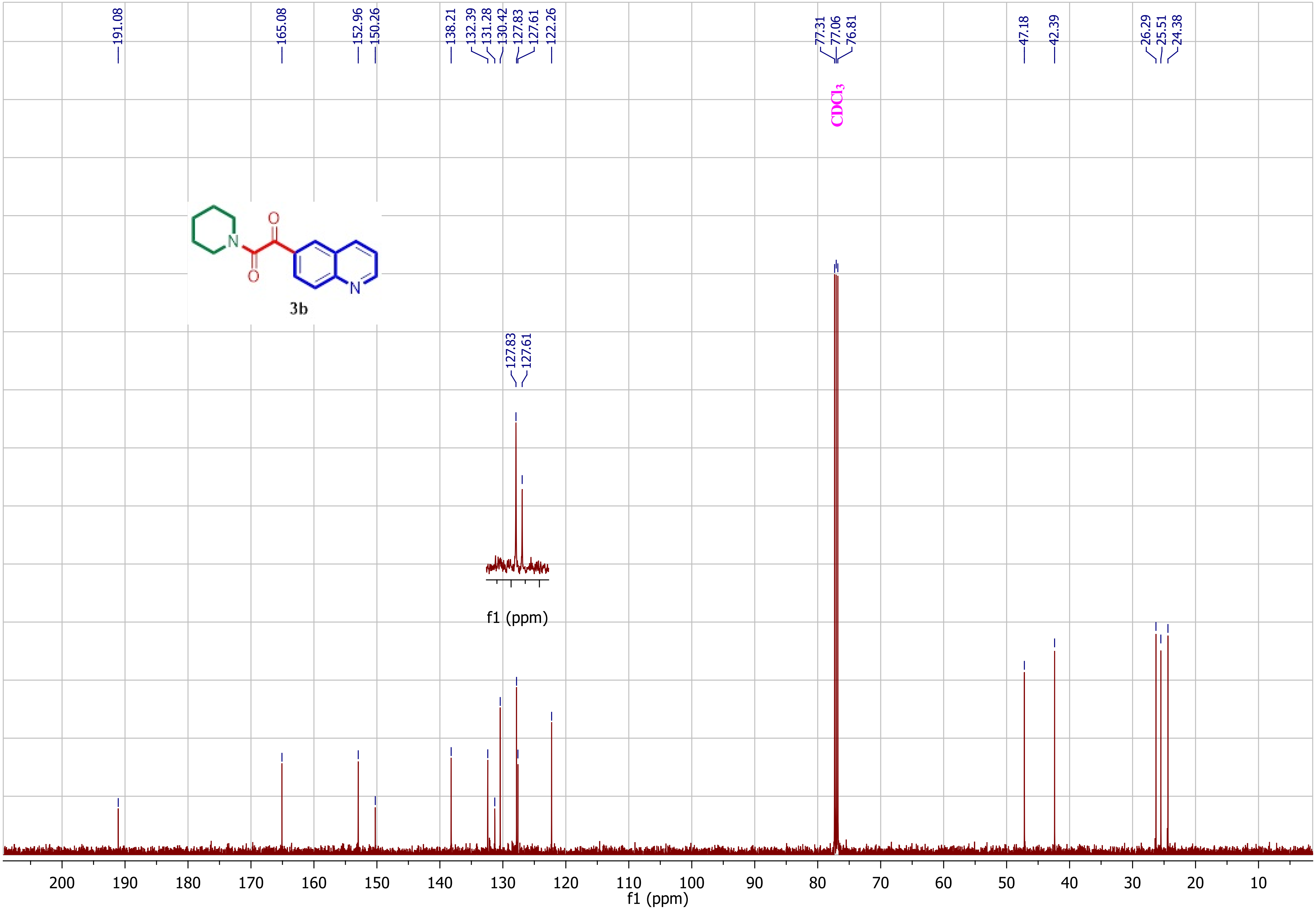
191.08
165.08
152.96
150.26
138.21
132.39
131.28
130.42
127.83
127.61
122.26
77.31
77.06
76.81
47.18
42.39
26.29
25.51
24.38
CDCl3
N
O
O
N
3b
127.83
127.61
f1 (ppm)
200 190 180 170 160 150 140 130 120 110 100 90 80 70 60 50 40 30 20 10
f1 (ppm)

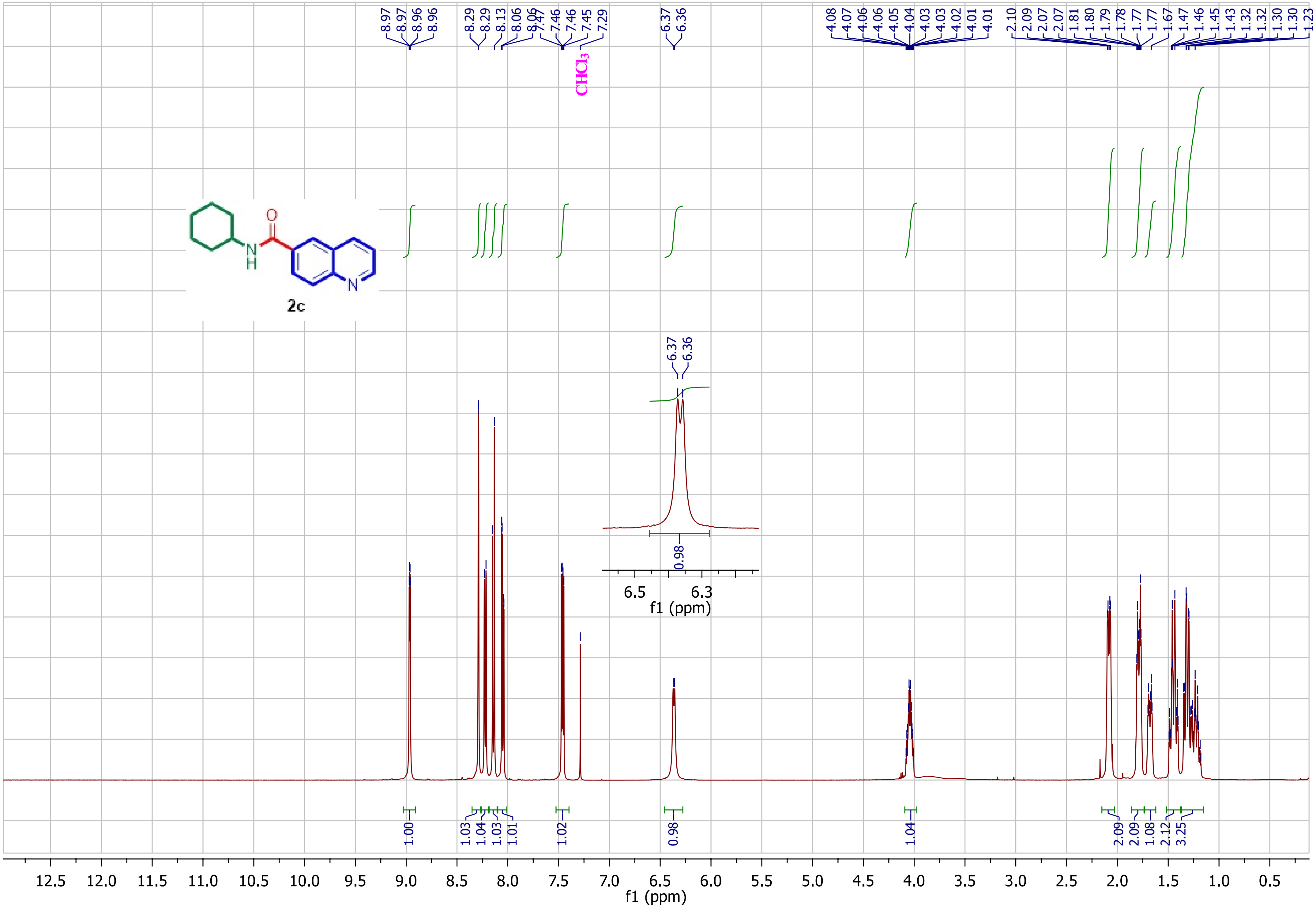
8.97 8.97 8.96 8.96 8.29 8.29 8.13 8.06 8.05 7.47 7.46 7.46 7.45 7.29
6.37 6.36
4.08 4.07 4.06 4.06 4.05 4.04 4.03 4.03 4.02 4.01 4.01
2.10 2.09 2.07 2.07 1.81 1.80 1.79 1.78 1.77 1.77 1.67 1.47 1.46 1.45 1.43 1.32 1.32 1.30 1.30 1.23
CHCl3
2c
6.37 6.36
0.98
6.5 6.3
f1 (ppm)
1.00 1.03 1.04 1.03 1.01 1.02 0.98 1.04 2.09 2.09 1.08 2.12 3.25
12.5 12.0 11.5 11.0 10.5 10.0 9.5 9.0 8.5 8.0 7.5 7.0 6.5 6.0 5.5 5.0 4.5 4.0 3.5 3.0 2.5 2.0 1.5 1.0 0.5
f1 (ppm)

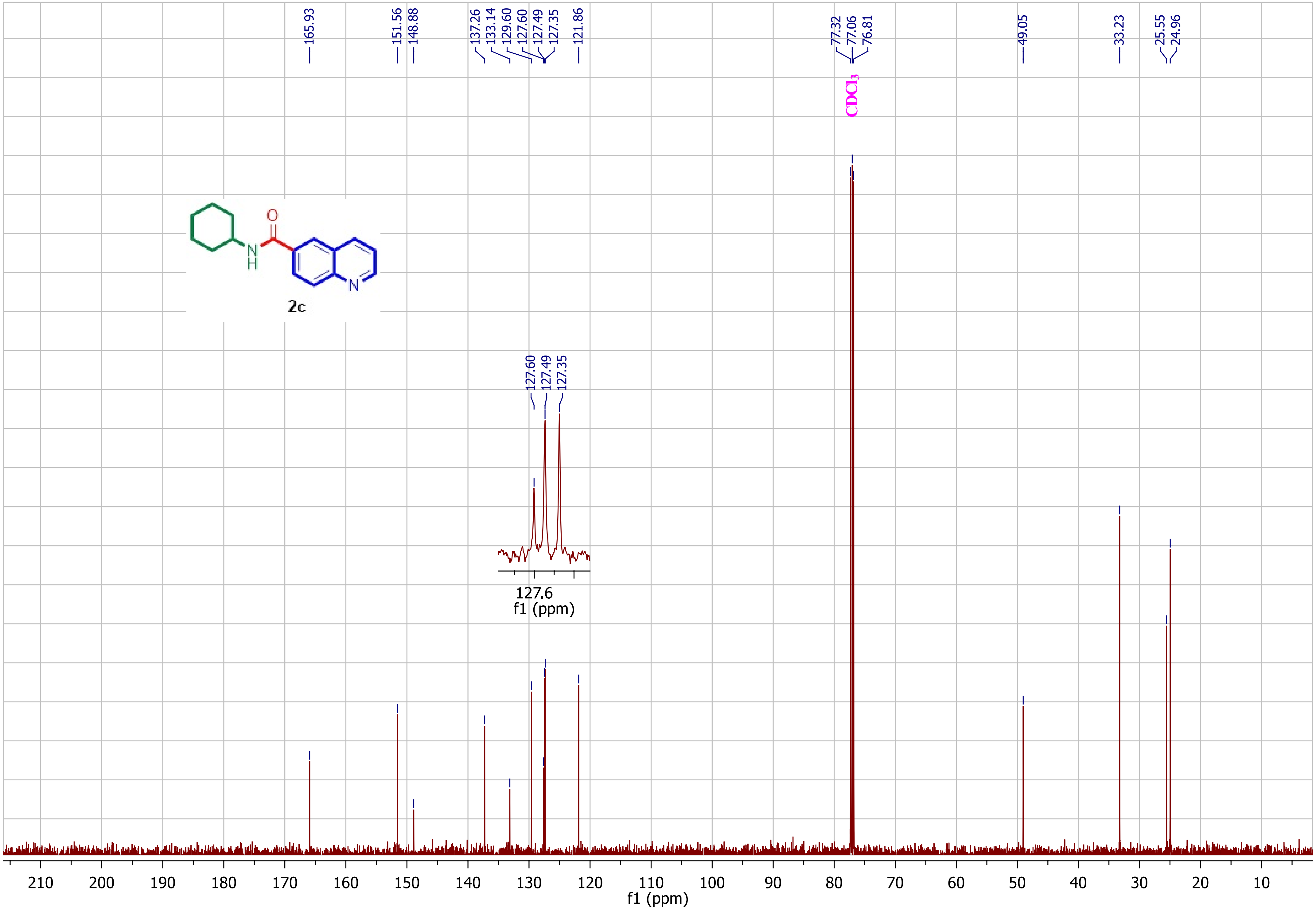

165.93
151.56
148.88
137.26
133.14
129.60
127.60
127.49
127.35
121.86
77.32
77.06
76.81
49.05
33.23
25.55
24.96
CDCl3
O
N
H
N
2c
127.60
127.49
127.35
127.6
f1 (ppm)
210
200
190
180
170
160
150
140
130
120
110
100
90
80
70
60
50
40
30
20
10
f1 (ppm)

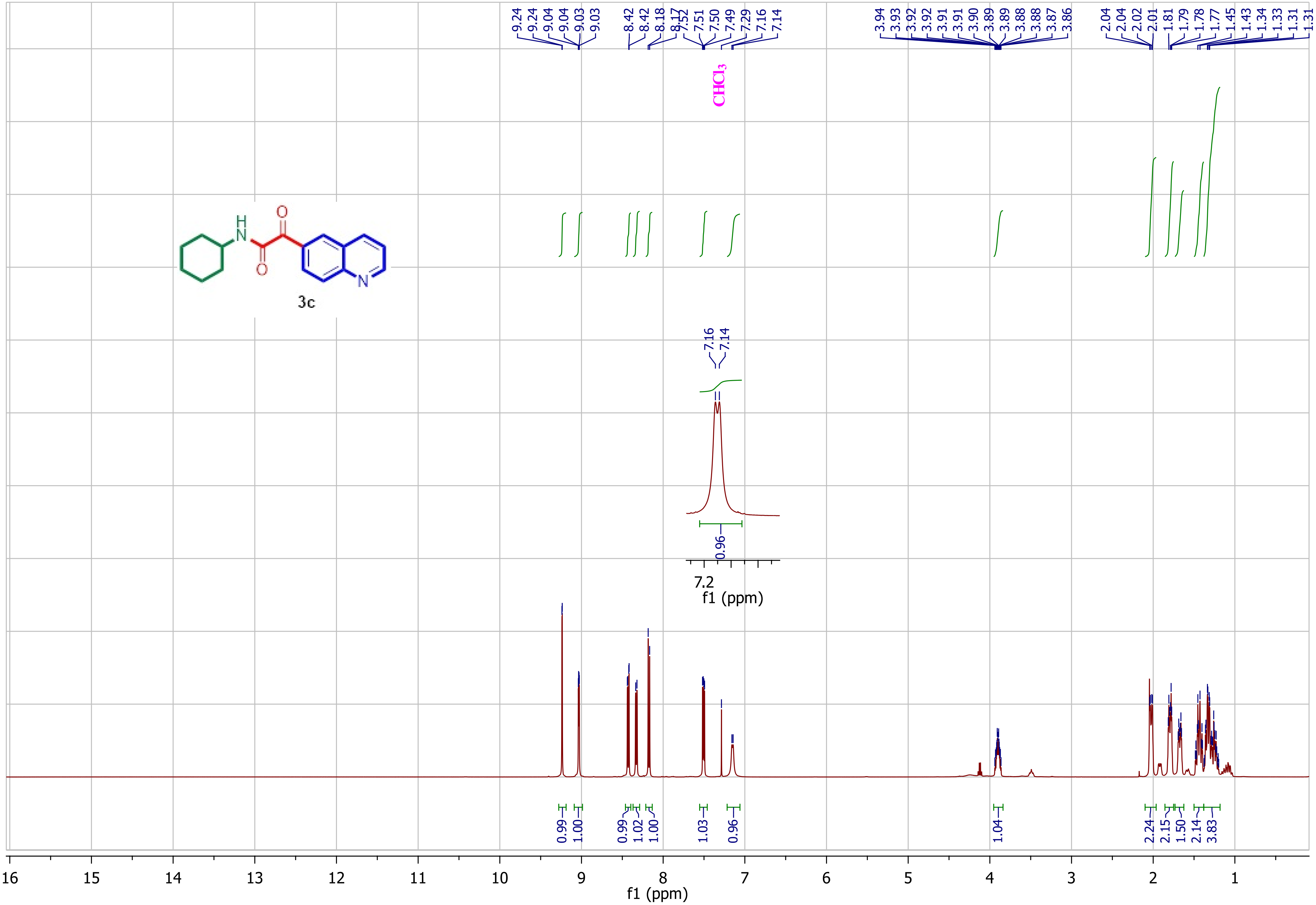

9.24 9.24 9.04 9.04 9.03 9.03 8.42 8.42 8.18 8.17 7.52 7.51 7.50 7.49 7.29 7.16 7.14
3.94 3.93 3.92 3.92 3.91 3.91 3.90 3.89 3.89 3.88 3.88 3.87 3.86
2.04 2.04 2.02 2.01 1.81 1.79 1.78 1.77 1.45 1.43 1.34 1.33 1.31 1.31
CHCl3
H
N
O
O
N
3c
7.16
7.14
0.96
7.2
f1 (ppm)
0.99 1.00 0.99 1.02 1.00 1.03 0.96 1.04 2.24 2.15 1.50 2.14 3.83
16 15 14 13 12 11 10 9 8 7 6 5 4 3 2 1
f1 (ppm)

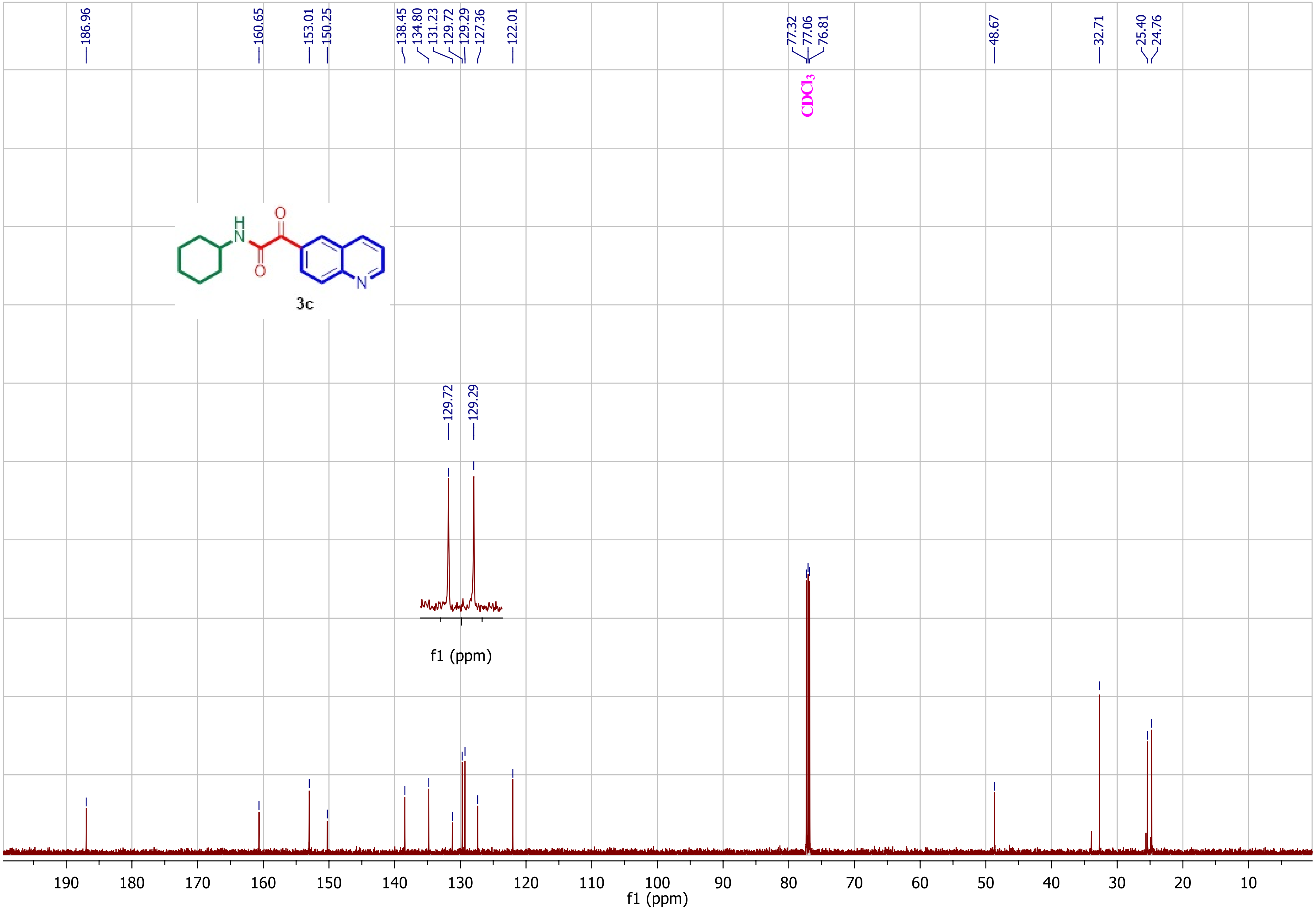
186.96
160.65
153.01
150.25
138.45
134.80
131.23
129.72
129.29
127.36
122.01
77.32
77.06
76.81
CDCl3
48.67
32.71
25.40
24.76
H
N
O
O
N
3c
129.72
129.29
f1 (ppm)
190 180 170 160 150 140 130 120 110 100 90 80 70 60 50 40 30 20 10
f1 (ppm)

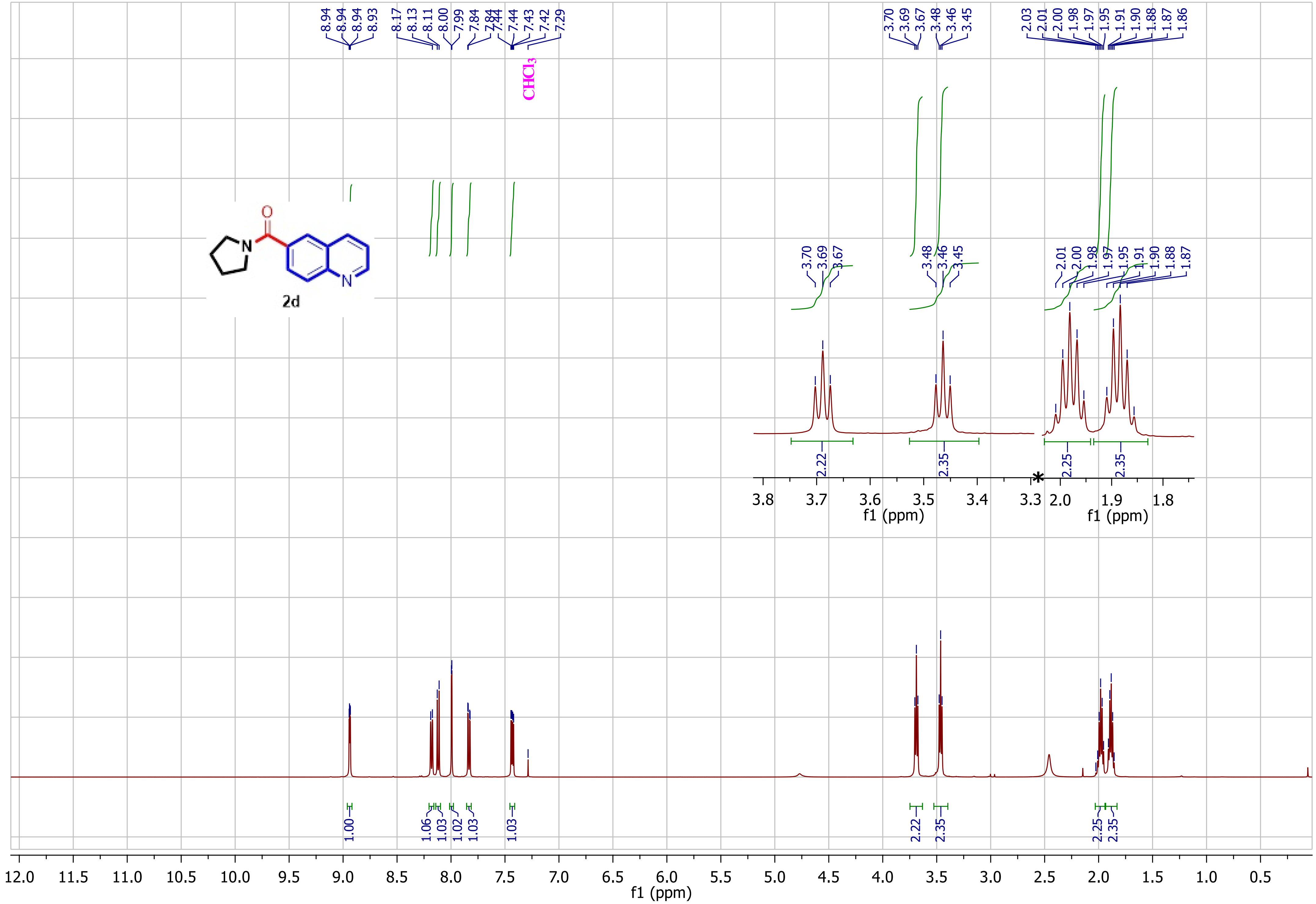
8.94
8.94
8.94
8.93
8.17
8.13
8.11
8.00
7.99
7.84
7.84
7.44
7.44
7.43
7.42
7.29
3.70
3.69
3.67
3.48
3.46
3.45
2.03
2.01
2.00
1.98
1.97
1.95
1.91
1.90
1.88
1.87
1.86
CHCl3
2d
1.00
1.06
1.03
1.02
1.03
1.03
2.22
2.35
2.25
2.35
f1 (ppm)

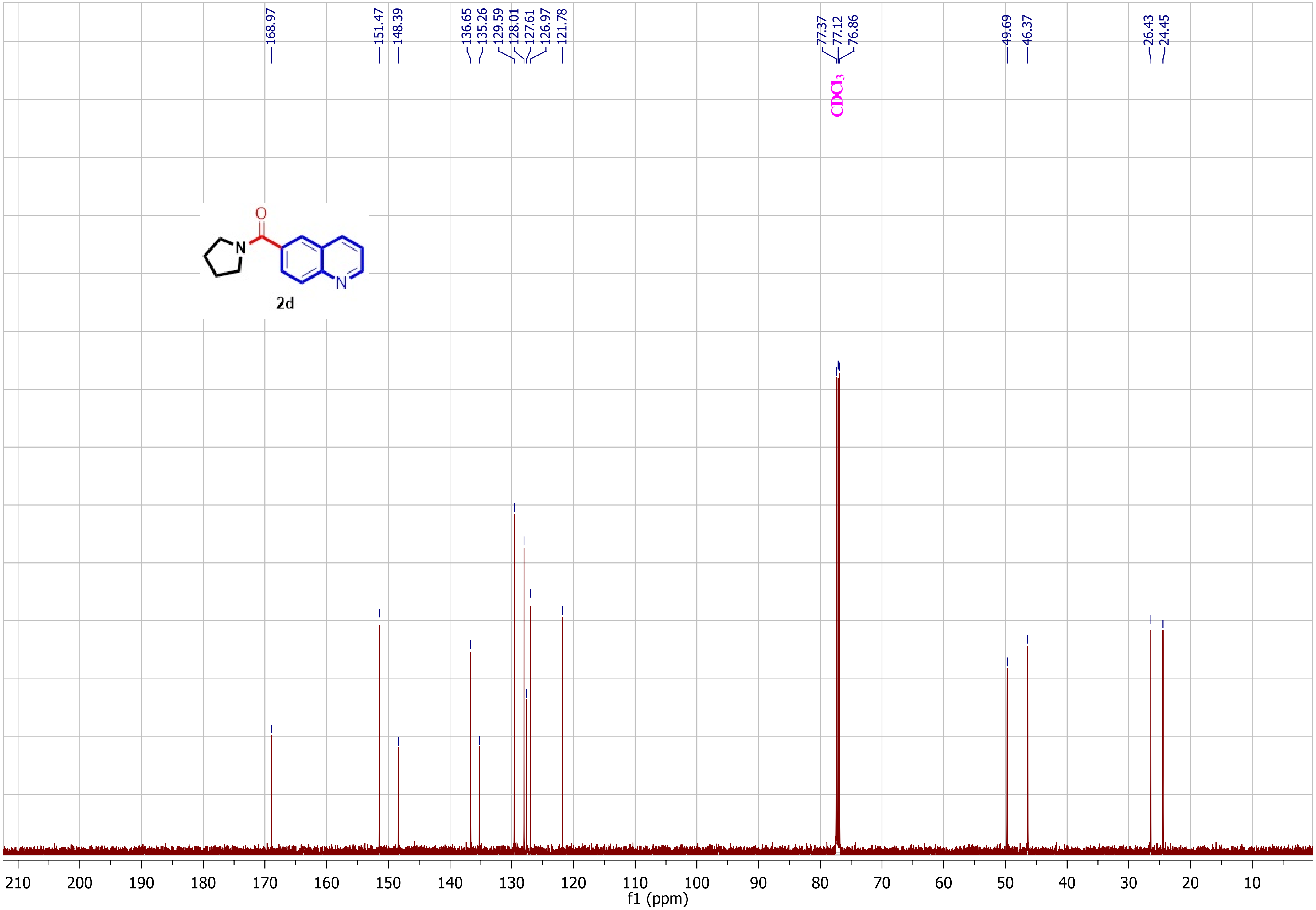

168.97
151.47
148.39
136.65
135.26
129.59
128.01
127.61
126.97
121.78
77.37
77.12
76.86
CDCl3
49.69
46.37
26.43
24.45
O
N
N
2d
210 200 190 180 170 160 150 140 130 120 110 100 90 80 70 60 50 40 30 20 10
f1 (ppm)

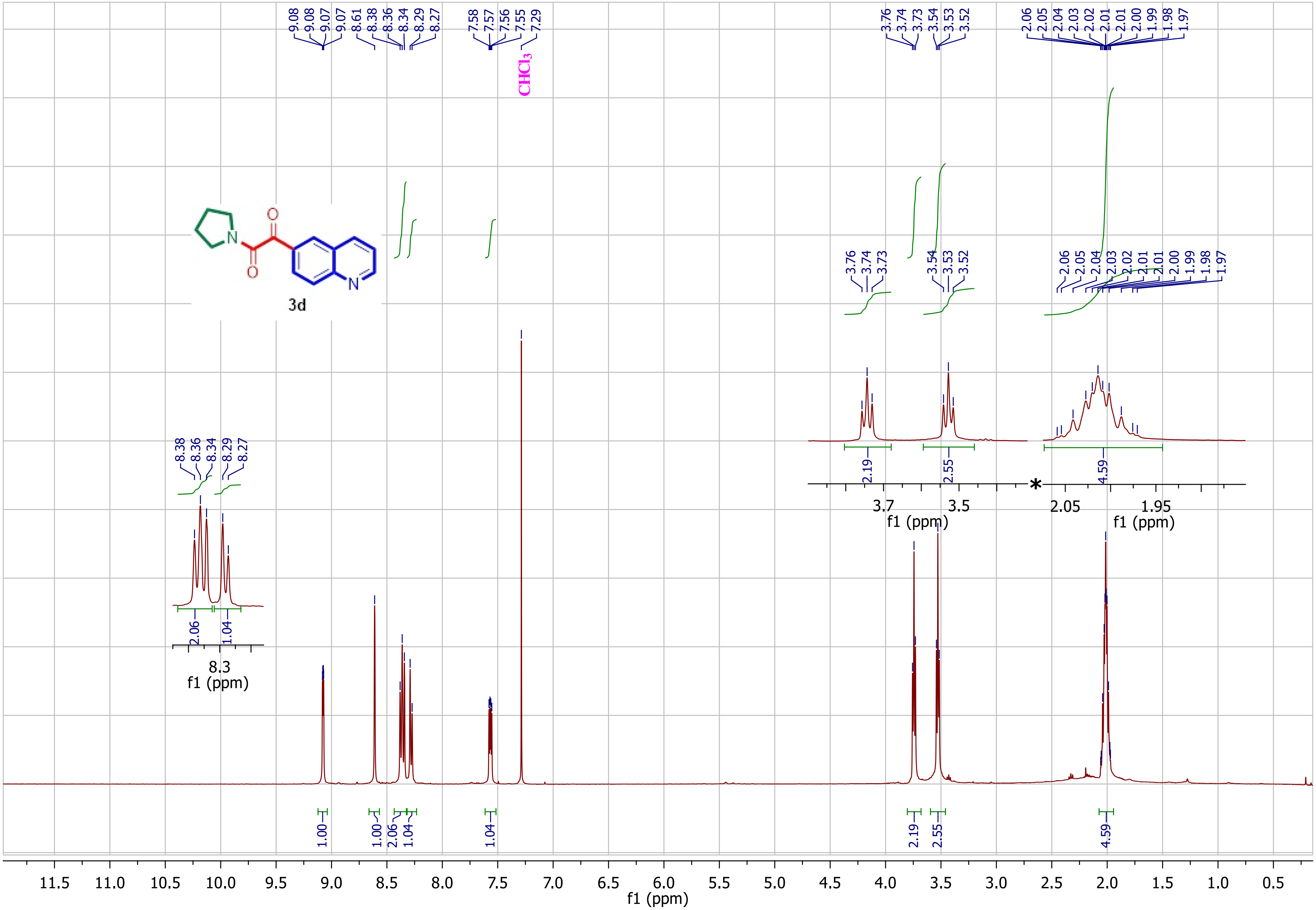

9.08 9.08 9.07 9.07 8.61 8.38 8.36 8.34 8.29 8.27
7.58 7.57 7.56 7.55 7.29
CHCl3
3.76 3.74 3.73 3.54 3.53 3.52
2.06 2.05 2.04 2.03 2.02 2.01 2.01 2.00 1.99 1.98 1.97
3d
2.06 1.04
8.3
f1 (ppm)
2.19 2.55 4.59
3.7 3.5
2.05 1.95
f1 (ppm)
1.00 1.00 2.06 1.04 1.04 2.19 2.55 4.59
11.5 11.0 10.5 10.0 9.5 9.0 8.5 8.0 7.5 7.0 6.5 6.0 5.5 5.0 4.5 4.0 3.5 3.0 2.5 2.0 1.5 1.0 0.5
f1 (ppm)

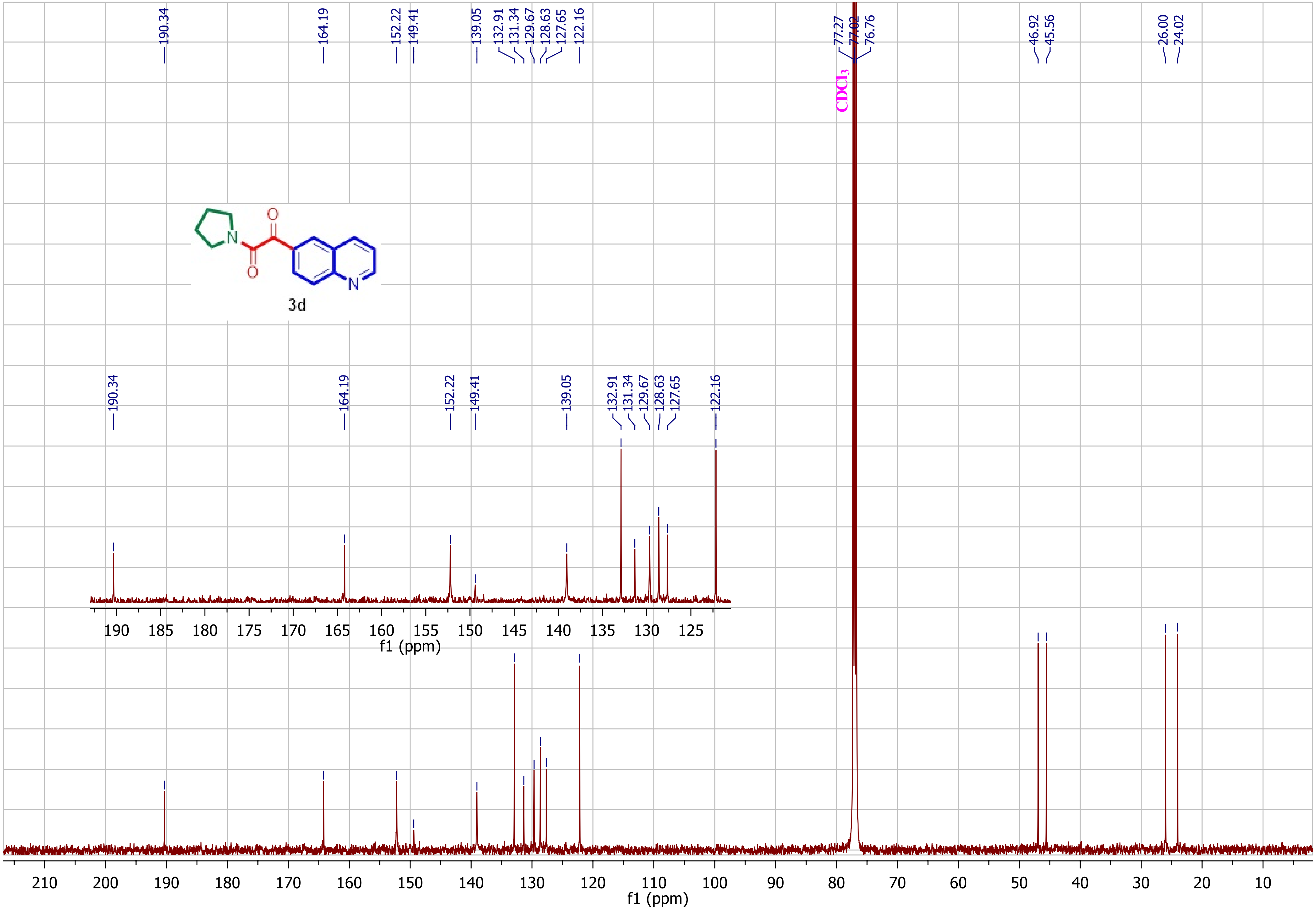
190.34
164.19
152.22
149.41
139.05
132.91
131.34
129.67
128.63
127.65
122.16
77.27
77.02
76.76
46.92
45.56
26.00
24.02
CDCl3
3d
f1 (ppm)

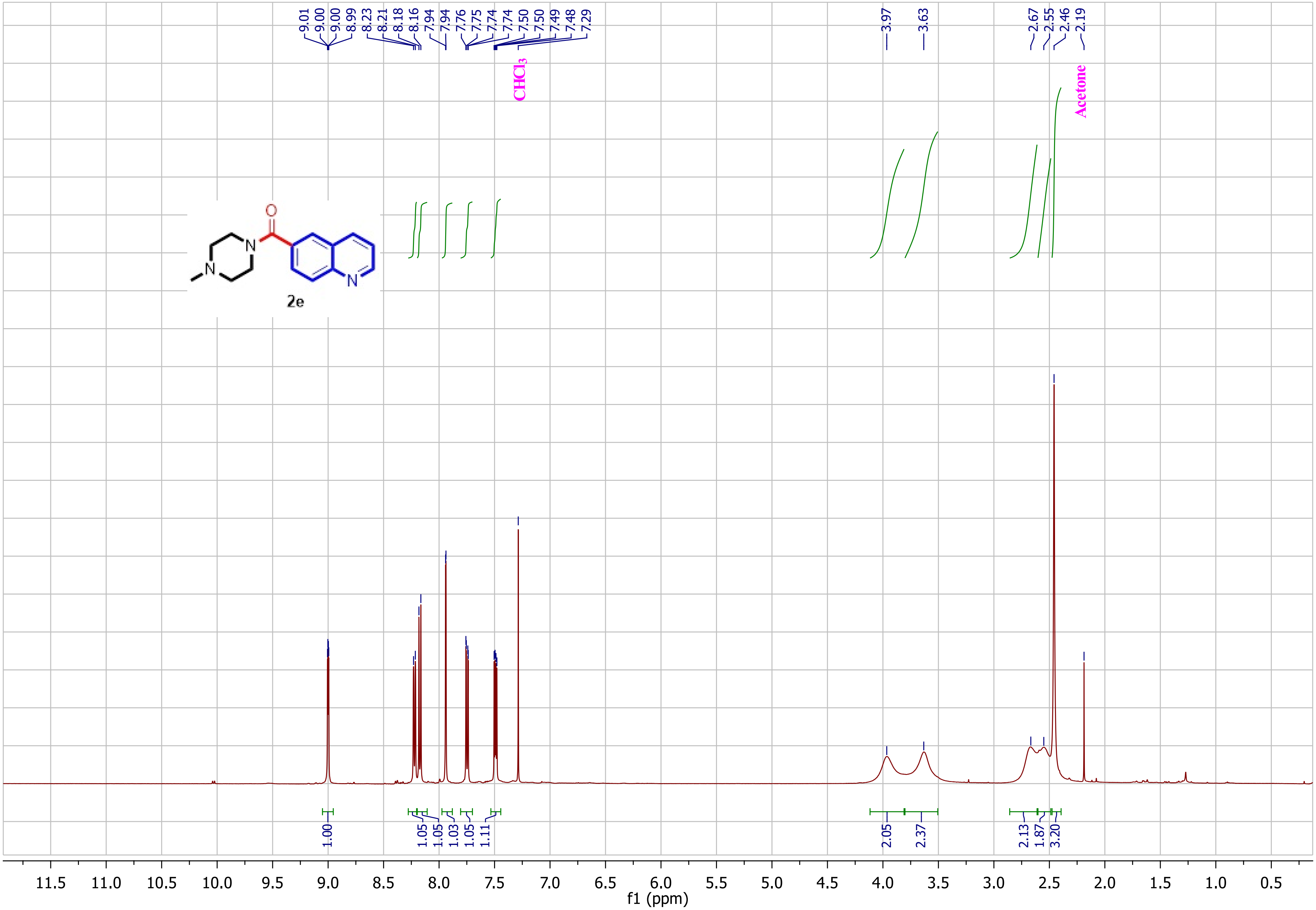
9.01
9.00
9.00
8.99
8.23
8.21
8.18
8.16
7.94
7.94
7.76
7.75
7.74
7.74
7.50
7.50
7.49
7.48
7.29
3.97
3.63
2.67
2.55
2.46
2.19
CHCl3
Acetone
O
N
N
N
2e
1.00
1.05
1.05
1.03
1.05
1.11
2.05
2.37
2.13
1.87
3.20
11.5
11.0
10.5
10.0
9.5
9.0
8.5
8.0
7.5
7.0
6.5
6.0
5.5
5.0
4.5
4.0
3.5
3.0
2.5
2.0
1.5
1.0
0.5
f1 (ppm)

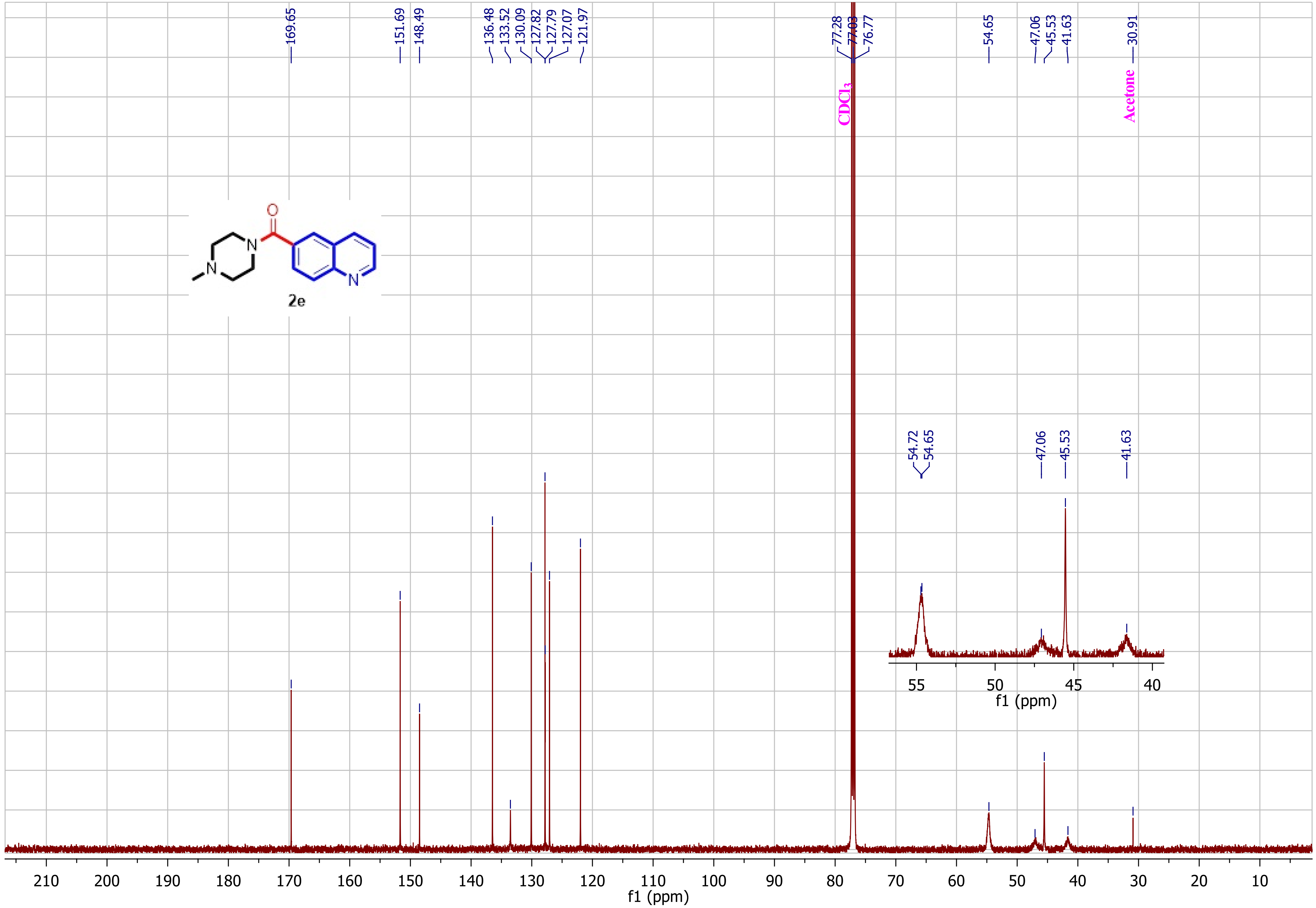

169.65
151.69
148.49
136.48
133.52
130.09
127.82
127.79
127.07
121.97
77.28
77.03
76.77
54.65
47.06
45.53
41.63
30.91
CDCl3
Acetone
O
N
N
N
2e
54.72
54.65
47.06
45.53
41.63
55
50
45
40
f1 (ppm)
210
200
190
180
170
160
150
140
130
120
110
100
90
80
70
60
50
40
30
20
10
f1 (ppm)

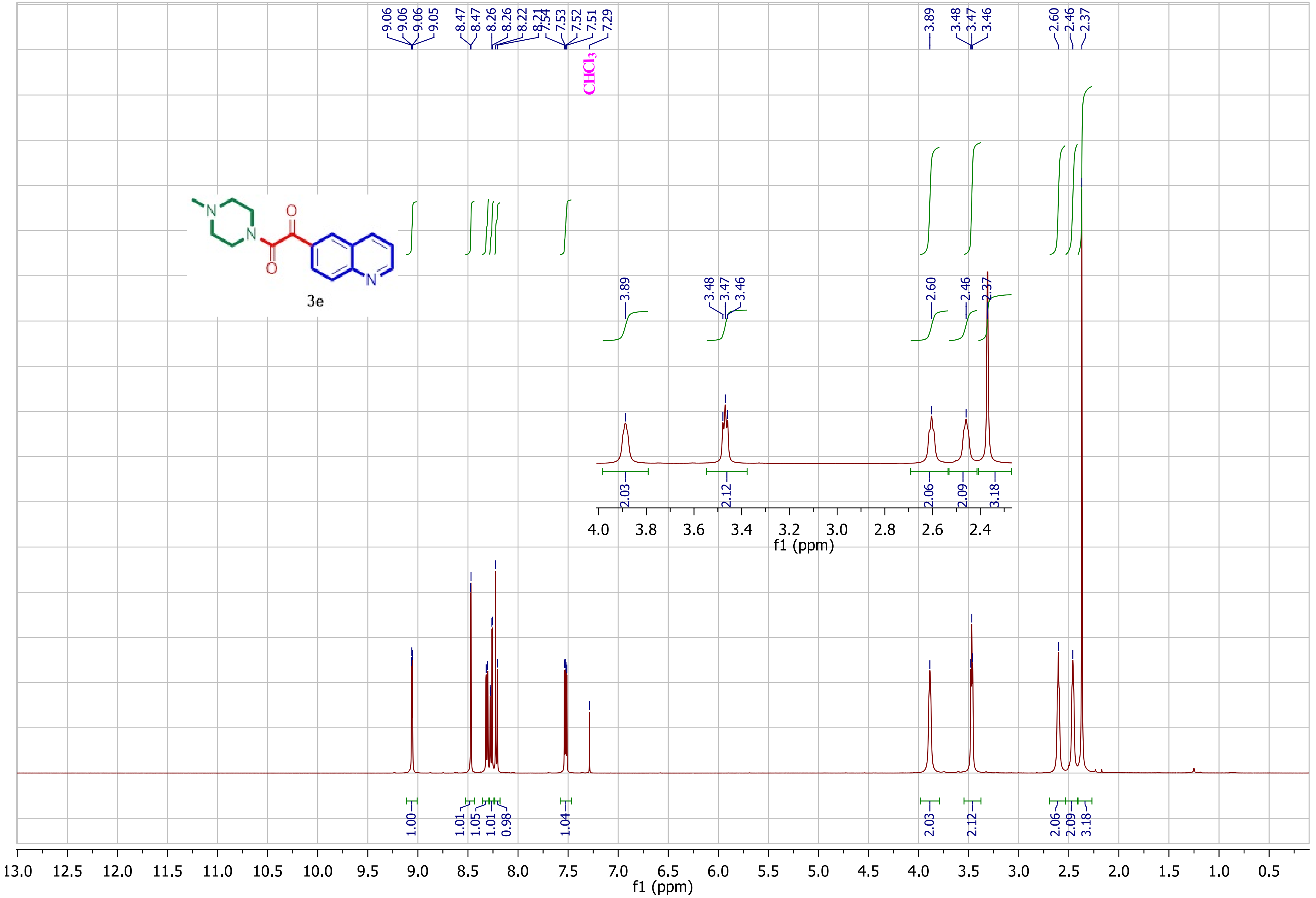

9.06
9.06
9.06
9.05
8.47
8.47
8.26
8.26
8.22
8.21
7.54
7.53
7.52
7.51
7.29
CHCl3
3.89
3.48
3.47
3.46
2.60
2.46
2.37
O
N
N
O
N
3e
1.00
1.01
1.05
1.01
0.98
1.04
2.03
2.12
2.06
2.09
3.18
13.0
12.5
12.0
11.5
11.0
10.5
10.0
9.5
9.0
8.5
8.0
7.5
7.0
6.5
6.0
5.5
5.0
4.5
4.0
3.5
3.0
2.5
2.0
1.5
1.0
0.5
f1 (ppm)
4.0
3.8
3.6
3.4
3.2
3.0
2.8
2.6
2.4
f1 (ppm)

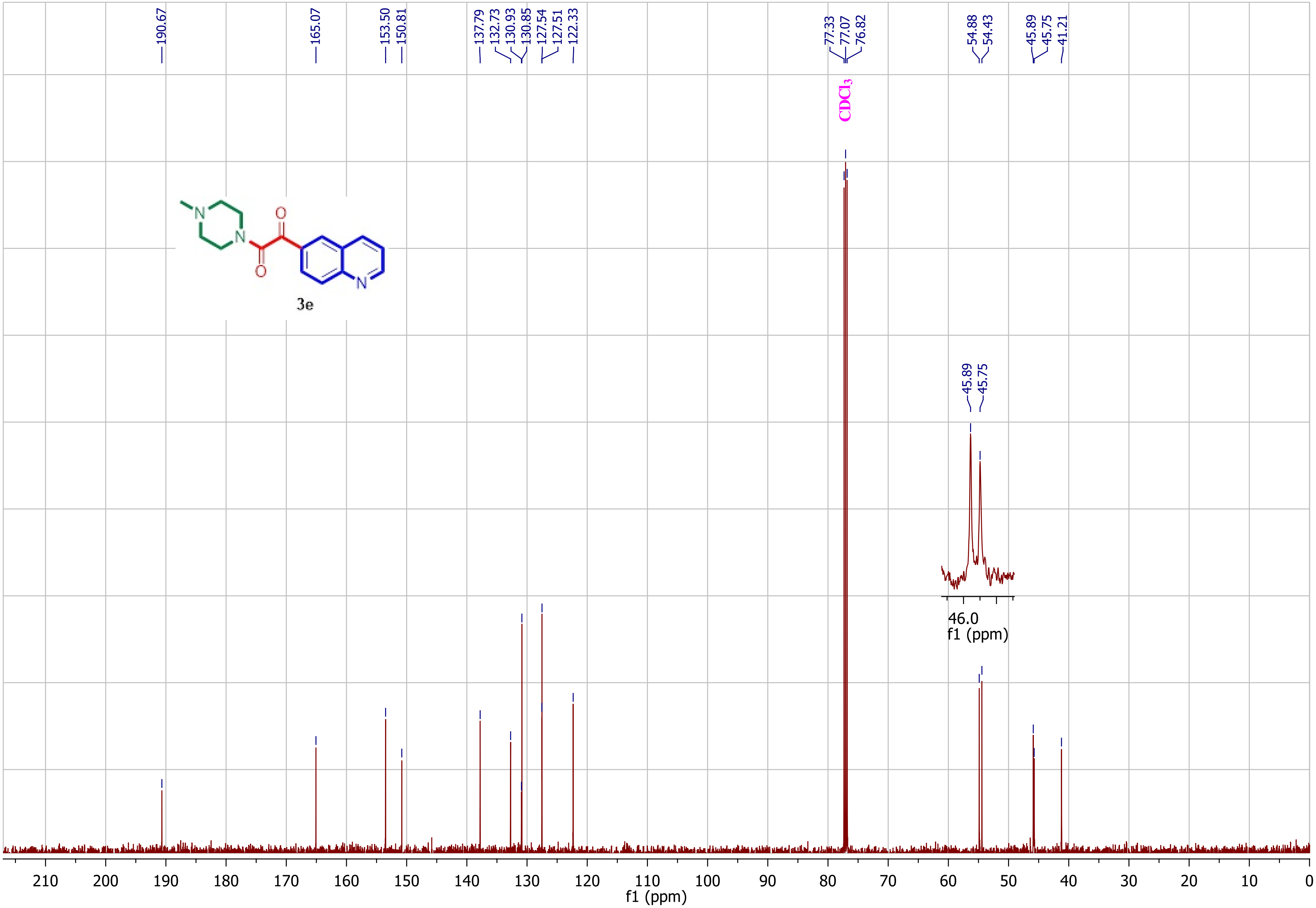
190.67
165.07
153.50
150.81
137.79
132.73
130.93
130.85
127.54
127.51
122.33
77.33
77.07
76.82
CDCl3
54.88
54.43
45.89
45.75
41.21
3e
45.89
45.75
46.0
f1 (ppm)
210 200 190 180 170 160 150 140 130 120 110 100 90 80 70 60 50 40 30 20 10 0
f1 (ppm)

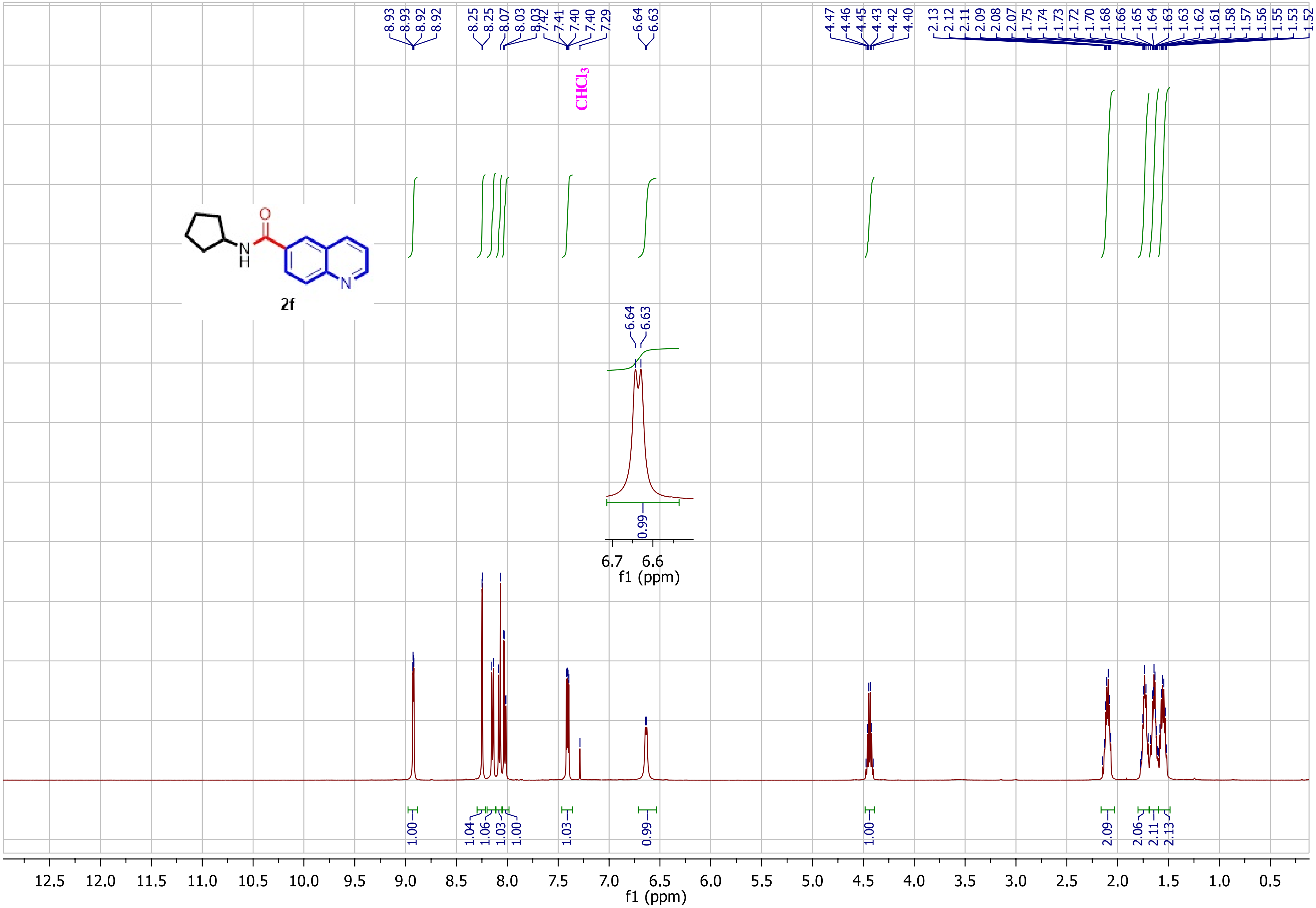
8.93
8.93
8.92
8.92
8.25
8.25
8.07
8.03
8.03
7.42
7.41
7.40
7.40
7.29
6.64
6.63
4.47
4.46
4.45
4.43
4.42
4.40
2.13
2.12
2.11
2.09
2.08
2.07
1.75
1.74
1.73
1.72
1.70
1.68
1.66
1.65
1.64
1.63
1.63
1.62
1.61
1.58
1.57
1.56
1.55
1.53
1.52
CHCl3
O
N
H
N
2f
6.64
6.63
0.99
6.7
6.6
f1 (ppm)
1.00
1.04
1.06
1.03
1.00
1.03
0.99
1.00
2.09
2.06
2.11
2.13
12.5
12.0
11.5
11.0
10.5
10.0
9.5
9.0
8.5
8.0
7.5
7.0
6.5
6.0
5.5
5.0
4.5
4.0
3.5
3.0
2.5
2.0
1.5
1.0
0.5
f1 (ppm)

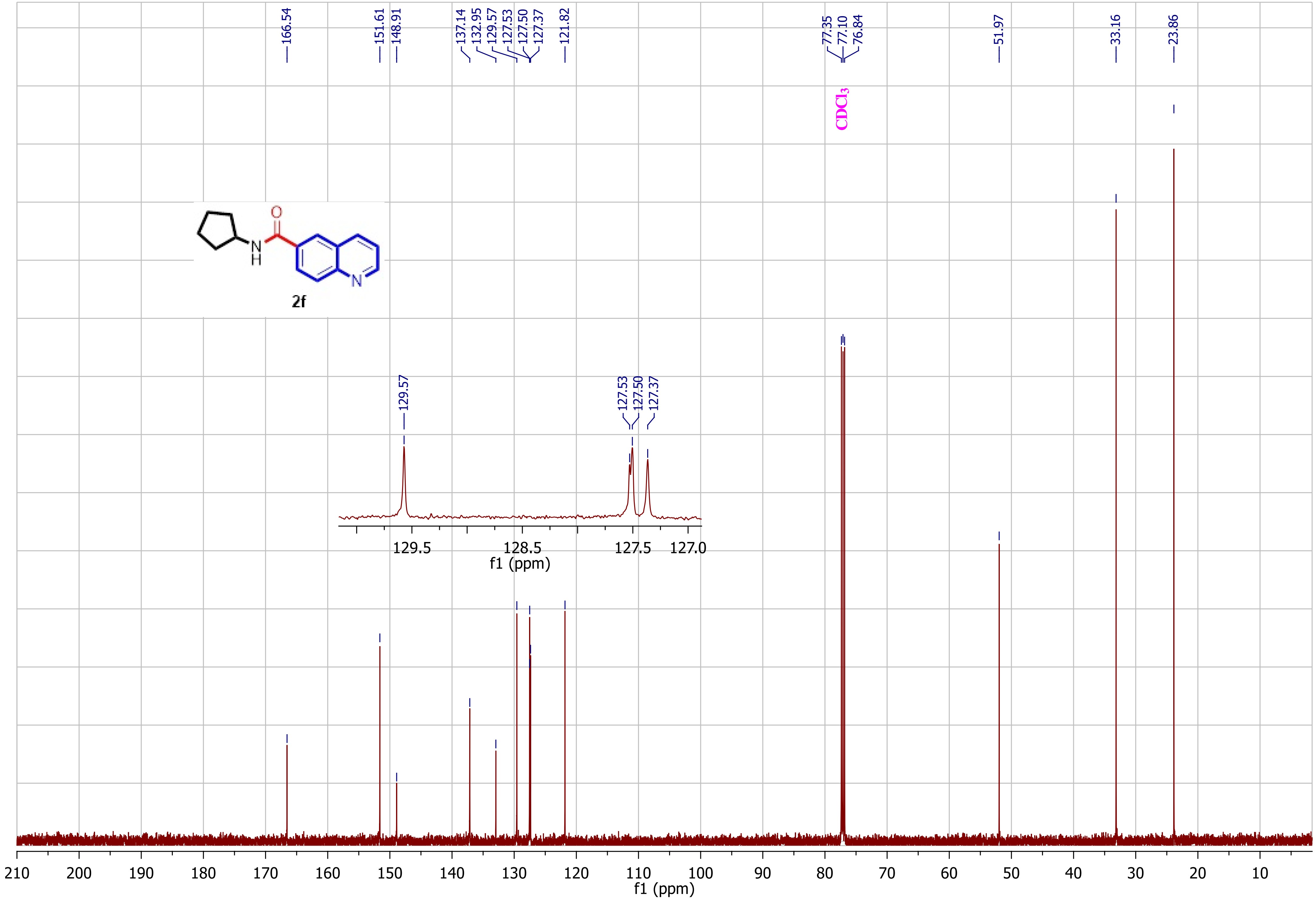

166.54
151.61
148.91
137.14
132.95
129.57
127.53
127.50
127.37
121.82
77.35
77.10
76.84
51.97
33.16
23.86
CDCl3
O
N
H
N
2f
129.57
127.53
127.50
127.37
129.5
128.5
127.5
127.0
f1 (ppm)
210
200
190
180
170
160
150
140
130
120
110
100
90
80
70
60
50
40
30
20
10
f1 (ppm)

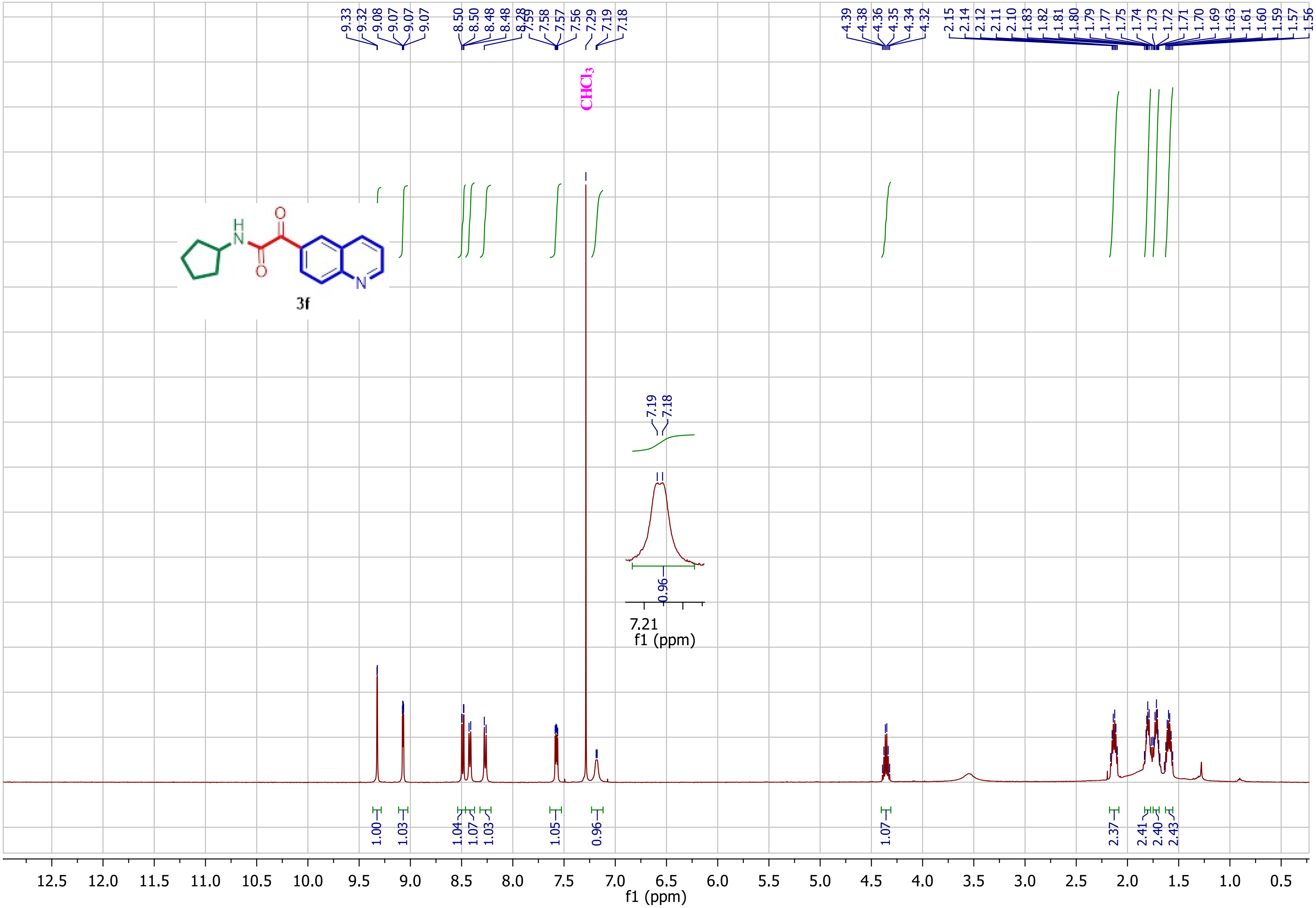

9.33 9.32 9.08 9.07 9.07 9.07
8.50 8.50 8.48 8.48 8.28 7.59 7.58 7.57 7.56 7.29 7.19 7.18
4.39 4.38 4.36 4.35 4.34 4.32
2.15 2.14 2.12 2.11 2.10 1.83 1.82 1.81 1.80 1.79 1.77 1.75 1.74 1.73 1.72 1.71 1.70 1.69 1.63 1.61 1.60 1.59 1.57 1.56
$CHCl_3$
3f
7.19 7.18
0.96
7.21
f1 (ppm)
1.00 1.03 1.04 1.07 1.03 1.05 0.96 1.07 2.37 2.41 2.40 2.43
12.5 12.0 11.5 11.0 10.5 10.0 9.5 9.0 8.5 8.0 7.5 7.0 6.5 6.0 5.5 5.0 4.5 4.0 3.5 3.0 2.5 2.0 1.5 1.0 0.5
f1 (ppm)

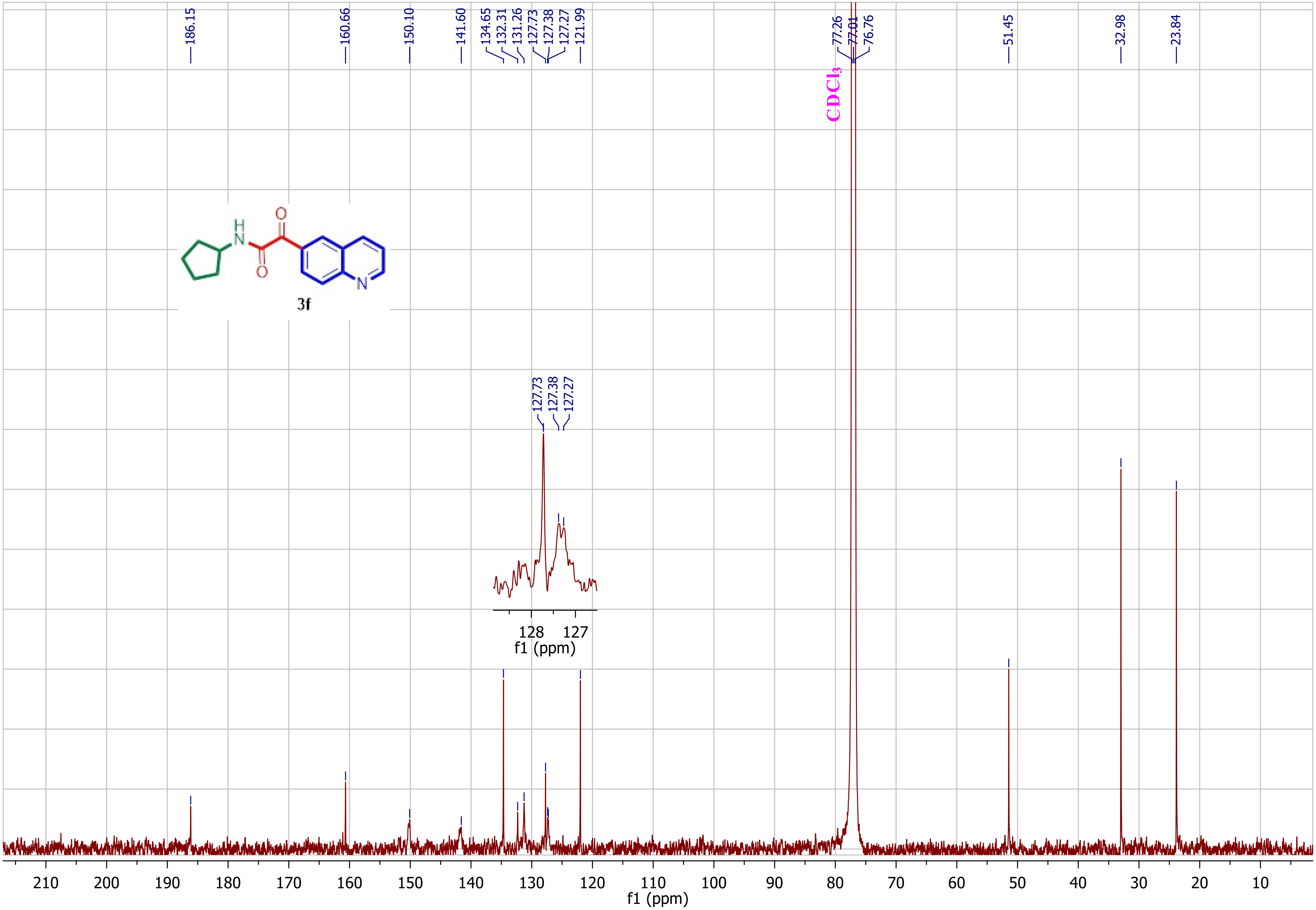

186.15
160.66
150.10
141.60
134.65
132.31
131.26
127.73
127.38
127.27
121.99
77.26
77.01
76.76
51.45
32.98
23.84
CDCl3
3f
127.73
127.38
127.27
128
127
f1 (ppm)
210
200
190
180
170
160
150
140
130
120
110
100
90
80
70
60
50
40
30
20
10
f1 (ppm)

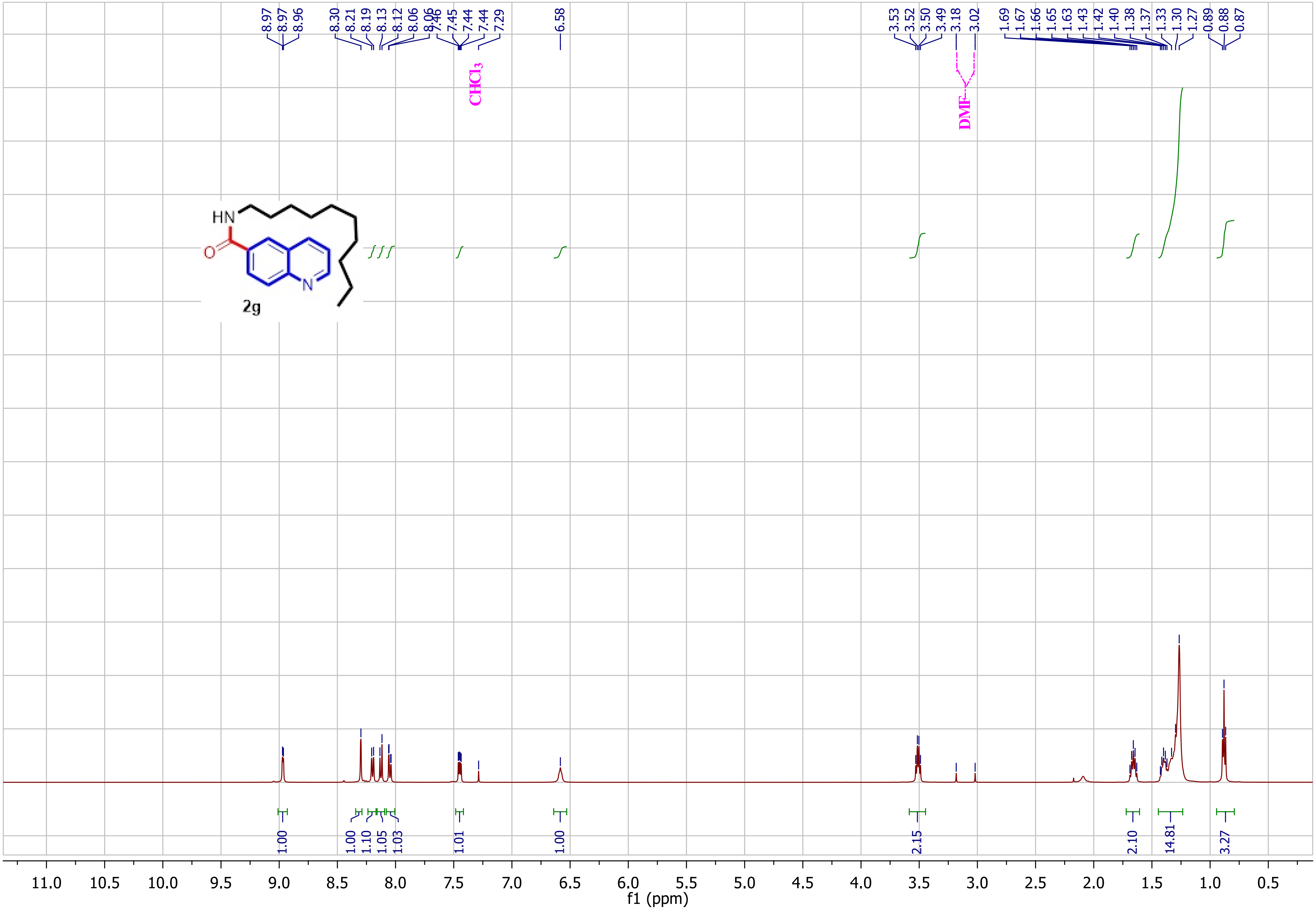
8.97
8.97
8.96
8.30
8.21
8.19
8.13
8.12
8.06
8.06
7.46
7.45
7.44
7.44
7.29
6.58
3.53
3.52
3.50
3.49
3.18
3.02
1.69
1.67
1.66
1.65
1.63
1.43
1.42
1.40
1.38
1.37
1.33
1.30
1.27
0.89
0.88
0.87
CHCl3
DMF
HN
O
N
2g
1.00
1.00
1.10
1.05
1.03
1.01
1.00
2.15
2.10
14.81
3.27
11.0
10.5
10.0
9.5
9.0
8.5
8.0
7.5
7.0
6.5
6.0
5.5
5.0
4.5
4.0
3.5
3.0
2.5
2.0
1.5
1.0
0.5
f1 (ppm)

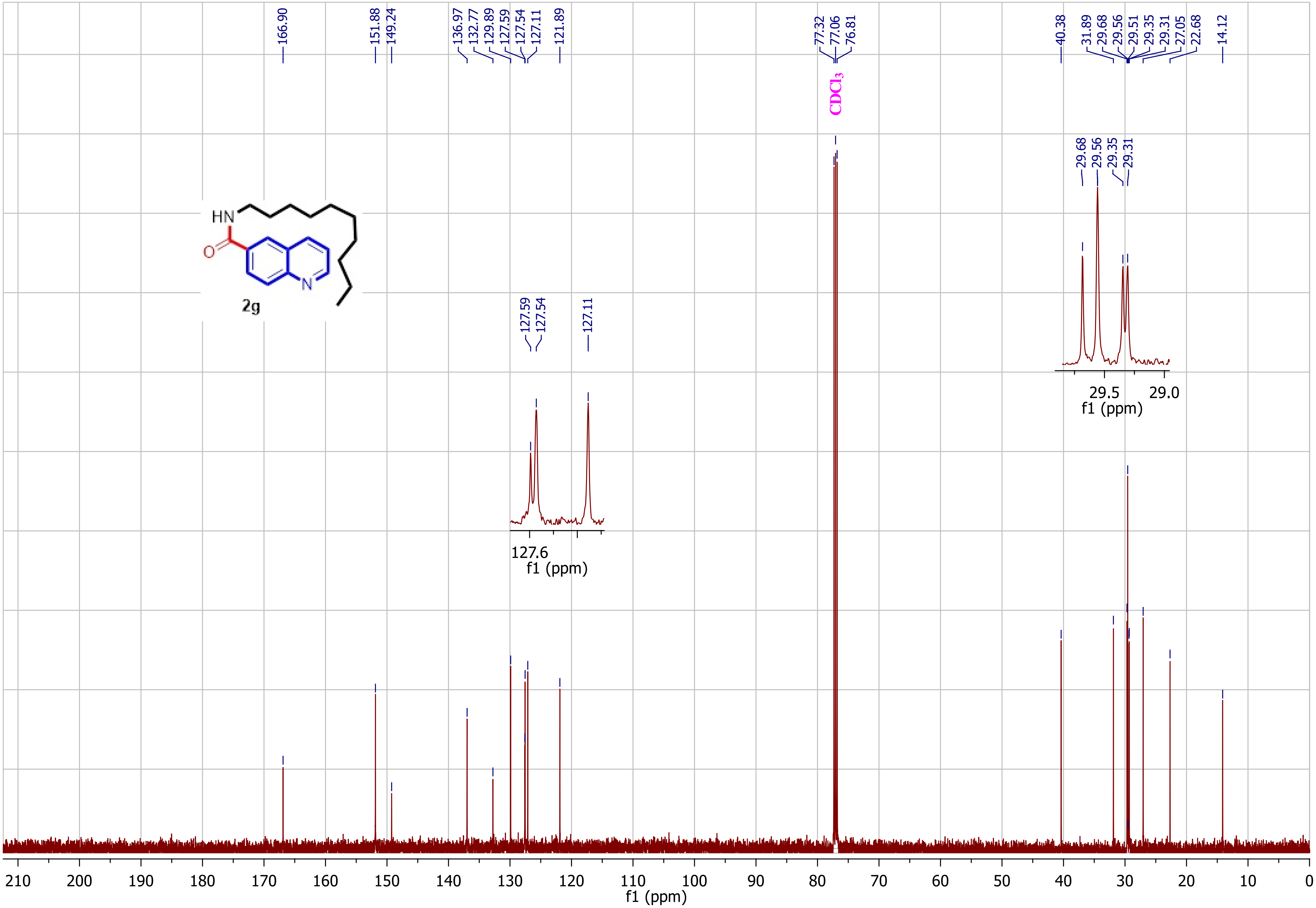

166.90
151.88
149.24
136.97
132.77
129.89
127.59
127.54
127.11
121.89
77.32
77.06
76.81
CDCl3
40.38
31.89
29.68
29.56
29.51
29.35
29.31
27.05
22.68
14.12
HN
O
N
2g
127.59
127.54
127.11
127.6
f1 (ppm)
29.68
29.56
29.35
29.31
29.5
29.0
f1 (ppm)
210 200 190 180 170 160 150 140 130 120 110 100 90 80 70 60 50 40 30 20 10 0
f1 (ppm)

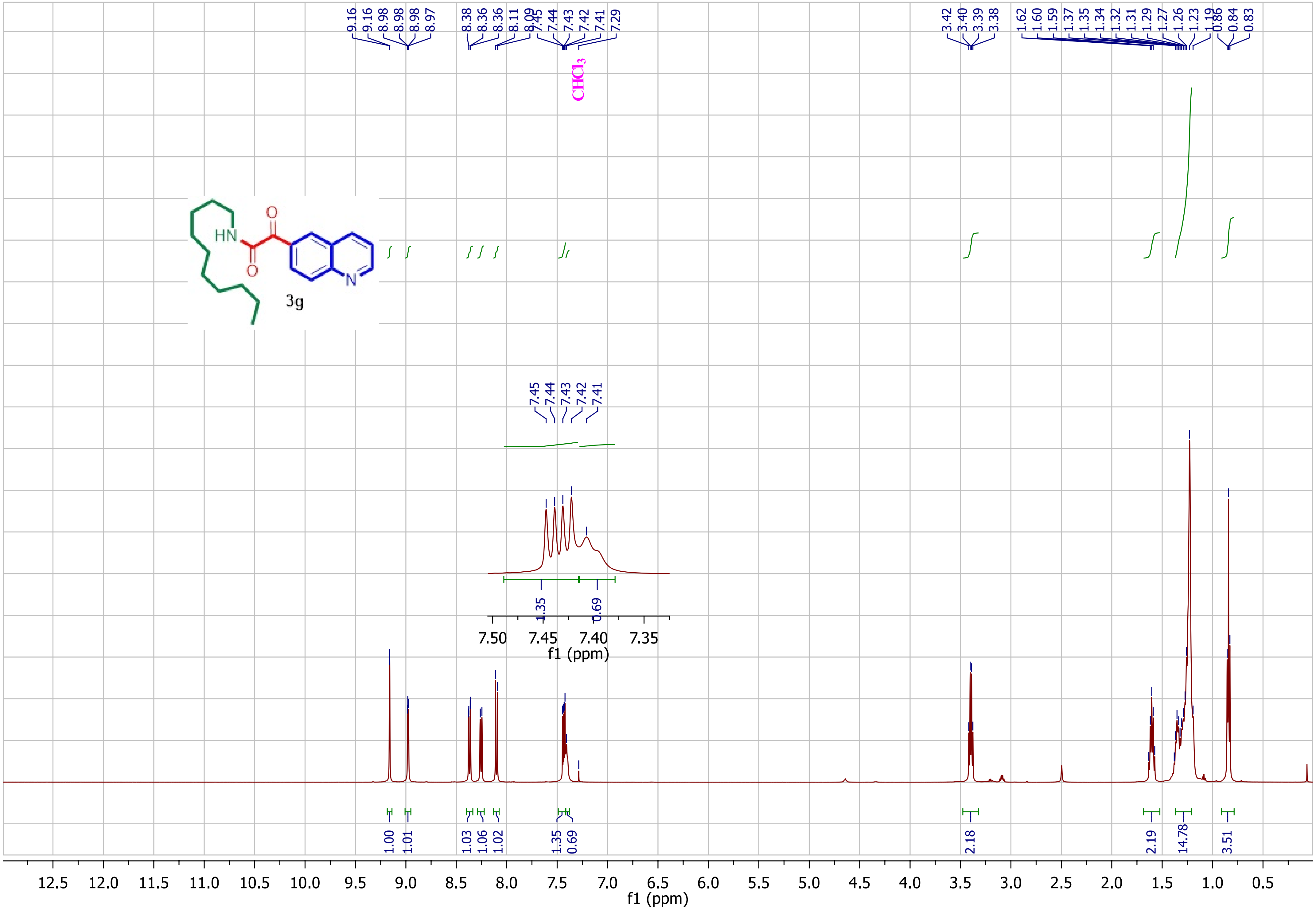

9.16
9.16
8.98
8.98
8.98
8.97
8.38
8.36
8.36
8.11
8.09
7.45
7.44
7.43
7.42
7.41
7.29
CHCl3
3.42
3.40
3.39
3.38
1.62
1.60
1.59
1.37
1.35
1.34
1.32
1.31
1.29
1.27
1.26
1.23
1.19
0.86
0.84
0.83
HN
O
O
N
3g
7.45
7.44
7.43
7.42
7.41
1.35
0.69
7.50 7.45 7.40 7.35
f1 (ppm)
1.00
1.01
1.03
1.06
1.02
1.35
0.69
2.18
2.19
14.78
3.51
12.5 12.0 11.5 11.0 10.5 10.0 9.5 9.0 8.5 8.0 7.5 7.0 6.5 6.0 5.5 5.0 4.5 4.0 3.5 3.0 2.5 2.0 1.5 1.0 0.5
f1 (ppm)

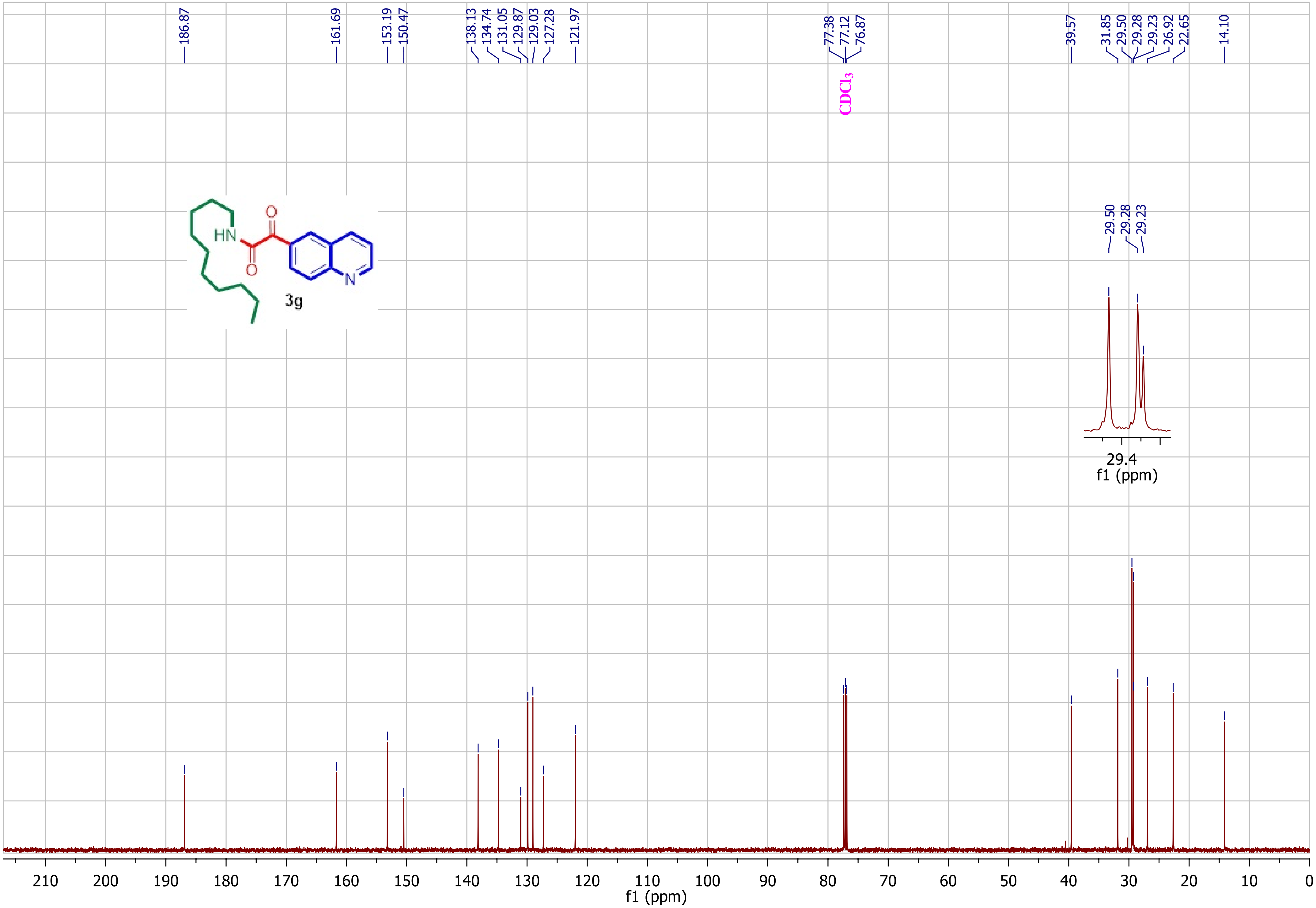
186.87
161.69
153.19
150.47
138.13
134.74
131.05
129.87
129.03
127.28
121.97
77.38
77.12
76.87
CDCl3
39.57
31.85
29.50
29.28
29.23
26.92
22.65
14.10
HN
O
O
N
3g
29.50
29.28
29.23
29.4
f1 (ppm)
210 200 190 180 170 160 150 140 130 120 110 100 90 80 70 60 50 40 30 20 10 0
f1 (ppm)

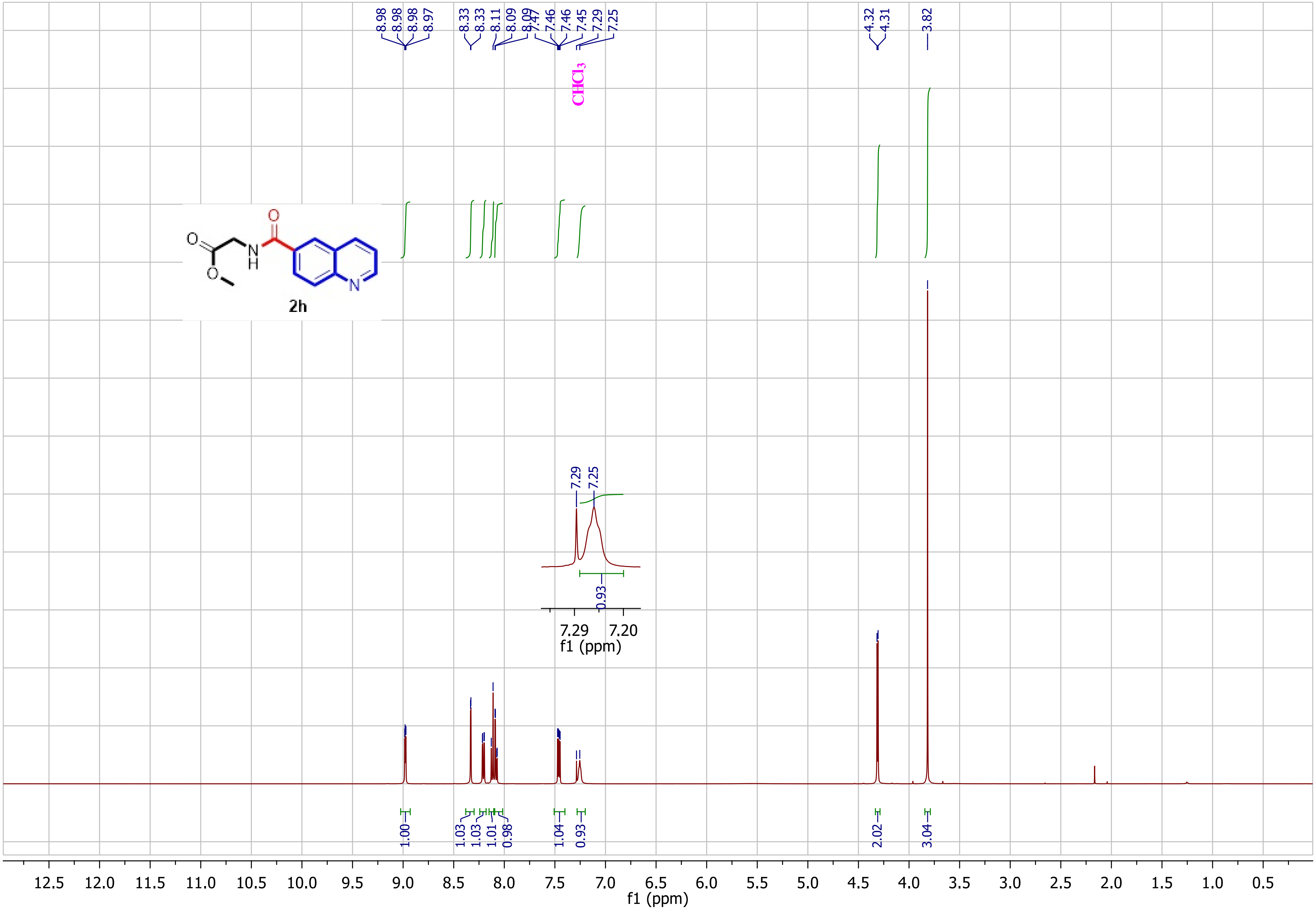
8.98
8.98
8.98
8.97
8.33
8.33
8.11
8.09
8.09
7.47
7.46
7.46
7.45
7.29
7.25
4.32
4.31
3.82
CHCl3
2h
1.00
1.03
1.03
1.01
0.98
1.04
0.93
2.02
3.04
7.29
7.20
f1 (ppm)

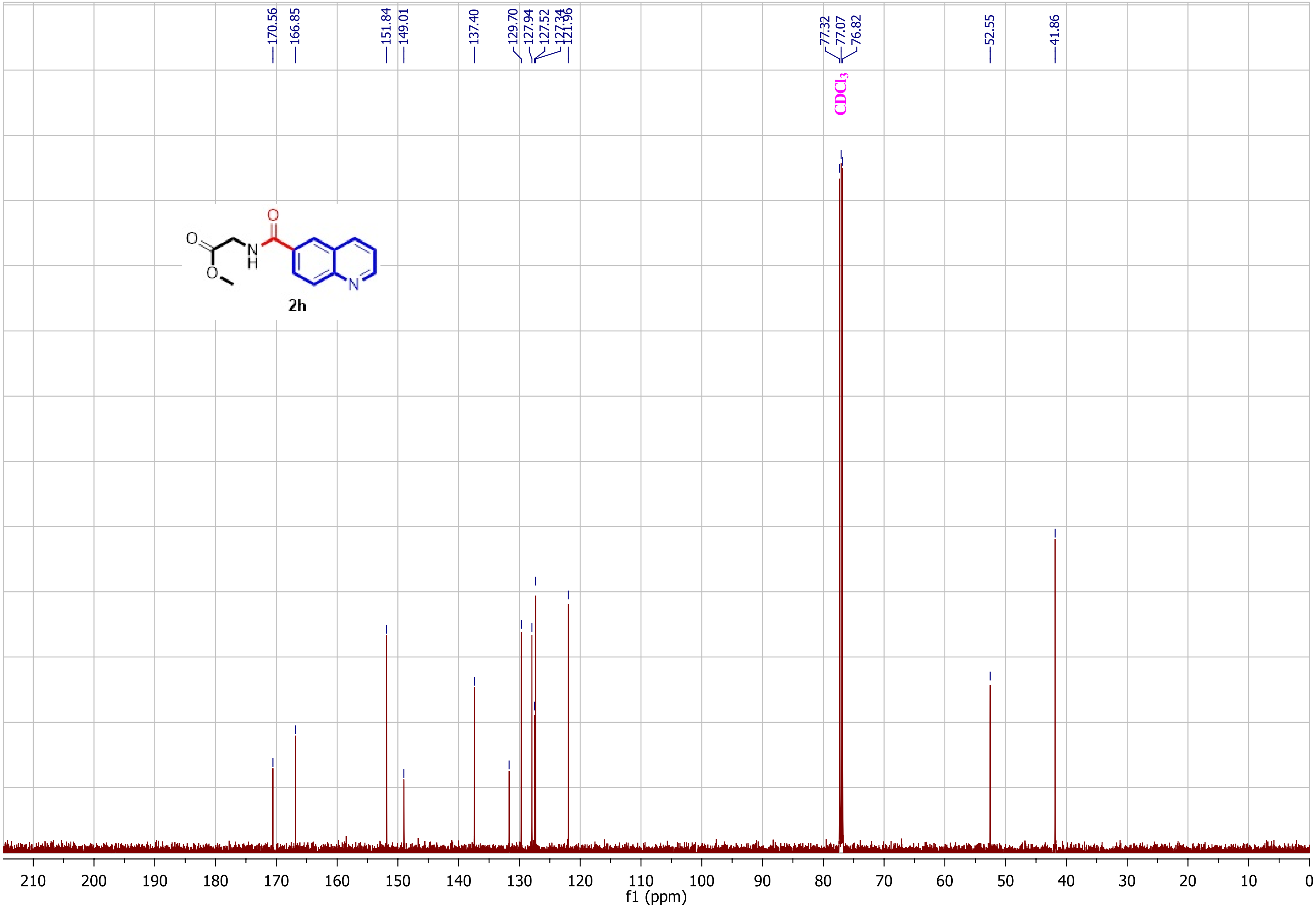
170.56
166.85
151.84
149.01
137.40
129.70
127.94
127.52
127.34
121.96
77.32
77.07
76.82
52.55
41.86
CDCl3
O
N
H
2h
210 200 190 180 170 160 150 140 130 120 110 100 90 80 70 60 50 40 30 20 10 0
f1 (ppm)

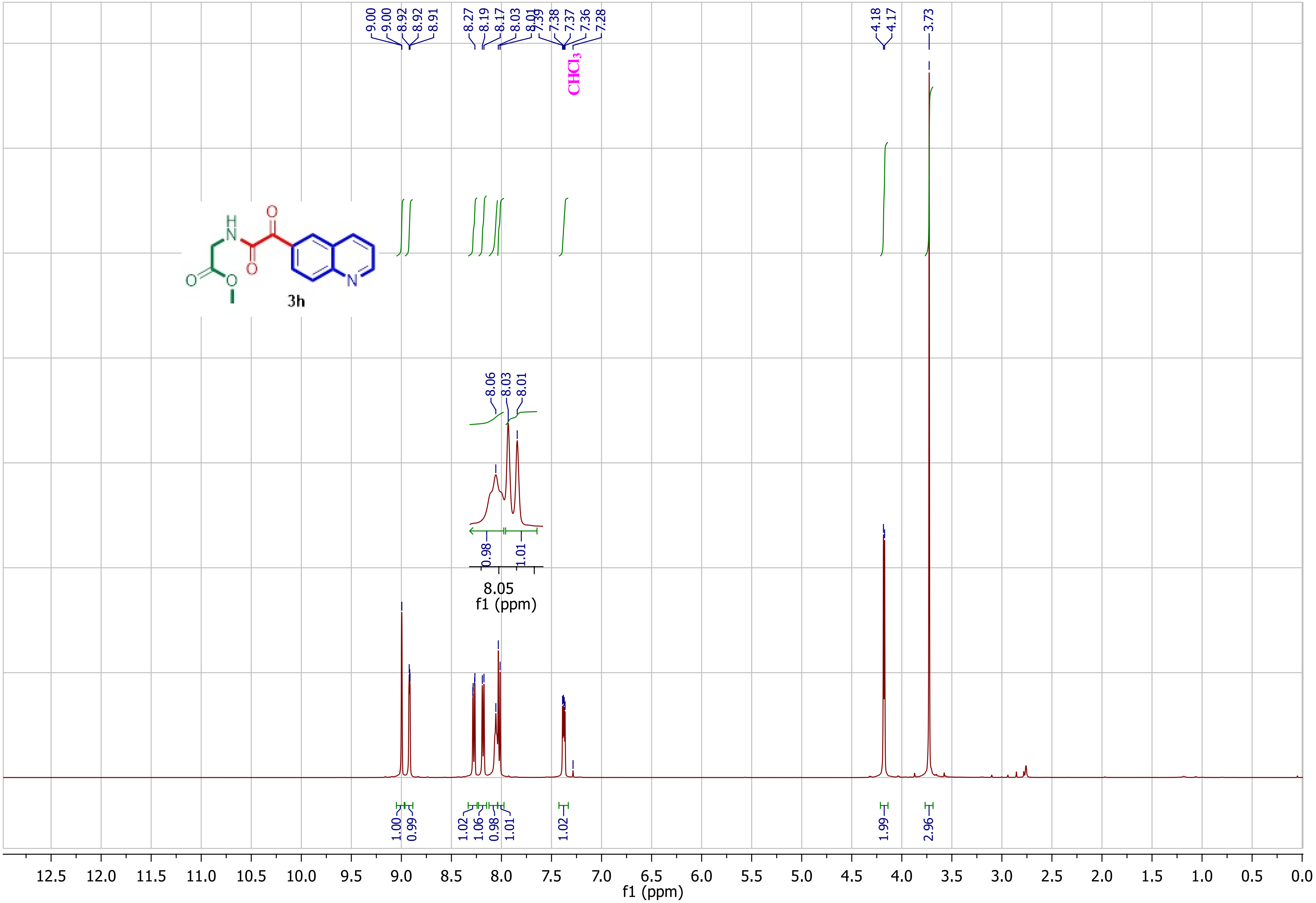

9.00
9.00
8.92
8.92
8.91
8.27
8.19
8.17
8.03
8.01
7.39
7.38
7.37
7.36
7.28
4.18
4.17
3.73
CHCl3
3h
8.06
8.03
8.01
0.98
1.01
8.05
f1 (ppm)
1.00
0.99
1.02
1.06
0.98
1.01
1.02
1.99
2.96
f1 (ppm)

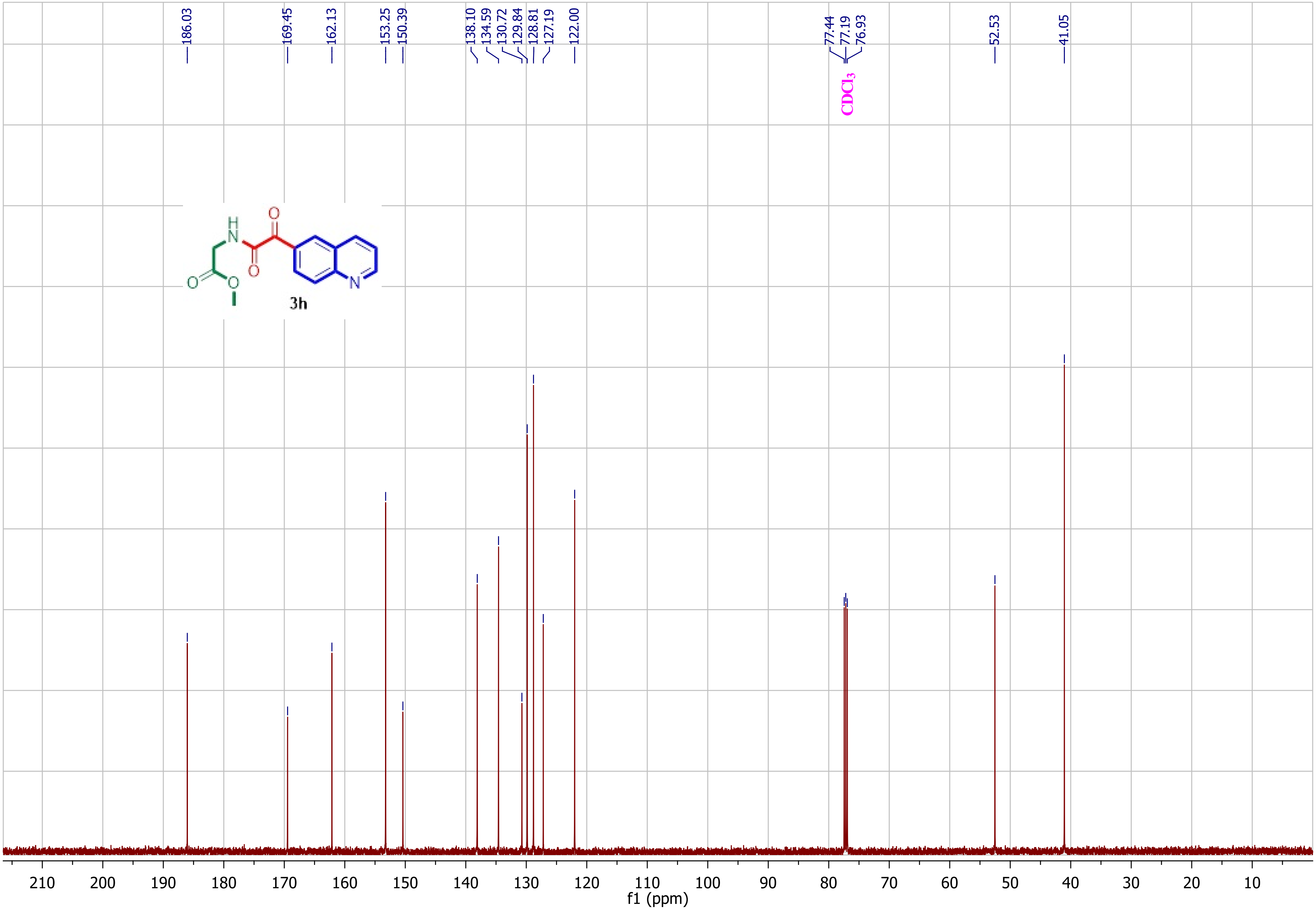

186.03
169.45
162.13
153.25
150.39
138.10
134.59
130.72
129.84
128.81
127.19
122.00
77.44
77.19
76.93
52.53
41.05
CDCl3
3h
210 200 190 180 170 160 150 140 130 120 110 100 90 80 70 60 50 40 30 20 10
f1 (ppm)

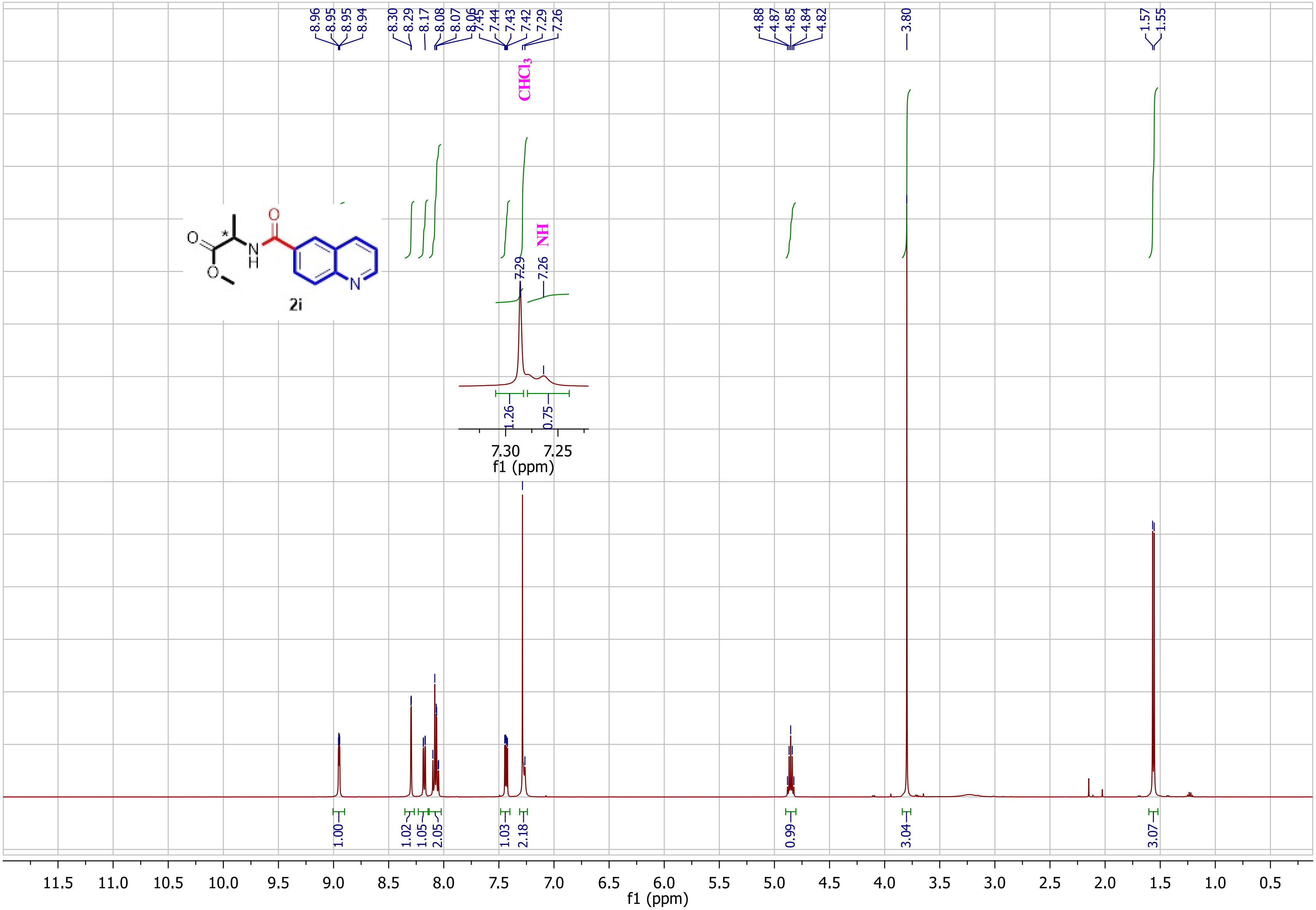

8.96
8.95
8.95
8.94
8.30
8.29
8.17
8.08
8.07
8.06
7.45
7.44
7.43
7.42
7.29
7.26
4.88
4.87
4.85
4.84
4.82
3.80
1.57
1.55
CHCl3
NH
2i
1.26
0.75
7.30
7.25
f1 (ppm)
1.00
1.02
1.05
2.05
1.03
2.18
0.99
3.04
3.07
11.5
11.0
10.5
10.0
9.5
9.0
8.5
8.0
7.5
7.0
6.5
6.0
5.5
5.0
4.5
4.0
3.5
3.0
2.5
2.0
1.5
1.0
0.5
f1 (ppm)

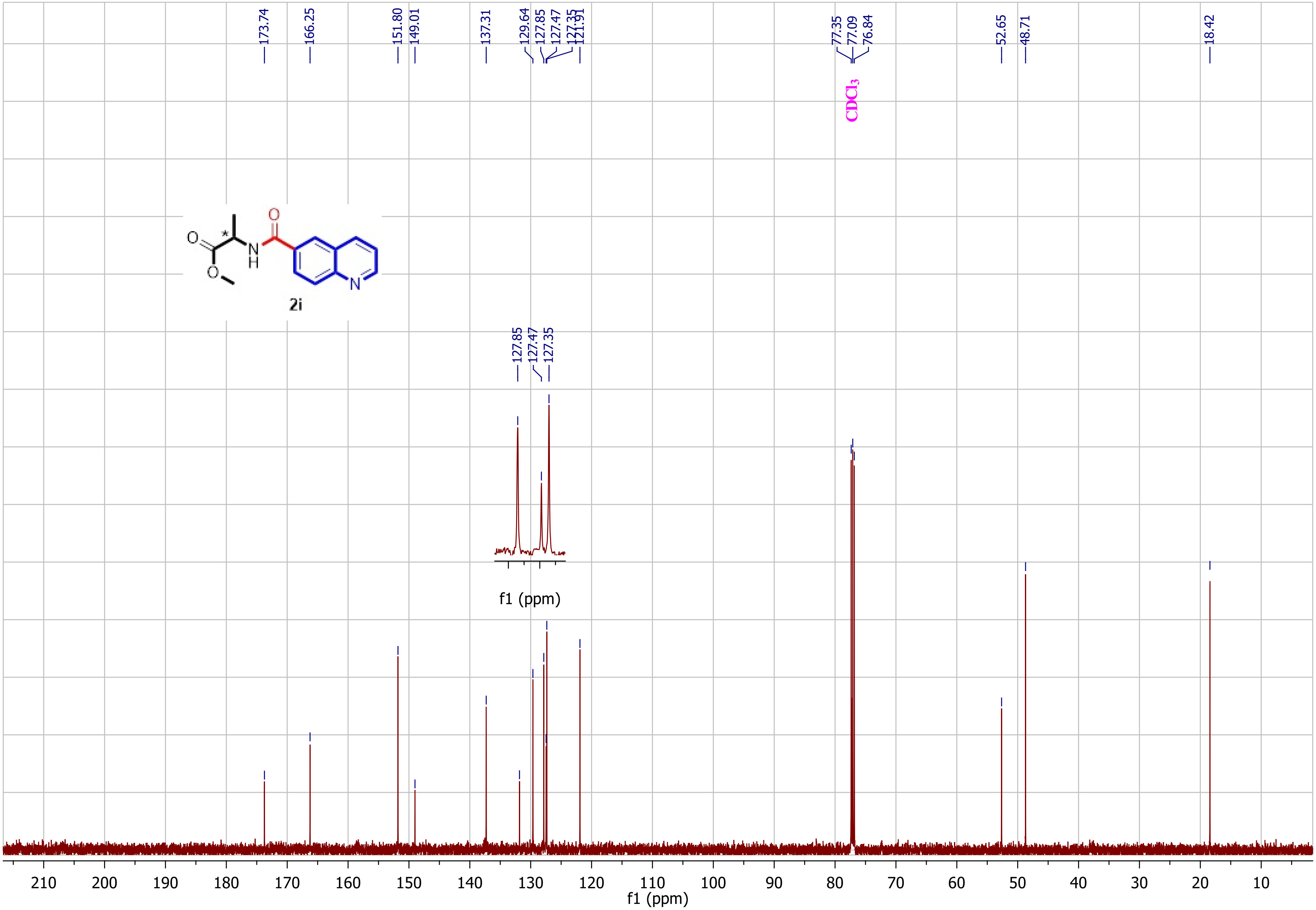

173.74
166.25
151.80
149.01
137.31
129.64
127.85
127.47
127.35
121.91
77.35
77.09
76.84
52.65
48.71
18.42
CDCl3
2i
127.85
127.47
127.35
f1 (ppm)
210 200 190 180 170 160 150 140 130 120 110 100 90 80 70 60 50 40 30 20 10
f1 (ppm)

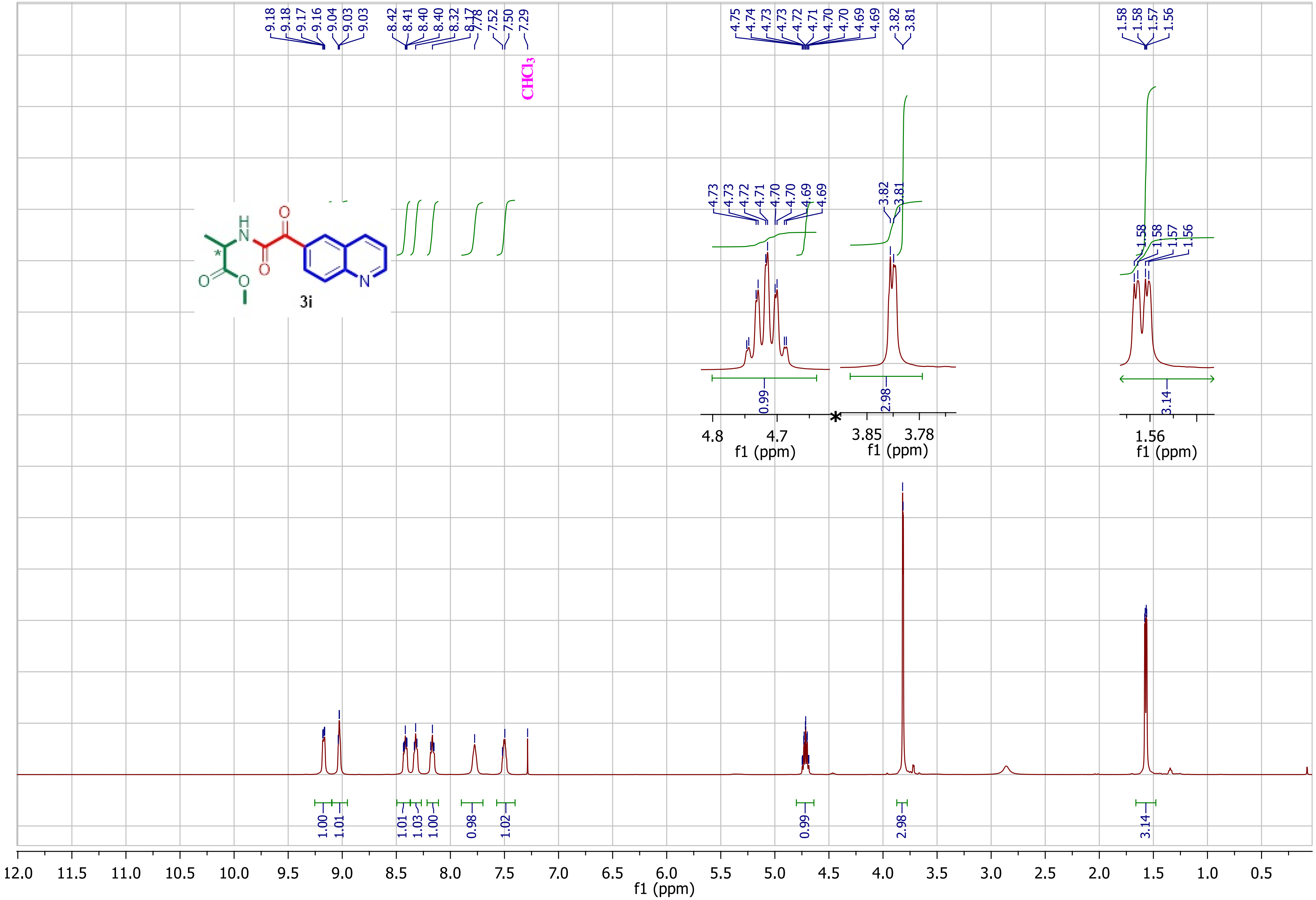
9.18 9.18 9.17 9.16 9.04 9.03 9.03
8.42 8.41 8.40 8.40 8.32 8.17 7.78 7.52 7.50 7.29
CHCl3
4.75 4.74 4.73 4.73 4.72 4.71 4.70 4.70 4.69 4.69 3.82 3.81
1.58 1.58 1.57 1.56
H
N
O
O
O
O
N
3i
4.73 4.73 4.72 4.71 4.70 4.70 4.69 4.69
3.82 3.81
1.58 1.58 1.57 1.56
0.99
2.98
3.14
4.8 4.7
f1 (ppm)
3.85 3.78
f1 (ppm)
1.56
f1 (ppm)
1.00 1.01 1.01 1.03 1.00 0.98 1.02 0.99 2.98 3.14
12.0 11.5 11.0 10.5 10.0 9.5 9.0 8.5 8.0 7.5 7.0 6.5 6.0 5.5 5.0 4.5 4.0 3.5 3.0 2.5 2.0 1.5 1.0 0.5
f1 (ppm)

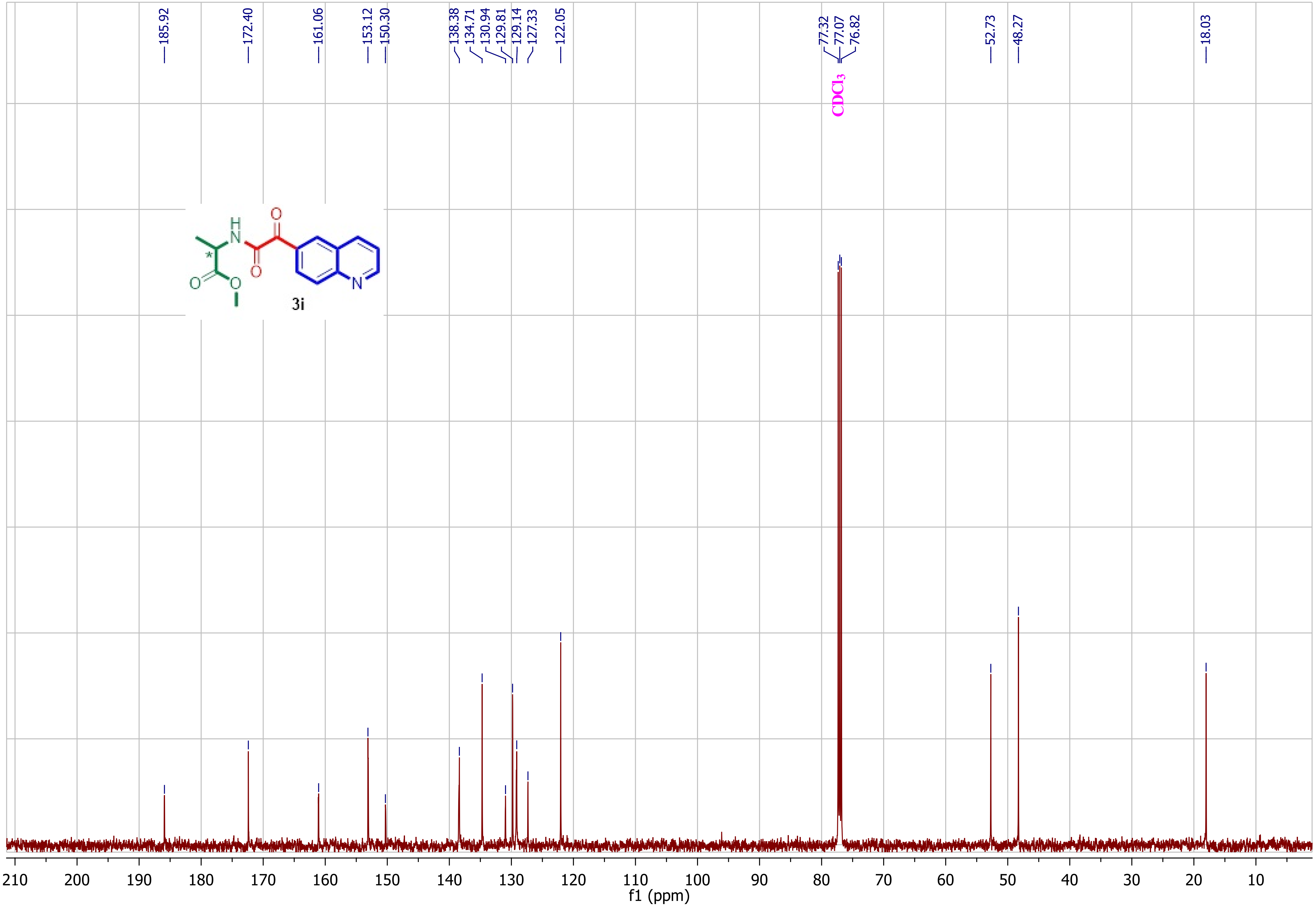

185.92
172.40
161.06
153.12
150.30
138.38
134.71
130.94
129.81
129.14
127.33
122.05
77.32
77.07
76.82
52.73
48.27
18.03
CDCl3
3i
210 200 190 180 170 160 150 140 130 120 110 100 90 80 70 60 50 40 30 20 10
f1 (ppm)

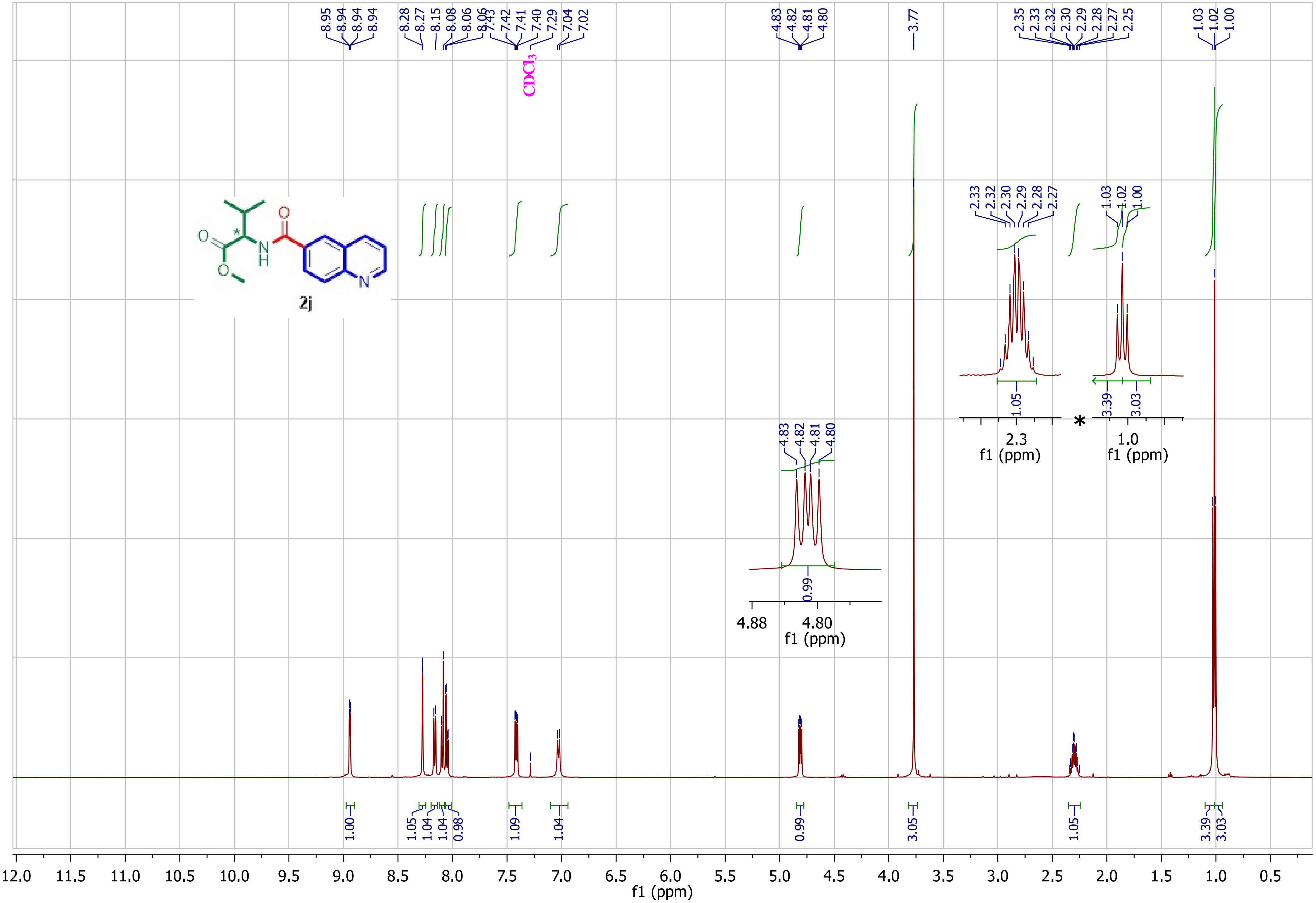

8.95 8.94 8.94 8.94 8.28 8.27 8.15 8.08 8.06 8.05 7.43 7.42 7.41 7.40 7.29 7.04 7.02
4.83 4.82 4.81 4.80
3.77
2.35 2.33 2.32 2.30 2.29 2.28 2.27 2.25
1.03 1.02 1.00
CDCl3
2j
4.83 4.82 4.81 4.80
0.99
4.88
4.80
f1 (ppm)
2.33 2.32 2.30 2.29 2.28 2.27
1.05
2.3
f1 (ppm)
*
1.03 1.02 1.00
3.39 3.03
1.0
f1 (ppm)
1.00 1.05 1.04 1.04 0.98 1.09 1.04 0.99 3.05 1.05 3.39 3.03
12.0 11.5 11.0 10.5 10.0 9.5 9.0 8.5 8.0 7.5 7.0 6.5 6.0 5.5 5.0 4.5 4.0 3.5 3.0 2.5 2.0 1.5 1.0 0.5
f1 (ppm)

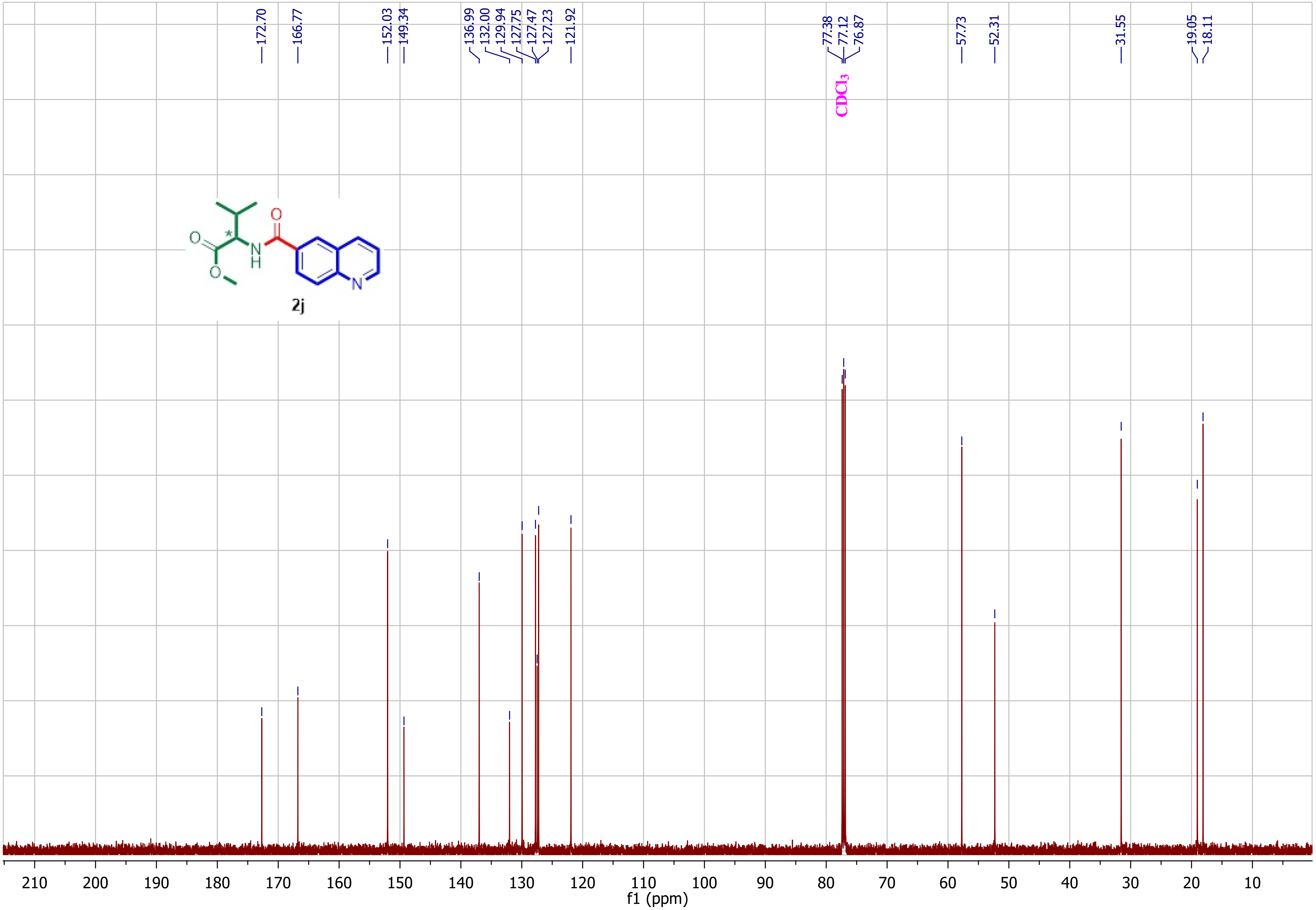
172.70
166.77
152.03
149.34
136.99
132.00
129.94
127.75
127.47
127.23
121.92
77.38
77.12
76.87
57.73
52.31
31.55
19.05
18.11
CDCl3
2j
210 200 190 180 170 160 150 140 130 120 110 100 90 80 70 60 50 40 30 20 10
f1 (ppm)

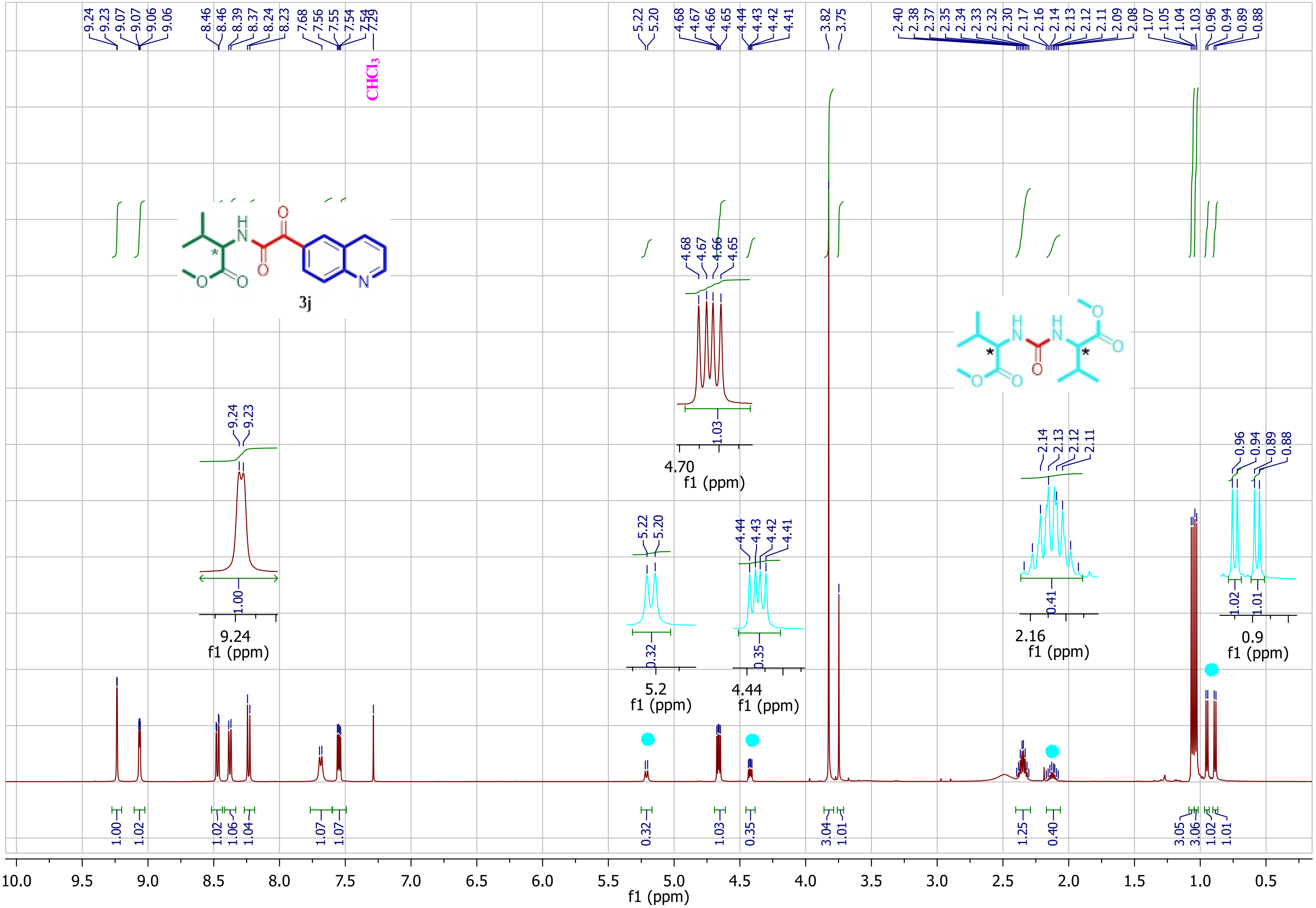
9.24
9.23
9.07
9.07
9.06
9.06
8.46
8.46
8.39
8.37
8.24
8.23
7.68
7.56
7.55
7.54
7.54
7.29
CHCl3
5.22
5.20
4.68
4.67
4.66
4.65
4.44
4.43
4.42
4.41
3.82
3.75
2.40
2.38
2.37
2.35
2.34
2.33
2.32
2.30
2.17
2.16
2.14
2.13
2.12
2.11
2.09
2.08
1.07
1.05
1.04
1.03
0.96
0.94
0.89
0.88
3j
f1 (ppm)

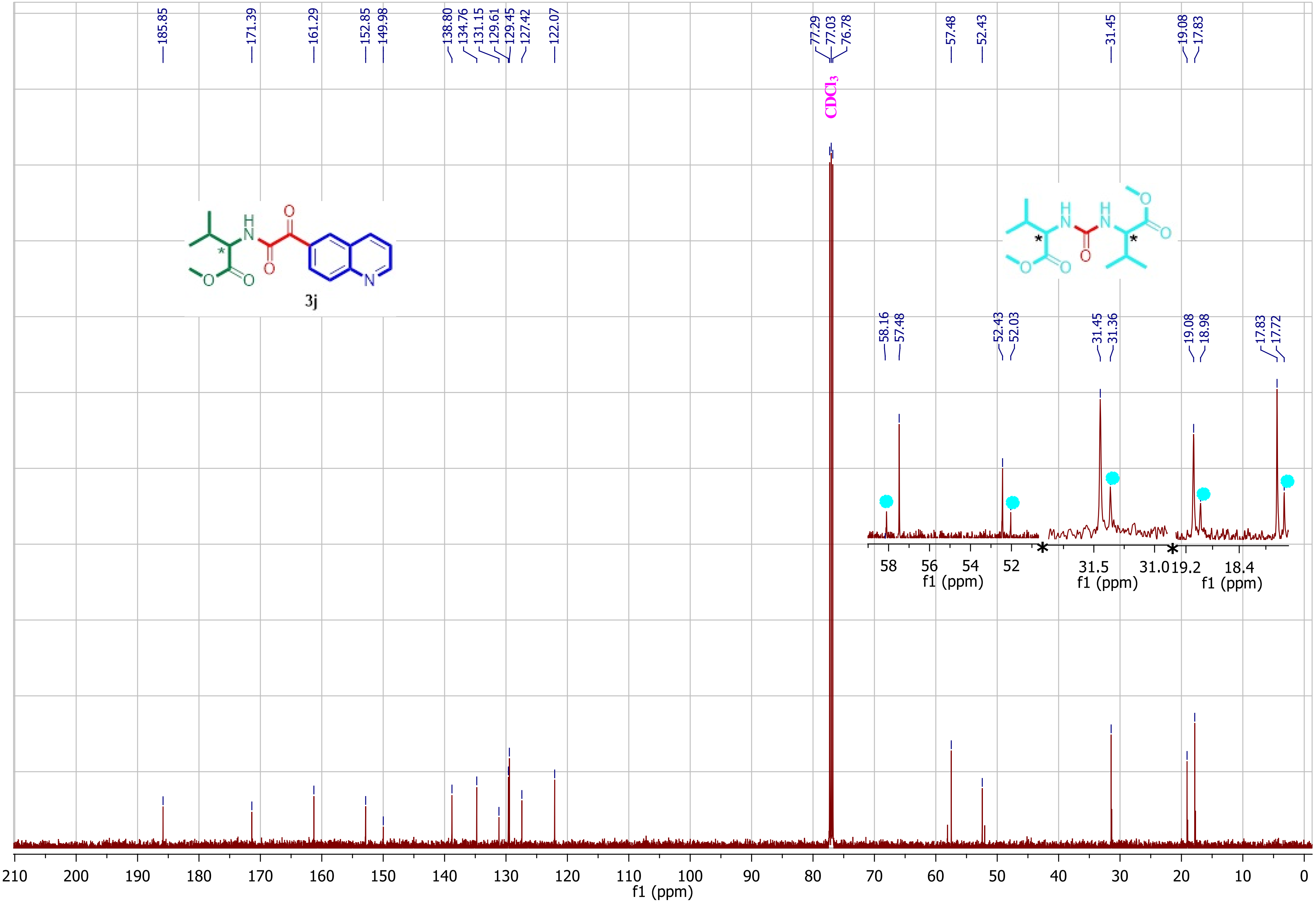

185.85
171.39
161.29
152.85
149.98
138.80
134.76
131.15
129.61
129.45
127.42
122.07
77.29
77.03
76.78
57.48
52.43
31.45
19.08
17.83
CDCl3
3j
58.16
57.48
52.43
52.03
31.45
31.36
19.08
18.98
17.83
17.72
58
56
54
52
f1 (ppm)
31.5
31.0
f1 (ppm)
19.2
18.4
f1 (ppm)
210
200
190
180
170
160
150
140
130
120
110
100
90
80
70
60
50
40
30
20
10
0
f1 (ppm)

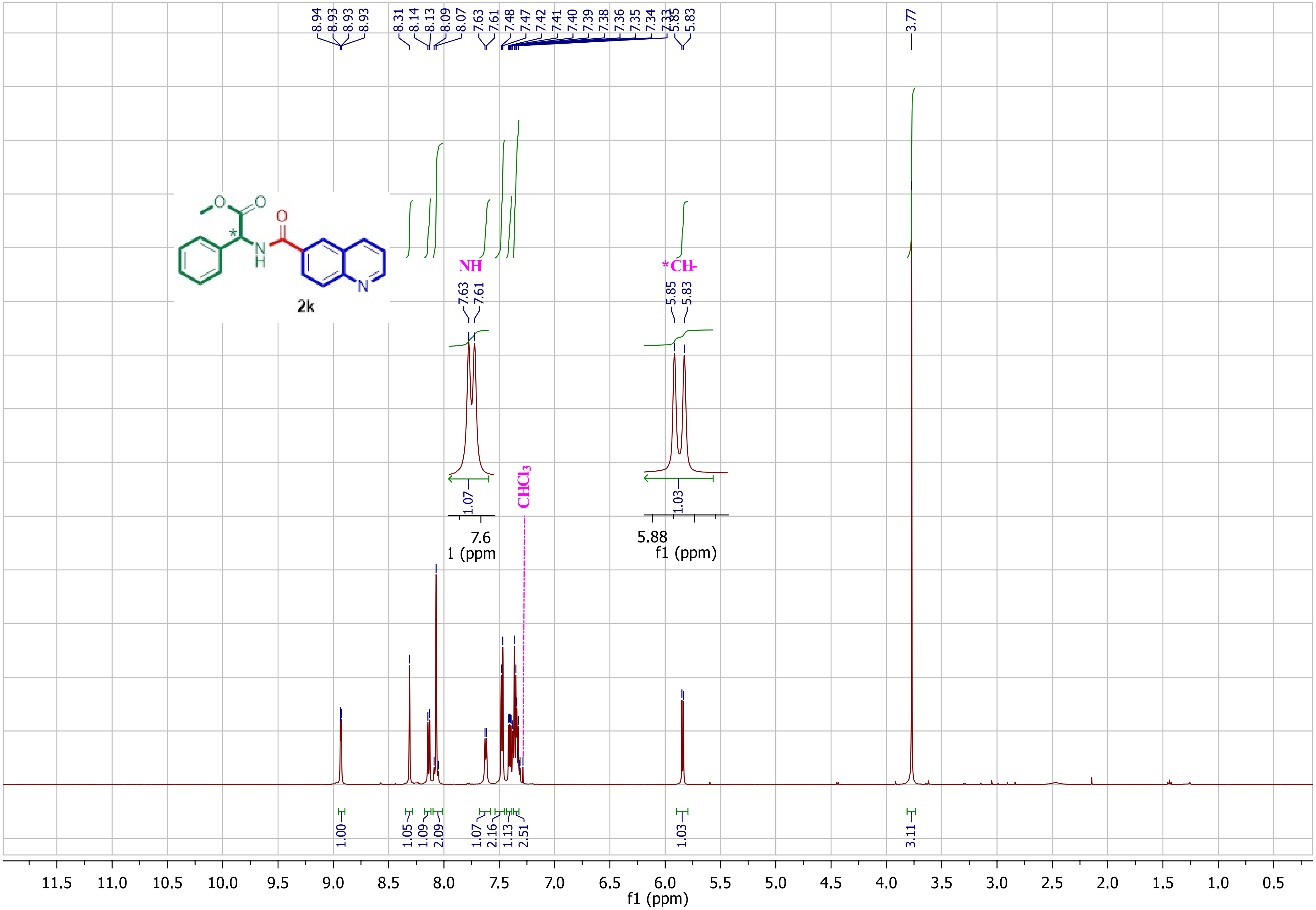

8.94
8.93
8.93
8.93
8.31
8.14
8.13
8.09
8.07
7.63
7.61
7.48
7.47
7.42
7.41
7.40
7.39
7.38
7.36
7.35
7.34
7.33
5.85
5.83
3.77
2k
NH
*CH-
CHCl3
1.07
1.03
7.6
5.88
f1 (ppm)
1.00
1.05
1.09
2.09
2.16
1.13
2.51
3.11
11.5
11.0
10.5
10.0
9.5
9.0
8.5
8.0
7.5
7.0
6.5
6.0
5.5
5.0
4.5
4.0
3.5
3.0
2.5
2.0
1.5
1.0
0.5

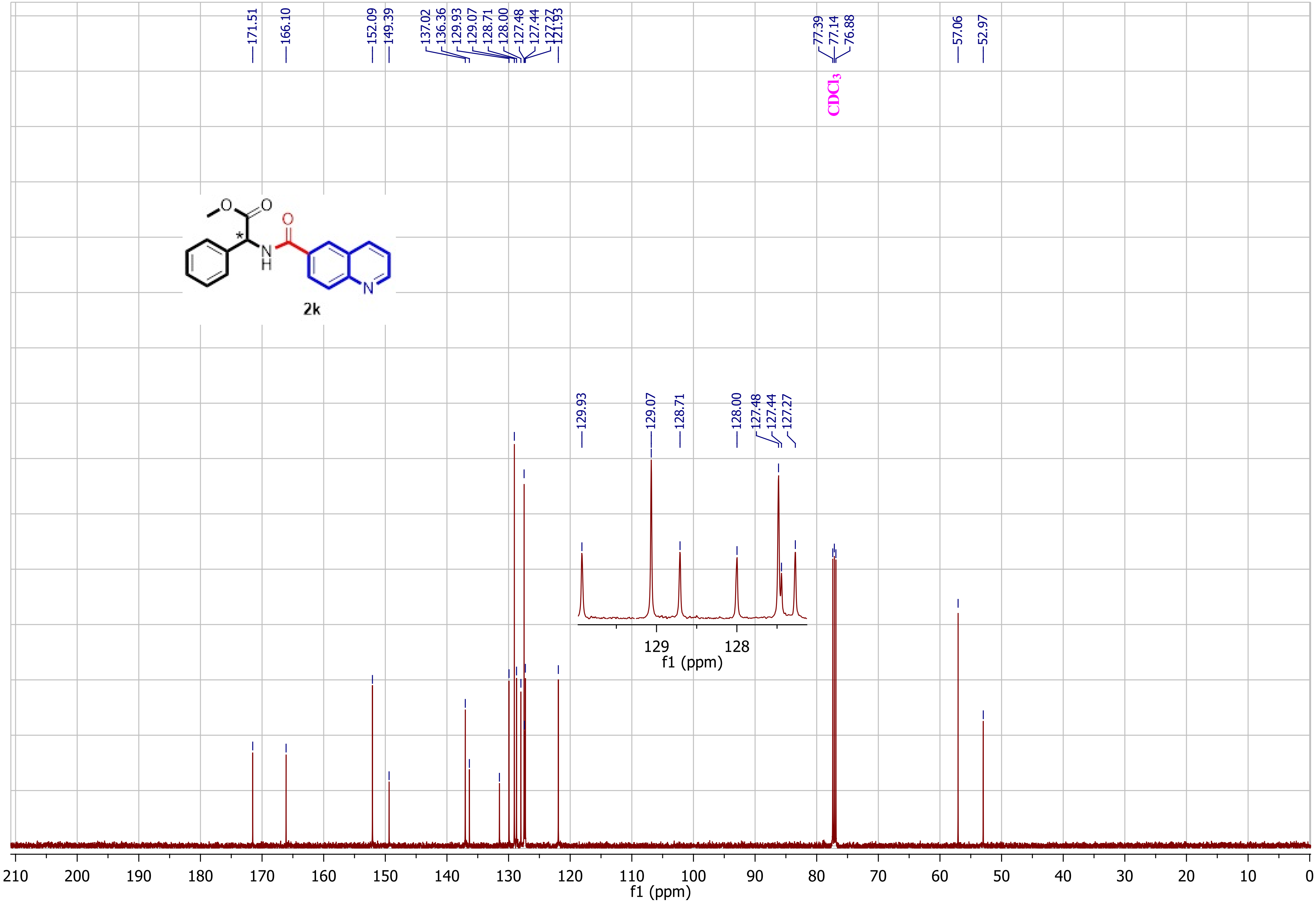
171.51
166.10
152.09
149.39
137.02
136.36
129.93
129.07
128.71
128.00
127.48
127.44
127.27
121.93
77.39
77.14
76.88
57.06
52.97
$CDCl_3$
2k
129.93
129.07
128.71
128.00
127.48
127.44
127.27
129
128
f1 (ppm)
210 200 190 180 170 160 150 140 130 120 110 100 90 80 70 60 50 40 30 20 10 0
f1 (ppm)

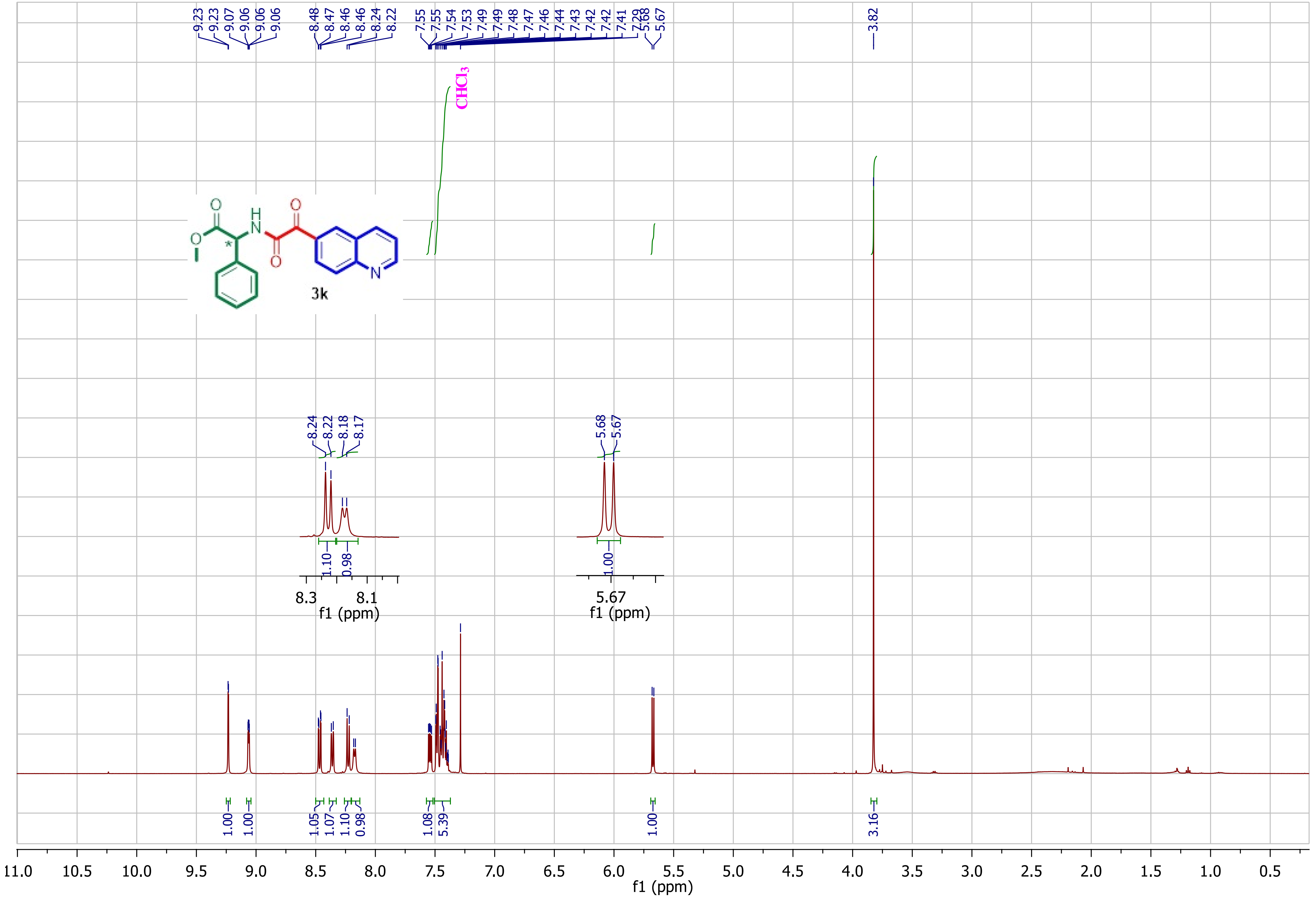
9.23
9.23
9.07
9.06
9.06
9.06
8.48
8.47
8.46
8.46
8.24
8.22
7.55
7.55
7.54
7.53
7.49
7.49
7.48
7.47
7.46
7.44
7.43
7.42
7.42
7.41
7.29
5.68
5.67
3.82
CHCl3
3k
8.24
8.22
8.18
8.17
1.10
0.98
8.3
8.1
f1 (ppm)
5.68
5.67
1.00
5.67
f1 (ppm)
1.00
1.00
1.05
1.07
1.10
0.98
1.08
5.39
1.00
3.16
11.0
10.5
10.0
9.5
9.0
8.5
8.0
7.5
7.0
6.5
6.0
5.5
5.0
4.5
4.0
3.5
3.0
2.5
2.0
1.5
1.0
0.5
f1 (ppm)

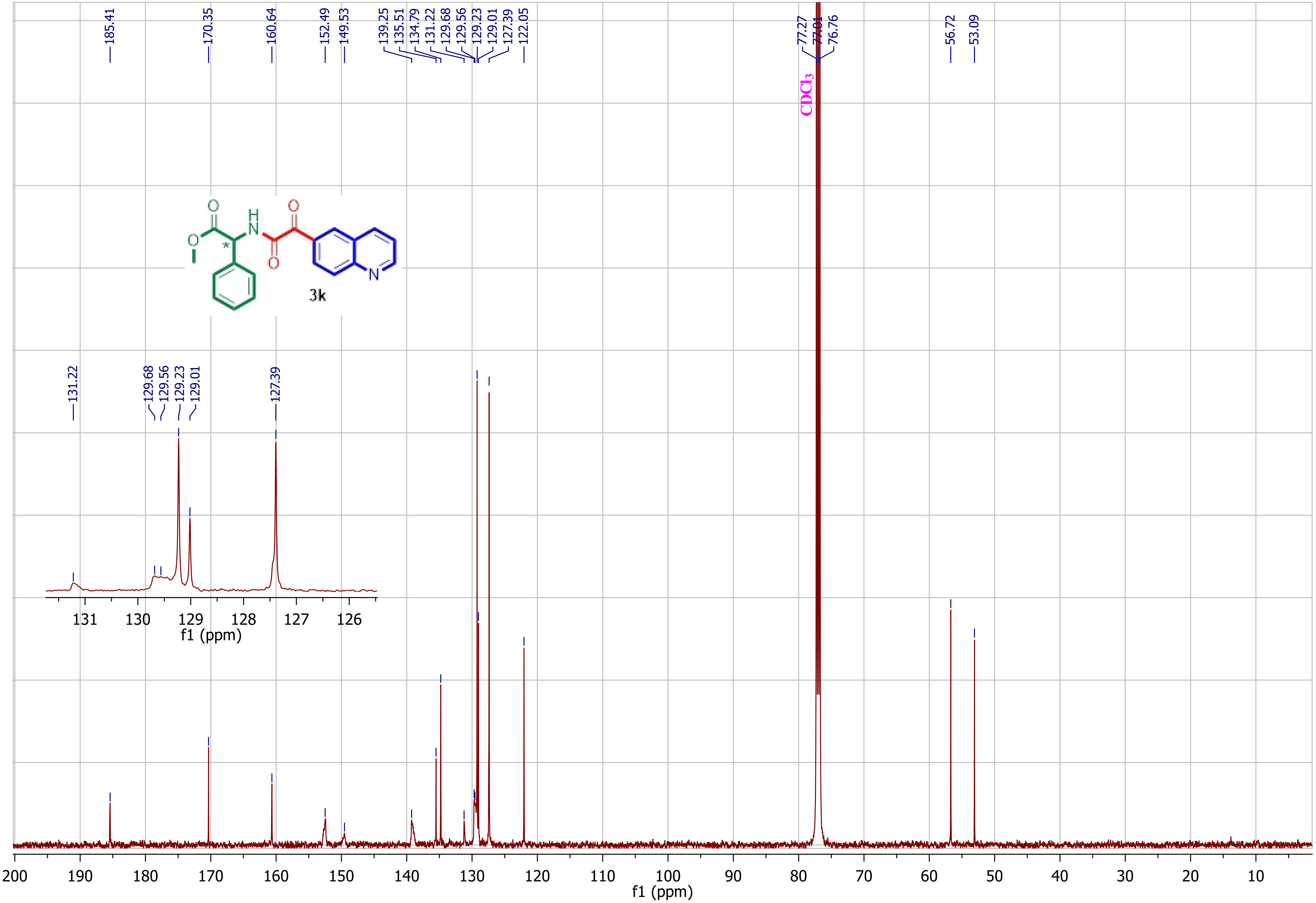
185.41
170.35
160.64
152.49
149.53
139.25
135.51
134.79
131.22
129.68
129.56
129.23
129.01
127.39
122.05
77.27
77.01
76.76
56.72
53.09
CDCl3
3k
131.22
129.68
129.56
129.23
129.01
127.39
131
130
129
128
127
126
f1 (ppm)
200
190
180
170
160
150
140
130
120
110
100
90
80
70
60
50
40
30
20
10
f1 (ppm)

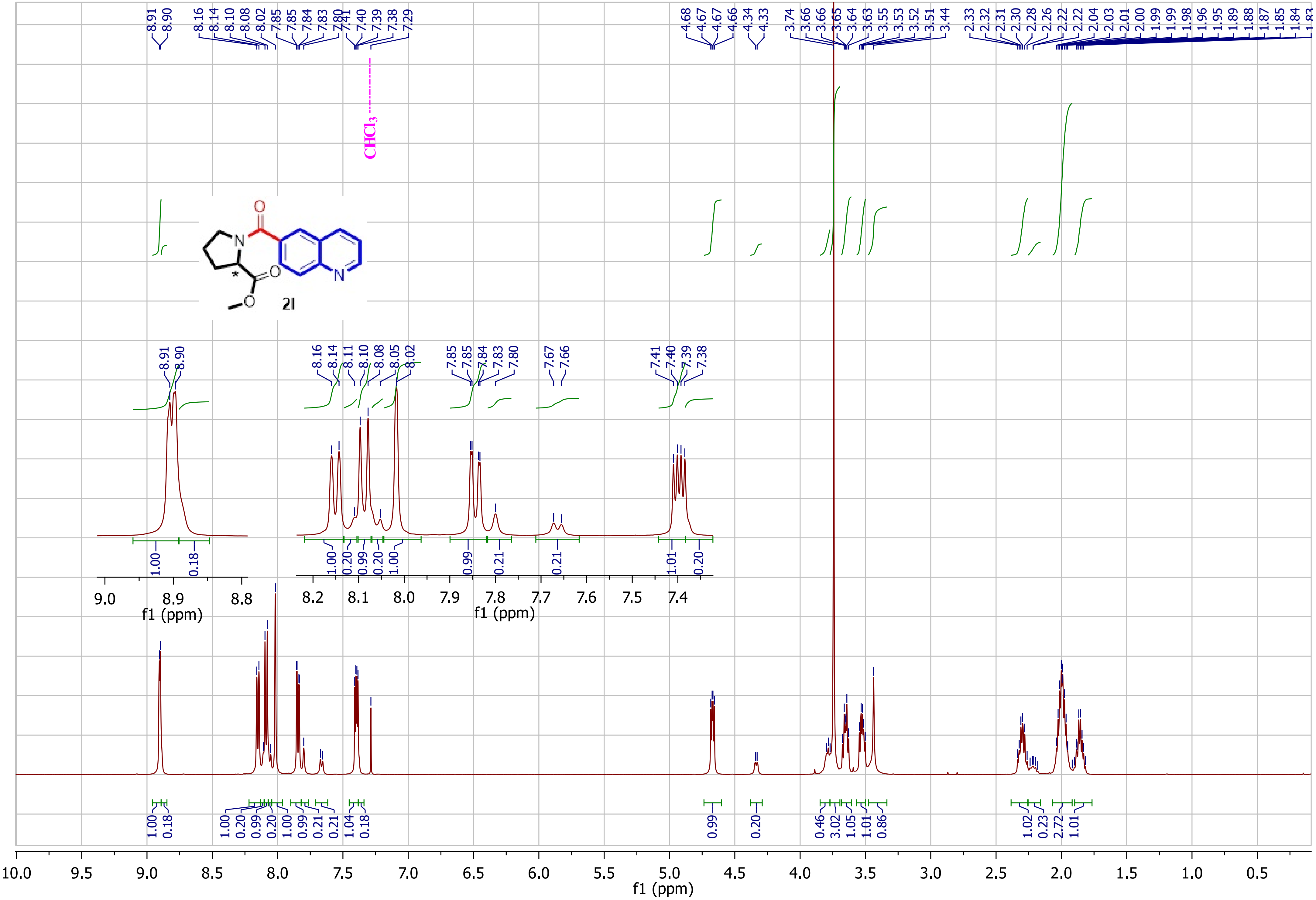
8.91
8.90
8.16
8.14
8.10
8.08
8.02
7.85
7.85
7.84
7.83
7.80
7.41
7.40
7.39
7.38
7.29
4.68
4.67
4.67
4.66
4.34
4.33
3.74
3.66
3.66
3.65
3.64
3.63
3.55
3.53
3.52
3.51
3.44
2.33
2.32
2.31
2.30
2.28
2.26
2.22
2.22
2.04
2.03
2.01
2.00
1.99
1.99
1.98
1.96
1.95
1.89
1.88
1.87
1.85
1.84
1.83
CHCl3
O
N
O
O
N
*
2l
1.00
0.18
1.00
0.20
0.99
0.20
1.00
0.99
0.21
0.21
1.04
0.18
0.99
0.20
0.46
3.02
1.05
1.01
0.86
1.02
0.23
2.72
1.01
10.0
9.5
9.0
8.5
8.0
7.5
7.0
6.5
6.0
5.5
5.0
4.5
4.0
3.5
3.0
2.5
2.0
1.5
1.0
0.5
f1 (ppm)
8.91
8.90
1.00
0.18
9.0
8.9
8.8
f1 (ppm)
8.16
8.14
8.11
8.10
8.08
8.05
8.02
7.85
7.85
7.84
7.83
7.80
7.67
7.66
7.41
7.40
7.39
7.38
1.00
0.20
0.99
0.20
1.00
0.99
0.21
0.21
1.01
0.20
8.2
8.1
8.0
7.9
7.8
7.7
7.6
7.5
7.4
f1 (ppm)

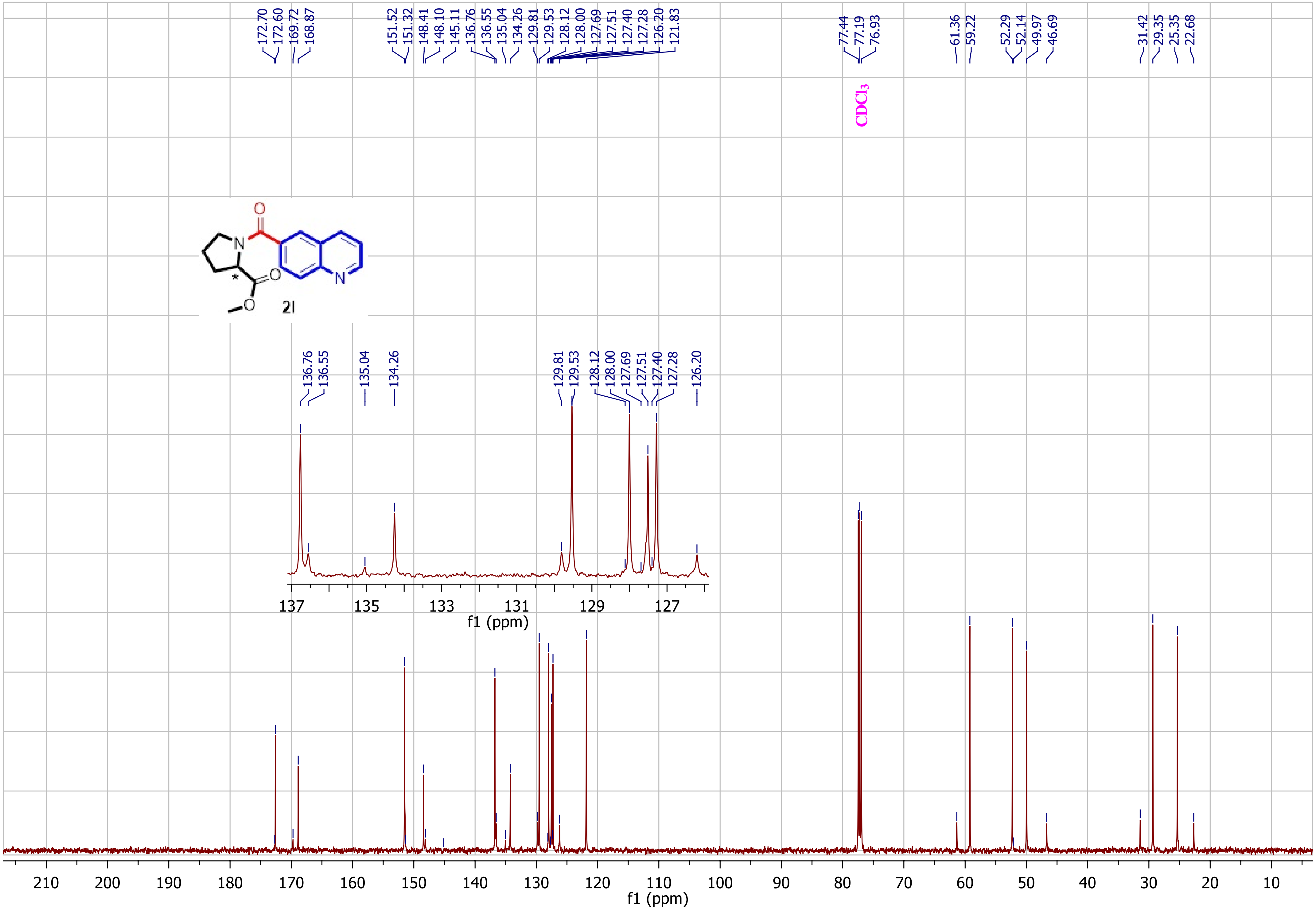
172.70
172.60
169.72
168.87
151.52
151.32
148.41
148.10
145.11
136.76
136.55
135.04
134.26
129.81
129.53
128.12
128.00
127.69
127.51
127.40
127.28
126.20
121.83
77.44
77.19
76.93
61.36
59.22
52.29
52.14
49.97
46.69
31.42
29.35
25.35
22.68
CDCl3
O
N
O
O
N
*
2l
137
135
133
131
129
127
f1 (ppm)
210
200
190
180
170
160
150
140
130
120
110
100
90
80
70
60
50
40
30
20
10
f1 (ppm)

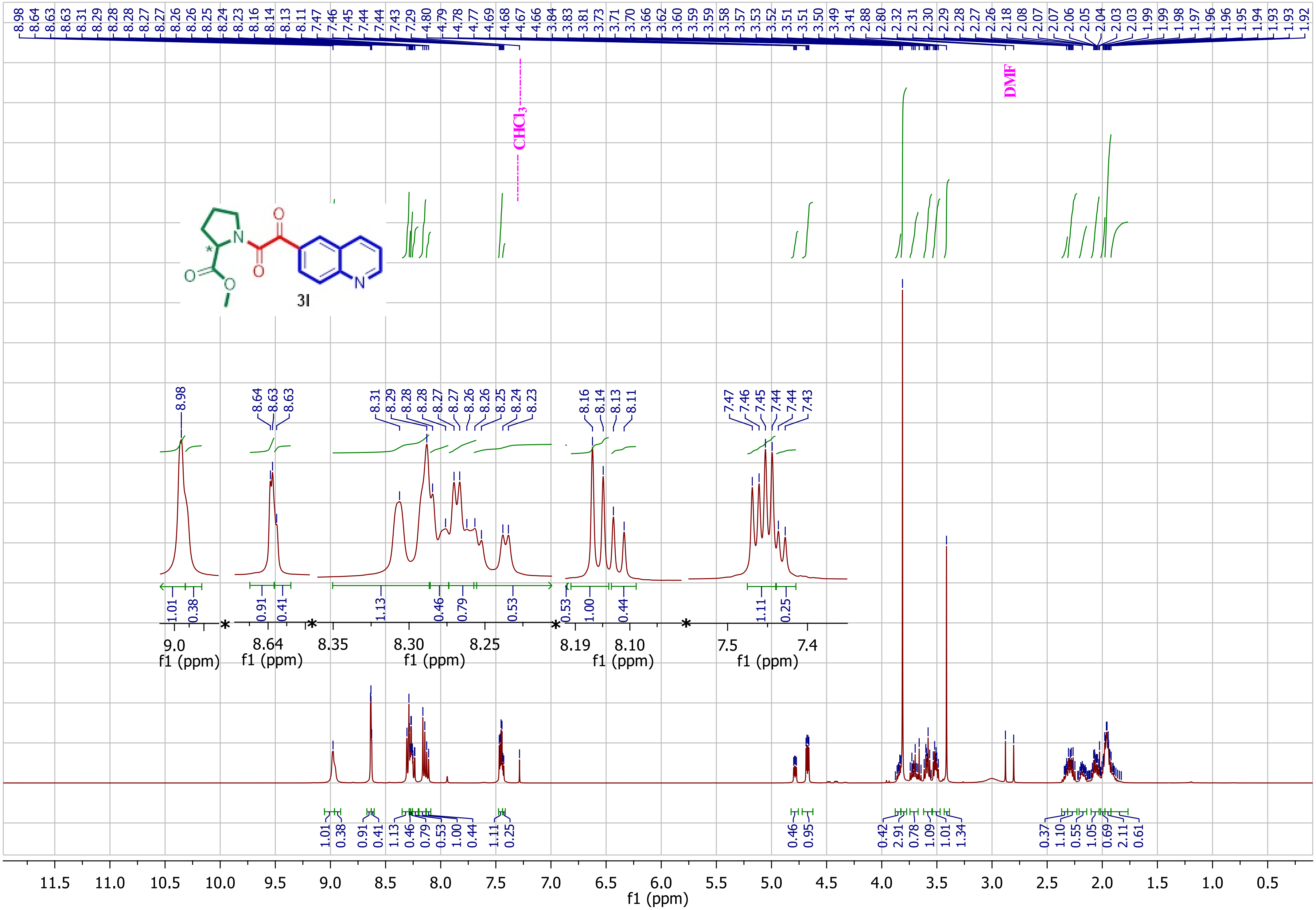
CHCl3
DMF
3l
f1 (ppm)

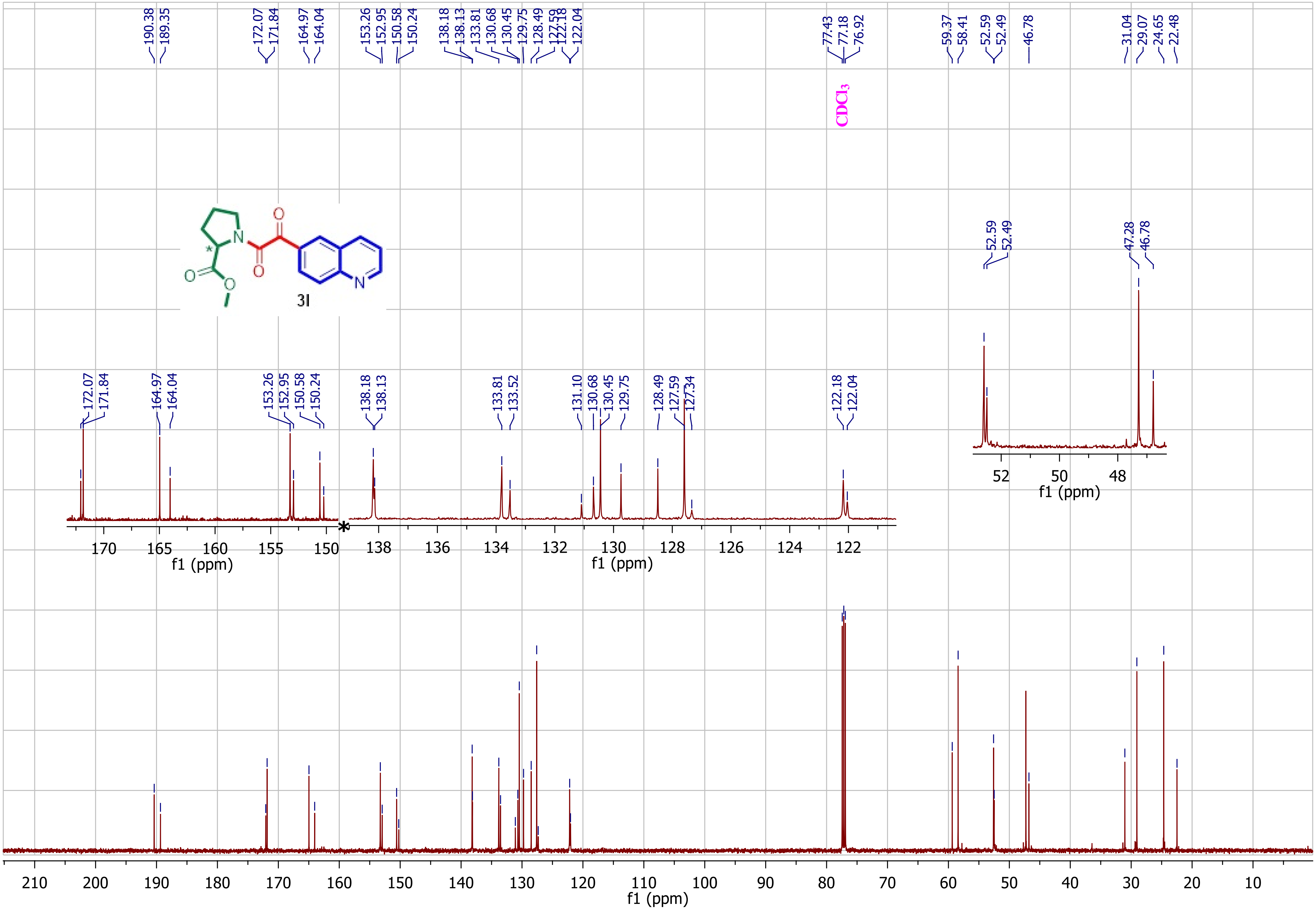
190.38
189.35
172.07
171.84
164.97
164.04
153.26
152.95
150.58
150.24
138.18
138.13
133.81
130.68
130.45
129.75
128.49
127.59
122.18
122.04
77.43
77.18
76.92
59.37
58.41
52.59
52.49
46.78
31.04
29.07
24.65
22.48
CDCl3
133.52
131.10
127.34
47.28
f1 (ppm)
3l

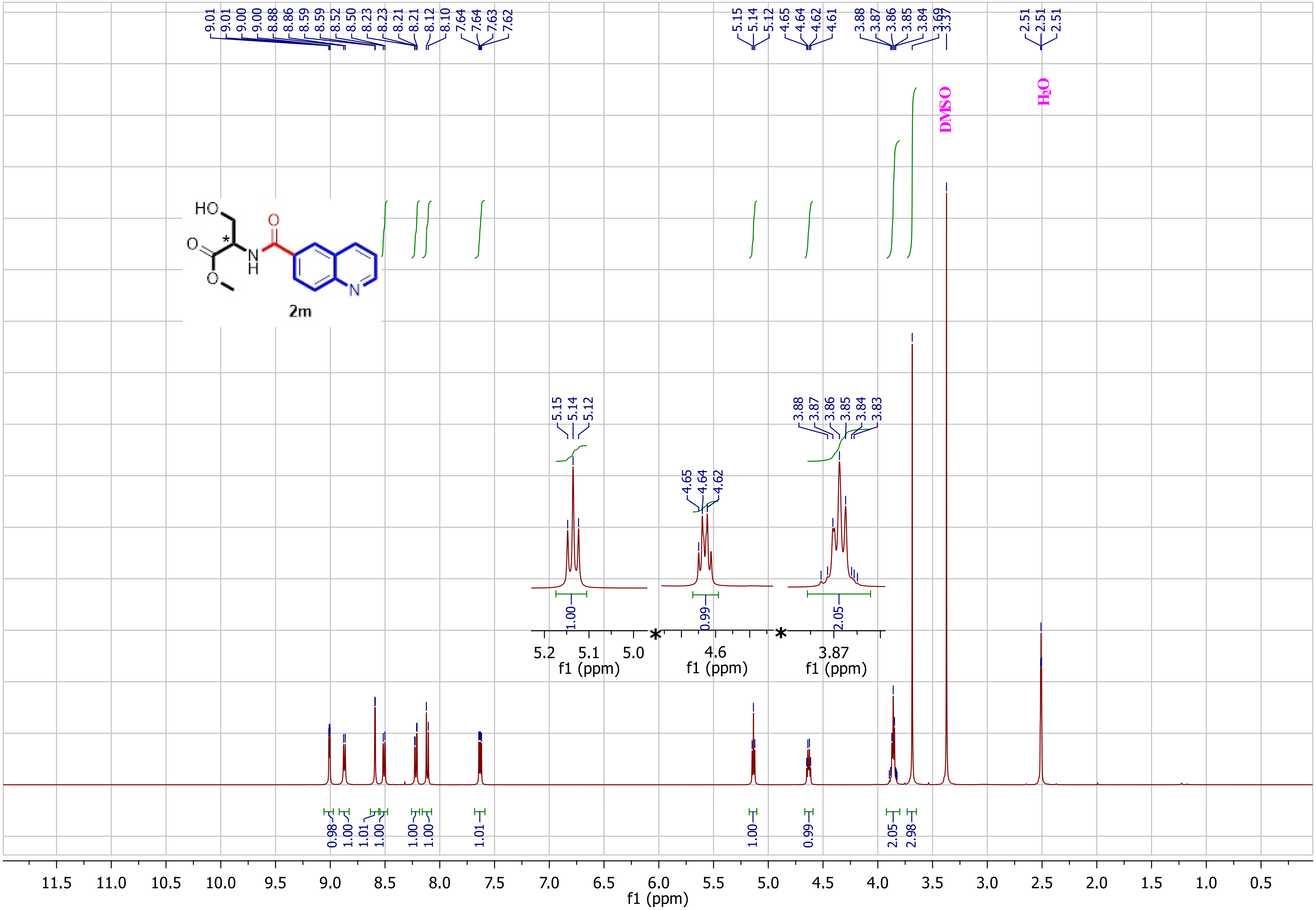
9.01 9.01 9.00 9.00 8.88 8.86 8.59 8.59 8.52 8.50 8.23 8.23 8.21 8.21 8.12 8.10 7.64 7.64 7.63 7.62
5.15 5.14 5.12 4.65 4.64 4.62 4.61 3.88 3.87 3.86 3.85 3.84 3.69 3.37
2.51 2.51 2.51
DMSO
H_2O
HO
O
O
N
H
O
N
*
2m
5.15 5.14 5.12
4.65 4.64 4.62
3.88 3.87 3.86 3.85 3.84 3.83
1.00
0.99
2.05
5.2 5.1 5.0
4.6
3.87
f1 (ppm)
0.98 1.00 1.01 1.00 1.00 1.00 1.01 1.00 0.99 2.05 2.98
11.5 11.0 10.5 10.0 9.5 9.0 8.5 8.0 7.5 7.0 6.5 6.0 5.5 5.0 4.5 4.0 3.5 3.0 2.5 2.0 1.5 1.0 0.5
f1 (ppm)

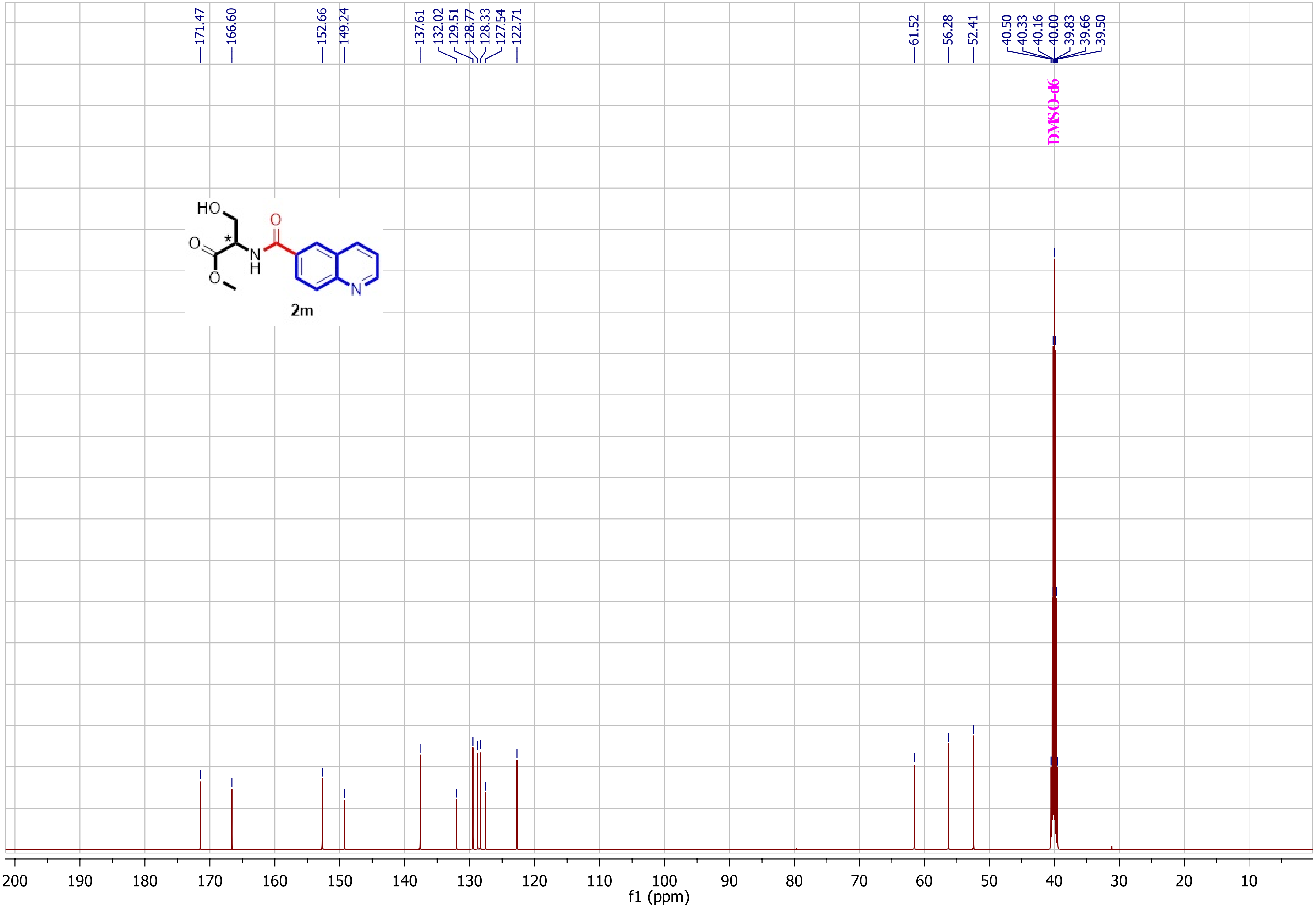
171.47
166.60
152.66
149.24
137.61
132.02
129.51
128.77
128.33
127.54
122.71
61.52
56.28
52.41
40.50
40.33
40.16
40.00
39.83
39.66
39.50
DMSO-d6
HO
O
O
N
H
N
O
*
2m
200 190 180 170 160 150 140 130 120 110 100 90 80 70 60 50 40 30 20 10
f1 (ppm)

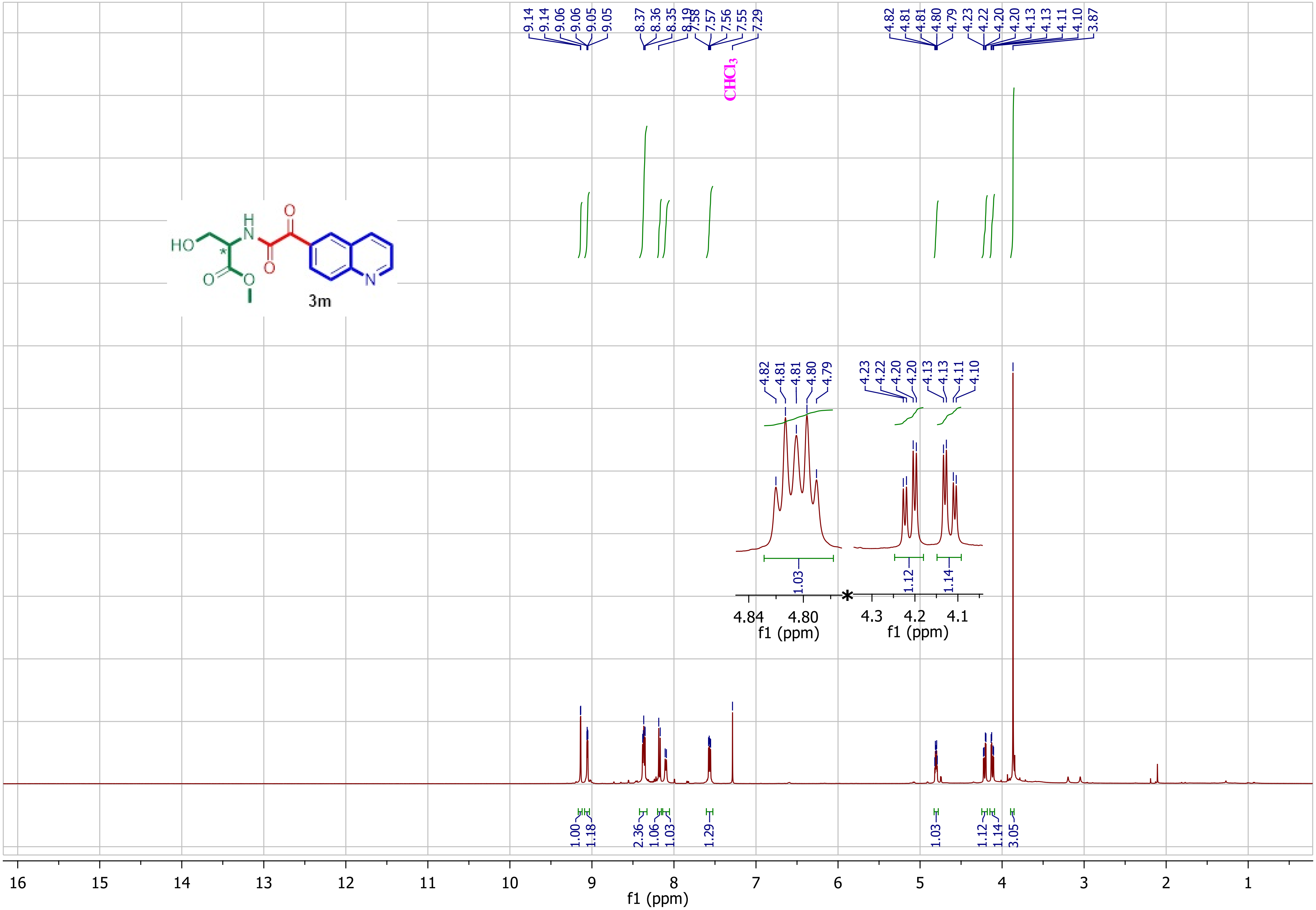

9.14 9.14 9.06 9.06 9.05 9.05 8.37 8.36 8.35 8.19 7.58 7.57 7.56 7.55 7.29 4.82 4.81 4.81 4.80 4.79 4.23 4.22 4.20 4.20 4.13 4.13 4.11 4.10 3.87
CHCl3
HO
H
N
O
O
O
O
N
3m
4.82 4.81 4.81 4.80 4.79
4.23 4.22 4.20 4.20 4.13 4.13 4.11 4.10
1.03
1.12
1.14
4.84 4.80
f1 (ppm)
4.3 4.2 4.1
f1 (ppm)
1.00 1.18 2.36 1.06 1.03 1.29 1.03 1.12 1.14 3.05
16 15 14 13 12 11 10 9 8 7 6 5 4 3 2 1
f1 (ppm)

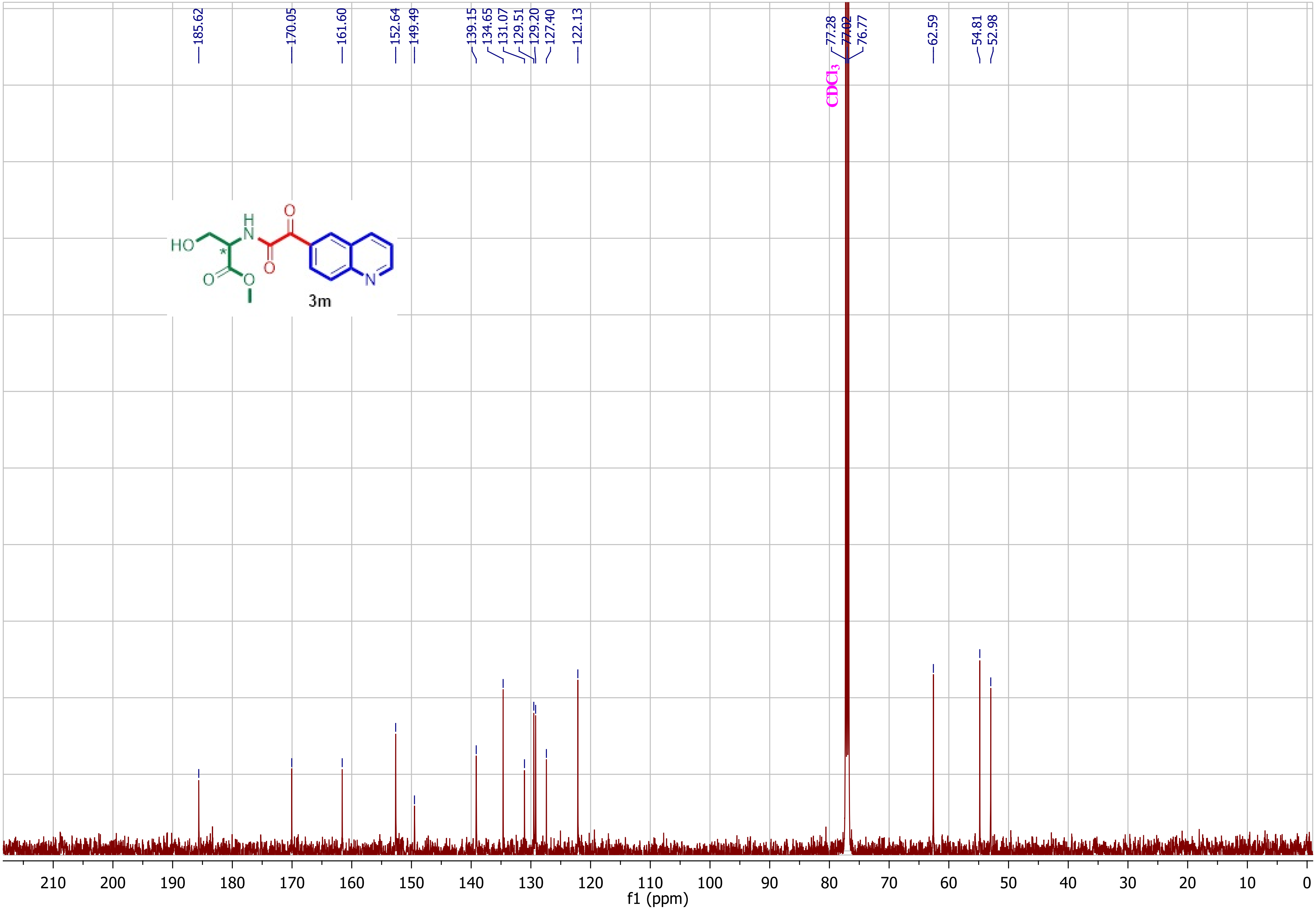
185.62
170.05
161.60
152.64
149.49
139.15
134.65
131.07
129.51
129.20
127.40
122.13
77.28
77.02
76.77
62.59
54.81
52.98
CDCl3
HO
H
N
O
O
O
O
N
*
3m
210 200 190 180 170 160 150 140 130 120 110 100 90 80 70 60 50 40 30 20 10 0
f1 (ppm)

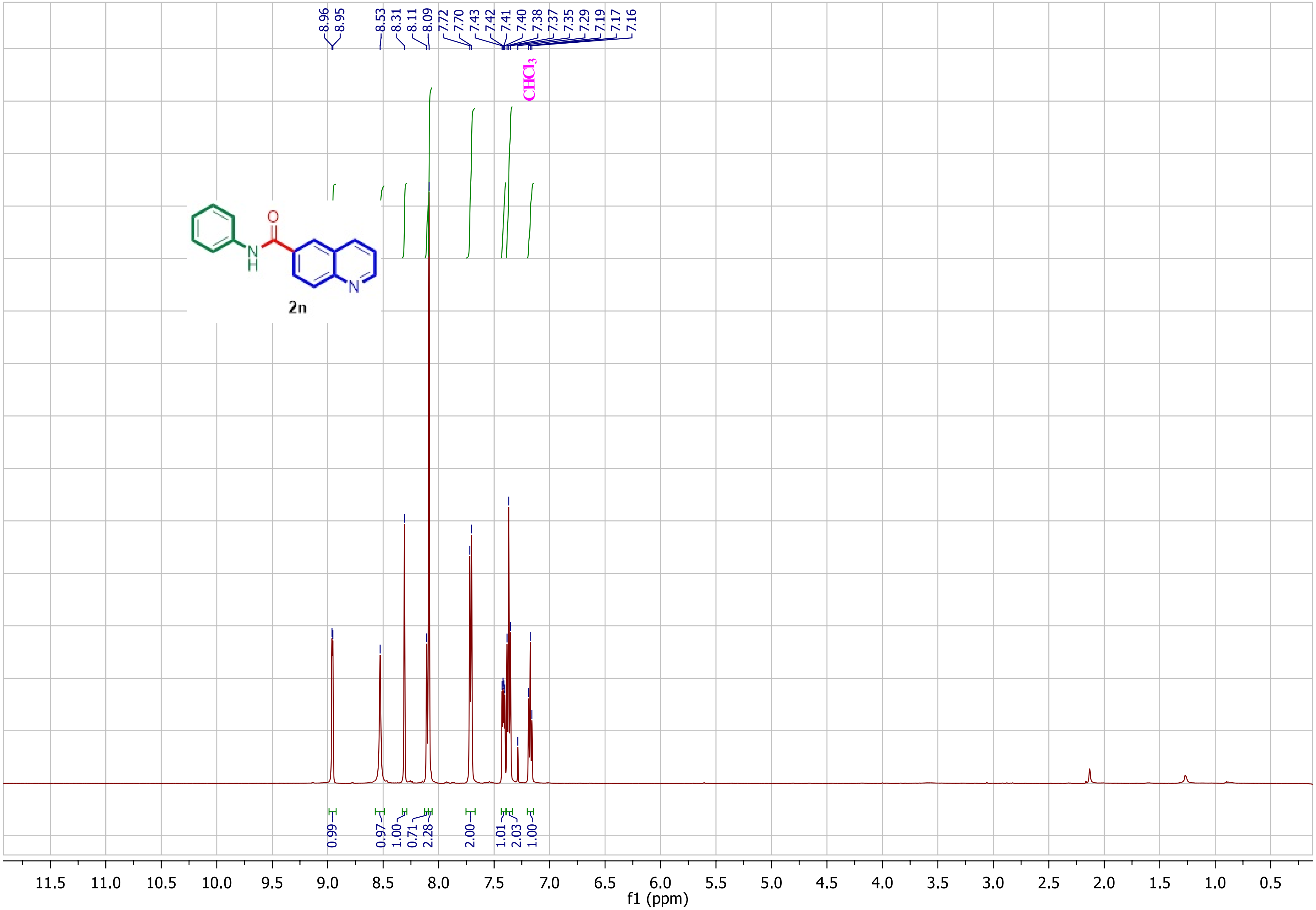
8.96
8.95
8.53
8.31
8.11
8.09
7.72
7.70
7.43
7.42
7.41
7.40
7.38
7.37
7.35
7.29
7.19
7.17
7.16
CHCl3
O
N
H
N
2n
0.99
0.97
1.00
0.71
2.28
2.00
1.01
2.03
1.00
11.5
11.0
10.5
10.0
9.5
9.0
8.5
8.0
7.5
7.0
6.5
6.0
5.5
5.0
4.5
4.0
3.5
3.0
2.5
2.0
1.5
1.0
0.5
f1 (ppm)

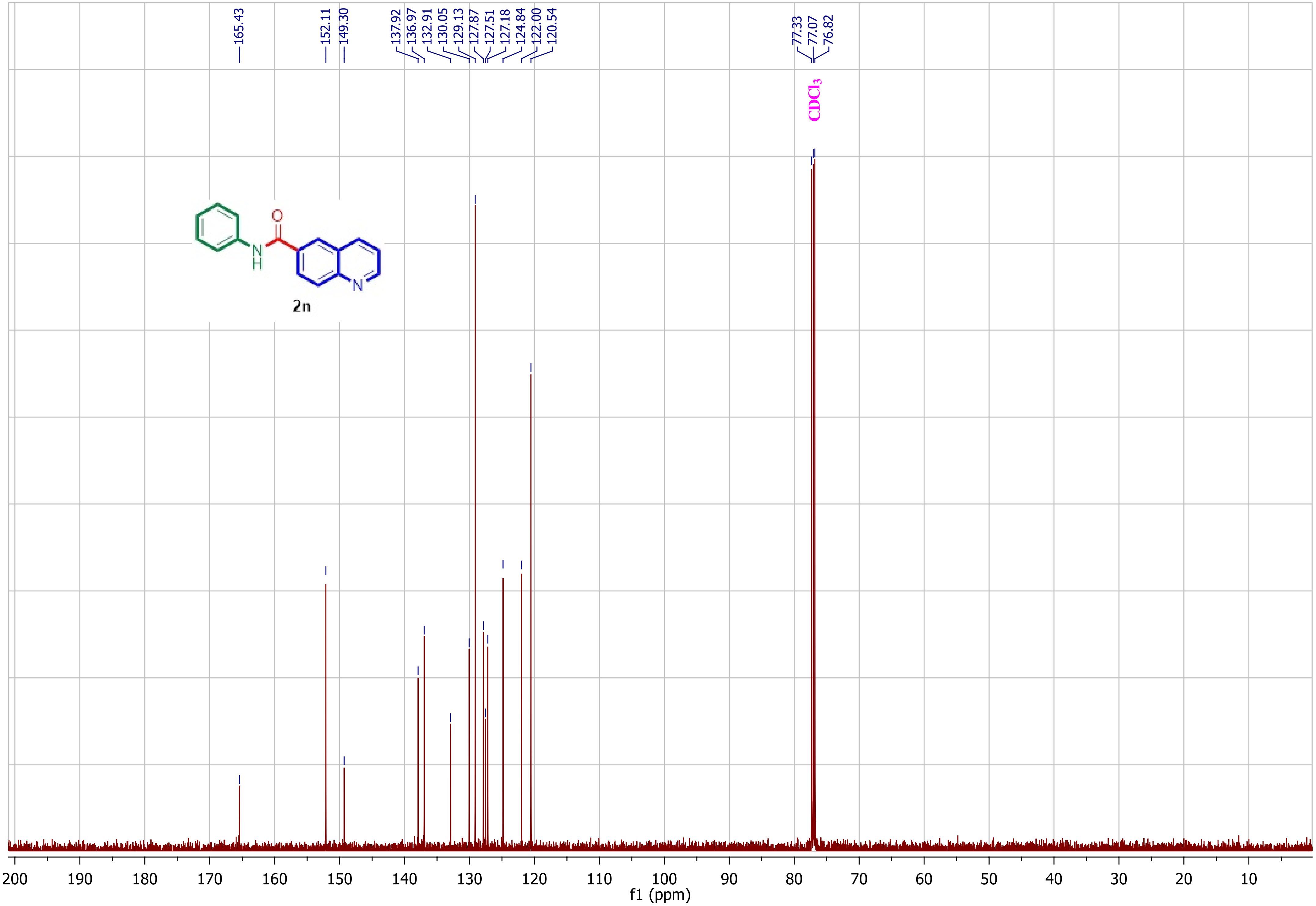

165.43
152.11
149.30
137.92
136.97
132.91
130.05
129.13
127.87
127.51
127.18
124.84
122.00
120.54
77.33
77.07
76.82
CDCl3
O
N
H
N
2n
200 190 180 170 160 150 140 130 120 110 100 90 80 70 60 50 40 30 20 10
f1 (ppm)

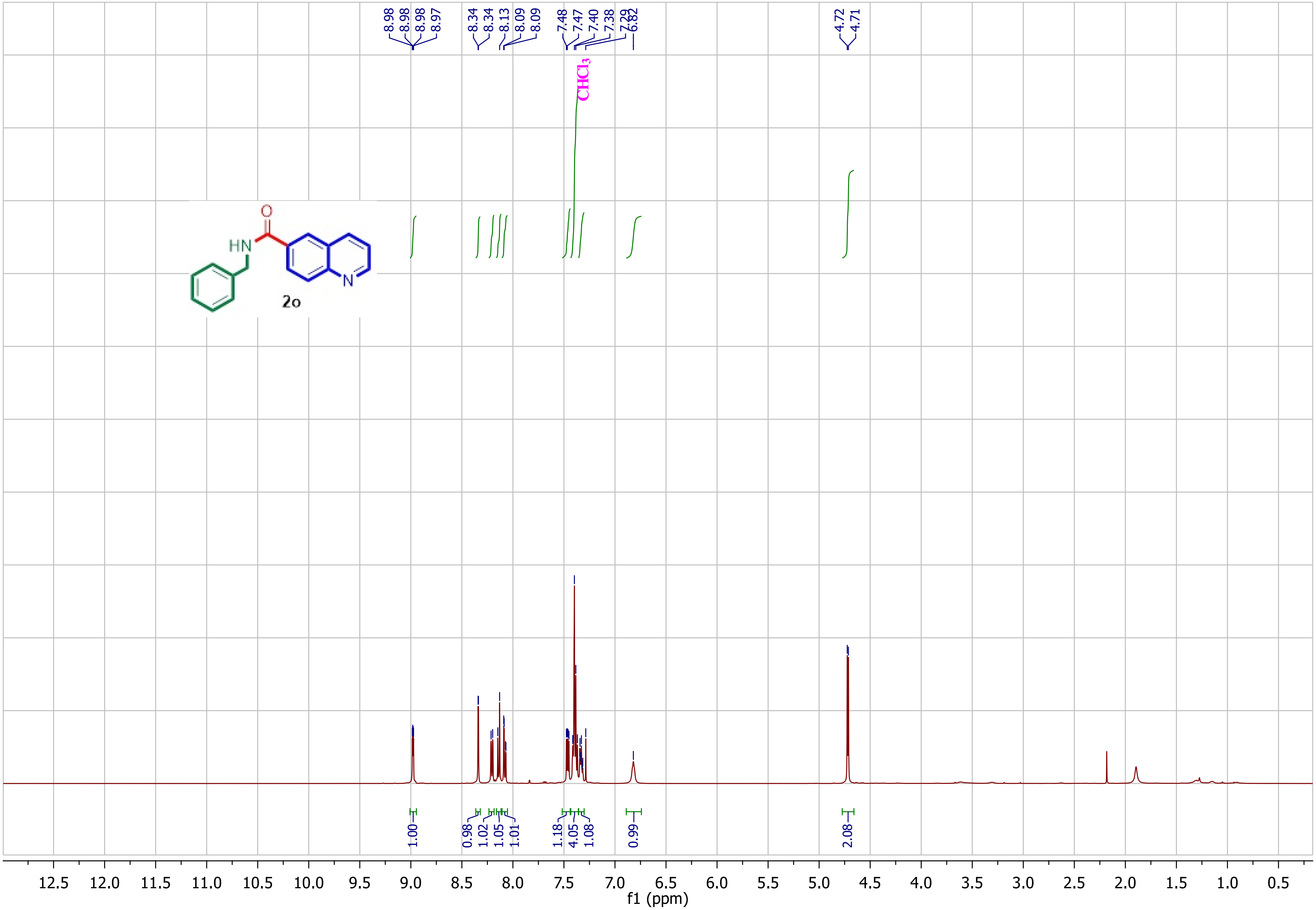
8.98
8.98
8.98
8.97
8.34
8.34
8.13
8.09
8.09
7.48
7.47
7.40
7.38
7.29
6.82
4.72
4.71
CHCl3
HN
O
N
2o
1.00
0.98
1.02
1.05
1.01
1.18
4.05
1.08
0.99
2.08
12.5
12.0
11.5
11.0
10.5
10.0
9.5
9.0
8.5
8.0
7.5
7.0
6.5
6.0
5.5
5.0
4.5
4.0
3.5
3.0
2.5
2.0
1.5
1.0
0.5
f1 (ppm)

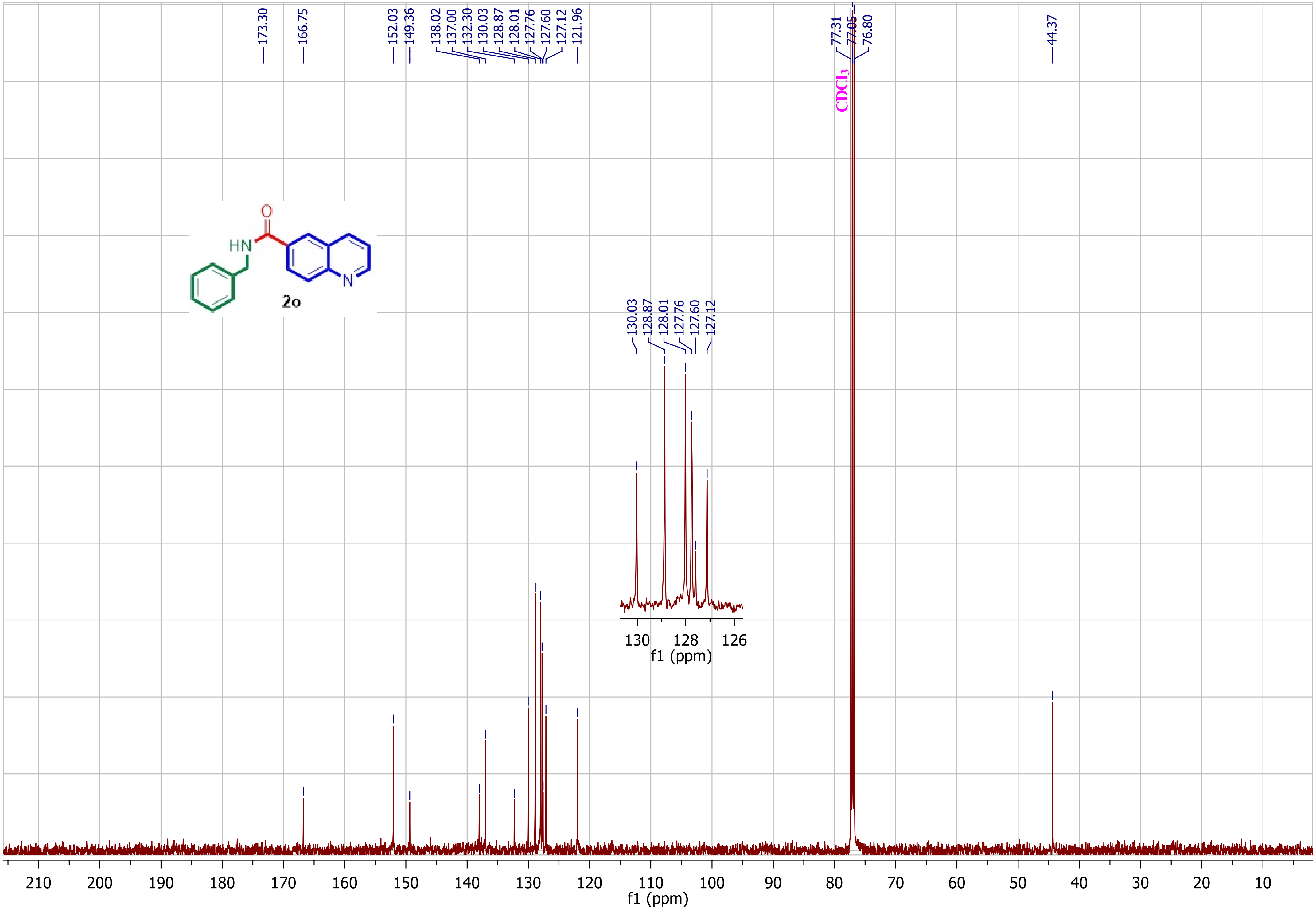

173.30
166.75
152.03
149.36
138.02
137.00
132.30
130.03
128.87
128.01
127.76
127.60
127.12
121.96
77.31
77.05
76.80
44.37
CDCl3
O
HN
N
2o
130.03
128.87
128.01
127.76
127.60
127.12
130
128
126
f1 (ppm)
210
200
190
180
170
160
150
140
130
120
110
100
90
80
70
60
50
40
30
20
10
f1 (ppm)

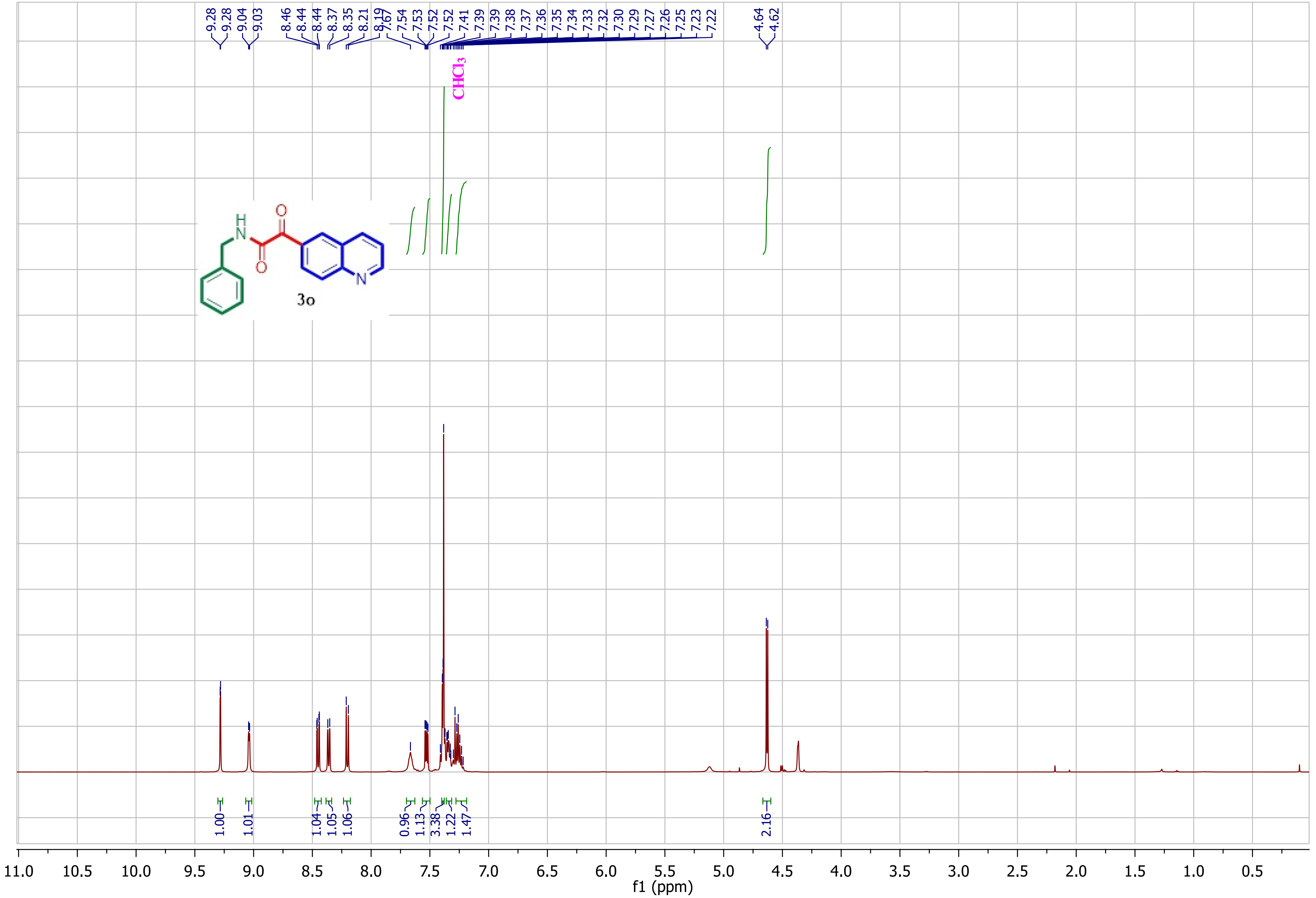
9.28
9.28
9.04
9.03
8.46
8.44
8.44
8.37
8.35
8.21
8.19
7.67
7.54
7.53
7.52
7.52
7.41
7.39
7.39
7.38
7.37
7.36
7.35
7.34
7.33
7.32
7.30
7.29
7.27
7.26
7.25
7.23
7.22
4.64
4.62
CHCl3
3o
1.00
1.01
1.04
1.05
1.06
0.96
1.13
3.38
1.22
1.47
2.16
11.0
10.5
10.0
9.5
9.0
8.5
8.0
7.5
7.0
6.5
6.0
5.5
5.0
4.5
4.0
3.5
3.0
2.5
2.0
1.5
1.0
0.5
f1 (ppm)

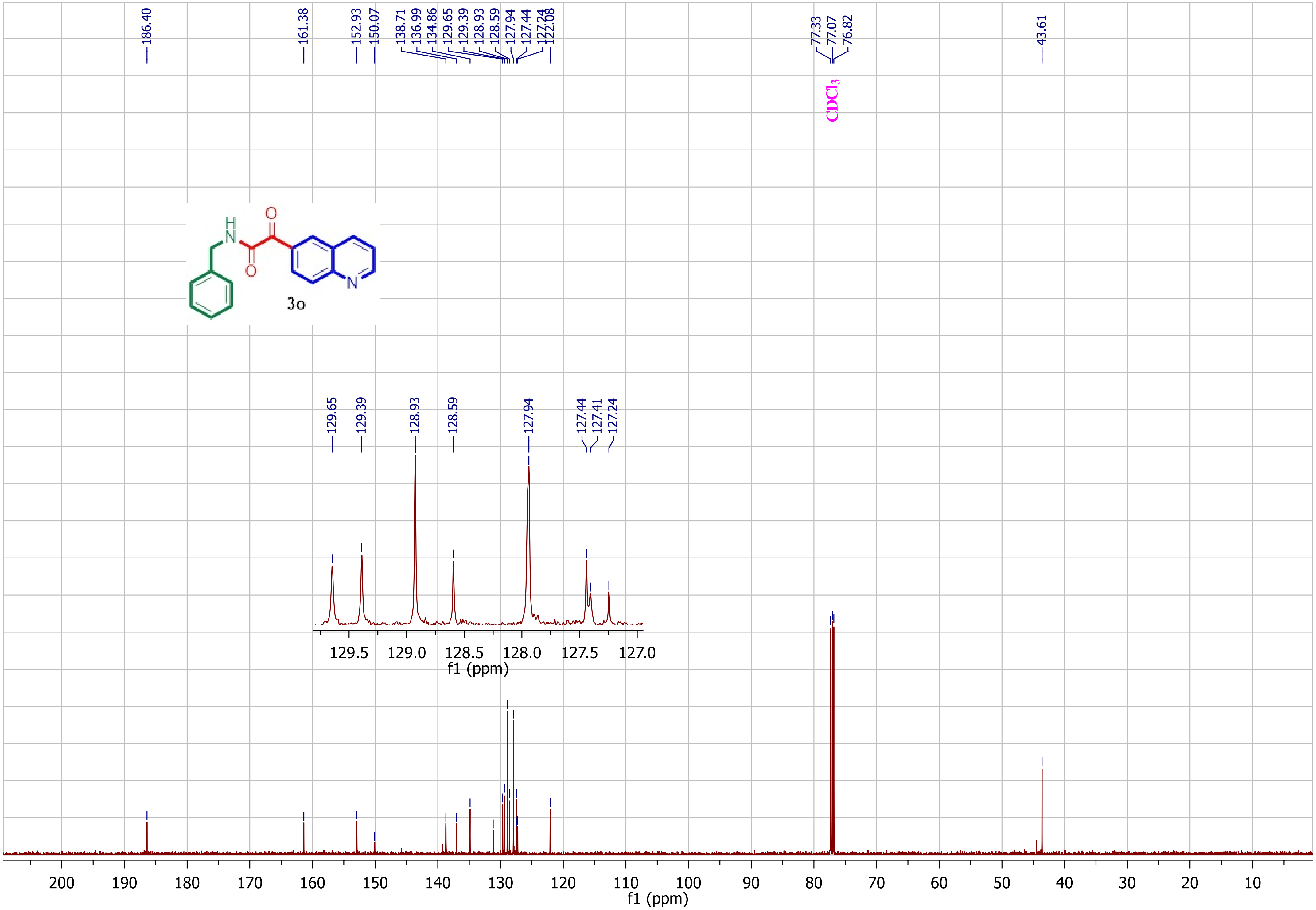
186.40
161.38
152.93
150.07
138.71
136.99
134.86
129.65
129.39
128.93
128.59
127.94
127.44
127.24
122.08
77.33
77.07
76.82
43.61
CDCl3
H
N
O
O
N
3o
129.65
129.39
128.93
128.59
127.94
127.44
127.41
127.24
129.5
129.0
128.5
128.0
127.5
127.0
f1 (ppm)
200
190
180
170
160
150
140
130
120
110
100
90
80
70
60
50
40
30
20
10
f1 (ppm)

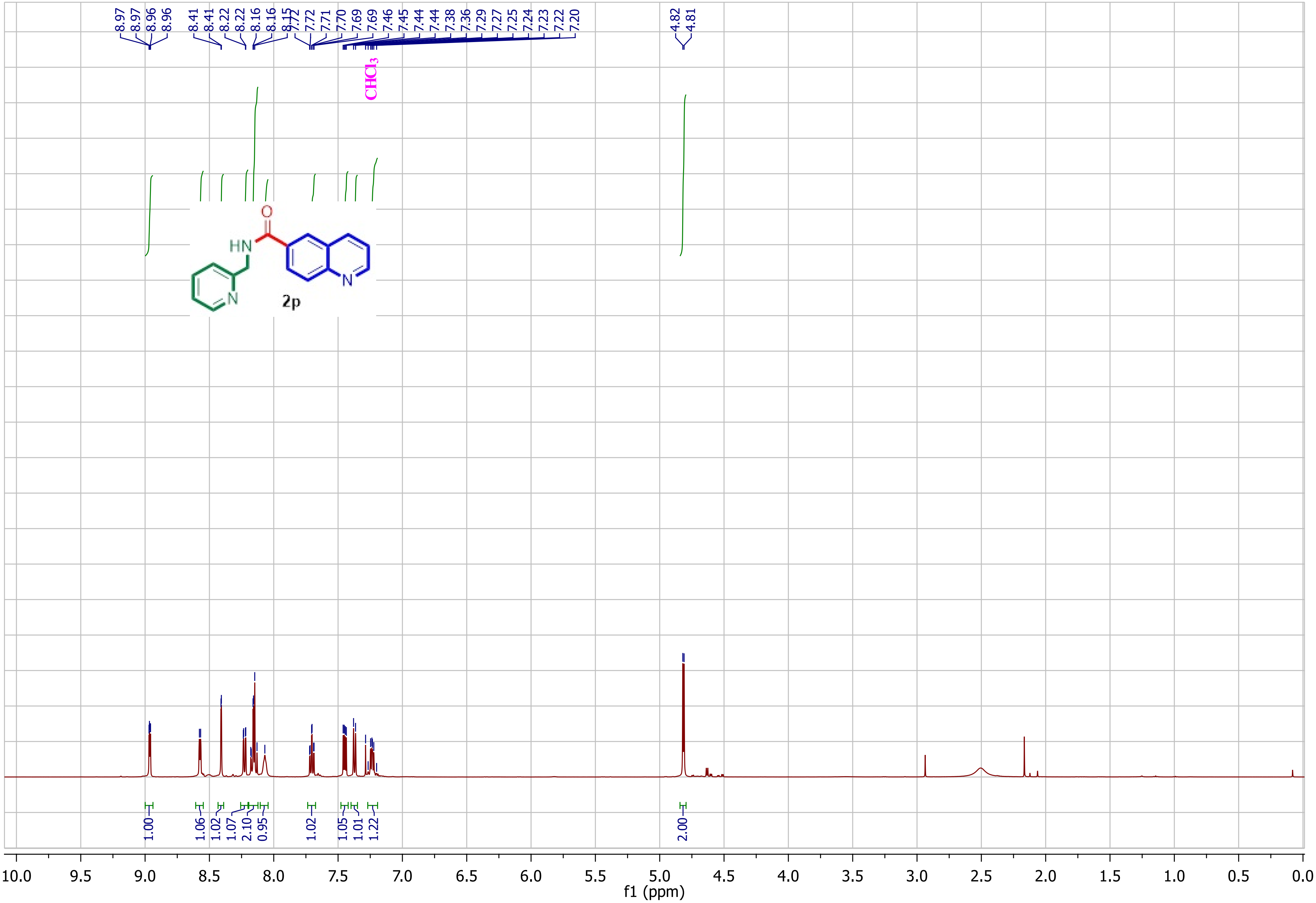
8.97
8.97
8.96
8.96
8.41
8.41
8.22
8.22
8.16
8.16
8.15
7.72
7.72
7.71
7.70
7.69
7.69
7.46
7.45
7.44
7.44
7.38
7.36
7.29
7.27
7.25
7.24
7.23
7.22
7.20
4.82
4.81
CHCl3
O
HN
N
N
2p
1.00
1.06
1.02
1.07
2.10
0.95
1.02
1.05
1.01
1.22
2.00
10.0
9.5
9.0
8.5
8.0
7.5
7.0
6.5
6.0
5.5
5.0
4.5
4.0
3.5
3.0
2.5
2.0
1.5
1.0
0.5
0.0
f1 (ppm)

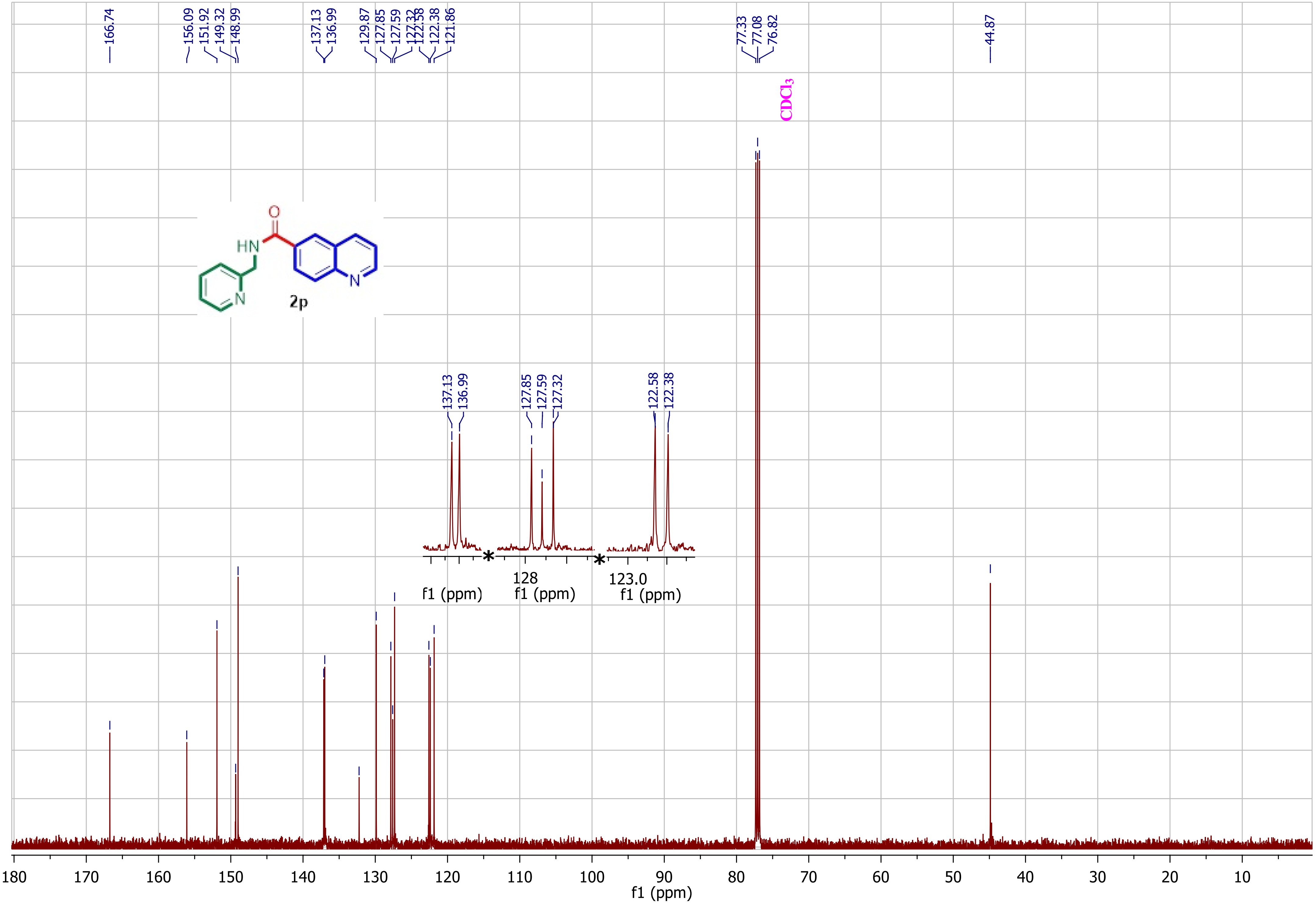

166.74
156.09
151.92
149.32
148.99
137.13
136.99
129.87
127.85
127.59
127.32
122.58
122.38
121.86
77.33
77.08
76.82
44.87
CDCl3
O
HN
N
N
2p
137.13
136.99
127.85
127.59
127.32
122.58
122.38
128
123.0
f1 (ppm)
f1 (ppm)
f1 (ppm)
180 170 160 150 140 130 120 110 100 90 80 70 60 50 40 30 20 10
f1 (ppm)

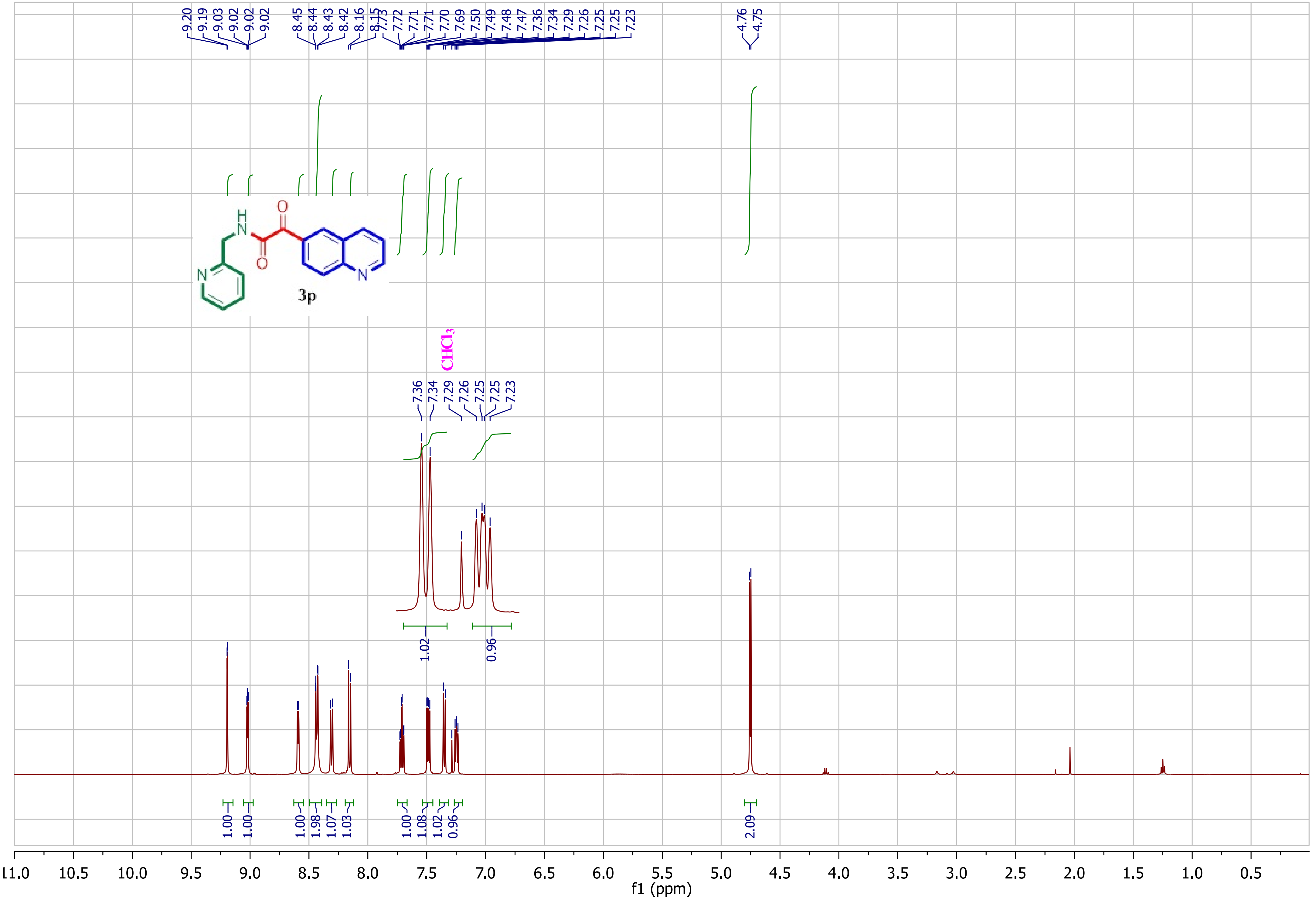
9.20 9.19 9.03 9.02 9.02 9.02 8.45 8.44 8.43 8.42 8.16 8.15 7.73 7.72 7.71 7.71 7.70 7.69 7.50 7.49 7.48 7.47 7.36 7.34 7.29 7.26 7.25 7.25 7.23
4.76 4.75
3p
CHCl3
7.36 7.34 7.29 7.26 7.25 7.25 7.23
1.02
0.96
1.00 1.00 1.00 1.98 1.07 1.03 1.00 1.08 1.02 0.96 2.09
11.0 10.5 10.0 9.5 9.0 8.5 8.0 7.5 7.0 6.5 6.0 5.5 5.0 4.5 4.0 3.5 3.0 2.5 2.0 1.5 1.0 0.5
f1 (ppm)

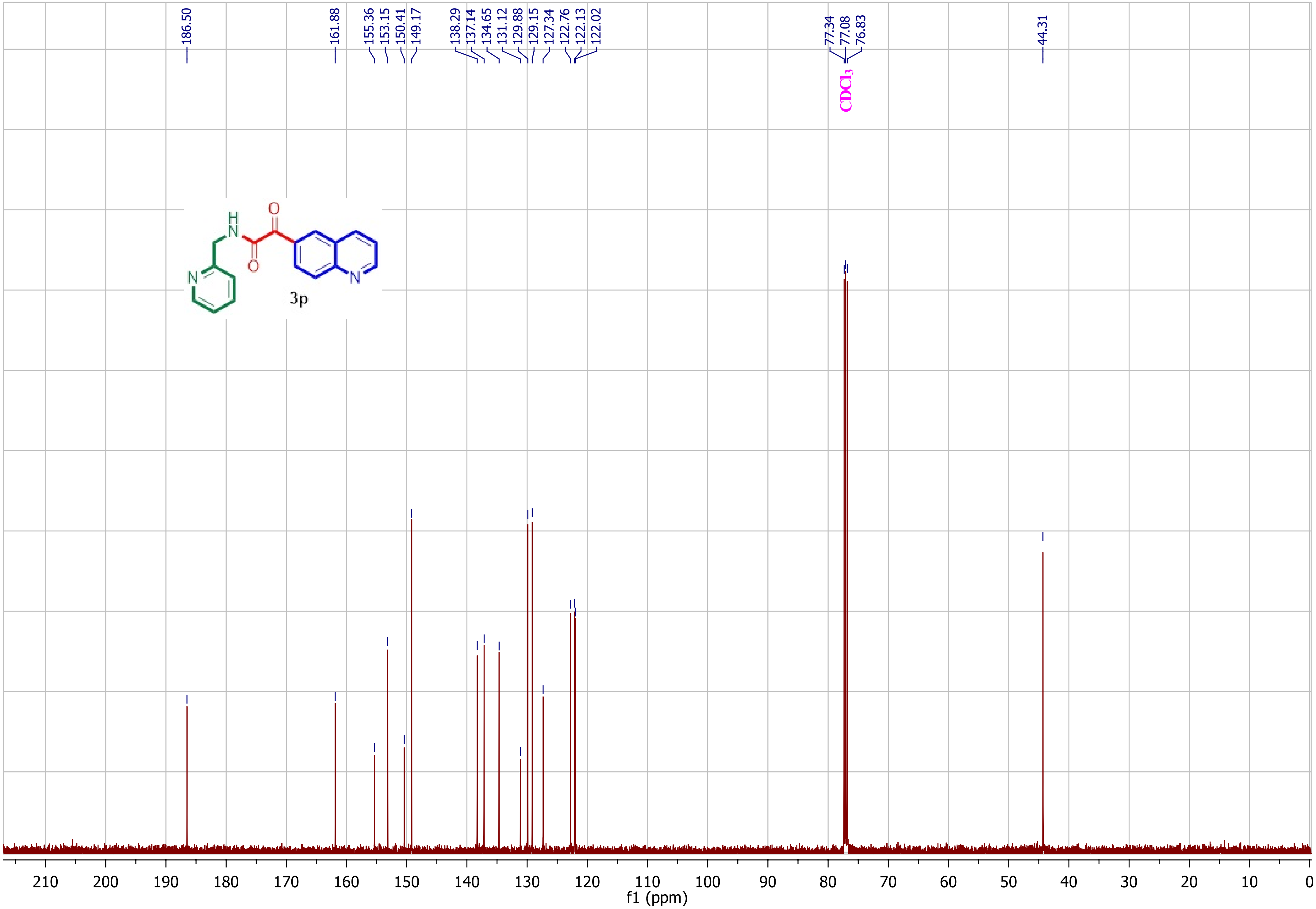

186.50
161.88
155.36
153.15
150.41
149.17
138.29
137.14
134.65
131.12
129.88
129.15
127.34
122.76
122.13
122.02
77.34
77.08
76.83
44.31
CDCl3
3p
210 200 190 180 170 160 150 140 130 120 110 100 90 80 70 60 50 40 30 20 10 0
f1 (ppm)

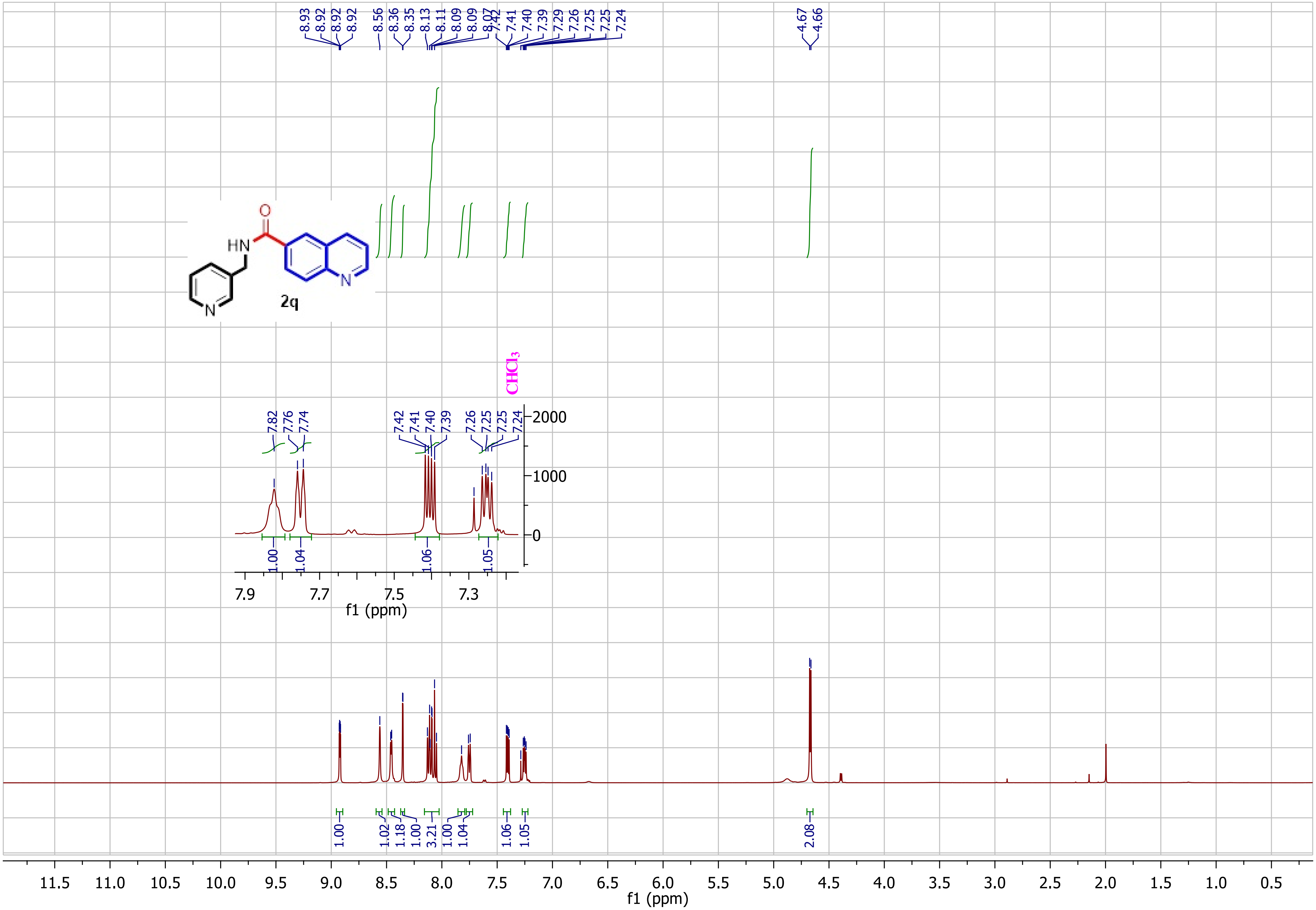
8.93
8.92
8.92
8.92
8.56
8.36
8.35
8.13
8.11
8.09
8.09
8.07
7.42
7.41
7.40
7.39
7.29
7.26
7.25
7.25
7.24
4.67
4.66
O
HN
N
N
2q
CHCl3
7.82
7.76
7.74
7.42
7.41
7.40
7.39
7.26
7.25
7.25
7.24
2000
1000
0
1.00
1.04
1.06
1.05
7.9
7.7
7.5
7.3
f1 (ppm)
1.00
1.02
1.18
1.00
3.21
1.00
1.04
1.06
1.05
2.08
11.5
11.0
10.5
10.0
9.5
9.0
8.5
8.0
7.5
7.0
6.5
6.0
5.5
5.0
4.5
4.0
3.5
3.0
2.5
2.0
1.5
1.0
0.5
f1 (ppm)

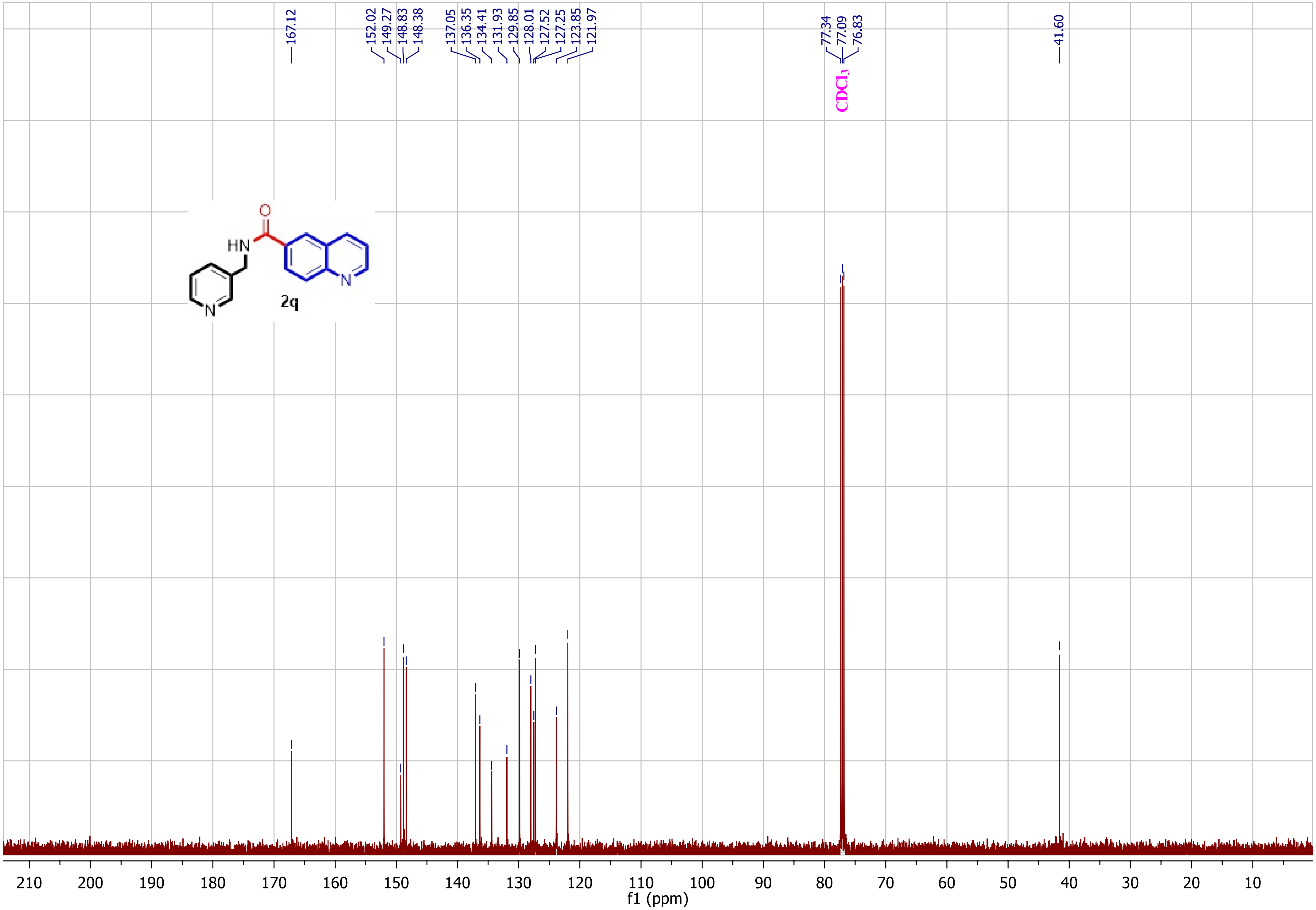

167.12
152.02
149.27
148.83
148.38
137.05
136.35
134.41
131.93
129.85
128.01
127.52
127.25
123.85
121.97
77.34
77.09
76.83
41.60
CDCl3
O
HN
N
N
2q
210 200 190 180 170 160 150 140 130 120 110 100 90 80 70 60 50 40 30 20 10
f1 (ppm)

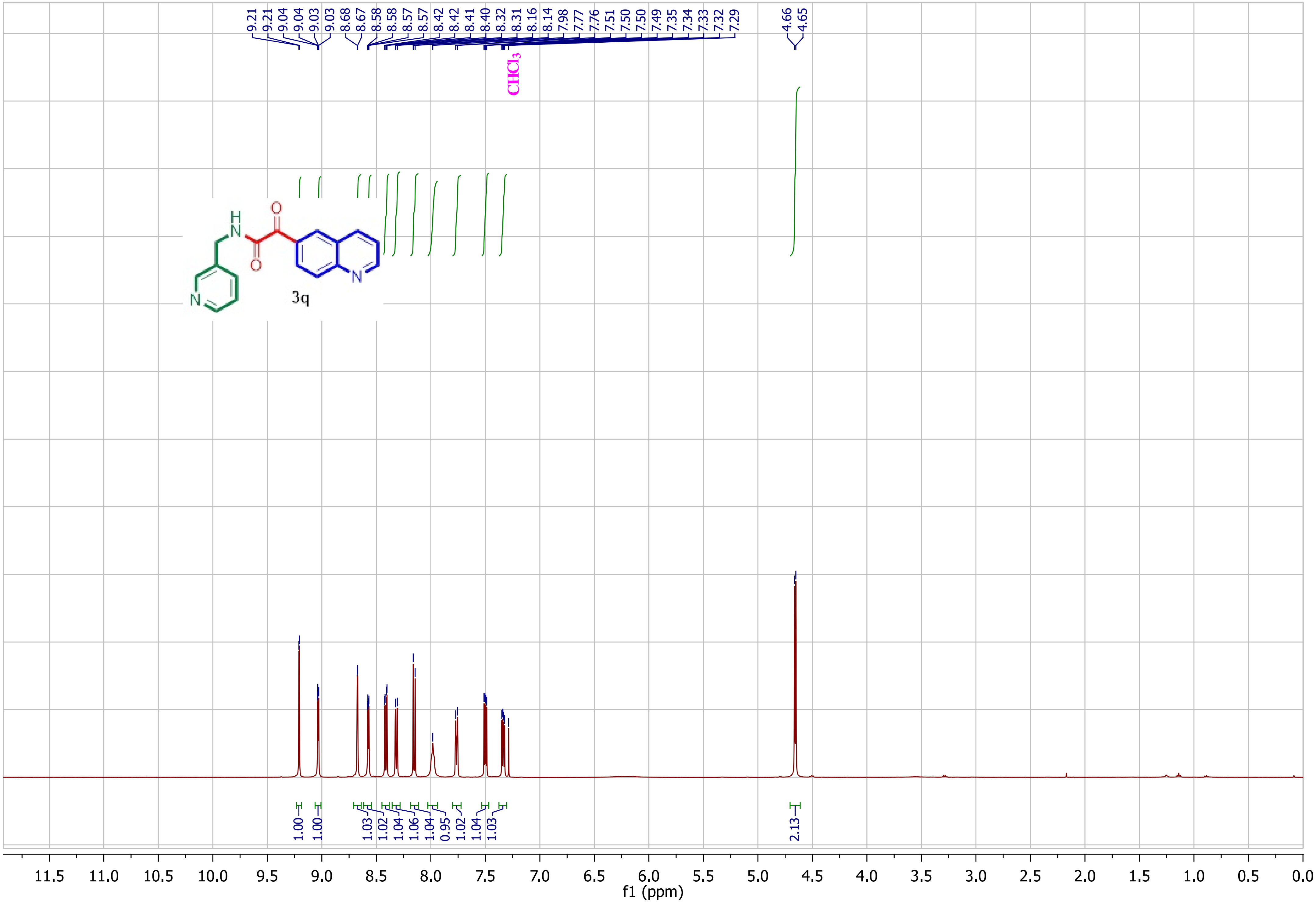
9.21
9.21
9.04
9.04
9.03
9.03
8.68
8.67
8.58
8.58
8.57
8.57
8.42
8.42
8.41
8.40
8.32
8.31
8.16
8.14
7.98
7.77
7.76
7.51
7.50
7.50
7.49
7.35
7.34
7.33
7.32
7.29
4.66
4.65
CHCl3
3q
1.00
1.00
1.03
1.02
1.04
1.06
1.04
0.95
1.02
1.04
1.03
2.13
11.5
11.0
10.5
10.0
9.5
9.0
8.5
8.0
7.5
7.0
6.5
6.0
5.5
5.0
4.5
4.0
3.5
3.0
2.5
2.0
1.5
1.0
0.5
0.0
f1 (ppm)

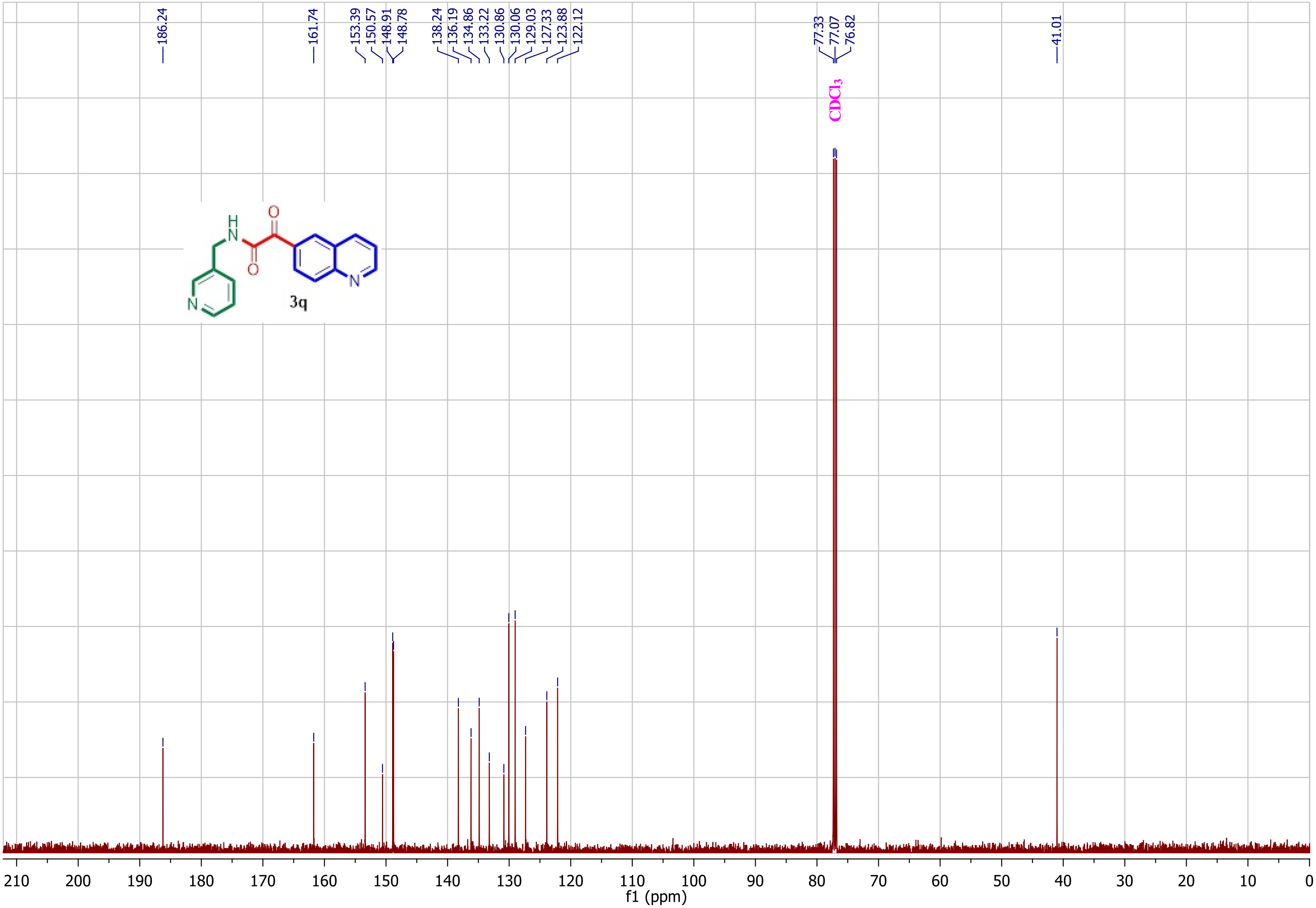
186.24
161.74
153.39
150.57
148.91
148.78
138.24
136.19
134.86
133.22
130.86
130.06
129.03
127.33
123.88
122.12
77.33
77.07
76.82
41.01
CDCl3
3q
210 200 190 180 170 160 150 140 130 120 110 100 90 80 70 60 50 40 30 20 10 0
f1 (ppm)

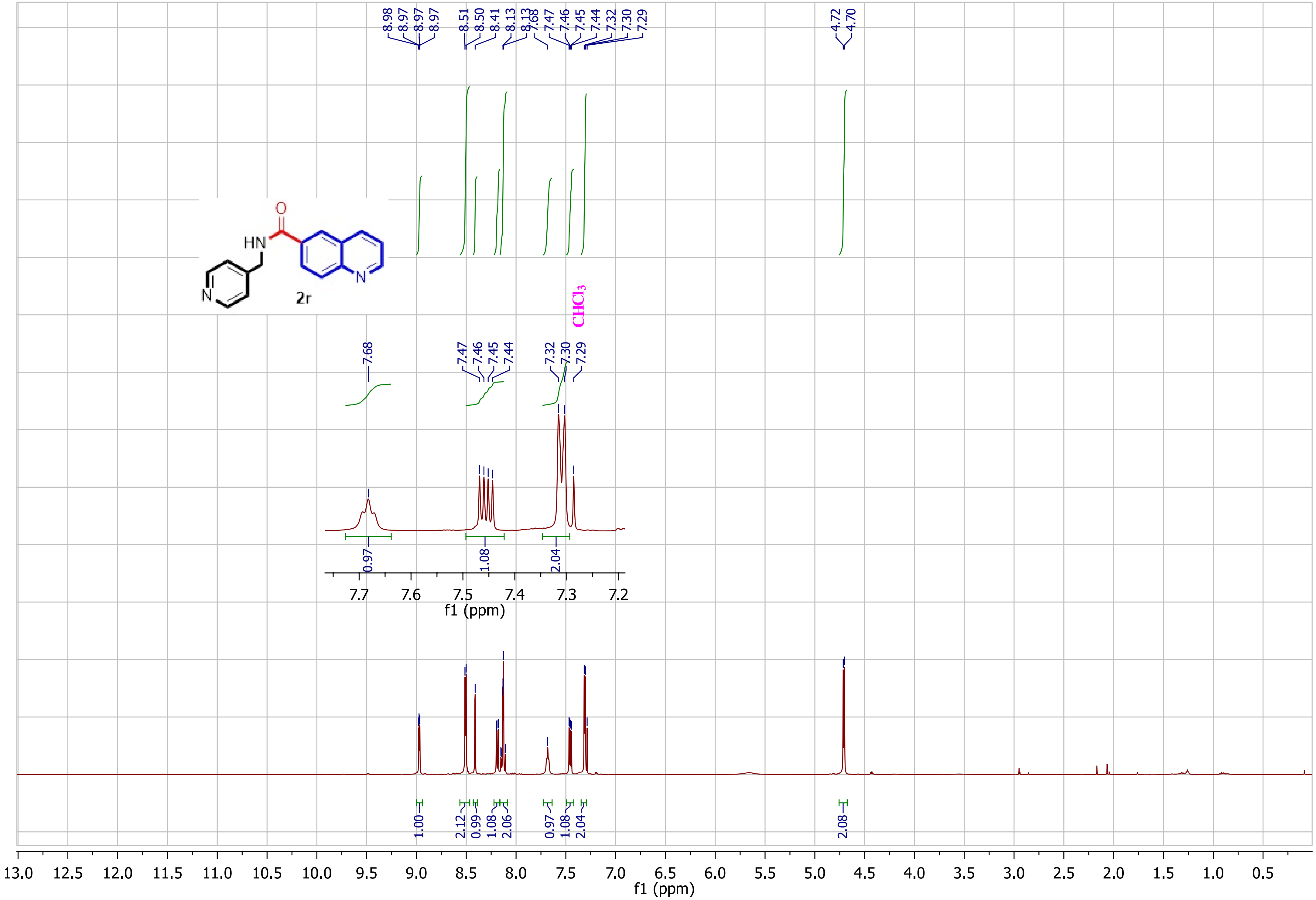
8.98
8.97
8.97
8.97
8.51
8.50
8.41
8.13
8.13
7.68
7.47
7.46
7.45
7.44
7.32
7.30
7.29
4.72
4.70
HN
O
N
N
2r
CHCl3
7.68
7.47
7.46
7.45
7.44
7.32
7.30
7.29
0.97
1.08
2.04
7.7
7.6
7.5
7.4
7.3
7.2
f1 (ppm)
1.00
2.12
0.99
1.08
2.06
0.97
1.08
2.04
2.08
13.0
12.5
12.0
11.5
11.0
10.5
10.0
9.5
9.0
8.5
8.0
7.5
7.0
6.5
6.0
5.5
5.0
4.5
4.0
3.5
3.0
2.5
2.0
1.5
1.0
0.5
f1 (ppm)

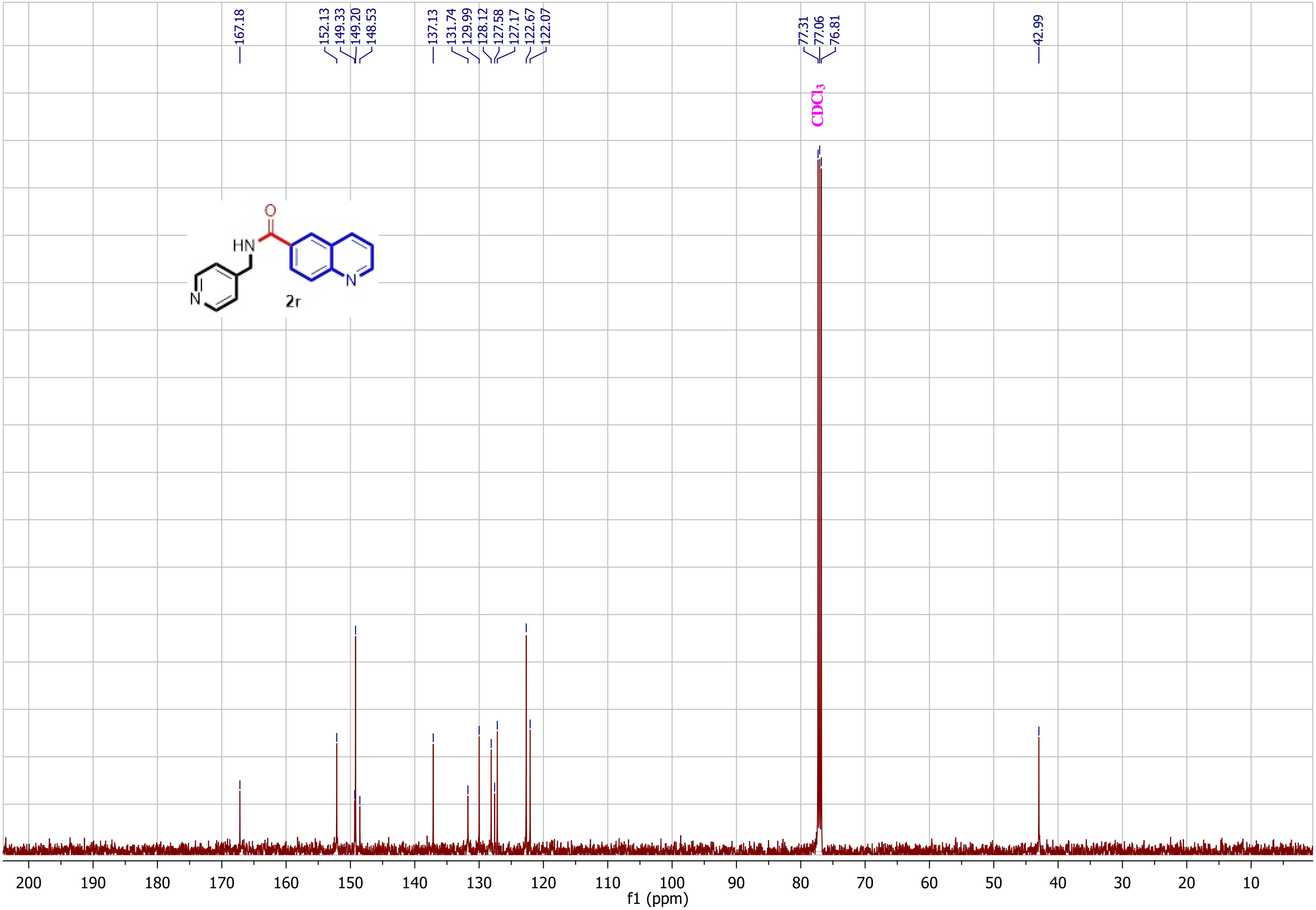
167.18
152.13
149.33
149.20
148.53
137.13
131.74
129.99
128.12
127.58
127.17
122.67
122.07
77.31
77.06
76.81
42.99
CDCl3
O
HN
N
N
2r
200 190 180 170 160 150 140 130 120 110 100 90 80 70 60 50 40 30 20 10
f1 (ppm)

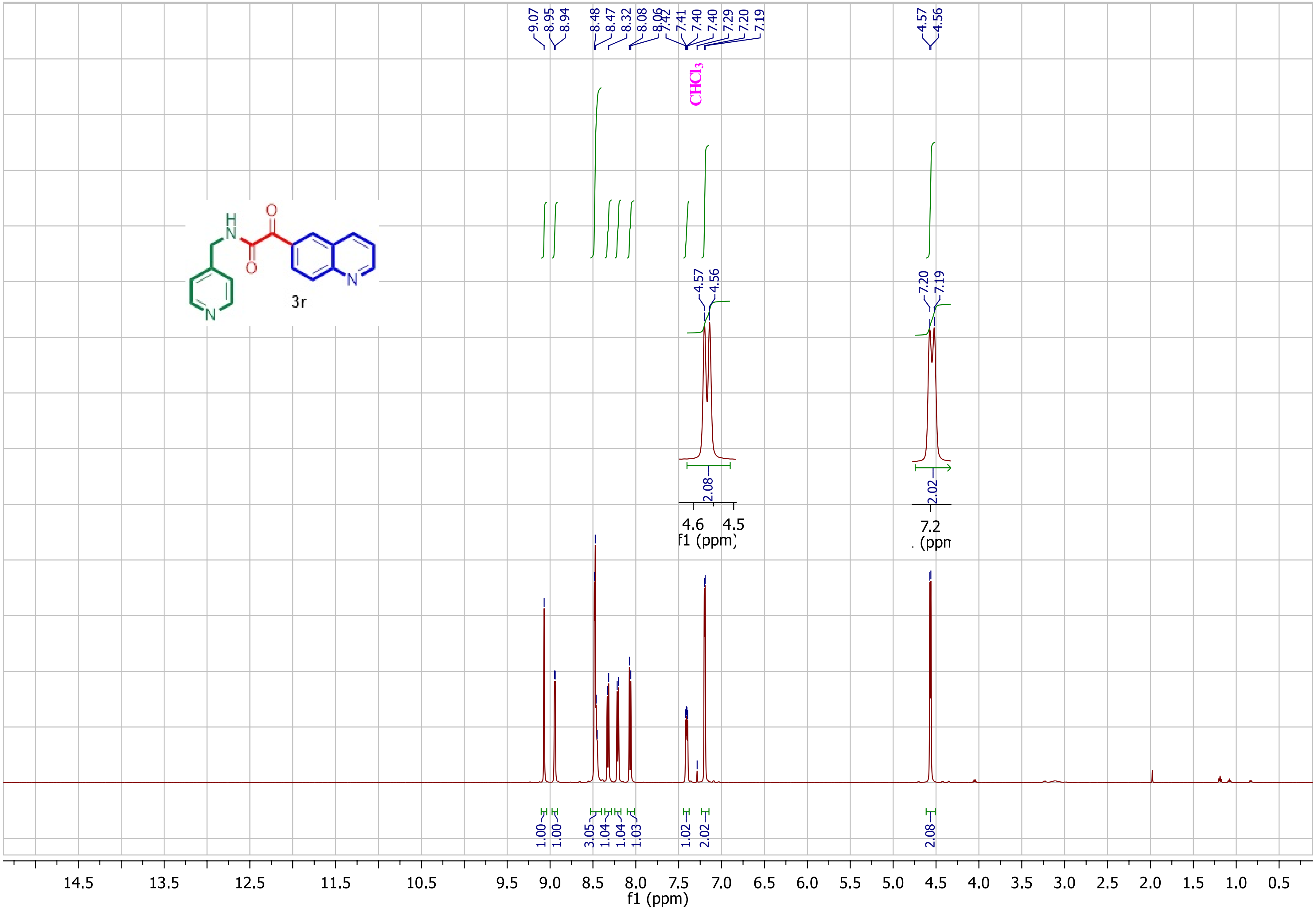

9.07
8.95
8.94
8.48
8.47
8.32
8.08
8.06
7.42
7.41
7.40
7.40
7.29
7.20
7.19
CHCl3
4.57
4.56
H
N
O
O
N
N
3r
4.57
4.56
2.08
4.6
4.5
f1 (ppm)
7.20
7.19
2.02
7.2
1.00
1.00
3.05
1.04
1.04
1.03
1.02
2.02
2.08
14.5
13.5
12.5
11.5
10.5
9.5
9.0
8.5
8.0
7.5
7.0
6.5
6.0
5.5
5.0
4.5
4.0
3.5
3.0
2.5
2.0
1.5
1.0
0.5
f1 (ppm)

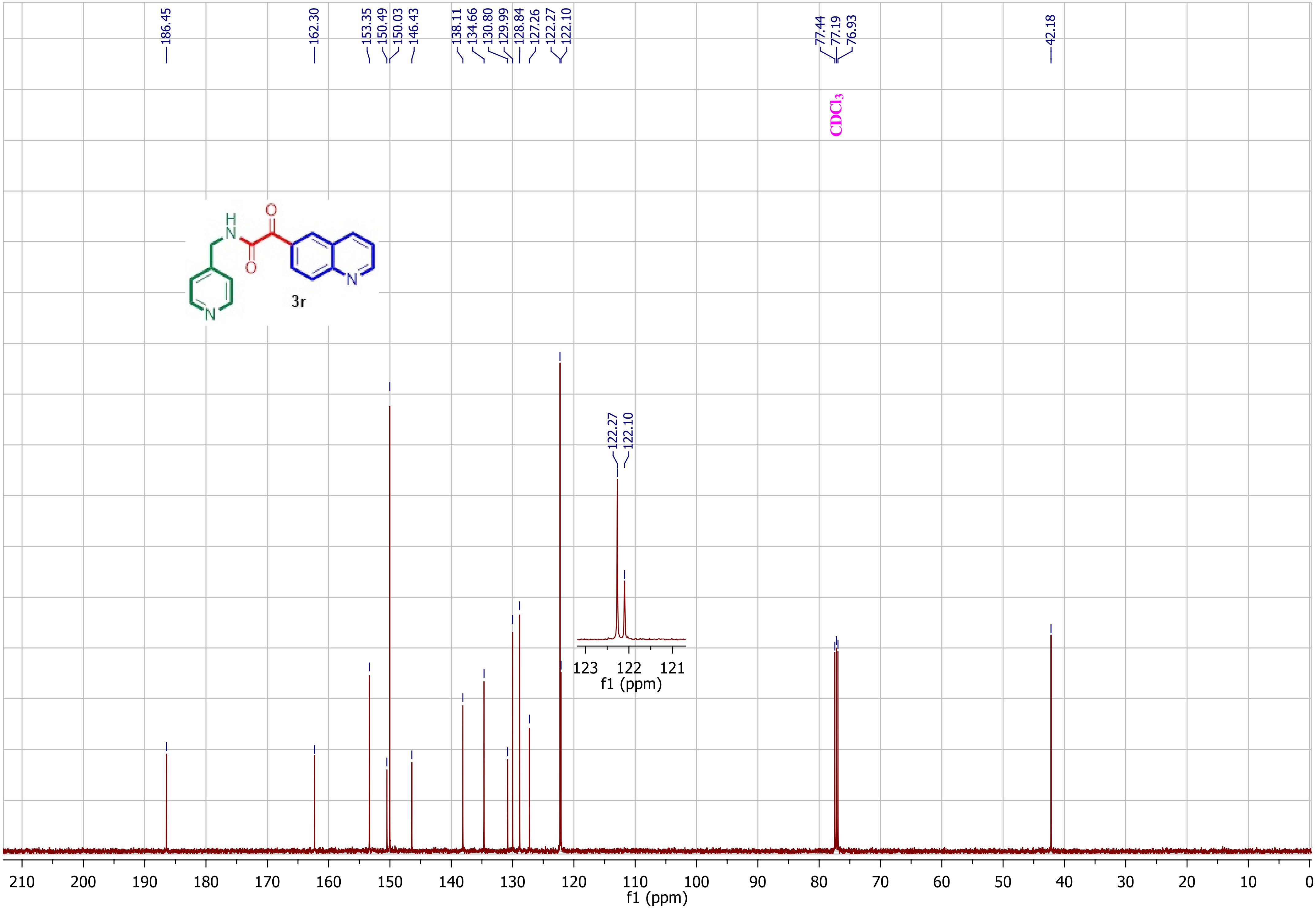
186.45
162.30
153.35
150.49
150.03
146.43
138.11
134.66
130.80
129.99
128.84
127.26
122.27
122.10
77.44
77.19
76.93
42.18
CDCl3
3r
122.27
122.10
123 122 121
f1 (ppm)
210 200 190 180 170 160 150 140 130 120 110 100 90 80 70 60 50 40 30 20 10 0
f1 (ppm)

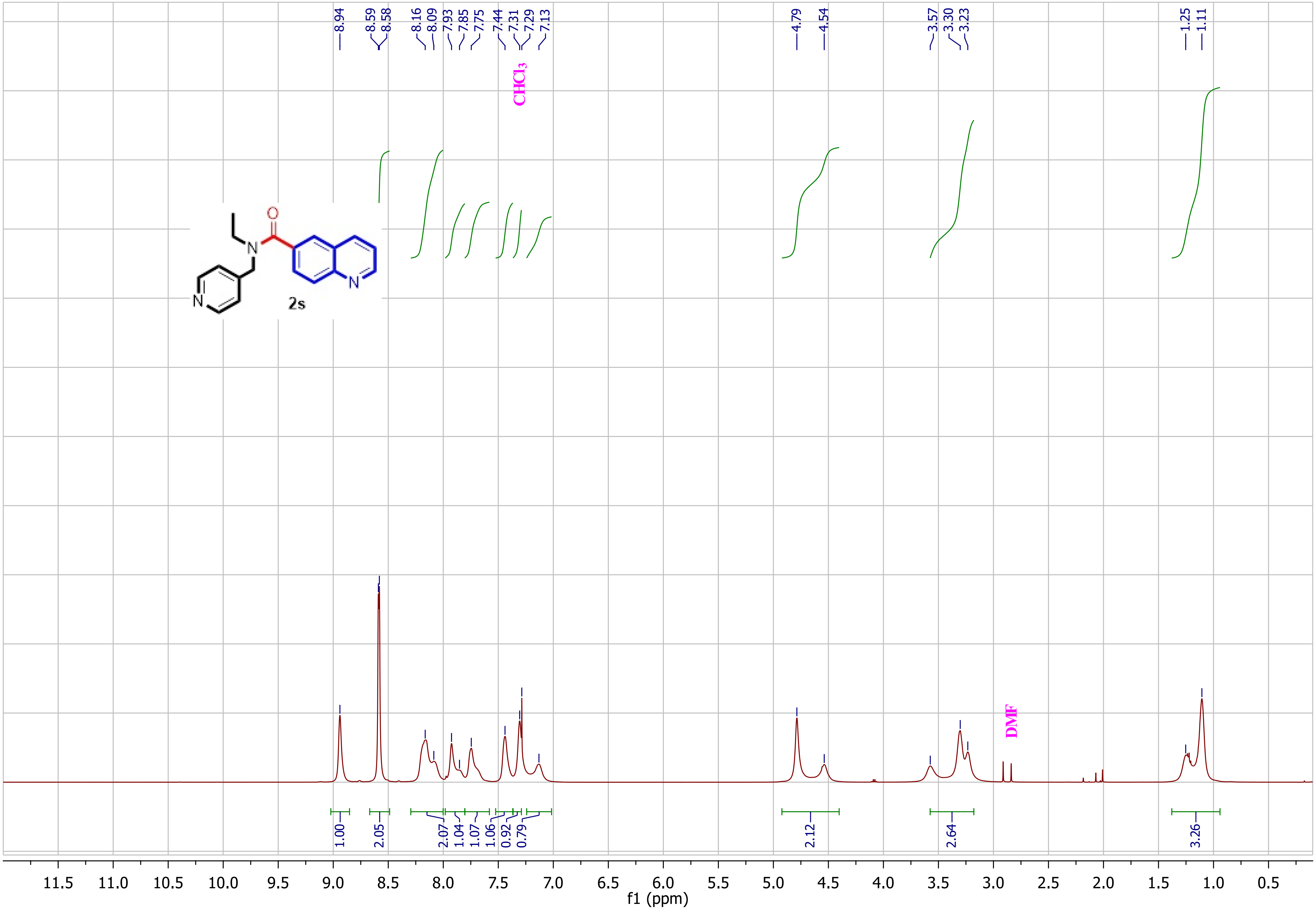
8.94
8.59
8.58
8.16
8.09
7.93
7.85
7.75
7.44
7.31
7.29
7.13
4.79
4.54
3.57
3.30
3.23
1.25
1.11
CHCl3
DMF
O
N
N
N
2s
1.00
2.05
2.07
1.04
1.07
1.06
0.92
0.79
2.12
2.64
3.26
11.5 11.0 10.5 10.0 9.5 9.0 8.5 8.0 7.5 7.0 6.5 6.0 5.5 5.0 4.5 4.0 3.5 3.0 2.5 2.0 1.5 1.0 0.5
f1 (ppm)

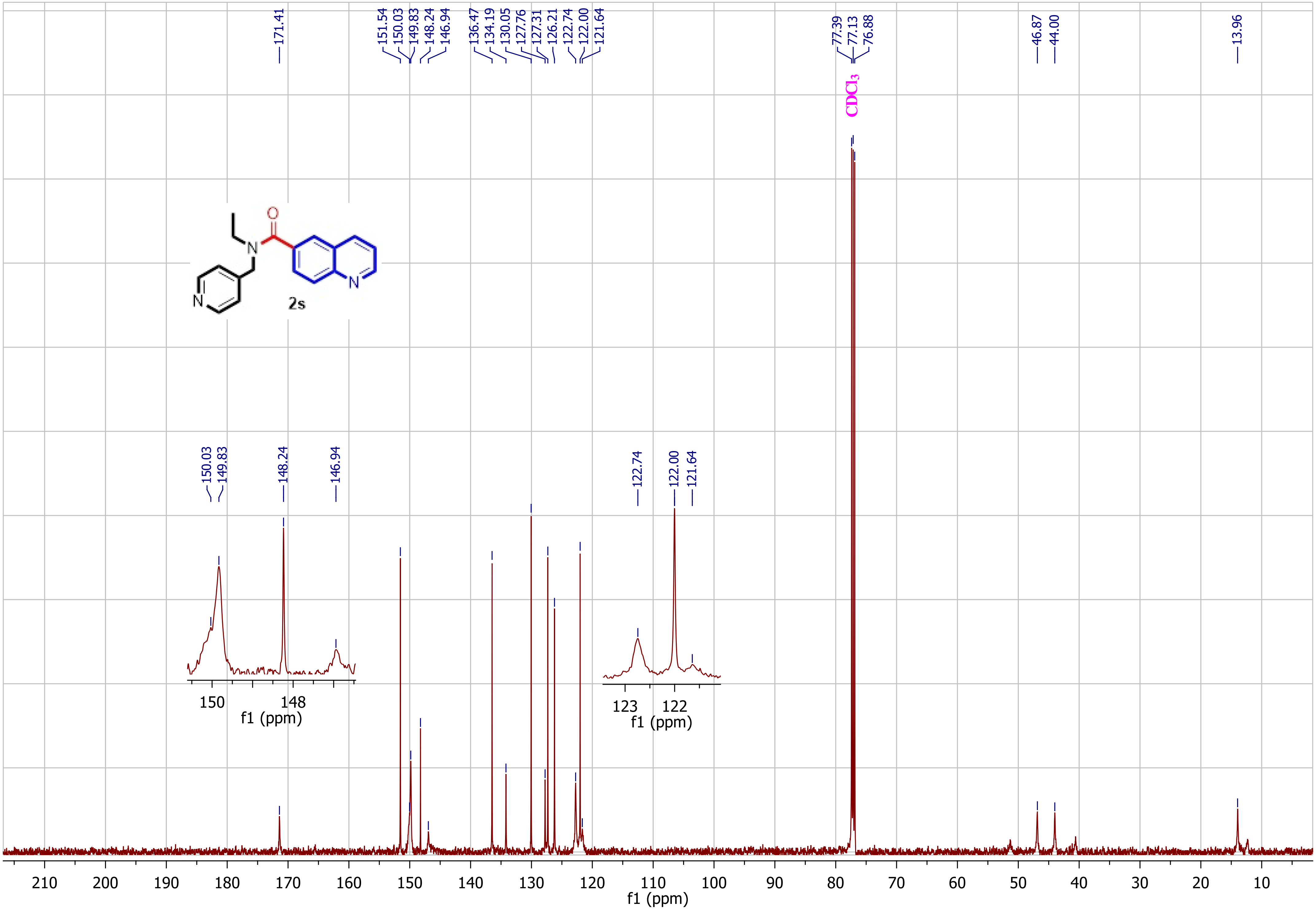

171.41
151.54
150.03
149.83
148.24
146.94
136.47
134.19
130.05
127.76
127.31
126.21
122.74
122.00
121.64
77.39
77.13
76.88
46.87
44.00
13.96
CDCl3
2s
150.03
149.83
148.24
146.94
150
148
f1 (ppm)
122.74
122.00
121.64
123
122
f1 (ppm)
210
200
190
180
170
160
150
140
130
120
110
100
90
80
70
60
50
40
30
20
10
f1 (ppm)

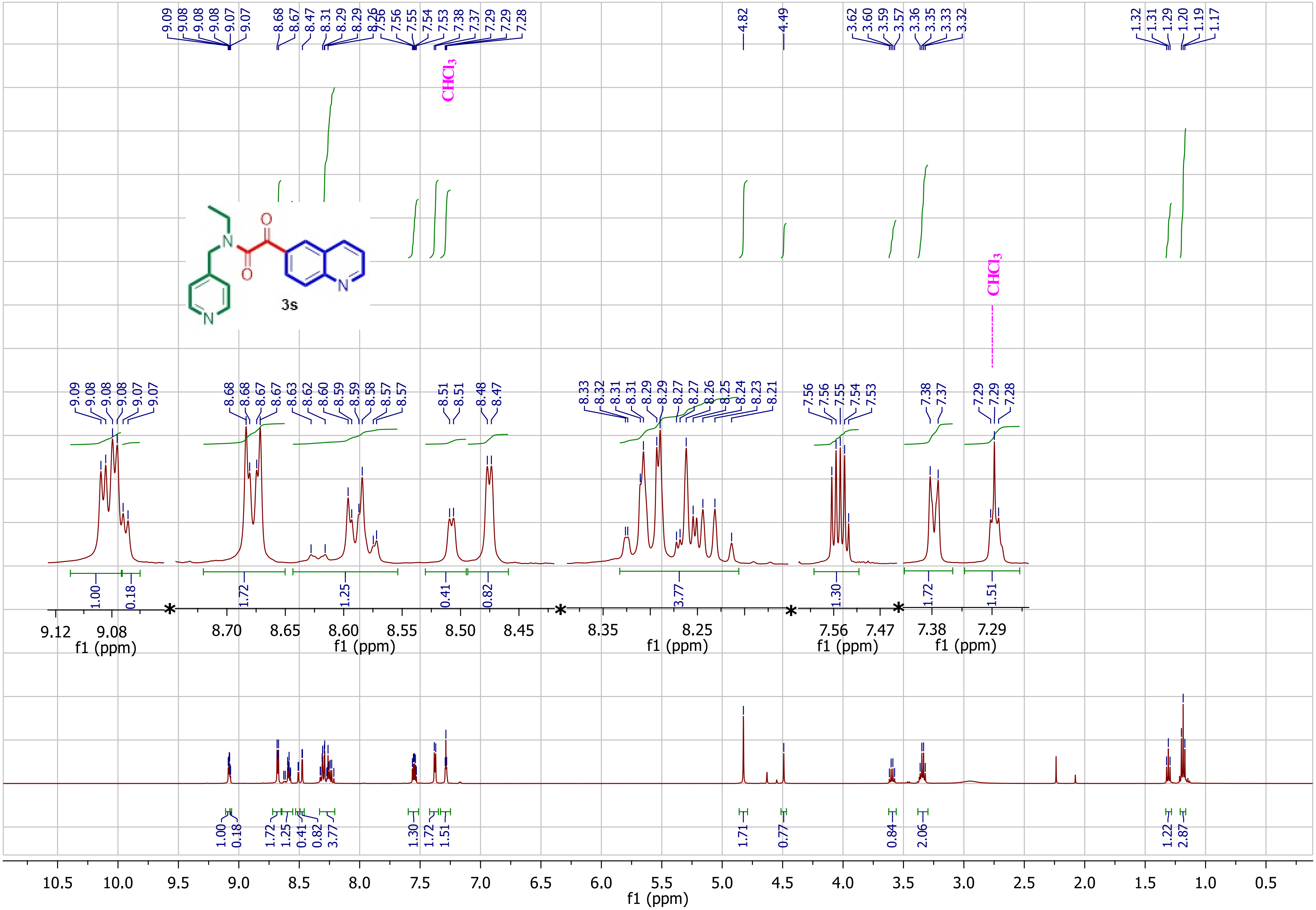

9.09 9.08 9.08 9.08 9.07 9.07 8.68 8.67 8.47 8.31 8.29 8.29 8.26 7.56 7.55 7.54 7.53 7.38 7.37 7.29 7.29 7.28
4.82
4.49
3.62 3.60 3.59 3.57 3.36 3.35 3.33 3.32
1.32 1.31 1.29 1.20 1.19 1.17
CHCl3
3s
1.00 0.18 1.72 1.25 0.41 0.82 3.77 1.30 1.72 1.51 1.71 0.77 0.84 2.06 1.22 2.87
f1 (ppm)

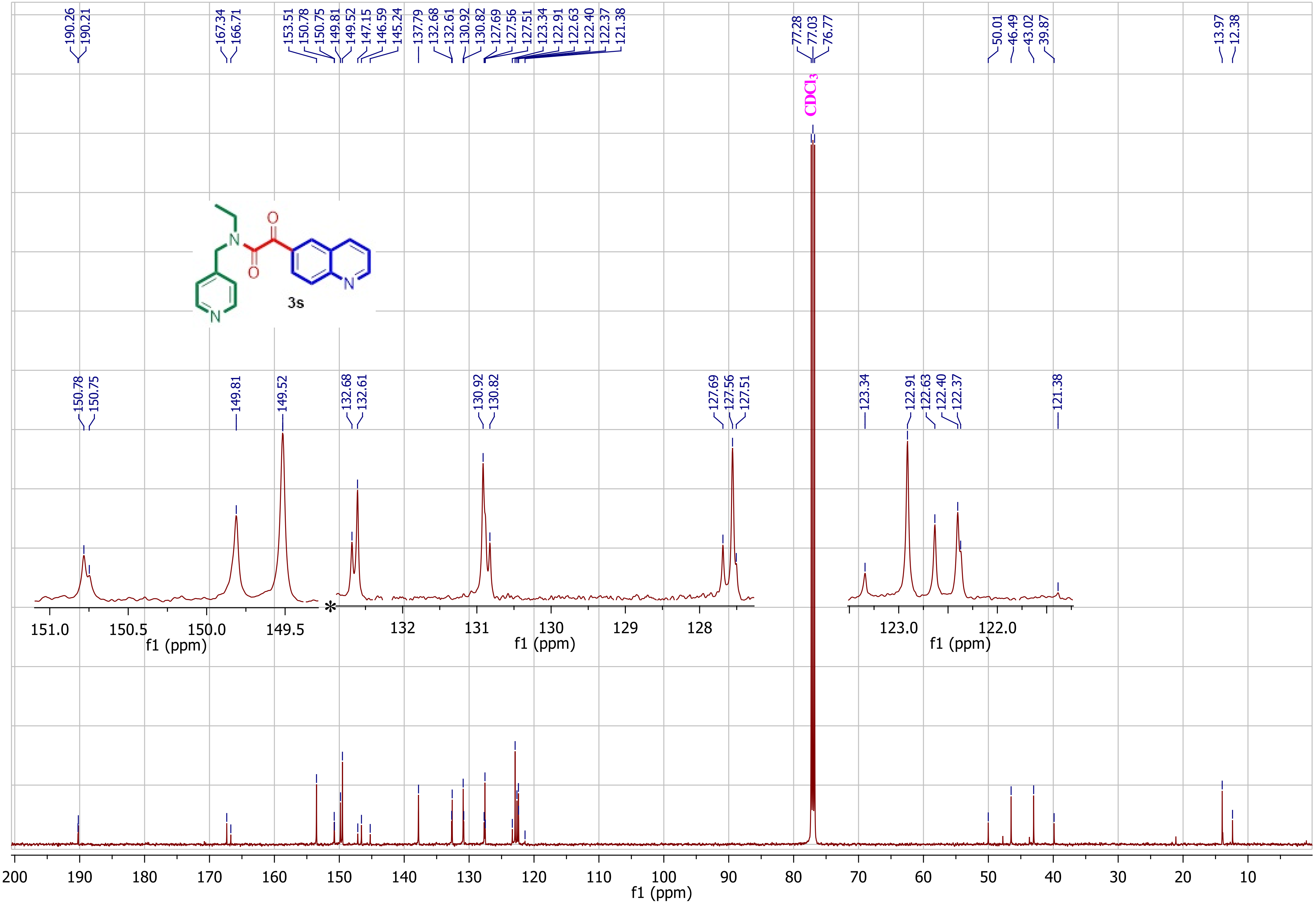

190.26
190.21
167.34
166.71
153.51
150.78
150.75
149.81
149.52
147.15
146.59
145.24
137.79
132.68
132.61
130.92
130.82
127.69
127.56
127.51
123.34
122.91
122.63
122.40
122.37
121.38
77.28
77.03
76.77
50.01
46.49
43.02
39.87
13.97
12.38
$CDCl_3$
3s
f1 (ppm)

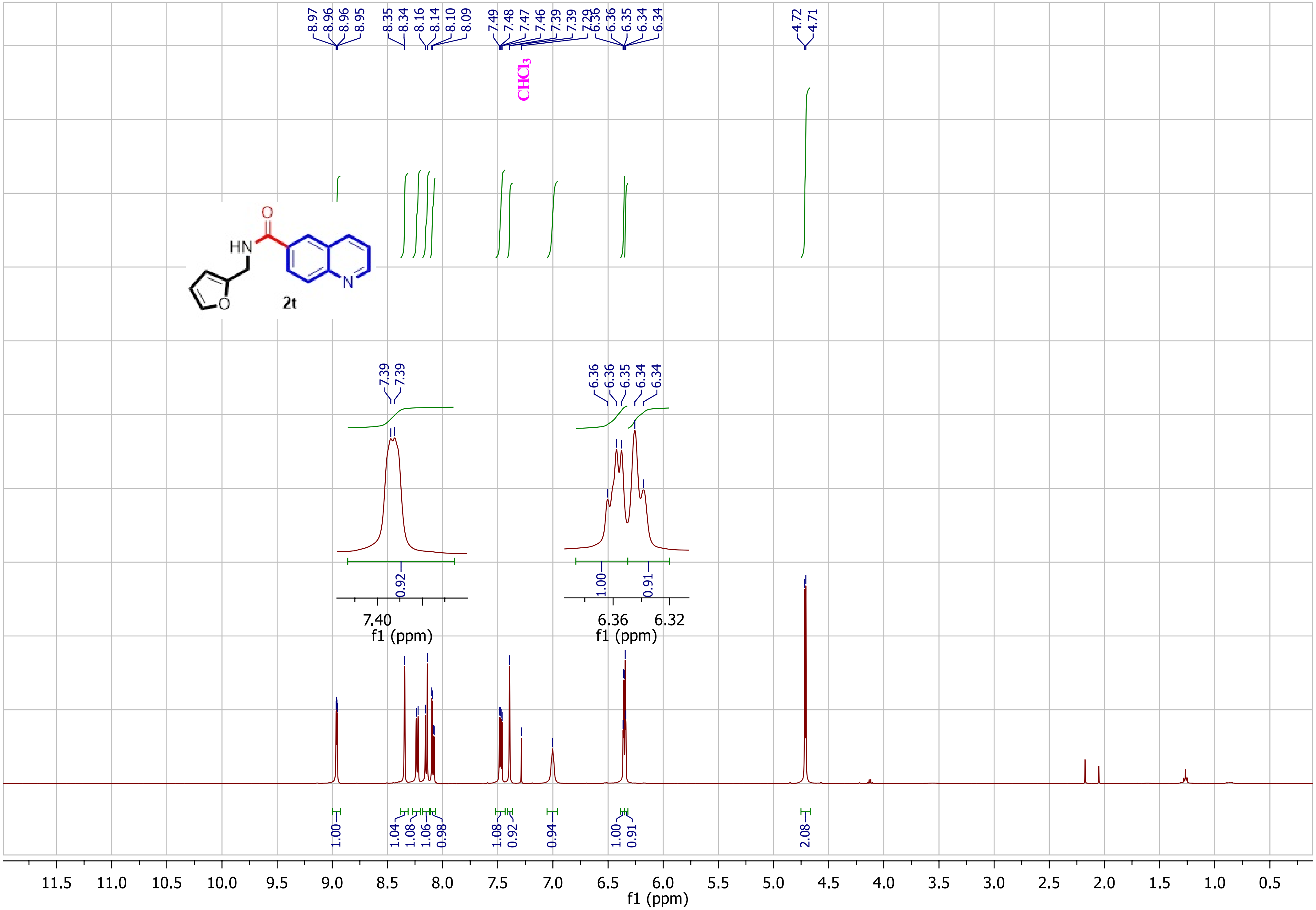
8.97
8.96
8.96
8.95
8.35
8.34
8.16
8.14
8.10
8.09
7.49
7.48
7.47
7.46
7.39
7.39
7.29
6.36
6.36
6.35
6.34
6.34
4.72
4.71
CHCl3
HN
O
N
O
2t
7.39
7.39
0.92
7.40
f1 (ppm)
6.36
6.36
6.35
6.34
6.34
1.00
0.91
6.36
6.32
f1 (ppm)
1.00
1.04
1.08
1.06
0.98
1.08
0.92
0.94
1.00
0.91
2.08
11.5
11.0
10.5
10.0
9.5
9.0
8.5
8.0
7.5
7.0
6.5
6.0
5.5
5.0
4.5
4.0
3.5
3.0
2.5
2.0
1.5
1.0
0.5
f1 (ppm)

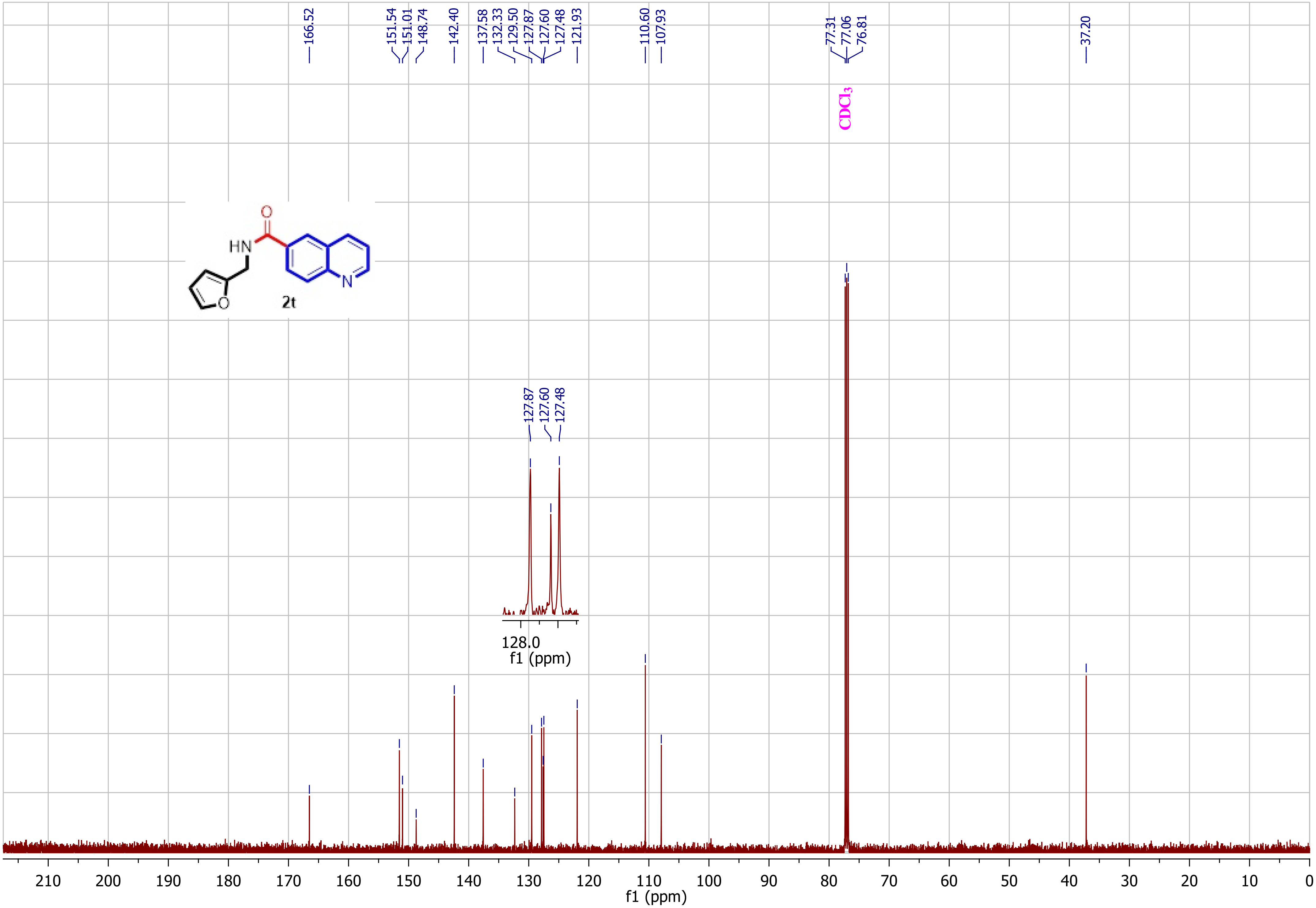
166.52
151.54
151.01
148.74
142.40
137.58
132.33
129.50
127.87
127.60
127.48
121.93
110.60
107.93
77.31
77.06
76.81
37.20
CDCl3
O
HN
N
O
2t
127.87
127.60
127.48
128.0
f1 (ppm)
210 200 190 180 170 160 150 140 130 120 110 100 90 80 70 60 50 40 30 20 10 0
f1 (ppm)

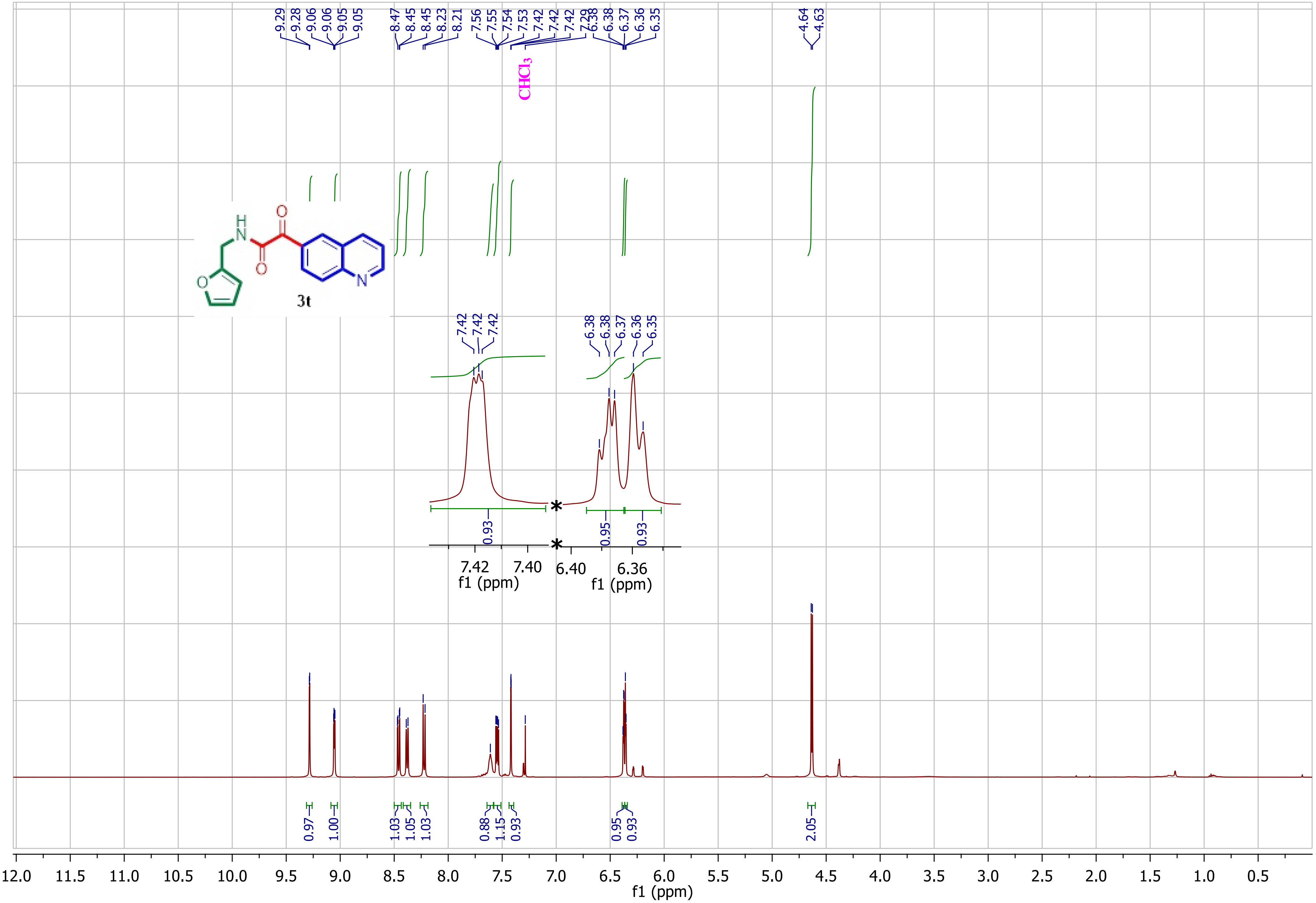

9.29
9.28
9.06
9.06
9.05
9.05
8.47
8.45
8.45
8.23
8.21
7.56
7.55
7.54
7.53
7.42
7.42
7.42
7.29
6.38
6.37
6.36
6.35
4.64
4.63
CHCl3
3t
7.42
7.42
7.42
6.38
6.38
6.37
6.36
6.35
0.93
0.95
0.93
7.42
7.40
6.40
6.36
f1 (ppm)
f1 (ppm)
0.97
1.00
1.03
1.05
1.03
0.88
1.15
0.93
0.95
0.93
2.05
12.0
11.5
11.0
10.5
10.0
9.5
9.0
8.5
8.0
7.5
7.0
6.5
6.0
5.5
5.0
4.5
4.0
3.5
3.0
2.5
2.0
1.5
1.0
0.5
f1 (ppm)

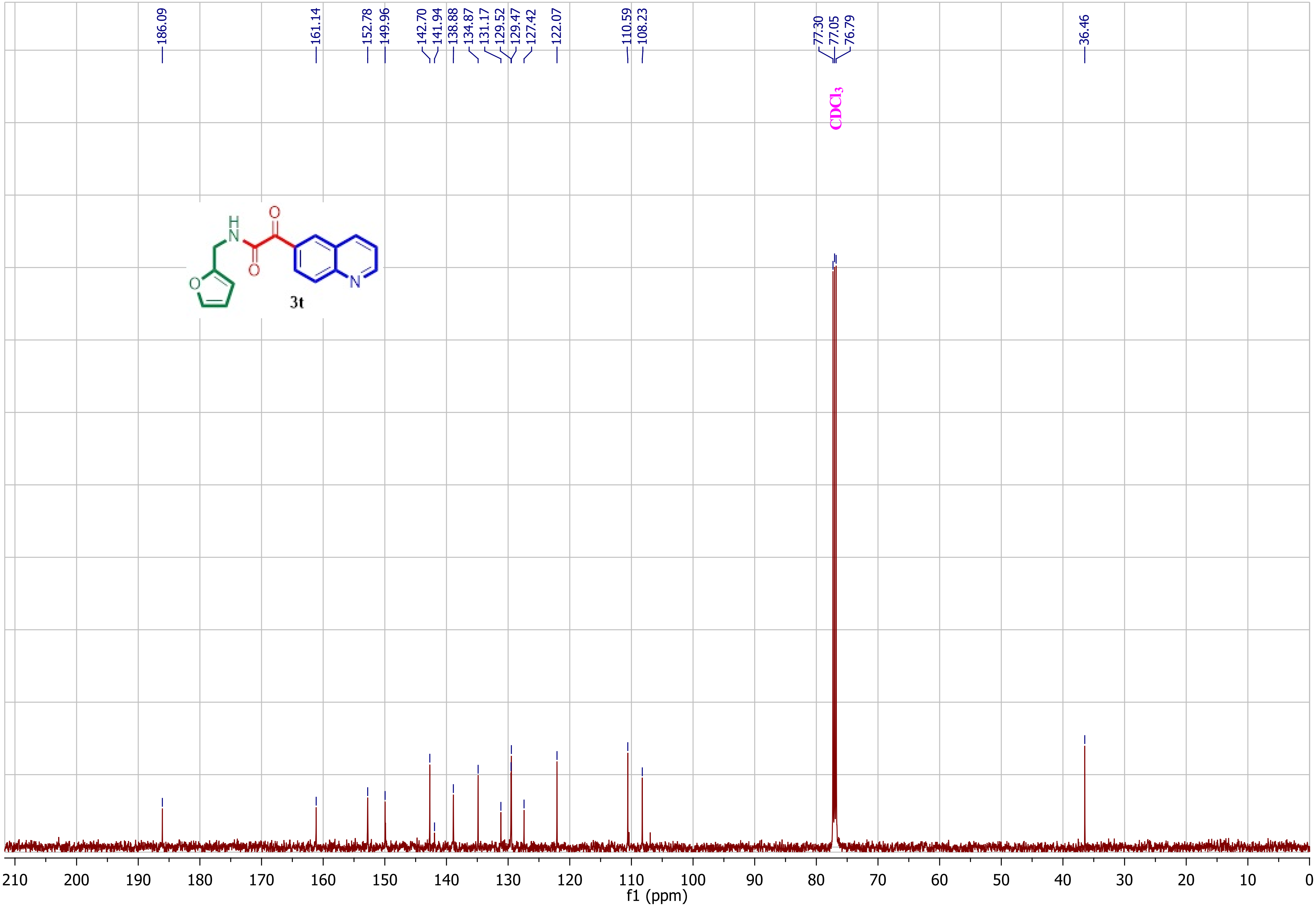
186.09
161.14
152.78
149.96
142.70
141.94
138.88
134.87
131.17
129.52
129.47
127.42
122.07
110.59
108.23
77.30
77.05
76.79
36.46
CDCl3
3t
210
200
190
180
170
160
150
140
130
120
110
100
90
80
70
60
50
40
30
20
10
0
f1 (ppm)

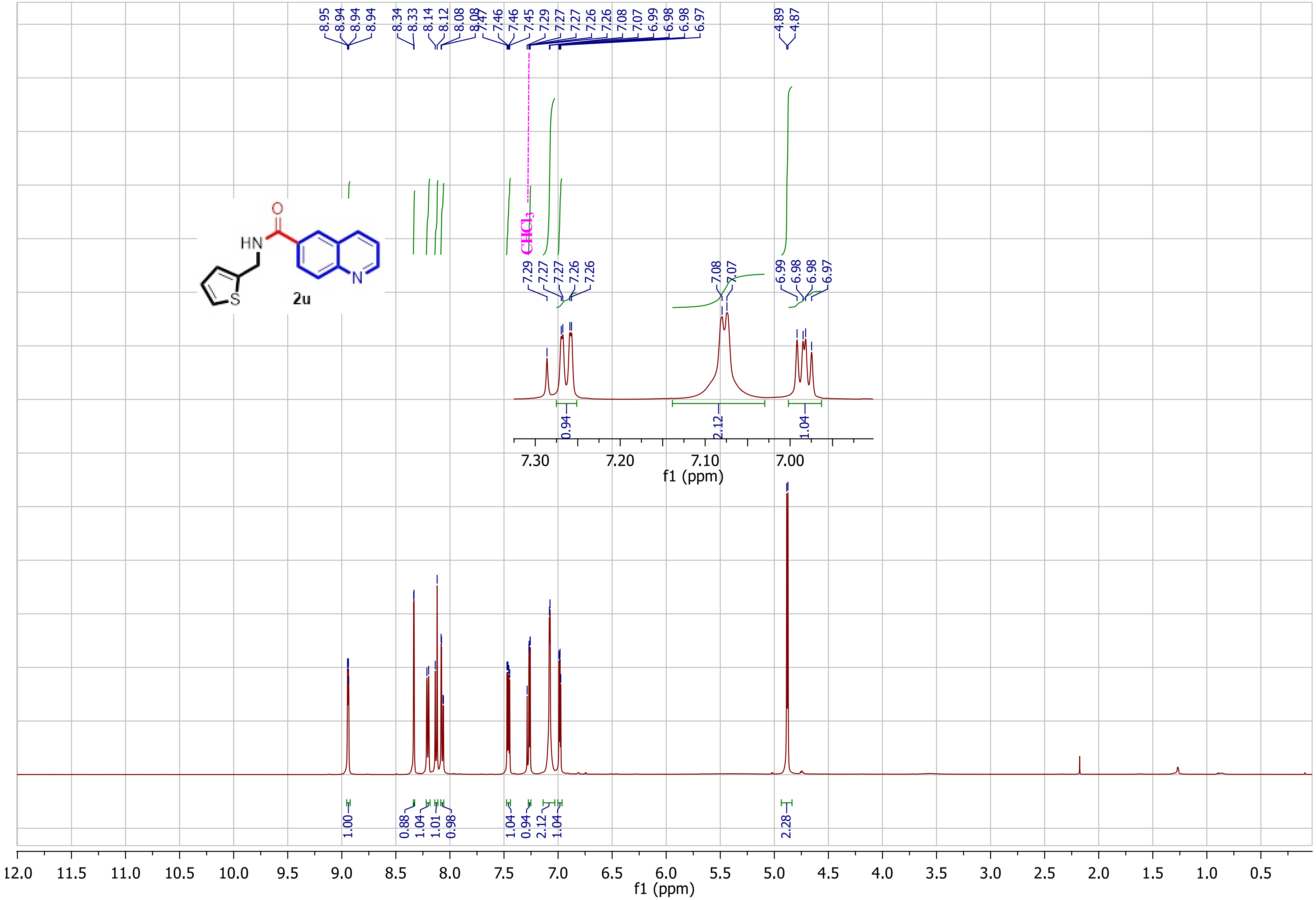

2u
CHCl3
f1 (ppm)

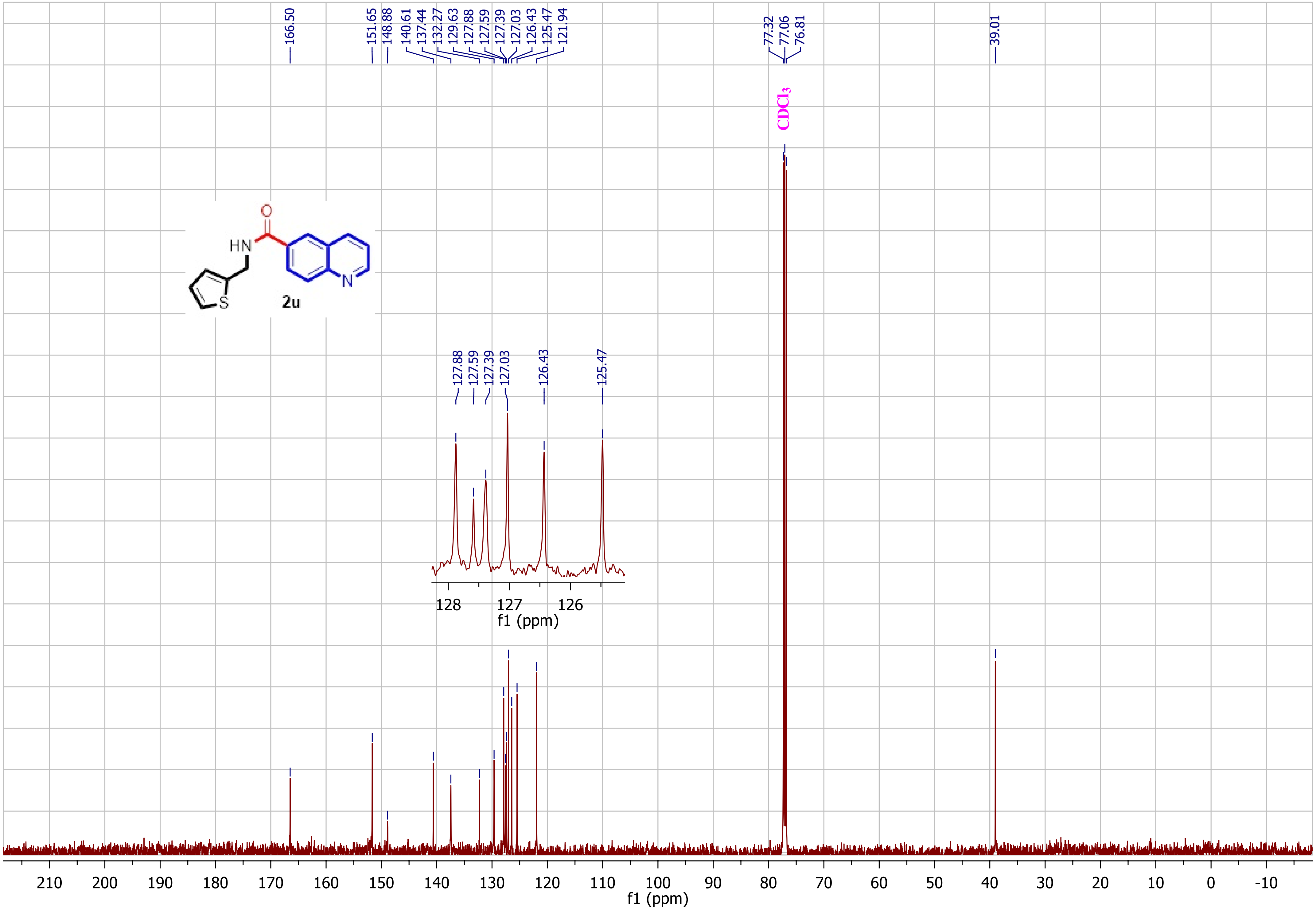
166.50
151.65
148.88
140.61
137.44
132.27
129.63
127.88
127.59
127.39
127.03
126.43
125.47
121.94
77.32
77.06
76.81
39.01
CDCl3
HN
O
N
S
2u
127.88
127.59
127.39
127.03
126.43
125.47
128
127
126
f1 (ppm)
210 200 190 180 170 160 150 140 130 120 110 100 90 80 70 60 50 40 30 20 10 0 -10
f1 (ppm)

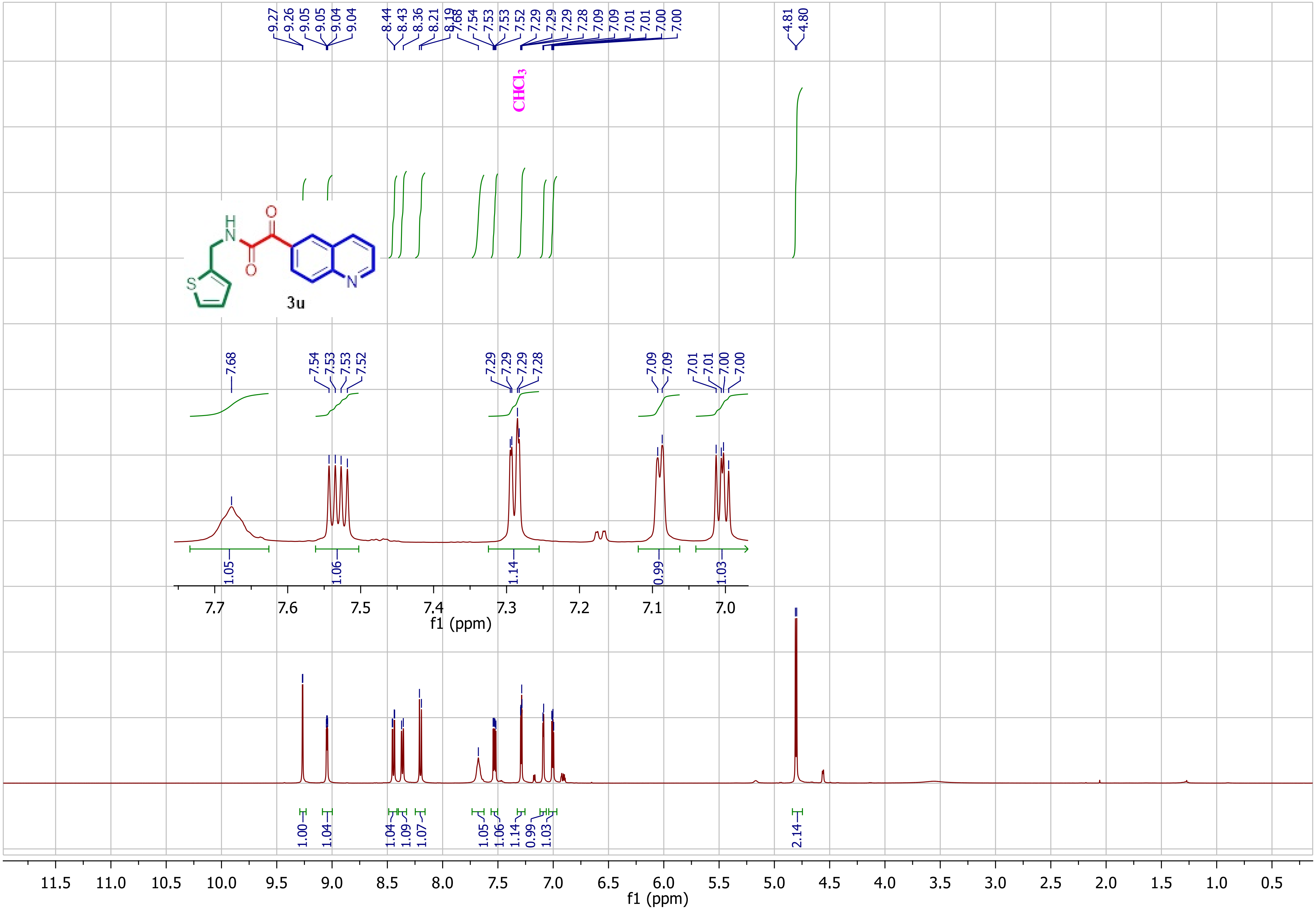

9.27
9.26
9.05
9.05
9.04
9.04
8.44
8.43
8.36
8.21
8.19
7.68
7.54
7.53
7.53
7.52
7.29
7.29
7.29
7.28
7.09
7.09
7.01
7.01
7.00
7.00
4.81
4.80
CHCl3
3u
1.05
1.06
1.14
0.99
1.03
f1 (ppm)
1.00
1.04
1.04
1.09
1.07
1.05
1.06
1.14
0.99
1.03
2.14
f1 (ppm)

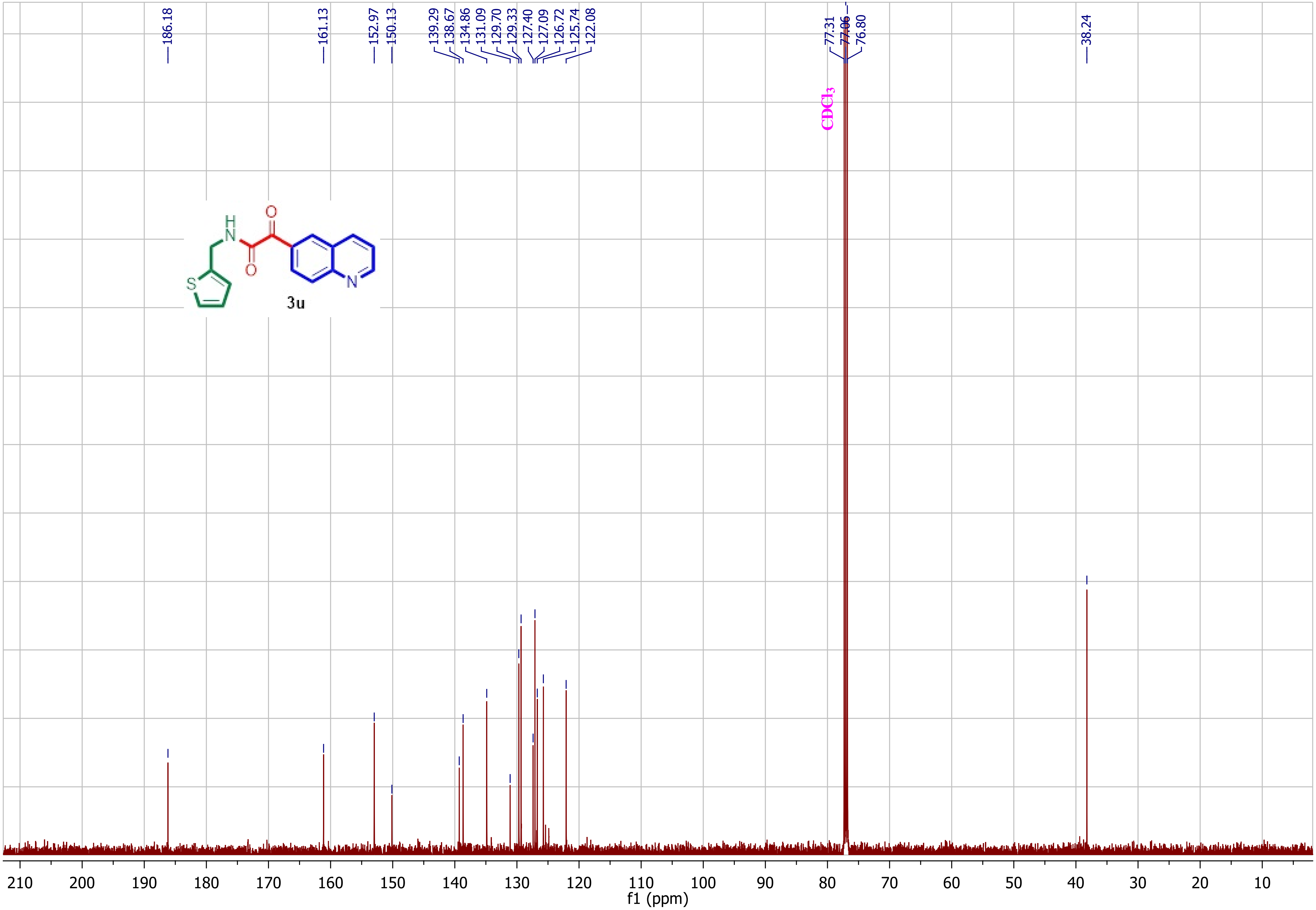
186.18
161.13
152.97
150.13
139.29
138.67
134.86
131.09
129.70
129.33
127.40
127.09
126.72
125.74
122.08
77.31
77.06
76.80
38.24
CDCl3
210
200
190
180
170
160
150
140
130
120
110
100
90
80
70
60
50
40
30
20
10
f1 (ppm)
H
N
O
O
S
N

3u

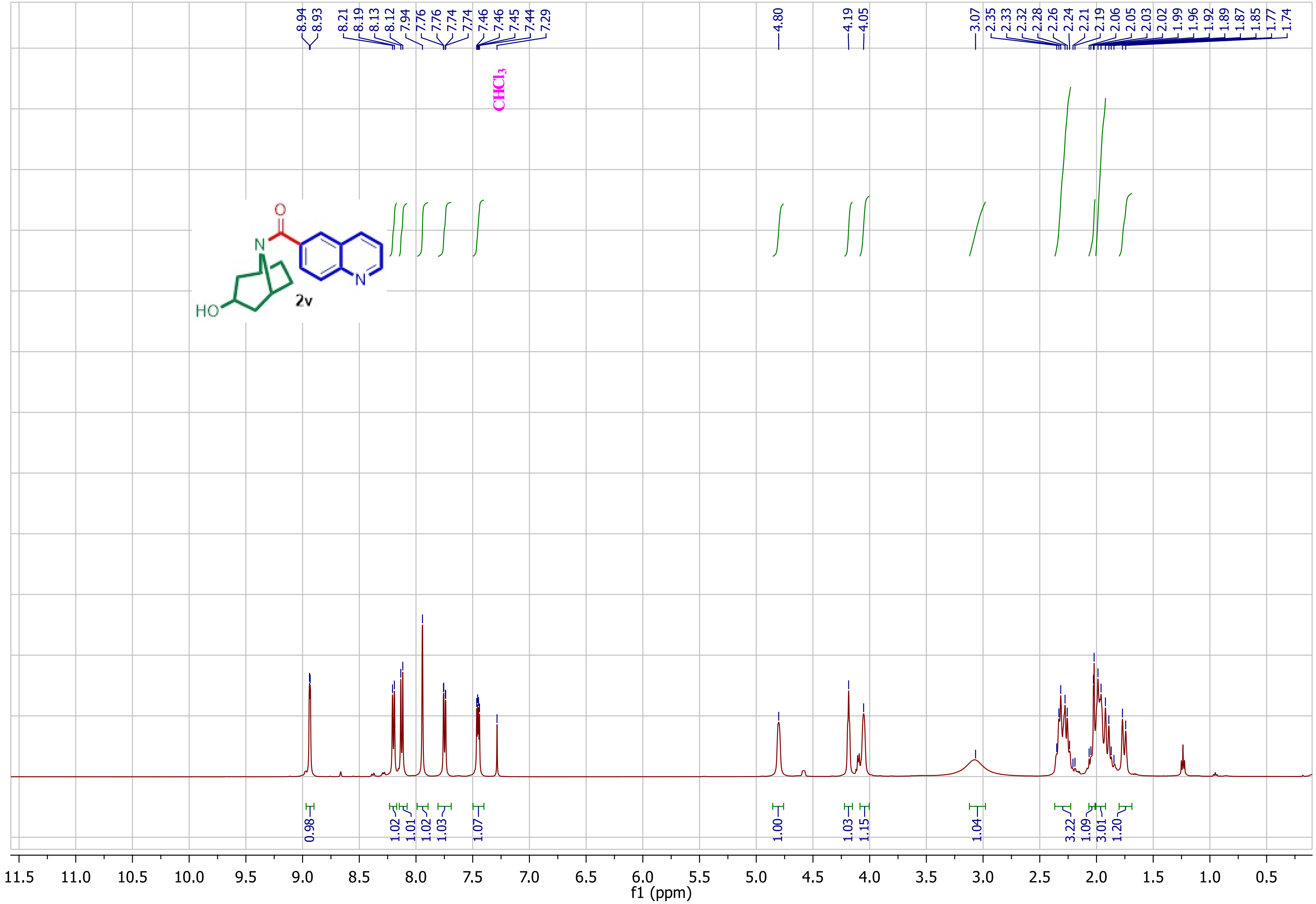

8.94
8.93
8.21
8.19
8.13
8.12
7.94
7.76
7.76
7.74
7.74
7.46
7.46
7.45
7.44
7.29
4.80
4.19
4.05
3.07
2.35
2.33
2.32
2.28
2.26
2.24
2.21
2.19
2.06
2.05
2.03
2.02
1.99
1.96
1.92
1.89
1.87
1.85
1.77
1.74
CHCl3
O
N
N
HO
2v
0.98
1.02
1.01
1.02
1.03
1.07
1.00
1.03
1.15
1.04
3.22
1.09
3.01
1.20
11.5
11.0
10.5
10.0
9.5
9.0
8.5
8.0
7.5
7.0
6.5
6.0
5.5
5.0
4.5
4.0
3.5
3.0
2.5
2.0
1.5
1.0
0.5
f1 (ppm)

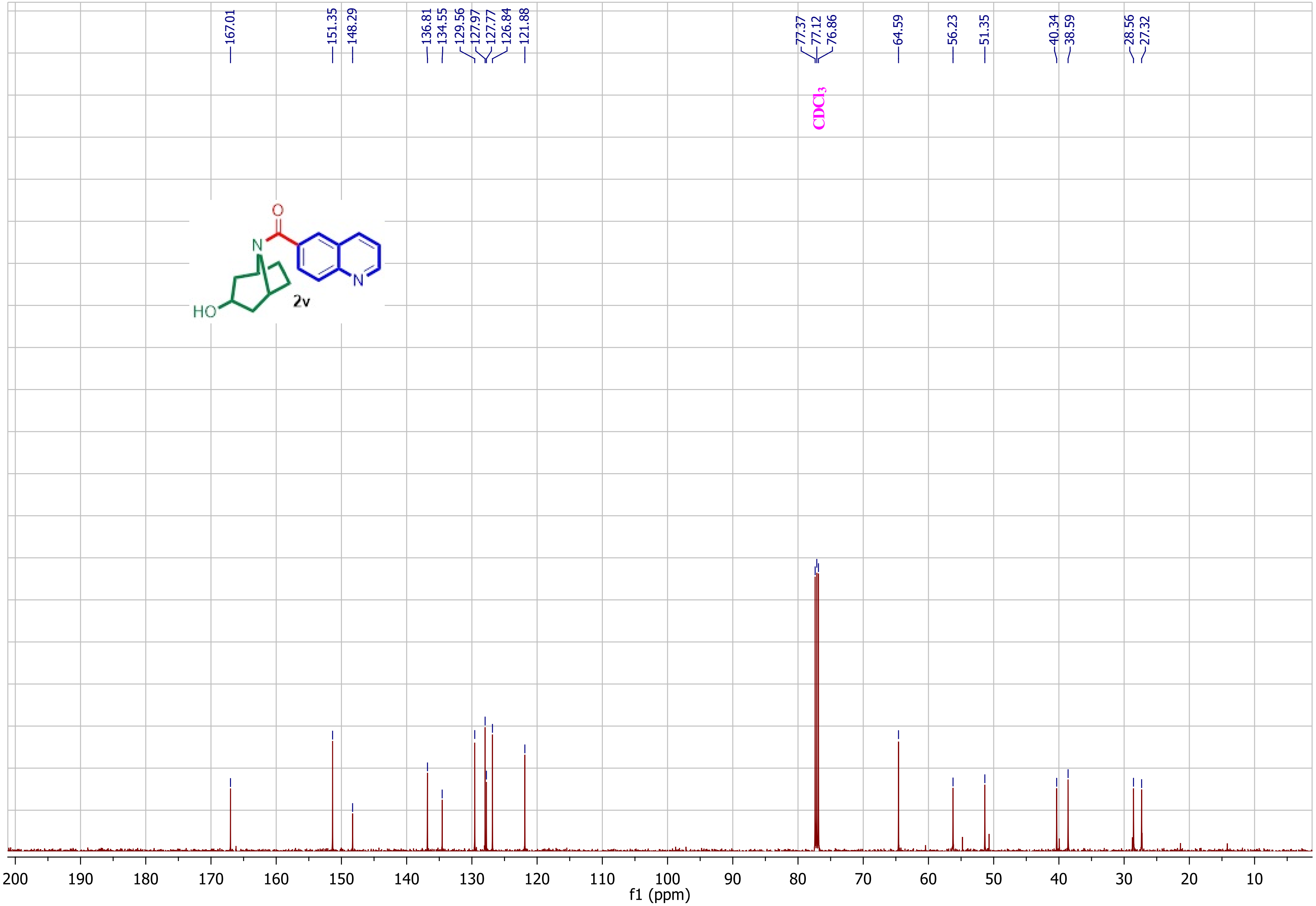
167.01
151.35
148.29
136.81
134.55
129.56
127.97
127.77
126.84
121.88
77.37
77.12
76.86
64.59
56.23
51.35
40.34
38.59
28.56
27.32
CDCl3
O
N
N
HO
2v
200 190 180 170 160 150 140 130 120 110 100 90 80 70 60 50 40 30 20 10
f1 (ppm)

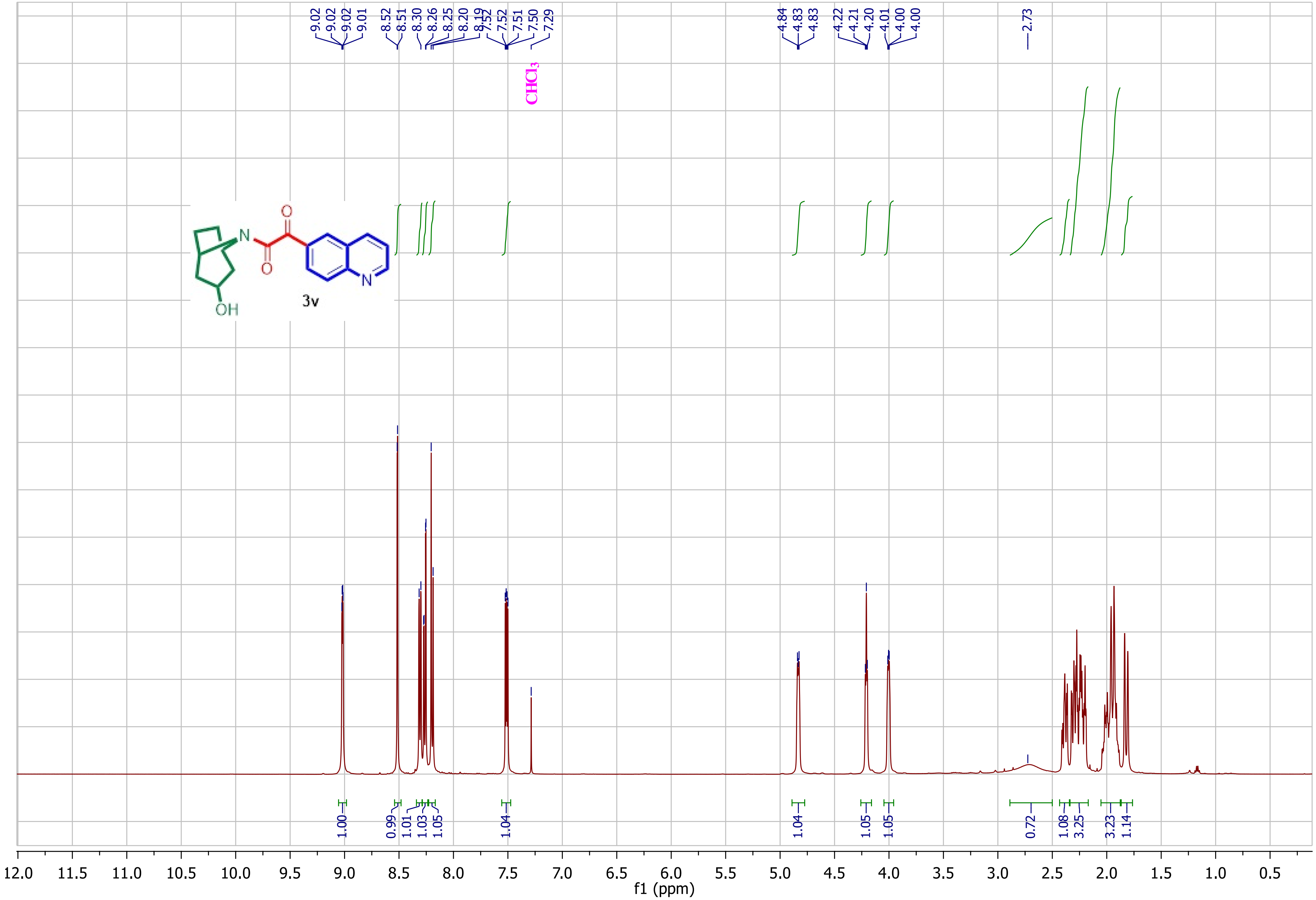
9.02
9.02
9.02
9.01
8.52
8.51
8.30
8.26
8.25
8.20
8.19
7.52
7.52
7.51
7.50
7.29
4.84
4.83
4.83
4.22
4.21
4.20
4.01
4.00
4.00
2.73
CHCl3
N
O
O
N
OH
3v
1.00
0.99
1.01
1.03
1.05
1.04
1.04
1.05
1.05
0.72
1.08
3.25
3.23
1.14
f1 (ppm)

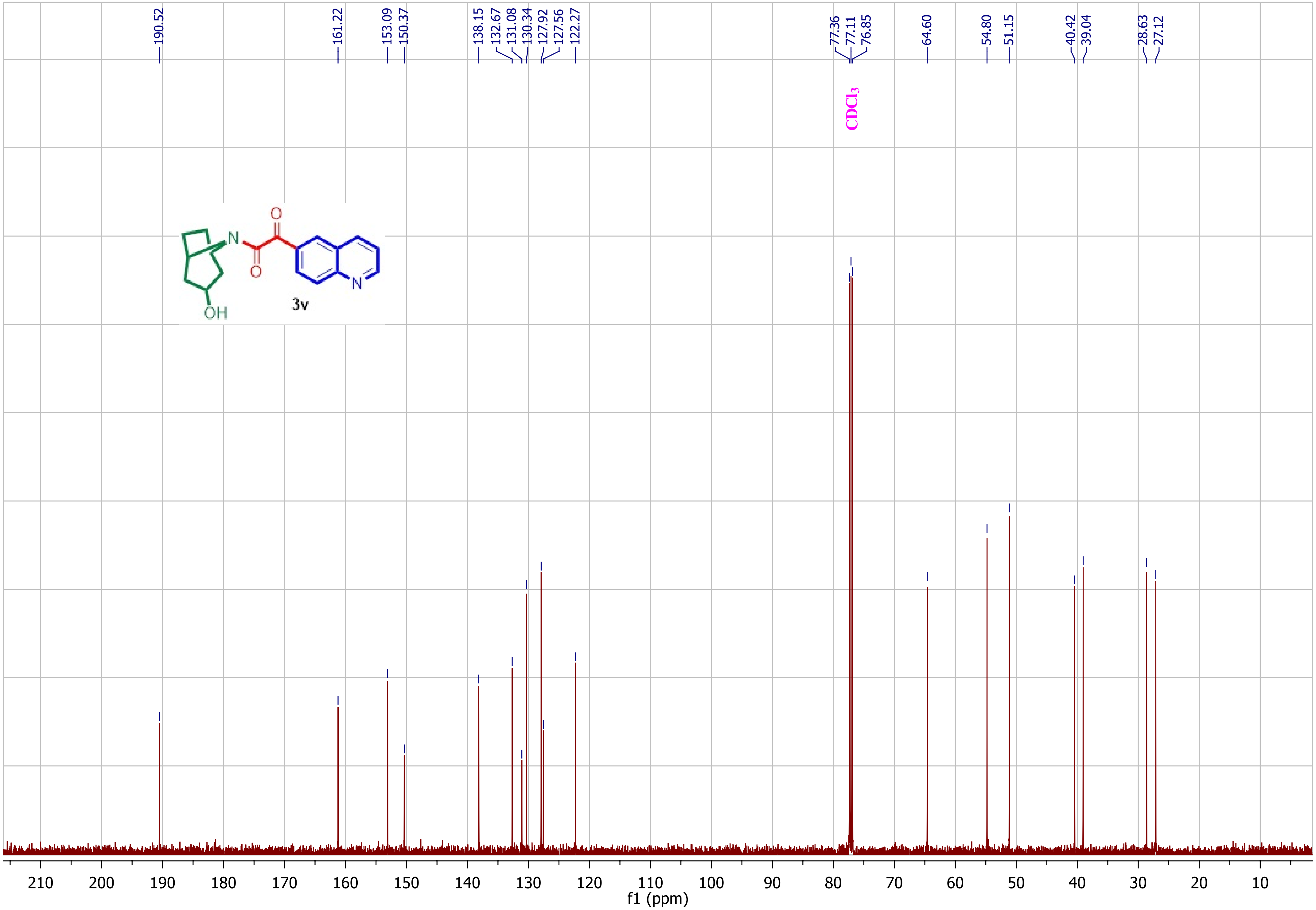
190.52
161.22
153.09
150.37
138.15
132.67
131.08
130.34
127.92
127.56
122.27
77.36
77.11
76.85
64.60
54.80
51.15
40.42
39.04
28.63
27.12
CDCl3
N
O
O
N
OH
3v
210
200
190
180
170
160
150
140
130
120
110
100
90
80
70
60
50
40
30
20
10
f1 (ppm)

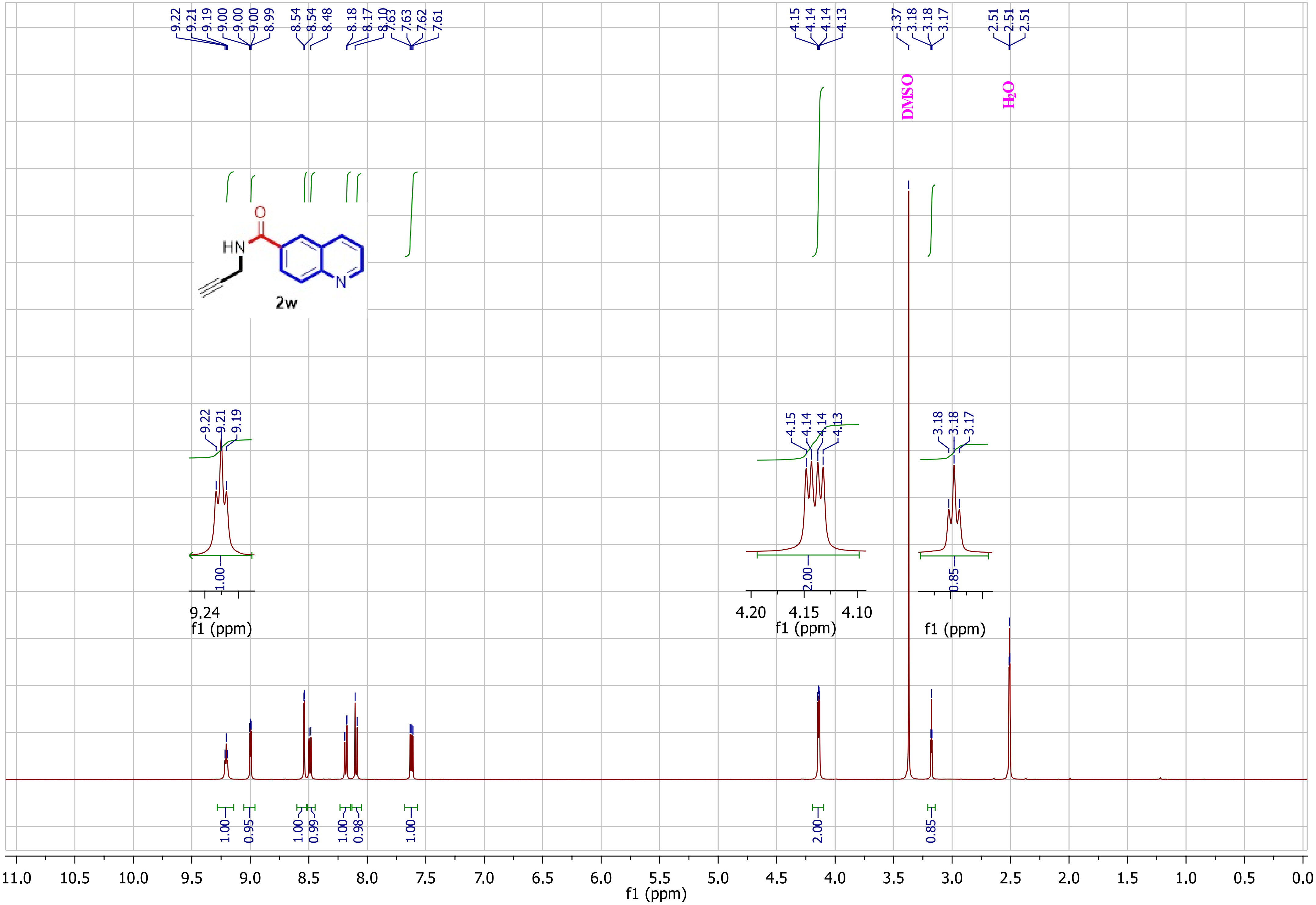

9.22
9.21
9.19
9.00
9.00
9.00
8.99
8.54
8.54
8.48
8.18
8.17
8.10
7.63
7.63
7.62
7.61
4.15
4.14
4.14
4.13
3.37
3.18
3.18
3.17
2.51
2.51
2.51
DMSO
H2O
HN
O
N
2w
1.00
0.95
1.00
0.99
1.00
0.98
1.00
2.00
0.85
f1 (ppm)

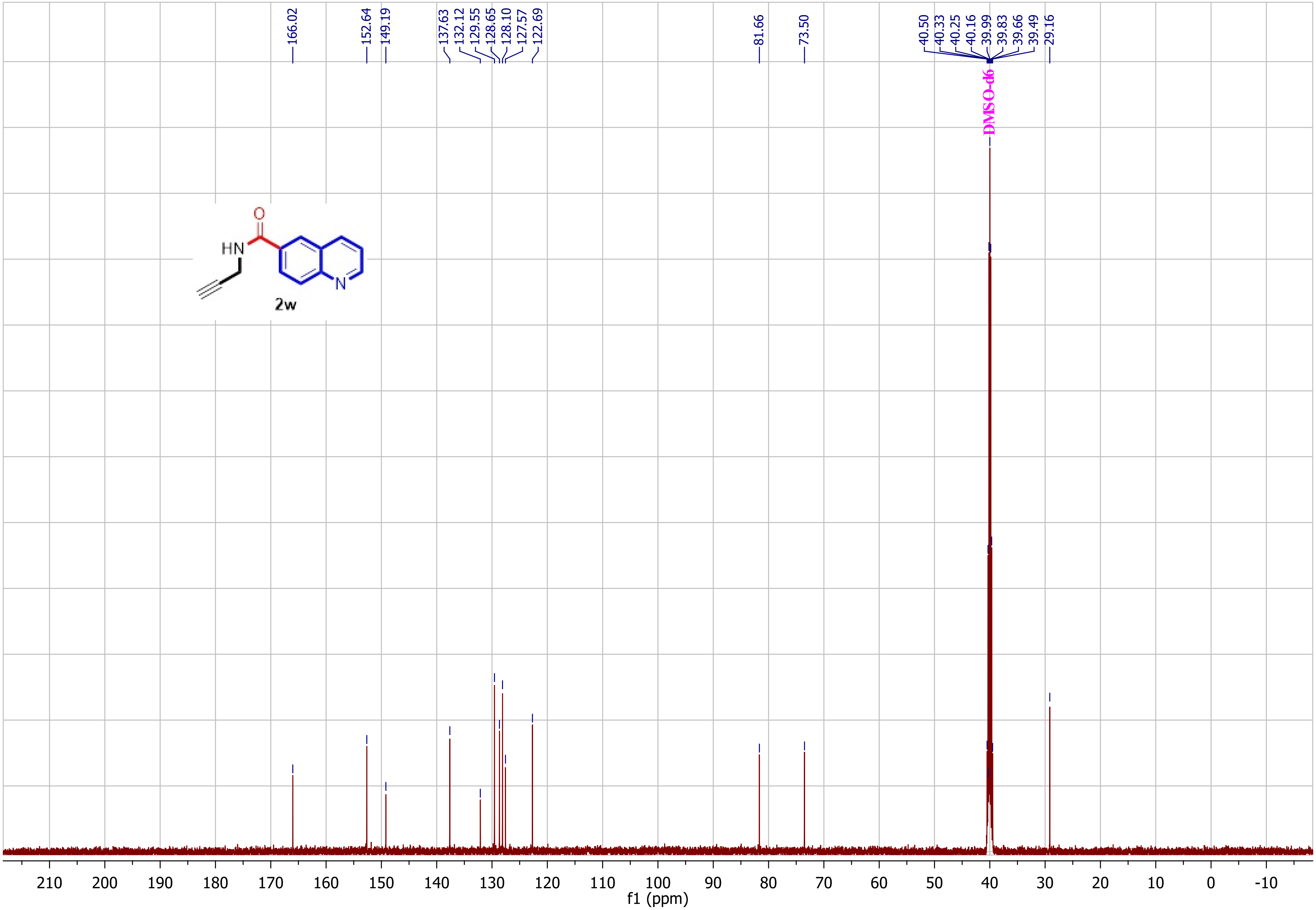
166.02
152.64
149.19
137.63
132.12
129.55
128.65
128.10
127.57
122.69
81.66
73.50
40.50
40.33
40.25
40.16
39.99
39.83
39.66
39.49
29.16
DMSO-d6
O
HN
N
2w
210 200 190 180 170 160 150 140 130 120 110 100 90 80 70 60 50 40 30 20 10 0 -10
f1 (ppm)

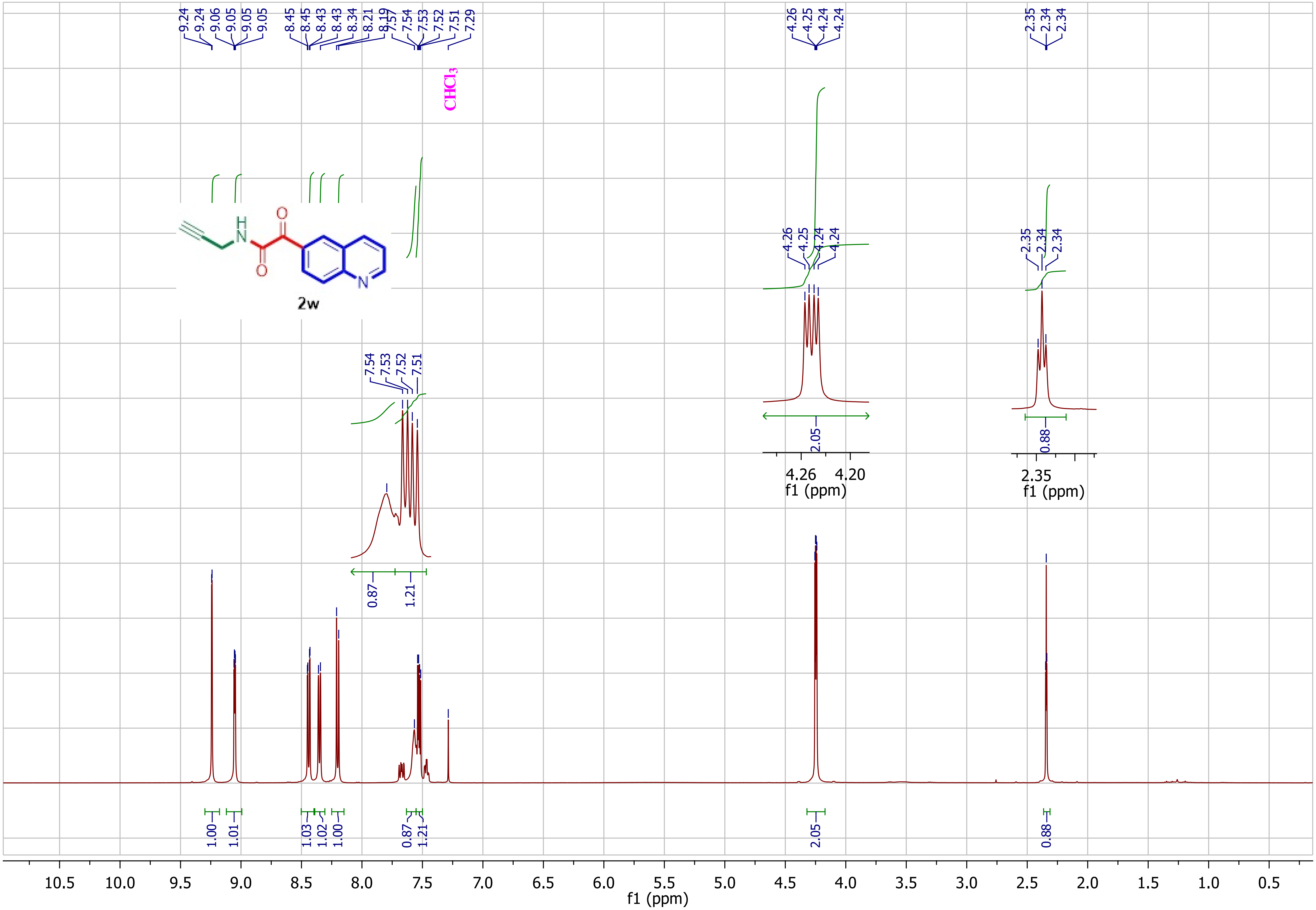
9.24
9.24
9.06
9.05
9.05
9.05
8.45
8.45
8.43
8.43
8.34
8.21
8.19
7.57
7.54
7.53
7.52
7.51
7.29
4.26
4.25
4.24
4.24
2.35
2.34
2.34
$CHCl_3$
2w
0.87
1.21
2.05
0.88
4.26
4.20
2.35
f1 (ppm)
1.00
1.01
1.03
1.02
1.00
10.5
10.0
9.5
9.0
8.5
8.0
7.5
7.0
6.5
6.0
5.5
5.0
4.5
4.0
3.5
3.0
2.5
2.0
1.5
1.0
0.5

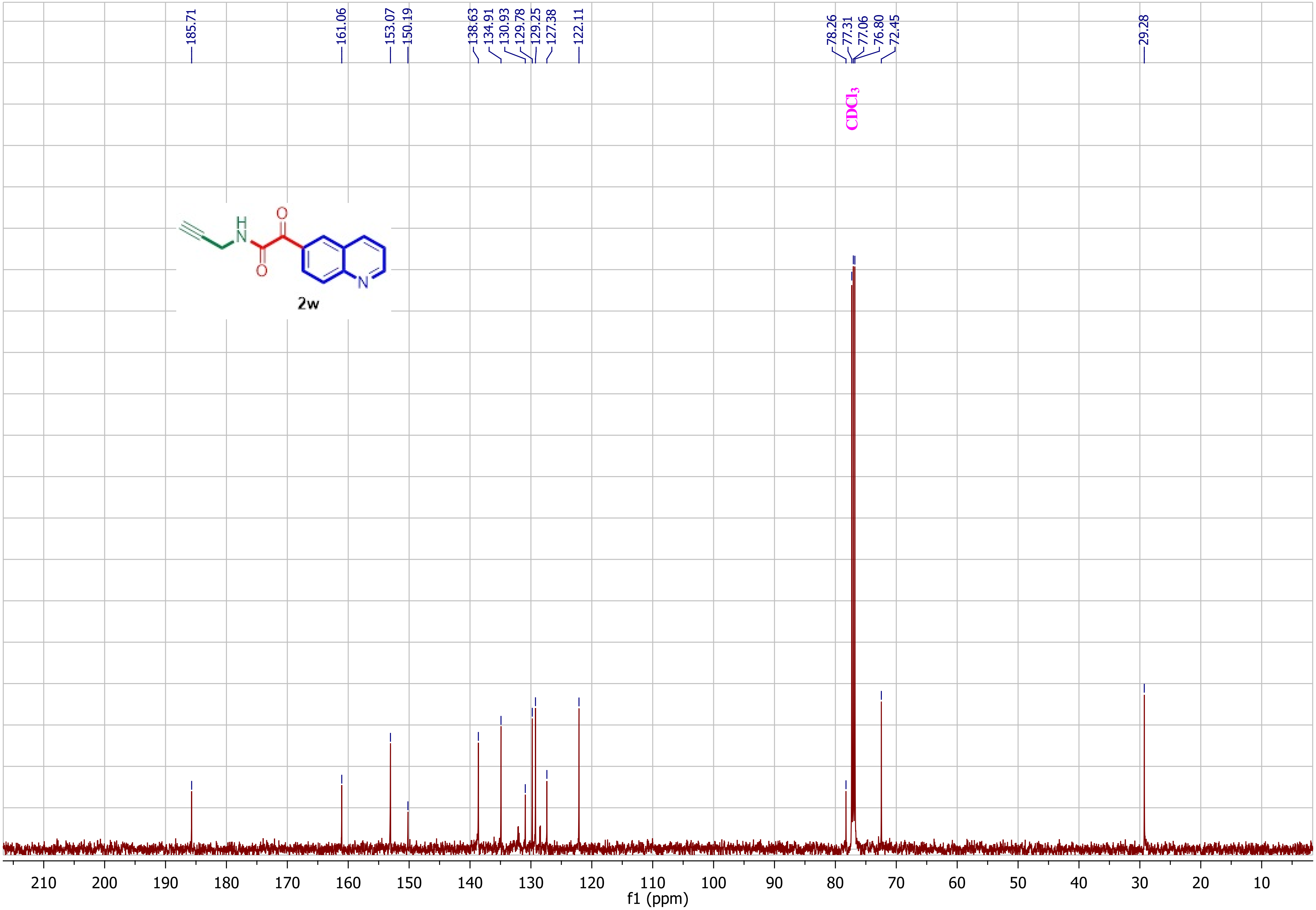

185.71
161.06
153.07
150.19
138.63
134.91
130.93
129.78
129.25
127.38
122.11
78.26
77.31
77.06
76.80
72.45
29.28
CDCl3
H
N
O
O
N
2w
210
200
190
180
170
160
150
140
130
120
110
100
90
80
70
60
50
40
30
20
10
f1 (ppm)
